# Microwave-Assisted Synthesis and Enzyme Stabilization Study of *N*‑Alkyl Praziquantel Analogs for Arylsulfatase B: Possible Leads for Mucopolysaccharidosis VI Therapy

**DOI:** 10.1021/acsomega.5c07075

**Published:** 2026-03-25

**Authors:** Lee S. Cantrell, Bret DeGraaff, Jack Hostetler, Rachel Koentopp, Karna Terpstra, Sarah Weissinger, Sarah M. Zeitler, Trisha A. Russell

**Affiliations:** Department of Chemistry, 7448 Whitworth University, 300 W. Hawthorne Rd., Spokane, Washington 99251, United States

## Abstract

Mutations in the lysosomal enzyme arylsulfatase B result in the genetic disorder mucopolysaccharidosis VI. Current treatment for mucopolysaccharidosis VI requires intravenous enzyme replacement therapy which suffers from incomplete biodistribution. Alternative therapeutic approaches based on small molecules acting as enzyme stabilizers or pharmacological chaperones facilitate the transport of mutant enzymes to the lysosome and may offer improved biodistribution. Herein we describe the development of a facile microwave-assisted reductive amination to synthesize *N*-alkyl substituted analogs of praziquantel. The analogs were then tested for their ability to stabilize wild-type arylsulfatase B against thermal denaturation. We identify one analog that does not inhibit recombinant arylsulfatase B but can stabilize it against thermal denaturation as a potential lead compound in the treatment of mucopolysaccharidosis VI.

## Graphical abstract

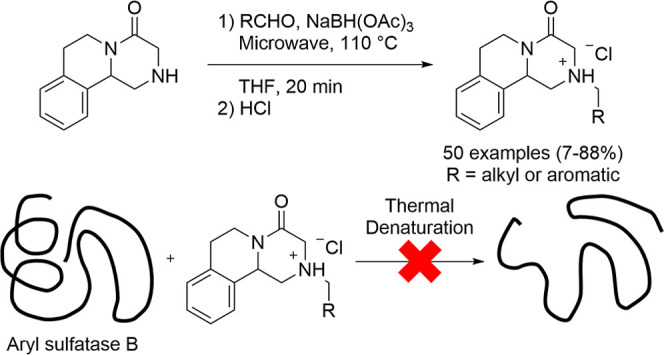

## Introduction

Mucopolysaccharidosis VI (MPS VI), also referred to as Maroteaux-Lamy Syndrome, is a rare but debilitating autosomal recessive lysosomal storage disorder (LSD) caused by mutations of *N*-acetylgalactosamine-4-sulfatase (arylsulfatase B or ARSB, EC 3.1.6.12) resulting in reduced or absent ARSB enzyme activity. Dysfunction of ARSB results in accumulation of glycosaminoglycans (GAGs) in the lysosome leading to cellular misfunction, multisystemic organ damage and skeletal malformation.^1,2^ The current clinical treatment of MPS VI is intravenous enzyme replacement therapy (ERT) with recombinant ARSB.^3^ ERT has been successful in treatment of many of the symptoms of MPS VI, however the biodistribution of recombinant ARSB is incomplete.^4^ The central nervous system, eyes, skeletal structure and joints have little to no resolution of symptoms due to the blood–brain barrier and poor vascularization.^5–8^ The limitations of ERT for treatment of MPS VI creates the need to investigate other treatment options.

Small molecule chaperones (SMC) have recently been investigated as an alternative or supplement to ERT treatments for several LSDs including Fabry disease, Gaucher disease, and Pompe disease.^9–13^ These SMCs have better oral availability and biodistribution than the recombinant enzymes and are classified as pharmacological chaperones (PCs) and enzyme stabilizers (ESs).^10,11^ The PCs bind and stabilize endogenous mutant enzymes to reduce premature degradation and facilitate intracellular trafficking to the lysosome from the endoplasmic reticulum.^14–17^ The increased concentration of rescued mutant enzyme in the lysosome results in clearance of the stored GAGs macromolecules and improved cellular function. Alternatively, ESs can increase the effectiveness of ERT; for example, clinical studies of the tandem use of ESs with ERT in Pompe and Fabry diseases demonstrate that the ES stabilizes the recombinant enzyme and increases its lifetime in circulation.^18,19^ In addition, the combination therapy increases skeletal muscle enzymatic levels with associated improved clinical outcomes in motor and respiratory function outcomes compared to ERT alone.

Currently, most of the SMCs under development act as competitive inhibitors of the target enzyme. Ideally, these SMCs would exhibit stronger inhibition at neutral pH (cytoplasmic environment, pH ≈ 7) than in acidic conditions (lysosomal environment, pH ≈ 5). This pH dependent behavior allows the SMC to dissociate once in the lysosome so that the enzyme regains activity to degrade the built-up substrate. The FDA approved drug migalastat (Fabry disease) demonstrates the potential of using pH-responsive chaperones,^20^ and substantial efforts have been devoted to developing SMCs for other LSDs many of which contain pH-sensitive protonation sites or labile functional groups that change the enzyme affinity, particularly in the context of Gaucher disease.^21–25^ In contrast, some SMCs under development are designed to stabilize but not inhibit the mutant enzymes. These SMCs do not require dissociation prior to substrate turnover since the enzyme remains active throughout intracellular trafficking and lysosomal localization.^26–29^

Our research aims to identify small molecules capable of stabilizing ARSB toward the goal of developing a PC for MPS VI. As of 2025, 225 known mutations of the ARSB gene have been identified, of which 171 are missense or nonsense mutations.^30^ Many of the missense mutations reduce the stability of the ARSB enzyme resulting in incorrect folding and premature enzymatic breakdown by the endoplasmic-reticulum-associated protein degradation pathway before the enzyme is transported into the lysosome.^31–35^ Some of the mutant forms of ARSB still retain residual enzyme activity that could clear stored GAGs if they reach the lysosome.^33^ Therefore, we sought to identify a small molecule that could stabilize ARSB as an initial step toward the development of a SMC for MPS VI.

There are very few reports of small molecules that interact with ARSB. In a search for inhibitors of sulfatase-2 (SUMP 1), a biphenyl ether trichloroethylsulfamate was reported as a micromolar inhibitor of ARSB.^36^ Recently, phenylboronic acid derivatives have been reported as competitive inhibitors for ARSB.^37^ In another study, praziquantel (**1**, PQA), a drug used to treat schistosomiasis, was shown to have a mixed-mode type inhibitory effect on liver ARSB of Schistosoma-infected mice.^38^ The mouse ARSB has 77% identity to human ARSB suggesting that the praziquantel analogs could bind to human ARSB.^39^ Praziquantel has been extensively modified and tested in the search for novel treatments for schistomiasis, so we chose it as the core structure for our studies.^40^ Herein we present a new approach to the synthesis of *N*-alkyl praziquantel analogs and evaluate their ability to stabilize ARSB against thermal denaturation.

## Results and Discussion

### Synthesis of Praziquantel Analogs

We synthesized a variety of N-substituted praziquantel analogs to test for their ability to stabilize human ARSB. Praziquantel (**1**) is readily hydrolyzed to praziquanamine (PQA, **2**) in 2 M HCl which provides access to a common intermediate for the synthesis of *N*-substituted analogs (Scheme 1).^41^ Coupling of **2** with benzoyl chloride readily afforded the *N*-benzoyl PQA analog **3**.^42^

**Scheme 1:**
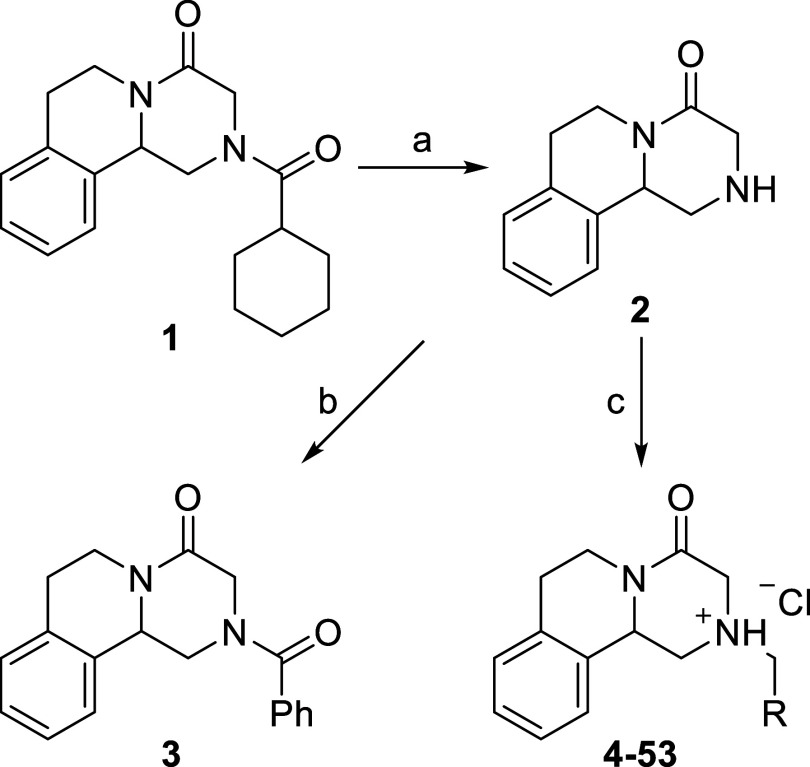
Synthesis of *N*-Substituted Praziquantel Analogs^a^

a Reagents and Conditions: (a) 2 M HCl (aq.), EtOH, reflux, 19 h; (b) PhCOCl, Na_2_CO_3_, 2-MeTHF (c) (1) RCHO, NaBH­(OAc)_3_, THF, MW (800 W), 110 °C, 20 min, (2) HCl (aqueous or anhydrous in EtOAc)

The alkylation of **2** is more difficult. There have been a few reports of *N*-alkyl PQA analogs synthesized through alkylation or reductive amination reactions of **2**, but the reaction times are long (12–72 h).^42,43^ We therefore sought to adapt a microwave-assisted reductive amination route to the *N*-alkyl analogs to accelerate the reaction.^44,45^ The microwave-assisted reductive amination of **2** using sodium triacetoxyborohydride and benzaldehyde in THF at 110 °C gave complete conversion to product within 20 min. The protonated ammonium hydrochloride salts were then easily isolated via extraction or precipitation with anhydrous HCl in ethyl acetate. The isolation of many of the analogs was facilitated by direct extraction of the hydrochloride salt from the acidic aqueous phase with chloroform to afford clean products with no evidence of byproducts.^46^ Subsequent recrystallization of the salts provided the pure materials for the biological assays. This method was successful with a variety of aldehydes to provide the *N*-alkyl PQA analogs (**4–53**, 7–88%, Table 1). The reaction was tolerant of R groups containing alkyl chains (**37**, **38**), pyridyl rings (**41**, **42**) and phenyl rings containing many different functional groups including halogens (**26**–**31**), nitro (**32**–**34**), and hydroxyl groups (**13**, **14**, **22**, **23**). The reaction even worked with the sterically hindered 2,6-dimethylbenzaldehyde (**8**). Conversion of **2** to the *N*-alkyl PQA analogs was generally high, but some of the yields were lower for the isolated hydrochloride products due to the lower crystallinity of those compounds. However, we obtained sufficient material to conduct the biological assays, so we did not focus on increasing the isolated yields.

**Table 1:** Microwave-Assisted Reductive Amination of **2** for the Synthesis of *N*-Alkyl PQA Analogs

compound	R	% yield^a^	compound	R	% yield^a^
4	Ph	25	**29**	2-BrPh	71
5	2-CH_3_Ph	48	**30**	3-BrPh	45
6	3-CH_3_Ph	27	**31**	4-BrPh	88
7	4-CH_3_Ph	68	**32**	2-O_2_NPh	58
8	2,6-(CH_3_)_3_Ph	39	**33**	3-O_2_NPh	33
9	2-EtPh	73	**34**	4-O_2_NPh	51
10	4-EtPh	25	**35**	2-Naphthyl	31
11	4-iPrPh	64	**36**	1-Naphthyl	31
12	4-tBuPh	86	**37**	PhCH_2_CH_2_	37
13	2-HOPh	24	**38**	PhCH_2_	72
14	4-HOPh	23	**39**	3-PhPh	71
15	2-CH_3_OPh	26	**40**	4-PhPh	75
16	3-CH_3_OPh	76	**41**	2-pyridyl	51
17	4-CH_3_OPh	12	**42**	3-pyridyl	18
18	4-PhOPh	43	**43**	2-CF_3_OPh	7
19	3,4-(OCH_2_O)Ph	35	**44**	3-CF_3_OPh	49
20	3,4-(CH_3_O)_2_Ph	56	**45**	4-CF_3_OPh	51
21	4-CH_3_O-2-CH_3_Ph	24	**46**	2-CF_3_Ph	47
22	3,5-(CH_3_O)_2_-4-HOPh	59	**47**	3-CF_3_Ph	27
23	3-CH_3_O-2-HOPh	56	**48**	4-CF_3_Ph	33
24	2-CH_3_O-4,5-(OCH_2_O)Ph	26	**49**	3-F-4-CF_3_Ph	24
25	3-EtO-4-HOPh	56	**50**	2-F-5-CF_3_OPh	42
26	2-FPh	38	**51**	2-CH_3_-4-CF_3_OPh	39
27	3-FPh	14	**52**	2-CH_3_O-4-CF_3_OPh	86
28	4-FPh	29	**53**	3-CF_3_O-4-CH_3_OPh	17

a Isolated yield.

Crystals suitable for X-ray diffraction were grown of **4** by the slow evaporation of a mixture of ethyl acetate and isopropanol. The crystal structure confirms the molecular structure of **4** (Figure 1a). The crystal structure of **4** is disordered with two different conformations of the 6-membered ring attached to the benzene in the praziquanamine core with refined occupancy factors of 0.644(8) and 0.356(8) (Figure 1b). The major (C5–C11) disordered component of the 6-membered ring resembles a boat conformation while the minor (C5a–C11a) disordered component adopts a half-chair conformation. The rac-PZQ also crystallizes as a mixture of conformers however the half-chair conformation is favored.^47^ The ^1^H NMR spectra for the alkyl portions of the praziquanamine core of the PQA analogs have broad peaks due to this conformational flexibility.

**Figure 1:**
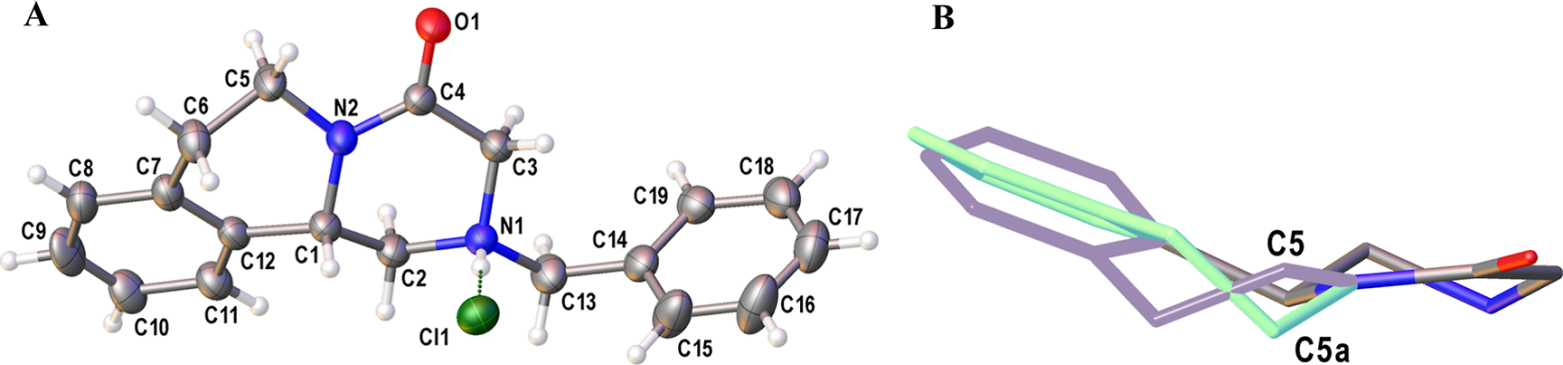
Crystal structure of PQA analog **4** (CCDC-2531889). (A) Molecular structure of PQA analog **4**. Displacement ellipsoids at a 30% probability and hydrogen atoms are drawn as arbitrary radius spheres. Only the major component of disorder is shown for clarity. (B) Conformations of praziquanamine core structure with benzyl substituent, chlorine and hydrogen atoms omitted for clarity. Boat conformation shown in green and half-chair conformation shown in lilac.

### Biological Evaluation

We then turned our attention to the ARSB enzyme activity assays. To act as a SMC, the molecule will need to stabilize the enzyme in either the endoplasmic reticulum as a PC or in the bloodstream as an ES. Two standard sulfate assays are reported, one in pH 5.0 NaOAc buffer and the other in pH 6.5 MES buffer. In the assay, ARSB removes the sulfate from *p*-nitrocatechol sulfate and the enzyme activity is measured via absorbance at 515 nm to determine the amount of *p*-nitrocatechol formed. We tested the activity in both buffers and found the amount of product formed after 1 h in the pH 6.5 MES buffer to be higher than the pH 5.0 NaOAc buffer (Figure S1).^48,49^ This was unexpected given that ARSB is a lysosomal enzyme with a reported pH optimum of 5.7 in NaOAc.^50^ However, it was fortunate since the endoplasmic reticulum and the bloodstream are both closer to neutral pH or even slightly alkaline, and we wanted to study the enzyme closer to those conditions to evaluate their potential as SMCs.^51^ We scaled and adapted the assay in MES buffer at pH 6.5 to a 96-well polypropylene plate to facilitate throughput of the samples.

In the initial screen of PQA and its analogs, PQA (**1**) and praziquanamine (**2**) appeared to be slight inhibitors of recombinant ARSB (rhARSB, Figure 2A, white bars). The *N*-benzoyl PQA analog (**3**) did not have any inhibitory effect, but the *N*-benzyl PQA analog (**4**) was a strong inhibitor. We then screened additional *N*-benzyl PQA analogs under these conditions and found some unusual effects. Specifically, several of the compounds appeared to be strong activators of rhARSB (**5** and **6**) whereas others were strong inhibitors (**7**–**9**). Eventually, we tested the same compounds using a set of 96 well plates coated with a nonbinding surface (Figure 2A, gray bars). Using the new plates, the enzyme activity normalized and there was little effect based on different PQA analogs. Comparison of the absorbance at 515 nm between the standard and nonbinding polypropylene plates showed that the rhARSB produced more product in the nonbinding plates and therefore was more active (Figure 2B). Analogs **12**–**42** showed similar trends between the two plates (Figure S2). These results lead us to conclude that the PQA analogs were not acting as strong activators or inhibitors of rhARSB but instead stabilized the enzyme in solution and prevented aggregation against the polypropylene plate walls.

**Figure 2:**
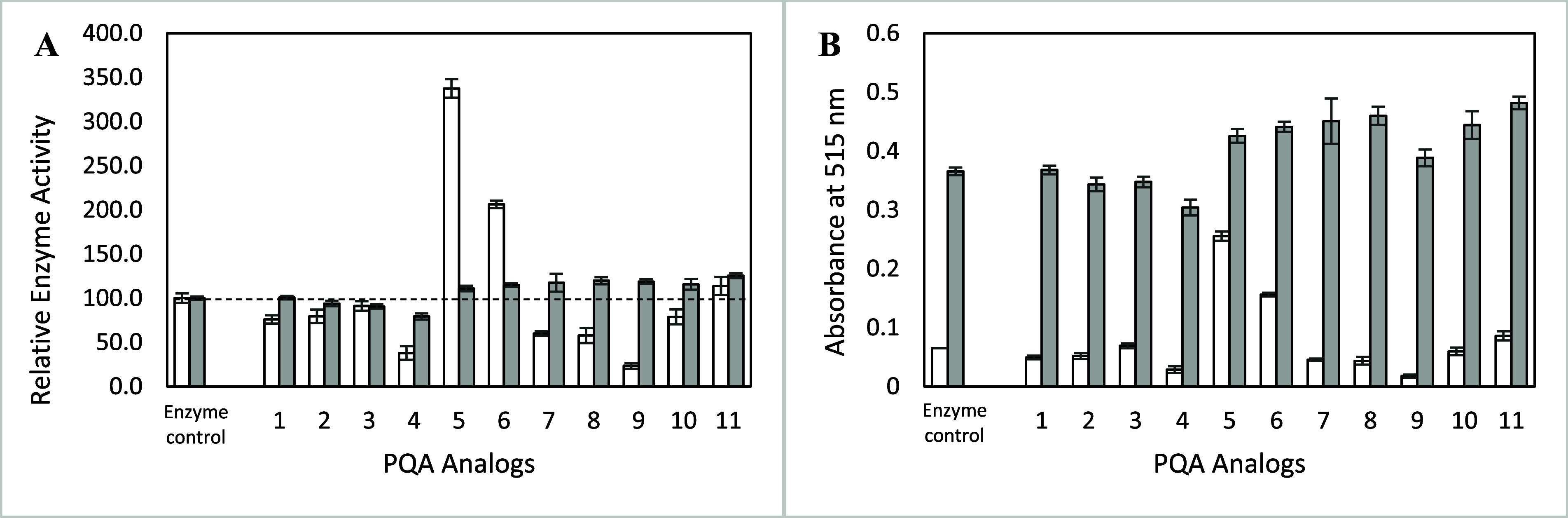
Screening of several PQA analogs as inhibitors of rhARSB. Enzyme activity of rhARSB (0.5 μg/mL) in the presence of PQA analogs (0.5 mM, added as a solution in DMSO) in pH 6.5 MES buffer. White bars represent the assays conducted in standard polypropylene 96 well plates. Gray bars represent the assays conducted in polypropylene 96 well plates coated with a nonbinding surface. (A) Relative enzyme activity of rhARSB. The *y*-axis is reported as the relative enzyme activity compared to rhARSB in the enzyme control with only DMSO added and the dashed line indicates the enzyme only control. (B) Absolute enzyme activity of rhARSB. The *y*-axis is the absorbance at 515 nm. Data are expressed as a mean ± standard deviation of triplicate assays.

Although most SMCs currently being studied are inhibitors of the target enzyme, they do not have to be inhibitors as shown by previous studies on other LSDs.^27,28,52,53^ Therefore, we sought to screen the praziquantel analogs for their ability to stabilize rhARSB, so we developed an assay to measure the residual enzyme activity after heat-induced denaturation of the enzyme.^27^ First, we incubated rhARSB to denature the enzyme at 50 °C and then incubated the same sample with *p*-nitrocatechol sulfate for an additional hour at 37 °C to determine the remaining enzyme activity. The enzyme activity of each time point was compared to a nonheated enzyme activity control that was kept on ice during the heat denaturation step. When tested alone, the rhARSB steadily lost most of its activity when heated over the course of an hour (Figure 3).

**Figure 3:**
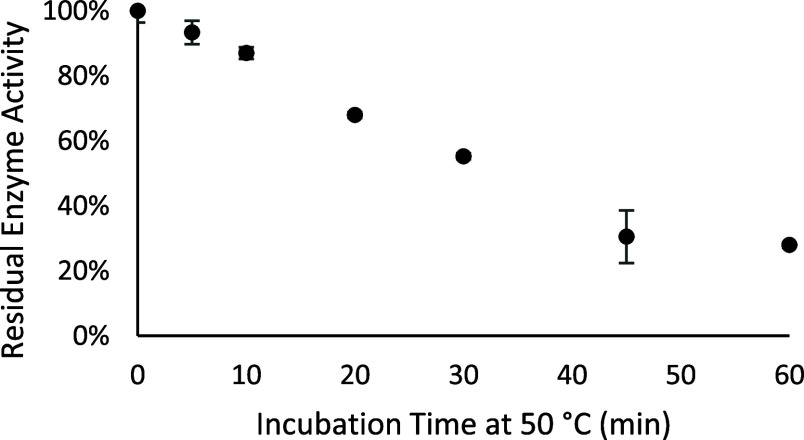
Heat-induced denaturation of rhARSB over time in pH 6.5 MES buffer in nonbinding 96-well plates. The *y*-axis is reported as the percent remaining activity calculated by dividing the enzyme activity of the heated sample by the enzyme activity of the samples kept on ice. Data are expressed as a mean ± standard deviation of triplicate assays.

Next, we tested the ability of the PQA analogs to stabilize rhARSB against heat-induced denaturation by heating a plate containing wells of the enzyme and a PQA analog in pH 6.5 MES buffer to 50 °C for 45 min followed by an assay of the residual enzyme activity at 37 °C. The enzyme activity of each well was then compared to corresponding wells of enzyme and PQA analog kept on ice during the thermal denaturation step to obtain the fraction of residual enzyme activity (Figure 4). The wells containing PQA analogs with exocyclic amide substituents (**1** and **3**) and the nonsubstituted praziquanamine (**2**) showed similar reductions in the residual enzyme activity to the control well, indicating that they did not stabilize rhARSB. In contrast, many of the PQA analogs containing exocylic amine substituents (**4**–**53**) retained more enzyme activity, indicating that they stabilized rhARSB against the heat-induced denaturation. In particular, PQA analogs containing an *N*-benzyl group with a substituent in the ortho position of the benzene ring had the highest ability to stabilize rhARSB. Two of the PQA analogs, 2-CH_3_PhCH_2_PQA (**5**) and 2-F-5-CF_3_OPhCH_2_PQA (**50**), strongly stabilized rhARSB against thermal denaturation. These results correlated with the initial results found in the regular polypropylene plates versus those coated with a nonbinding surface (Figure 2a). Specifically, the rhARSB had the highest activity in the presence of **5** in the regular polypropylene plate suggesting that **5** did stabilize rhARSB against aggregation as well as thermal denaturation.
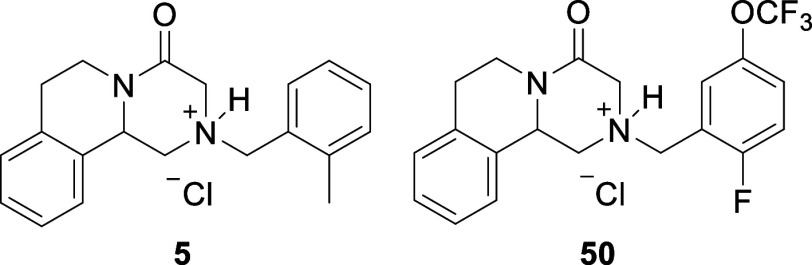


**Figure 4:**
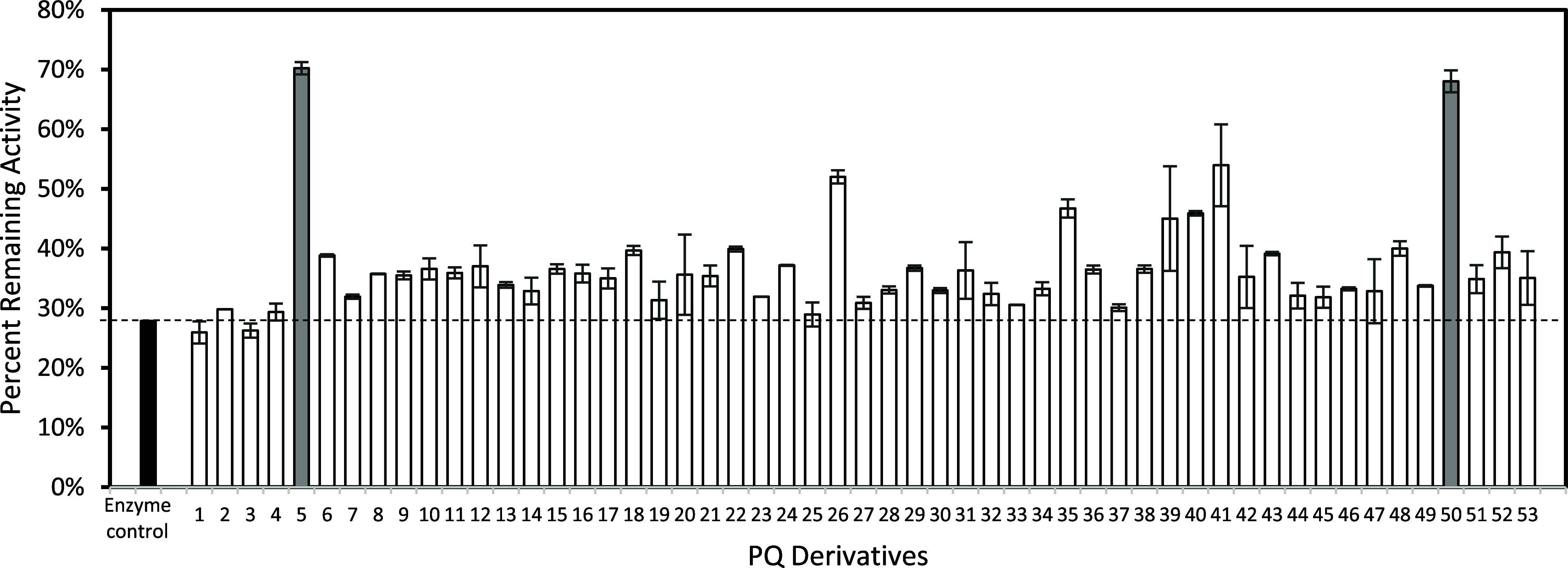
Screening of PQA analogs as potential PC for MPS VI using thermal stability assays. Thermal stability assay of rhARSB (0.24 μg/mL) in the presence of 55 PQA analogs (**1–53**, 400 μM, added as a solution in DMSO) in pH 6.5 MES buffer in nonbinding 96-well plates. The *y*-axis is reported as the percent remaining activity calculated by dividing the enzyme activity of the heated sample by the enzyme activity of the samples kept on ice. Black bar is enzyme control with only DMSO added. Gray bars represent the most stabilizing compounds. Data are expressed as a mean ± standard deviation of duplicate assays.

Secondary screening of compounds **5** and **50** tested their ability to stabilize rhARSB against thermal denaturation at additional concentrations (Figure 5). Analog **5** maintained the ability to stabilize rhARSB at lower concentrations, so further evaluation focused on **5**. To begin, rhARSB was incubated at 50 °C for various time intervals and then the residual enzyme activity was tested. The percent remaining activity of rhARSB decreased steadily over 60 min with a loss 60% of the enzyme activity after 60 min (Figure 6A). The addition of **5** to the assay resulted in a dose-dependent stabilization of rhARSB. At the highest concentration, **5** prevented most of the enzyme denaturation and only 15% of the enzyme activity was lost at 60 min.

**Figure 5:**
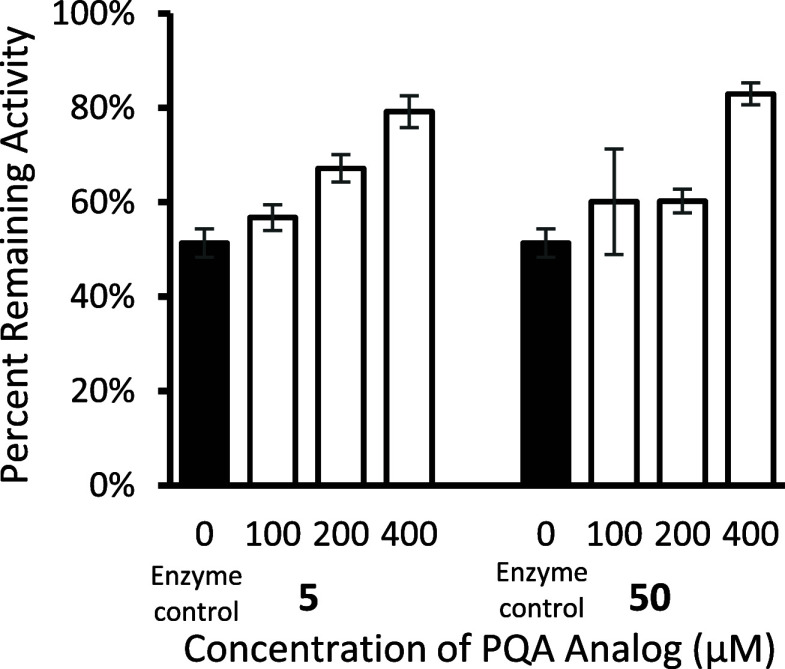
Thermal stability assay of rhARSB (0.24 μg/mL) versus concentration of PQA analogs **5** and **50** in nonbinding 96-well plates. The *y*-axis is reported as the percent remaining enzyme activity calculated by dividing the enzyme activity of the sample heated for 60 min by the enzyme activity of the samples kept on ice. Data are expressed as a mean ± standard deviation of duplicate assays.

**Figure 6:**
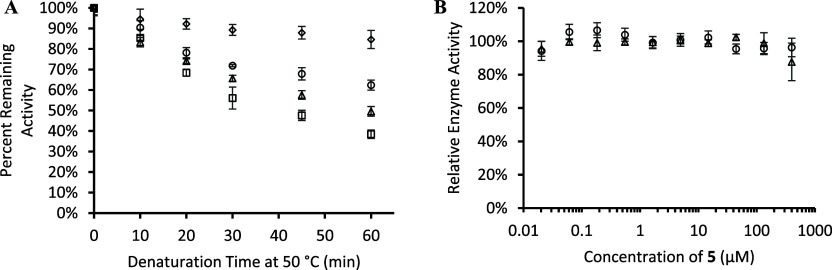
Evaluation of **5** against rhARSB for thermal stabilization and inhibition in nonbinding 96-well plates. (A) Thermal denaturation assay of rhARSB (0.24 μg/mL) with **5** (Enzyme only control, squares; 0.1 mM **5** triangles; 0.2 mM **5** circles; 0.4 mM **5** diamonds). (B) Enzyme activity assay of rhARSB with **5** (0.4 mM; pH 5.0 NaOAc, triangles; pH 6.5 MES, circles). Data are expressed as a mean ± standard deviation of triplicate assays.

Finally, we verified that PQA analog **5** did not inhibit rhARSB enzyme activity in a standard inhibition assay. So far, the enzyme activity and enzyme denaturation assays have been at pH 6.5 to mimic the environment of the endoplasmic reticulum. However, ARSB is a lysosomal enzyme, so it also needs to remain active at lysosomal pH levels (4.7).^51^ We therefore tested rhARSB enzyme activity in the presence of **5** at pH 6.5 MES and at pH 5.0 NaOAc (Figures 6B and S3). The activity of the enzyme remained around 100%, so PQA analog **5** was not found to be an inhibitor at either pH value. This contrasts with the mode of action of most of the other SMC candidates for other LSDs that are competitive inhibitors of the target enzyme and rely on binding constant changes in the more acidic lysosome to dissociate the inhibitor and regain enzymatic activity.^9^

In summary, we have shown an efficient manner for the production and purification of various *N*-alkyl PQA analogs via microwave-assisted reductive amination. One of these *N*-alkyl PQA analogs strongly stabilizes rhARSB against thermal denaturation and yet does not inhibit the enzyme activity at pH 5.0 or 6.5. The fact that **5** stabilizes rhARSB against denaturation demonstrates that there is the potential for the development of a SMC for the treatment of MPS VI.

## Experimental Section

### Chemistry

#### General Methods

All Chemicals were reagent grade from Alfa Aesar, Fluka, Fisher, Matrix Scientific, RAD Systems, Sigma-Aldrich, and TCI and used without further purification except where noted. Praziquantel was purchased from Carbosynthesis Limited. Deionized water was used for all synthetic procedures. THF was dried with activated 4 Å molecular sieves for at least 12 h prior to use. All ^1^H NMR spectra were recorded on a Jeol ECS-400 MHz spectrometer in DMSO-*d*_6_ (with DMSO as a standard at 2.50 ppm) or CDCl_3_ (with TMS as a standard at 0 ppm). All ^13^C NMR spectra were acquired at 100 MHz in DMSO-*d*_6_ (with DMSO-*d*_6_ as a standard at 39.51 ppm) or CDCl_3_ (with CDCl_3_ as a standard at 77.0 ppm). Coupling constants are reported in Hertz (Hz). Abbreviations are used as follows: s = singlet, d = doublet, t = triplet, q = quartet, hept = heptet, m = multiplet, dd = doublet of doublet and dt = doublet of triplet. The microwave reactions were run on a CEM Mars 6 Microwave Reaction System with a 20-vessel set. Column chromatography was carried out on a Varian 971-FP automated purification system using refillable cartridges (Thomson) using 60 Å silica gel (Acros Organics) with a 1 mL/min flow rate.

### Compound Synthesis

Praziquanamine (**2**) and N-benzoylpraziquanamine (**3**) were prepared according to literature procedures.^42,43,54^

### General Reductive Amination Procedure

To a solution of praziquanamine (0.1734 g, 0.857 mmol), and a benzaldehyde derivative (1.285 mmol) in dried THF (3 mL) in a glass microwave pressure vessel with a stir bar, was added sodium triacetoxyborohydride (0.257 g, 1.2 mmol). The vessel was capped and the resulting solution was stirred and heated in a CEM Mars 6 microwave to 110 °C over 3 min and then held for 20 min. The reaction was quenched with NaOH (10 mL, 1 M, aq.) and extracted with ethyl acetate (3×, 5 mL). The organic layer was isolated and dried with magnesium sulfate. Ethereal HCl or HCl in ethyl acetate was added (1 mol equiv) to obtain the hydrochloride salt of the PQA derivative.^55^ Alternative work up procedures are indicated for each compound as necessary.

### 2-(2-Methylbenzyl)-4-oxo-1,3,4,6,7,11*b*-hexahydro-2*H*-pyrazino­[2,1-*a*]­isoquinolin-2-ium Chloride (**5**)

**5** was synthesized according to the general reductive amination procedure with praziquanamine (0.1734 g, 0.857 mmol), sodium triacetoxyborohydride (0.257 g, 1.2 mmol), and *o*-tolualdehyde (0.15 mL, 1.3 mmol), resulting in a pale-yellow solid upon addition of ethereal HCl. This solid was recrystallized in isopropanol to yield **5** as a white solid (0.1482 g, 48%). ^1^H NMR (400 MHz, DMSO-*d*_6_): δ 7.68 (1H, d), 7.7.39–7.22 (7H, m), 5.41 (1H, br d), 4.59–4.45 (2H, m), 4.45–4.20 (2H, br s), 4.00–3.82 (1H, br s), 3.57 (1H, d) 3.4 (1H, br s), 2.91–2.74 (3H, m), 2.47 (3H, s). ^13^C NMR (100 MHz, DMSO-*d*_6_): δ 160.59, 138.65, 134.67, 132.09, 130.95, 129.65, 129.31, 127.39, 126.78, 126.17, 124.92, 61.98, 56.38, 52.50, 51.57, 38.06, 27.93, 19.54.

Complete information about the synthesis of all novel PQA derivatives can be found in the Supporting Information.

### Single Crystal Growth

Crystals of **4** suitable for single-crystal X-ray diffraction were grown by slow evaporation of a solution in a mixture of ethyl acetate and isopropanol at room temperature.

### Single Crystal X-ray Diffraction and Structural Refinement

The diffraction data on single crystals were collected at 298 K using a Bruker D8 Venture IμS microfocus dual-source diffractometer equipped with a PHOTON II CPAD detector and an Oxford cryogenic system. Monochromatic Cu Kα (λ = 1.54178 Å) radiation was used in the data collection using phi (φ) and omega (ω) scan strategies. Cell measurement, data collection, integration, scaling, and absorption correction were performed using SADABS programs incorporated in APEX6.^56^ The structure was solved using SHELXT^57^ and refined using SHELXL^58^ programs, both implemented in OLEX2.^59^ The non-hydrogen atoms were located in successive difference Fourier syntheses and refined with anisotropic thermal parameters. The CH hydrogen atoms were placed at calculated positions and refined using a riding model with appropriate HFIX commands and *U*_iso_ = 1.2 × *U*_eqiv_. NH hydrogen atom was located in different Fourier syntheses (NH, 1.2 × *U*_eqiv_), and their positions were refined independently. The program OLEX2 was used for molecular images. Crystallographic data are summarized in Table S1. Crystallographic data for the crystal structure has been deposited with the Cambridge Crystallographic Data Centre as supplementary publication no. CCDC 2531889. Copies of the data can be obtained free of charge on application to CCDC, 12 Union Road, Cambridge CB2 1EZ, U.K. (fax (+44)­1223–336–033; e-mail: deposit@ccdc.cam.ac.uk; www: http://www.ccdc.cam.ac.uk).

### Biology

#### General

Recombinant arylsulfatase B (ARSB) was purchased from R&D Systems. Milli-Q system (MilliporeSigma) ultrapure water was used for enzymatic assays. Enzymatic assays were screened using a ELx808 Absorbance Microplate Reader (BioTek).

### Enzyme Assay Protocol

Compounds were screened for enzyme activity in a 96-well flat bottom polystyrene clear plate (Corning, low binding). A 50 μL reaction mixture containing ARSB (0.235 μg/mL), PQA analog (0.4 mM dissolved in 2 μL DMSO; final concentration of DMSO in reaction = 2%) and 50 mM MES buffer (pH 6.5) was incubated on ice for 50 min. Substrate solution containing 4-nitrocatechol sulfate (2.5 mM) in 50 mM MES buffer (50 μL, pH 6.5) was added to the reaction mixture and the plates were incubated at 37 °C for 1 h. The reaction was quenched with the addition of aqueous NaOH (150 μL, 0.2 M). The well plates were centrifuged at 2500 rpm and 200 μL of the solution was transferred to a clean well plate. The absorbance was read at 515 nm and analyzed using Gen5 data analysis software. Blank runs containing no enzyme or PQA analogs were used to determine background noise. Control enzyme activity was determined using only DMSO (2 μL). Relative enzyme activity was determined by dividing the enzyme activity of samples containing the PQA analogs by the control enzyme activity and multiplying by 100.

### Thermal Denaturation Enzyme Assay Protocol

Thermal denaturation assay was run the same as the enzyme assay protocol except that the plates were incubated on ice for 5 min followed by incubation at 50 °C for 45 min prior to the substrate solution addition. Percent remaining enzyme activity was determined by dividing the enzyme activity of the samples by the enzyme activity of the samples that remained on ice.

### Statistical Analysis

All biological assay data are expressed as means ± standard deviations, based on duplicate or triplicate trials as indicated.

## Supplementary Material



## Data Availability

All data supporting the findings of this study are included within the manuscript and its Supporting Information files.

## Notes

Testing rhARSB activity in different buffers, raw enzyme data for enzyme activity assay of rhARSB with 5, general methods, enzyme assay protocol, thermal denaturation enzyme assay protocol, statistical analysis, compound synthesis, general reductive amination procedure, praziquantel analog synthesis, single-crystal X-ray crystallography, NMR spectra for synthesized compounds (PDF)
