# Supplementary material for: Microwave-Assisted Synthesis and Enzyme Stabilization Study of N‑Alkyl Praziquantel Analogs for Arylsulfatase B: Possible Leads for Mucopolysaccharidosis VI Therapy
Source: ACS Omega. 2026 Mar 25;11(13):20106–14. doi: 10.1021/acsomega.5c07075 (PMC13063022; doi:10.1021/acsomega.5c07075)
Supplement: Supplementary file 1 [file ao5c07075_si_001.pdf]

Microwave-Assisted Synthesis and Enzyme Stabilization Study of *N*-Alkyl Praziquantel Analogs for Arylsulfatase B: Possible Leads for Mucopolysaccharidosis VI Therapy

Cantrell, Lee S.; DeGraaff, Bret; Hostetler, Jack; Koentopp, Rachel; Terpstra, Karna; Weissinger, Sarah; Zeitler, Sarah M.; Russell, Trisha A.*

Department of Chemistry, Whitworth University, 300 W. Hawthorne Rd., Spokane, Washington 99251, United States

Corresponding author: *trisharussell@whitworth.edu

Supplemental Information

Table of Contents

Supplemental Figures....................2
Supplemental Figure 1. Testing rhARSB activity in different buffers. 2
Supplemental Figure 3. Raw enzyme data for enzyme activity assay of rhARSB with 5. 2
General Methods....................3
Biology....................3
Enzyme Assay Protocol....................3
Thermal Denaturation Enzyme Assay Protocol....................3
Statistical Analysis....................3
Chemistry....................4
Compound Synthesis....................4
General Reductive Amination Procedure....................4
Praziquantel Analog Synthesis....................4
Single-Crystal X-ray Crystallography....................26
References....................27
NMR Spectra for Synthesized Compounds....................29

Supplemental Figures

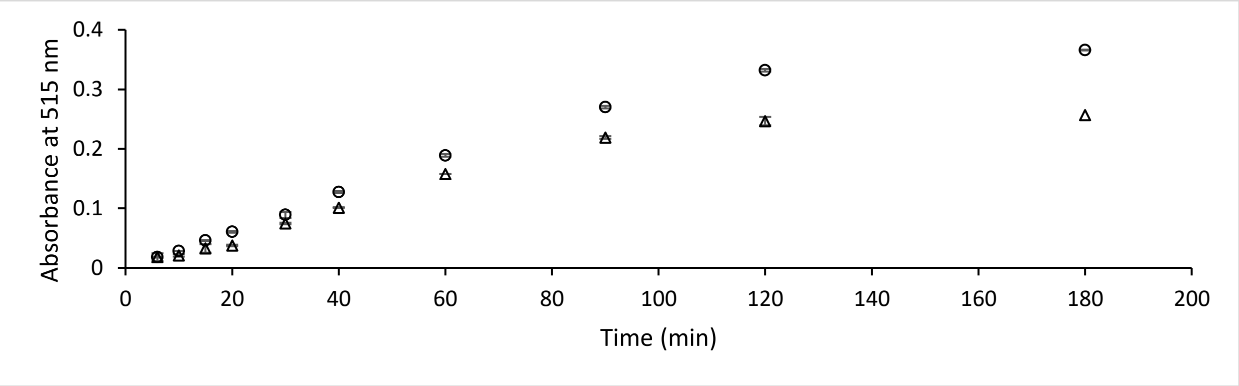

Supplemental Figure 1. Testing rhARSB activity in different buffers. Enzyme activity assay of rhARSB (0.24 µg/mL) in buffer (*circles*, pH 6.5 MES; *triangles,* pH 5.0 NaOAc) conducted in polypropylene 96 well plates coated with a non-binding surface. Data are expressed as a mean ± standard deviation of duplicate assays.

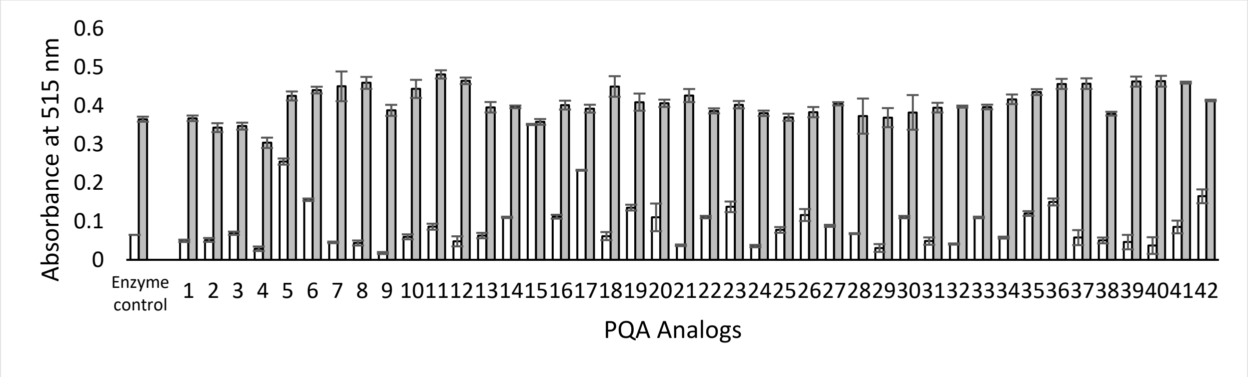

Supplemental Figure 2. Screening of several PQA analogs as inhibitors of rhARSB. Enzyme activity of rhARSB (0.5 µg/mL) in presence of PQA analogs (0.5 mM, added as a solution in DMSO) in pH 6.5 MES buffer. White bars represent the assays conducted in standard polypropylene 96 well plates. Gray bars represent the assays conducted in polypropylene 96 well plates coated with a non-binding surface. Compounds 43-53 were not synthesized at the time of the assay. The y-axis is the absorbance at 515 nm. Data are expressed as a mean ± standard deviation of triplicate assays.

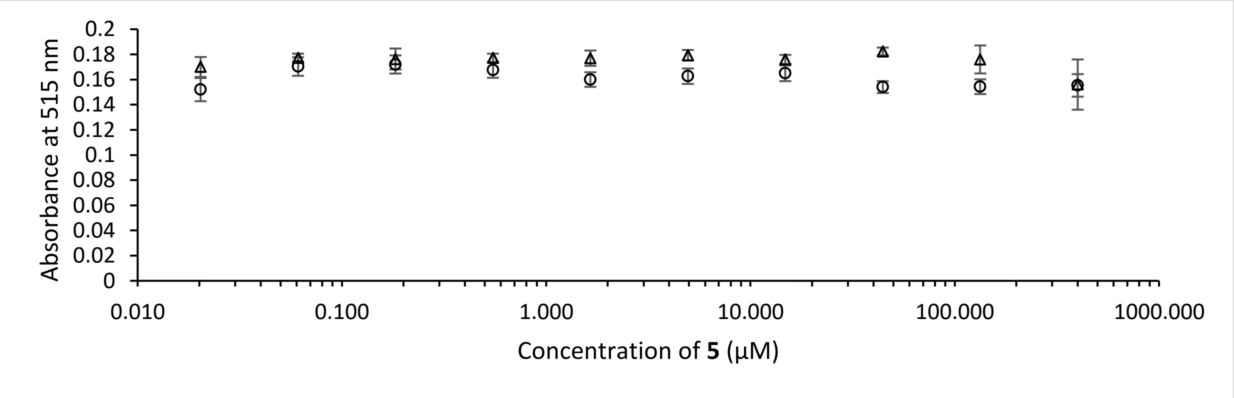

Supplemental Figure 3. Raw enzyme data for enzyme activity assay of rhARSB with 5. Raw Enzyme activity assay of rhARSB (0.24 µg/mL) in buffer (*circles*, pH 6.5 MES; *triangles,* pH 5.0 NaOAc) conducted in polypropylene 96 well plates coated with a non-binding surface. Data are expressed as a mean ± standard deviation of triplicate assays.

General Methods

All Chemicals were reagent grade from Alfa Aesar, Fluka, Fisher, Matrix Scientific, RAD Systems, Sigma-Aldrich, and TCI and used without further purification except where noted. Praziquantel was purchased from Carbosynthesis Limited. Deionized water was used for all synthetic procedures. THF was dried with activated 4 Å molecular sieves for at least 12 h prior to use.

All ^{1}H NMR spectra were recorded on a Jeol ECS-400 MHz spectrometer in DMSO-d_6 (with DMSO as a standard at 2.50 ppm) or $CDCl_3$ (with TMS as a standard at 0 ppm). All ^{13}C NMR spectra were acquired on a 100 MHz in DMSO-d_6 (with DMSO-d_6 as a standard at 39.51 ppm) or $CDCl_3$ (with $CDCl_3$ as a standard at 77.0 ppm). Coupling constants are reported in Hertz (Hz). Abbreviations are used as follows: s = singlet, d = doublet, t = triplet, q = quartet, hept = heptet, m = multiplet, dd = doublet of doublet and dt = doublet of triplet. The microwave reactions were run on a CEM Mars 6 Microwave Reaction System with a 20-vessel set. Column chromatography was carried out on a Varian 971-FP automated purification system using refillable cartridges (Thomson) using 60 Å silica gel (Acros Organics) with a 1 mL/min flow rate. Recombinant arylsulfatase B (ARSB) was purchased from R&D Systems. Milli-Q system (MilliporeSigma) ultrapure water was used for enzymatic assays. Enzymatic assays were screened using a ELx808 Absorbance Microplate Reader (BioTek).

Biology

Enzyme Assay Protocol

Compounds were screened for enzyme activity in a 96-well flat bottom polystyrene clear plate (Corning, low binding). A 50 µL reaction mixture containing ARSB (0.235 µg/mL), PQA analog (0.4 mM dissolved in 2 µL DMSO; final concentration of DMSO in reaction = 2%) and 50 mM MES buffer (pH 6.5) was incubated on ice for 50 min. Substrate solution containing 4-nitrocatechol sulfate (2.5 mM) in 50 mM MES buffer (50 µL, pH 6.5) was added to the reaction mixture and the plates were incubated at 37 °C for 1 h. The reaction was quenched with the addition of aqueous NaOH (150 µL, 0.2 M). The well plates were centrifuged at 2500 rpm and 200 µL of the solution was transferred to a clean well plate. The absorbance was read at 515 nm and analyzed using Gen5 data analysis software. Blank runs containing no enzyme or PQA analogs were used to determine background noise. Control enzyme activity was determined using only DMSO (2 µL). Relative enzyme activity was determined by dividing the enzyme activity of samples containing the PQA analogs by the control enzyme activity and multiplying by 100.

Thermal Denaturation Enzyme Assay Protocol

Thermal denaturation assay was run the same as the enzyme assay protocol except that the plates were incubated on ice for 5 min followed by incubation at 50 °C for 45 min prior to the substrate solution addition. Percent remaining enzyme activity was determined by dividing the enzyme activity of the samples by the enzyme activity of the samples that remained on ice.

Statistical Analysis

All biological assay data are expressed as means ± standard deviations, based on duplicate or triplicate trials as indicated.

Chemistry

Compound Synthesis

Praziquanamine (**2**) and *N*-benzoylpraziquanamine (**3**) were prepared according to literature procedures.[1,2,3]

General Reductive Amination Procedure

To a solution of praziquanamine (0.1734 g, 0.857 mmol), and a benzaldehyde derivative (1.285 mmol) in dried THF (3 mL) in a glass microwave pressure vessel with a stir bar, was added sodium triacetoxyborohydride (0.257 g, 1.2 mmol). The vessel was capped and the resulting solution was stirred and heated in a CEM Mars 6 microwave to 110 °C over 3 min and then held for 20 min. The reaction was quenched with NaOH (10 mL, 1 M, aq.) and extracted with ethyl acetate (3x, 5 mL). The organic layer was isolated and dried with magnesium sulfate. Ethereal HCl or HCl in ethyl acetate was added (1 mol equiv.) to obtain the hydrochloride salt of the PQA derivative.[4] Alternative work up procedures are indicated for each compound as necessary.

Praziquantel Analog Synthesis

2-(Benzyl)-4-oxo-1,3,4,6,7,11*b*-hexahydro-*2H*-pyrazino[2,1-a]isoquinolin-2-ium Chloride (4).

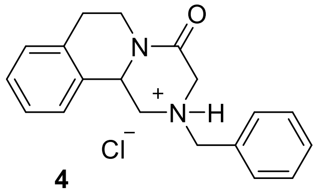

4

4 was synthesized according to the general reductive amination procedure with praziquanamine (0.2019 g, 1.00 mmol), sodium triacetoxyborohydride (0.2967 g, 1.4 mmol), and benzaldehyde (0.14 mL, 1.4 mmol), After washing the organic layer with NaOH, the organic layer was extracted with 6 M HCl (3X, 2 mL). The combined acidic aqueous layers were washed with EtOAc (1X, 2 mL) and then the ammonium chloride salt was extracted with chloroform (3X, 2 mL). The combined organic layers were dried over $MgSO_4$, filtered and concentrated under reduced pressure to form a red oil that was treated with isopropanol/ethyl acetate to grow pale yellow crystals of **4** (0.0891 g, 25%). ^{1}H NMR (400 MHz, DMSO-*d6*) δ 7.68-7.64 (2H, m), 7.50-7.46 (3H, m), 7.31-7.21 (4H, m), 5.41 (1H, br d), 4.59-4.45 (2H, m), 4.58-4.49 (2H, br m), 4.48-4.26 (2H, br s), 3.84 (1H, d), 3.56 (2H, br d) 3.23 (1H, br t), 2.94-2.74 (3H, m). ^{13}C NMR (100 MHz, DMSO-*d6*) δ 160.51, 134.78, 132.00, 131.48, 129.68, 129.34, 128.89, 127.44, 126.83, 125.01, 58.48, 52.20, 51.54, 50.86, 38.06, 27.91.

2-(2-Methylbenzyl)-4-oxo-1,3,4,6,7,11*b*-hexahydro-*2H*-pyrazino[2,1-a]isoquinolin-2-ium Chloride (5).

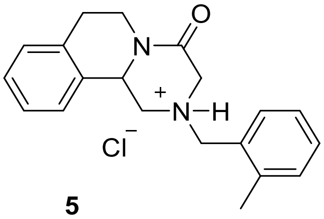

5

5 was synthesized according to the general reductive amination procedure with praziquanamine (0.1734 g, 0.857 mmol), sodium triacetoxyborohydride (0.257 g, 1.2 mmol), and *o*-tolualdehyde (0.15 mL, 1.3 mmol), resulting in a pale-yellow solid upon addition of ethereal HCl. This solid was recrystallized in isopropanol to yield **5** as a white solid (0.1482 g, 48%). ^{1}H NMR (400 MHz, DMSO-d_6) δ 7.68 (1H, d), 7.39-7.22 (7H, m), 5.41 (1H, br d), 4.59-4.45 (2H, m), 4.45-4.20 (2H, br s), 4.00-3.82 (1H, br s), 3.57 (1H, d) 3.4 (1H, br s), 2.91-2.74 (3H, m), 2.47 (3H, s). ^{13}C NMR (100 MHz, DMSO-d_6) δ 160.59, 138.65, 134.67, 132.09, 130.95, 129.65, 129.31, 127.39, 126.78, 126.17, 124.92, 61.98, 56.38, 52.50, 51.57, 38.06, 27.93, 19.54. HRMS (ESI/Q-TOF) m/z: $[M + H]^+$ Calcd for $C_{20}H_{23}N_2O$ 307.1810; Found 307.1810.

2-(3-Methylbenzyl)-4-oxo-1,3,4,6,7,11*b*-hexahydro-*2H*-pyrazino[2,1-a]isoquinolin-2-ium Chloride (6).

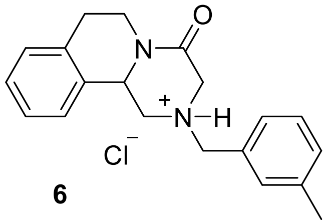

6

6 was synthesized according to the general reductive amination procedure with praziquanamine (0.2069 g, 1.02 mmol), 3-tolualdehyde (0.16 mL, 1.4 mmol) and sodium triacetoxyborohydride (0.3016 g, 1.42 mmol). The addition of 2 M HCl to the ethyl acetate layer in the extraction formed a solid, which was vacuum filtered and recrystallized with isopropanol to give **6** as an iridescent, white powder (0.093g, 27%). ^{1}H NMR: (400 MHz, DMSO-d_6) δ 7.46-7.41 (2H, m), 7.36 (1H, t), 7.32-7.22 (5H, m), 5.37 (1H, d), 4.55 (1H, dt), 4.50-4.18 (2H, m) 3.79 (1H, br s), 3.66-3.2 (3H, m), 2.93-2.73 (3H, m), 2.34 (3H, s). ^{13}C NMR: (100 MHz, DMSO-d_6) δ 160.57, 138.10, 134.77, 132.09, 131.82, 130.18, 129.29, 128.73, 128.36, 127.39, 126.78, 124.98, 58.71, 52.38, 51.63, 50.99, 30.05, 27.89, 20.93. HRMS (ESI/Q-TOF) m/z: $[M + H]^+$ Calcd for $C_{20}H_{23}N_2O$ 307.1810; Found 307.1813.

2-(4-Methylbenzyl)-4-oxo-1,3,4,6,7,11*b*-hexahydro-*2H*-pyrazino[2,1-a]isoquinolin-2-ium Chloride (7).

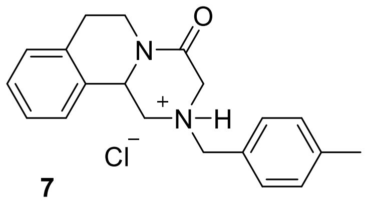

7

7 was synthesized according to the general reductive amination procedure with praziquanamine (0.1734 g, 0.857 mmol), sodium triacetoxyborohydride (0.257 g, 1.2 mmol), and *p*-tolualdehyde (0.15 mL, 1.3 mmol). Addition of HCl in ethyl acetate to the ethyl acetate solution yielded **7** as a white, shiny solid. (0.2082 g, 68%). ^{1}H NMR (400 MHz, DMSO-d_6) δ 7.53 (2H, d), 7.30-7.21 (6H, m), 5.39 (1H, d), 4.55 (1H, dt), 4.51-4.24 (2H, m), 3.87-3.71 (1H, br d), 3.61-3.43 (3H, m), 2.92-2.73 (3H, m), 2.34 (3H, s), ^{13}C NMR (100 MHz, DMSO-d_6) δ 160.49, 139.21, 134.77, 131.40, 129.42, 129.31, 127.41, 126.80, 125.00, 58.27, 52.04, 51.51, 50.77, 38.04, 27.90, 20.77. HRMS (ESI/Q-TOF) m/z: $[M + H]^+$ Calcd for $C_{20}H_{23}N_2O$ 307.1810; Found 307.1815.

2-(2,6-Dimethylbenzyl)-4-oxo-1,3,4,6,7,11*b*-hexahydro-*2H*-pyrazino[2,1-a]isoquinolin-2-ium Chloride (8).

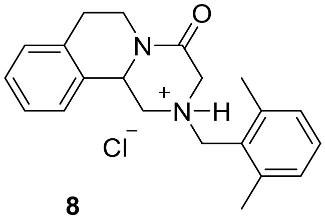

8

8 was synthesized according to the general reductive amination procedure with praziquanamine (0.1734 g, 0.857 mmol), sodium triacetoxyborohydride (0.257 g, 1.2 mmol), and 2,6-dimethylbenzaldehyde (0.17 mL, 1.3 mmol). Addition of HCl in ethyl acetate to the ethyl acetate solution yielded **8** as a white solid (0.1195 g, 39%). ^{1}H NMR (400 MHz, $CDCl_3$) δ 13.28 (1H, br s), 7.30-7.24 (3H, m), 7.22-7.13 (4H, m), 5.86 (1H, s), 4.76 (1H, d), 4.62 (1H, d), 4.50 (1H, d), 4.23 (1H, d), 3.98 (1H, br d), 3.56 (1H, br s), 3.05-2.75 (4H, m), 2.57 (6H, s), ^{13}C NMR (100 MHz, $CDCl_3$) δ 160.09, 139.55, 134.66, 130.87, 130.66, 129.64, 127.96, 127.24, 125.04, 124.70, 77.20, 54.66, 51.77, 51.17, 38.75, 28.44, 21.34. HRMS (ESI/Q-TOF) m/z: $[M + H]^+$ Calcd for $C_{21}H_{25}N_2O$ 321.1967; Found 321.1971.

2-(2-Ethylbenzyl)-4-oxo-1,3,4,6,7,11*b*-hexahydro-*2H*-pyrazino[2,1-a]isoquinolin-2-ium Chloride (9).

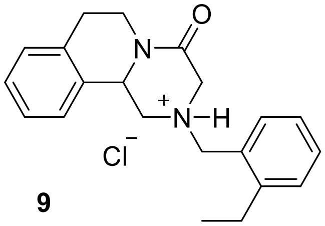

9

9 was synthesized according to the general reductive amination procedure with praziquanamine (0.1734 g, 0.857 mmol), sodium triacetoxyborohydride (0.257 g, 1.2 mmol), and 2-ethylbenzaldehyde (0.17 mL, 1.3 mmol). Addition of HCl in ethyl acetate to the ethyl acetate solution yielded **9** as a white solid (0.2217 g, 73%). ^{1}H NMR (400 MHz, DMSO-d_6) δ 7.67 (1H, br s), 7.44-7.22 (7H, m), 5.35 (1H, br s), 4.59-4.12 (3H, m), 3.90-3.48 (4H, m), 2.95-2.75 (3H, m), 1.16 (3H, t). ^{13}C NMR (100 MHz, DMSO-d_6) δ 160.65, 144.30, 134.68, 132.32, 131.85, 129.75, 129.31, 129.15, 127.37, 126.76, 126.14, 124.89, 55.94, 52.66, 51.77, 51.58, 38.07, 27.94, 25.05, 15.52. HRMS (ESI/Q-TOF) m/z: $[M + H]^+$ Calcd for $C_{21}H_{25}N_2O$ 321.1967; Found 321.1969.

2-(4-Ethylbenzyl)-4-oxo-1,3,4,6,7,11*b*-hexahydro-*2H*-pyrazino[2,1-a]isoquinolin-2-ium Chloride (10).

10

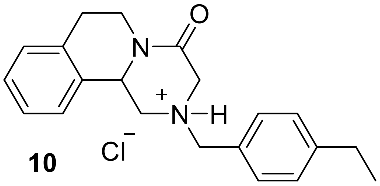

10 was synthesized according to the general reductive amination procedure with praziquanamine (0.1734 g, 0.857 mmol), sodium triacetoxyborohydride (0.257 g, 1.2 mmol), and 4-

ethylbenzaldehyde (0.18 mL, 1.3 mmol) to yield an orange oil upon addition of HCl in ethyl acetate. The oil was extracted with HCl (2M, 10 mL, 3X). Addition of brine to the organic layer formed a white precipitate. The solution was vacuum filtered and the solid was recrystallized in ethyl acetate to yield **10** as a light brown solid (80 mg, 25%). ^{1}H NMR (400 MHz, $CDCl_3$) δ 7.56 (2H, d), 7.33-7.21 (6H, m), 5.42 (1H, br d), 4.55 (1H, dt), 4.52-4.24 (3H, m), 3.82 (1H, br d), 3.54 (1H, br d), 3.20 (1H, br s), 2.92-2.73 (3H, m), 2.63 (2H, q), 1.19 (3H, t). ^{13}C NMR (100 MHz, DMSO-*d*$_6$) δ 160.49, 145.34, 134.75, 132.02, 131.51, 129.29, 128.21, 127.39, 126.79, 124.99, 58.21, 52.07, 51.53, 50.64, 38.03, 27.91, 15.35. HRMS (ESI/Q-TOF) m/z: [M + H]$^+$ Calcd for $C_{21}H_{25}N_2O$ 321.1967; Found 321.1966.

2-(4-Isopropylbenzyl)-4-oxo-1,3,4,6,7,11*b*-hexahydro-*2H*-pyrazino[2,1-a]isoquinolin-2-ium Chloride (11).

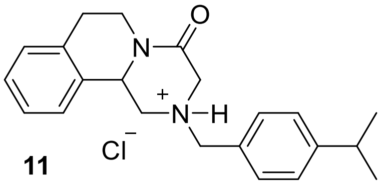

11

11 was synthesized according to the general reductive amination procedure with praziquanamine (0.1734 g, 0.857 mmol), sodium triacetoxyborohydride (0.257 g, 1.2 mmol), and 4-isopropylbenzaldehyde (0.19 mL, 1.3 mmol). Addition of HCl in ethyl acetate to the ethyl acetate solution yielded **11** as a white solid (0.2853 g, 64%). ^{1}H NMR (400 MHz, $CDCl_3$) δ 7.52 (2H, d), 7.33-7.24 (5H, m), 7.17 (1H, br d), 5.84 (1H, br d), 4.74 (1H, d), 4.48 (1H, d), 4.27 (2H, d), 3.94 (1H, d), 3.54 (1H, d), 3.00-2.73 (5H, m), 1.25 (6H, d). ^{13}C NMR (100 MHz, $CDCl_3$) δ 159.95, 151.73, 134.63, 131.62, 130.68, 129.61, 127.98, 127.70, 127.26, 125.33, 123.76, 60.27, 53.83, 51.78, 50.37, 38.78, 33.92, 28.42, 23.72. HRMS (ESI/Q-TOF) m/z: [M + H]$^+$ Calcd for $C_{22}H_{27}N_2O$ 335.2123; Found 335.2126.

2-(4-(*tert*-Butyl)benzyl)-4-oxo-1,3,4,6,7,11*b*-hexahydro-*2H*-pyrazino[2,1-a]isoquinolin-2-ium Chloride (12).

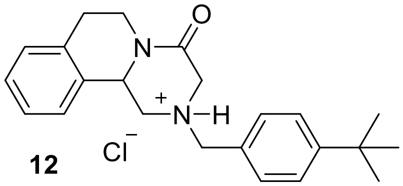

12

12 was synthesized according to the general reductive amination procedure with praziquanamine (0.1734 g, 0.857 mmol), sodium triacetoxyborohydride (0.257 g, 1.2 mmol), and 4-*t*-butylbenzaldehyde (0.20 mL, 1.2 mmol). Addition of HCl in ethyl acetate to the dried ethyl acetate solution yielded **12** as a crystalline white solid (0.2853 g, 86%). ^{1}H NMR (400 MHz, DMSO-*d*$_6$) δ 7.58-7.46 (4H, m), 7.32-7.21 (4H, m), 5.37 (1H, d), 4.55 (1H, dt), 4.52-4.18 (3H, br m), 3.77 (1H, br s), 3.54 (1H, br d), 3.19 (1H, br s), 2.94-2.75 (3H, m), 1.29 (9H, s). ^{13}C NMR (100 MHz, DMSO-*d*$_6$) δ 160.52, 152.15, 134.76, 132.04, 131.17, 129.28, 127.28, 126.76, 125.63, 124.97, 58.25, 52.29, 51.60, 50.84, 38.02, 34.47, 30.99, 27.87. HRMS (ESI/Q-TOF) m/z: [M + H]$^+$ Calcd for $C_{23}H_{29}N_2O$ 349.2280; Found 349.2283.

2-(2-Hydroxybenzyl)-4-oxo-1,3,4,6,7,11*b*-hexahydro-*2H*-pyrazino[2,1-a]isoquinolin-2-ium Chloride (13).

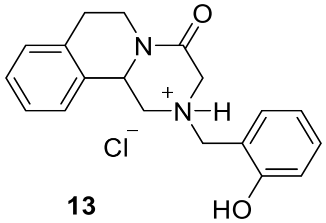

13

13 was synthesized according to the general reductive amination procedure with praziquanamine (0.1734 g, 0.857 mmol), sodium triacetoxyborohydride (0.257 g, 1.2 mmol), and 2-hydroxybenzaldehyde (0.14 mL, 1.3 mmol), except that saturated sodium bicarbonate was used in the extraction instead of aqueous NaOH. Addition of 2 M HCl to the ethyl acetate solution produced **13** as a pale yellow solid. (74.6 mg, 24%). ^{1}H NMR (400 MHz, $CDCl_3$ and DMSO-*d*$_6$) δ 13.36 (1H, br s), 9.77 (1H, br s), 7.52 (1H, d), 7.31-7.16 (5H, m), 6.99 (1H, d), 6.91 (1H, t), 5.73 (1H, br d), 4.74 (1H, d), 4.45 (2H, s), 4.25 (1H, d), 3.96 (1H, d), 3.75 (1H, d), 3.05-2.75 (4H, m). ^{13}C NMR (100 MHz, $CDCl_3$ and DMSO-*d*$_6$) δ 160.08, 156.50, 134.23, 133.24, 131.51,130.67, 129.09, 127.35, 126.62, 124.66, 119.71, 115.96, 113.02, 53.78, 52.84, 51.43, 49.92, 38.22, 27.95. HRMS (ESI/Q-TOF) m/z: $[M + H]^+$ Calcd for $C_{19}H_{21}N_2O_2$ 309.1603; Found 309.1601.

2-(4-Hydroxybenzyl)-4-oxo-1,3,4,6,7,11*b*-hexahydro-*2H*-pyrazino[2,1-a]isoquinolin-2-ium Chloride (14).

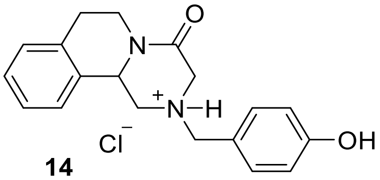

14

14 was synthesized according to the general reductive amination procedure with praziquanamine (0.1734 g, 0.857 mmol), sodium triacetoxyborohydride (0.257 g, 1.2 mmol), and 4-hydroxybenzaldehyde (0.1569 g, 1.285 mmol), except that saturated sodium bicarbonate was used in the extraction instead of aqueous NaOH. 2M aq. HCl was added to the extraction which formed a solid. The solid was isolated by vacuum filtration, yielding a white solid that was recrystallized in ethanol to yield **14** as a white solid (69.9 mg, 23%). ^{1}H NMR (400 MHz, DMSO-*d*$_6$) δ 12.06 (1H, s), 9.88 (1H, s), 7.42 (2H, d), 7.32-7.20 (4H, m), 6.84 (2H, d), 5.35 (1H, d), 4.54 (1H, dt), 4.37 (2H, br m), 3.85 (1H, br d), 3.65 (1H, br d), 3.29 (2H, br s), 2.90-2.73 (3H, m). ^{13}C NMR (100 MHz, DMSO-*d*$_6$) δ 160.45, 158.70, 134.78, 133.02, 131.91, 129.32, 127.44, 126.82, 125.02, 115.60, 58.29, 51.78, 51.55, 50.51, 38.04, 27.89. HRMS (ESI/Q-TOF) m/z: $[M + H]^+$ Calcd for $C_{19}H_{21}N_2O_2$ 309.1603; Found 309.1607.

2-(2-Methoxybenzyl)-4-oxo-1,3,4,6,7,11*b*-hexahydro-*2H*-pyrazino[2,1-a]isoquinolin-2-ium Chloride (15).

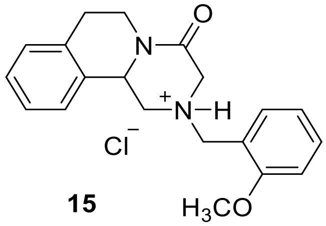

15

15 was synthesized according to the general reductive amination procedure with praziquanamine (0.2046 g, 1.0 mmol), sodium triacetoxyborohydride (0.3035 g, 1.4 mmol), and *o*-anisaldehyde (0.2083 g, 1.4 mmol) which yielded an orange oil. The crude oil was purified by column chromatography on silica gel (hexanes:isopropanol with 1% NH_4OH, 0-20%). The isolated amine was diluted with ethyl acetate (5 mL) and protonated with HCl in ethyl acetate. Addition of hexanes formed **15** as a white solid. (94.4 mg, 26%). ^{1}H NMR (400 MHz, $CDCl_3$) δ 7.71 (1H, d), 7.44 (1H, t), 7.21-7.31 (3H, m), 7.16 (1H, d), 7.04 (1H, t), 6.97 (1H, d), 5.81 (1H, br s), 4.74 (1H, d), 4.53 (2H, s), 4.39 (1H, br d), 3.98 (1H, br d), 3.87 (3H, s), 3.60 (1H, br d), 2.71-2.98 (4H, m). ^{13}C NMR (100 MHz, $CDCl_3$) δ 160.29, 158.17, 134.55, 132.27, 134.12, 130.98, 129.48, 127.78, 127.11, 125.13, 121.48, 115.07, 111.13, 55.60, 53.60, 51.78, 50.13, 38.65, 28.35. HRMS (ESI/Q-TOF) m/z: $[M + H]^+$ Calcd for $C_{20}H_{23}N_2O_2$ 323.1760; Found 323.1763.

2-(3-Methoxybenzyl)-4-oxo-1,3,4,6,7,11*b*-hexahydro-*2H*-pyrazino[2,1-a]isoquinolin-2-ium Chloride (16).

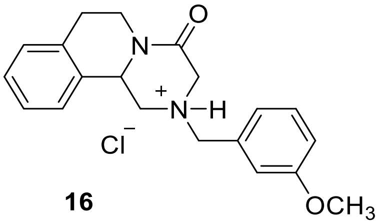

16

16 was synthesized according to the general reductive amination procedure with praziquanamine (0.2046 g, 1.0 mmol), sodium triacetoxyborohydride (0.3035 g, 1.4 mmol), and m-anisaldehyde (0.157 mL, 1.285 mmol). Addition of HCl in ethyl acetate to the ethyl acetate solution yielded **16** as a white solid (0.2429 g, 76%). ^{1}H NMR (400 MHz, DMSO-d_6) δ 7.38 (1H, t), 7.23-7.33 (5H, m), 7.16 (1H, d), 7.03 (1H, d), 5.37 (1H, d), 4.55 (1H, dd), 4.20-4.50 (2H, m), 3.79 (3H, s), 3.50 (1H, br m), 2.90 (1H, m), 2.80 (2H, t). ^{13}C NMR (100 MHz, DMSO-d_6) δ 160.61, 159.47, 134.79, 132.03, 129.98, 129.33, 127.43, 126.81, 124.98, 116.63, 115.28, 58.76, 55.25, 52.45, 51.60, 51.13, 30.08, 27.91. HRMS (ESI/Q-TOF) m/z: $[M + H]^+$ Calcd for $C_{20}H_{23}N_2O_2$ 323.1760; Found 323.1764.

2-(4-Methoxybenzyl)-4-oxo-1,3,4,6,7,11*b*-hexahydro-*2H*-pyrazino[2,1-a]isoquinolin-2-ium Chloride (17).

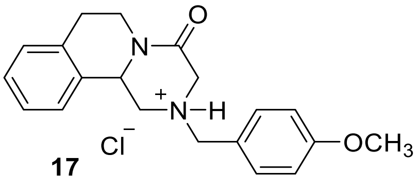

17

17 was synthesized according to the general reductive amination procedure with praziquanamine (0.2032 g, 1.0 mmol), sodium triacetoxyborohydride (0.3035 g, 1.4 mmol), and m-anisaldehyde (0.17 mL, 1.4 mmol). After washing the organic layer with NaOH, the organic layer was extracted with 6 M HCl (3X, 2 mL). The combined acidic aqueous layers were washed with EtOAc (1X, 2 mL) and then the ammonium chloride salt was extracted with chloroform (3X, 2 mL). The combined organic layers were dried over $MgSO_4$, filtered and concentrated under reduced pressure to form a solid that was recrystallized from isopropanol to give **17** as a white powder (0.0450 g, 12%). ^{1}H NMR (400 MHz, DMSO-*d*$_6$) δ 7.57 (2H, d), 7.32-21 (4H, m), 7.02 (2H, t), 5.41 (1H, d), 4.55 (1H, dt), 4.50-4.24 (2H, br m), 3.83 (1H, br m), 3.78 (3H, s), 3.60-3.41 (3H, br m), 3.21-3.11 (1H, br m), 2.90-2.74 (3H, br m). ^{13}C NMR (100 MHz, DMSO-*d*$_6$) δ 160.51, 160.20, 134.77, 133.03, 132.00, 129.32, 127.42, 126.81, 125.03, 114.22, 57.94, 55.23, 51.84, 51.53, 50.52, 38.04, 27.90. HRMS (ESI/Q-TOF) m/z: $[M + H]^+$ Calcd for $C_{20}H_{23}N_2O_2$ 323.1760; Found 323.1766.

4-Oxo-2-(4-phenoxybenzyl)-1,3,4,6,7,11*b*-hexahydro-*2H*-pyrazino[2,1-a]isoquinolin-2-ium Chloride (18).

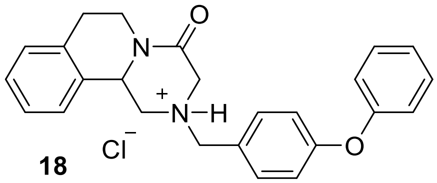

18

18 was synthesized according to the general reductive amination procedure with praziquanamine (0.173 g, 0.857 mmol), 4-(phenoxy)benzaldehyde (0.23 mL, 1.9 mmol) and sodium triacetoxyborohydride (0.257 g, 1.2 mmol). The addition of 2 M HCl in ethyl acetate after the extraction formed a solid, which was vacuum filtered to give **18** as a white, powdery solid (0.156 g, 43%). ^{1}H NMR (400 MHz, DMSO-*d*$_6$) δ 7.65 (2H, d), 7.42 (2H, dd), 7.32-7.22 (4H, m), 7.19 (1H, t), 7.09-7.04 (4H, m), 4.55 (1H, dt), 4.52-4.23 (3H, m), 3.82 (1H, br d), 3.62 (1H, br d), 3.20 (1H, br s), 2.95-2.74 (3H, m). ^{13}C NMR (100 MHz, DMSO-*d*$_6$) δ 160.61, 157.90, 155.93, 134.75, 133.39, 132.06, 130.16, 129.30, 127.41, 126.79, 125.03, 124.00, 119.15, 118.35, 57.81, 52.03, 51.59, 50.75, 38.06, 27.90. HRMS (ESI/Q-TOF) m/z: $[M + H]^+$ Calcd for $C_{225}H_{25}N_2O_2$ 385.1916; Found 385.1916.

2-(Benzo[*d*][1,3]dioxol-5-ylmethyl)-4-oxo-1,3,4,6,7,11*b*-hexahydro-*2H*-pyrazino[2,1-a]isoquinolin-2-ium Chloride (19).

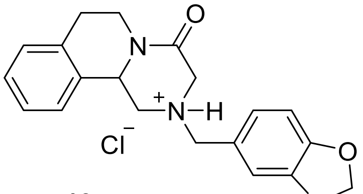

19

19 was synthesized according to the general reductive amination procedure with praziquanamine (0.173 g, 0.857 mmol), 1,3-benzodioxole-5-carboxaldehyde (0.192 g, 1.3 mmol) and sodium triacetoxyborohydride (0.257 g, 1.2 mmol). The addition of aqueous 2 M HCl to the organic layer of the liquid-liquid extraction formed a solid, which was vacuum filtered to give **19** as a white solid (0.111 g, 35%). ^{1}H NMR (400 MHz, DMSO-*d*$_6$) *δ* 7.32-7.21 (5H, m), 7.09 (1H, d), 6.99 (1H, d), 6.08 (2H, d), 5.38 (1H, br d), 4.55 (1H, dt), 4.51-4.19 (3H, m), 3.80 (1H, br d), 3.60

(1H, br d), 3.17 (1H, br s), 2.92-2.73 (3H, m). ^{13}C NMR (100 MHz, DMSO-d_6) δ 160.52, 148.33, 147.51, 134.75, 132.02, 129.28, 127.40, 126.78, 125.65, 125.03, 111.31, 108.43, 101.48, 58.27, 51.89, 51.57, 50.67, 38.04, 27.89. HRMS (ESI/Q-TOF) m/z: $[M + H]^+$ Calcd for $C_{20}H_{21}N_2O_3$ 337.1552; Found 337.1554.

2-(3,4-Dimethoxybenzyl)-4-oxo-1,3,4,6,7,11*b*-hexahydro-*2H*-pyrazino[2,1-a]isoquinolin-2-ium Chloride (20).

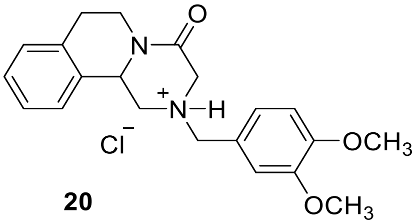

20

20 was synthesized according to the general reductive amination procedure with praziquanamine (0.1734 g, 0.857 mmol), sodium triacetoxyborohydride (0.257 g, 1.2 mmol), and veratraldehyde (0.214 g, 1.29 mmol). Addition of aqueous 2M HCl after to the ethyl acetate layer of the liquid-liquid extraction resulted in formation of a white solid that was filtered to yield **20** as a white solid. (0.199 g, 56%). 1H NMR (400 MHz, $CDCl_3$) δ 7.37 (1H, s), 7.31-7.22 (4H, m), 7.11 (1H, d), 7.02 (1H, d), 5.39 (1H, br d), 4.55 (1H, dt), 4.49-4.21 (3H, m), 3.84 (1H, br s), 3.80 (3H, s), 3.78 (3H, s), 3.56 (1H, d), 3.20 (1H, br s), 2.95-2.86 (3H, m). ^{13}C NMR (100 MHz, DMSO-d_6) δ 160.57, 149.76, 148.69, 134.81, 132.00, 129.36, 127.49, 126.86, 125.04, 124.06, 120.90, 114.71, 111.54, 58.72, 55.61, 55.53, 52.08, 51.54, 50.75, 38.15, 27.94. HRMS (ESI/Q-TOF) m/z: $[M + H]^+$ Calcd for $C_{21}H_{25}N_2O_3$ 353.1865; Found 353.1867.

2-(4-Methoxy-2-methylbenzyl)-4-oxo-1,3,4,6,7,11*b*-hexahydro-*2H*-pyrazino[2,1-a]isoquinolin-2-ium Chloride (21).

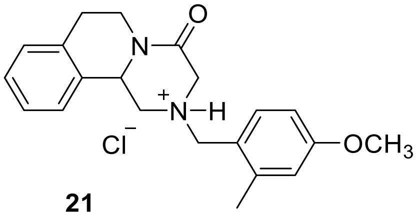

21

21 was synthesized according to the general reductive amination procedure with praziquanamine (0.1734 g, 0.857 mmol), sodium triacetoxyborohydride (0.257 g, 1.2 mmol), and 4-methoxy-2-methyl benzaldehyde (0.17 mL, 1.3 mmol) to yield an orange oil. The ethyl acetate layer was then extracted with aq. 6M HCl (3x, 2 mL). The combined acidic aqueous layers were washed with EtOAc (1X, 2 mL) and then the ammonium chloride salt was extracted with chloroform (3X, 2 mL). The combined organic layers were dried over $MgSO_4$, filtered and concentrated under reduced pressure to form a solid that was recrystallized from acetone to form **21** as a white solid. (76 mg, 24%). ^1H NMR (400 MHz, $CDCl_3$) δ 7.58 (1H, d), 7.32-7.21 (5H, m), 6.87 (1H, s), 6.85 (1H, dd), 5.42 (1H, br d), 4.55 (1H, dt), 4.51-4.23 (2H, br m), 3.93 (1H, br s), 3.77 (3H, s), 3.57 (2H, br d), 3.37 (1H, br s), 2.92-2.75 (3H, m), 2.45 (3H, s). ^{13}C NMR (100 MHz, DMSO-d_6) δ 160.03, 140.55, 134.67, 133.81, 132.16, 129.31, 127.39, 126.79, 124.94, 119.48, 116.11, 111.62, 55.98, 55.11, 52.15, 51.57, 51.14, 27.94, 19.84. HRMS (ESI/Q-TOF) m/z: $[M + H]^+$ Calcd for $C_{21}H_{25}N_2O_2$ 337.1916; Found 337.1916.

2-(4-Hydroxy-3,5-dimethoxybenzyl)-4-oxo-1,3,4,6,7,11*b*-hexahydro-*2H*-pyrazino[2,1-a]isoquinolin-2-ium Chloride (22).

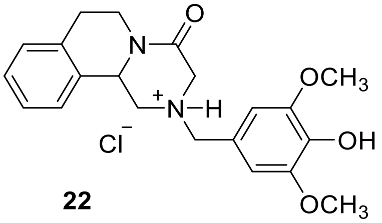

22

22 was synthesized according to the general reductive amination procedure with praziquanamine (0.1734 g, 0.857 mmol), sodium triacetoxyborohydride (0.257 g, 1.2 mmol), and syringaldehyde (0.196 g, 1.3 mmol) except that saturated sodium bicarbonate was used in the extraction instead of aqueous NaOH. Addition of 2 M HCl to the ethyl acetate solution produced a solid which was vacuum filtered, yielding **22** as a white solid (0.180 g, 59%). ^{1}H NMR (400 MHz, DMSO-d_6) δ 11.99 (1H, br s), 8.72 (1H, br s), 7.31-7.22 (4H, m), 6.95 (2H, s), 5.38 (1H, br d), 4.55 (1H, dt), 4.49-4.414 (2H, m), 3.82 (1H, m), 3.78 (6H, s), 3.58 (1H, br d), 3.21 (1H, br s), 2.95-2.75 (3H, m). ^{13}C NMR (100 MHz, DMSO-d_6) δ 160.62, 147.92, 136.65, 134.82, 132.05, 129.37, 127.50, 126.87, 125.02, 108.83, 59.45, 56.15, 52.20, 51.60, 50.96, 38.15, 27.94. HRMS (ESI/Q-TOF) m/z: $[M + H]^+$ Calcd for $C_{21}H_{25}N_2O_4$ 369.1814; Found 369.1819.

2-(2-Hydroxy-3-methoxybenzyl)-4-oxo-1,3,4,6,7,11*b*-hexahydro-*2H*-pyrazino[2,1-a]isoquinolin-2-ium Chloride (23).

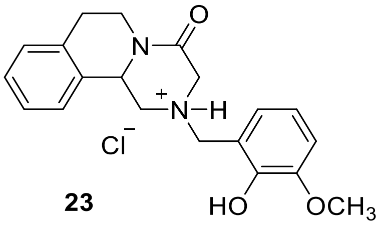

23

23 was synthesized according to the general reductive amination procedure with praziquanamine (0.1734 g, 0.857 mmol), sodium triacetoxyborohydride (0.257 g, 1.2 mmol), and *o*-vanillin (0.234 g, 1.3 mmol) except that saturated sodium bicarbonate was used in the extraction instead of aqueous NaOH. The ethyl acetate solution was extracted with aq. 2M HCl (3X, 2 mL) followed by brine (1X, 2 mL). and then the ammonium chloride salt was extracted with chloroform (3X, 2 mL). The combined organic layers were dried over $MgSO_4$, filtered and concentrated under reduced pressure to yield a solid product. The solid was recrystallized from isopropanol to afford **23** as a white solid (0.120 g, 56%). ^{1}H NMR (400 MHz, DMSO-d_6) δ 12.12 (1H, br s), 9.50 (1H, br s), 7.31-7.20 (4H, m), 7.16 (1H, d), 7.06 (1H, d), 6.86 (1H, t), 5.37 (1H, d), 4.55 (1H, d), 4.46-4.31 (3H, m), 3.86 (1H, br s), 3.82 (3H, s), 3.66 (1H, d), 3.30 (1H, br t), 2.91-2.70 (3H, m). ^{13}C NMR (100 MHz, DMSO-d_6) δ 160.64, 147.84, 146.07, 134.87, 132.08, 129.28, 127.37, 126.76, 125.06, 124.77, 119.25, 115.48, 113.78, 62.01, 55.91, 53.05, 52.03, 51.54, 50.98, 38.02, 27.89, 25.49. HRMS (ESI/Q-TOF) m/z: $[M + H]^+$ Calcd for $C_{20}H_{23}N_2O_3$ 339.1709; Found 339.1716.

2-((6-Methoxybenzo[d][1,3]dioxol-5-yl)methyl)-4-oxo-1,3,4,6,7,11*b*-hexahydro-*2H*-pyrazino[2,1-a]isoquinolin-2-ium Chloride (24).

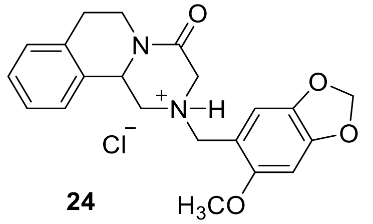

24

24 was synthesized according to the general reductive amination procedure with praziquanamine (0.1734 g, 0.857 mmol), sodium triacetoxyborohydride (0.257 g, 1.2 mmol), and 6-methoxy-1,3-dibenzodioxole-5-carbaldehyde (0.216 g, 1.3 mmol). After washing with the aqueous NaOH, the ethyl acetate solution was extracted with aq. 2M HCl (3X, 2 mL) followed by brine (1X, 2 mL) and then the ammonium chloride salt was extracted with $CHCl_3$ (3X 2 mL). The combined $CHCl_3$ layers were dried with $MgSO_4$, filtered and evaporated under reduced pressure to yield a solid product. The solid was recrystallized from ethyl acetate to afford **24** as a white solid (0.079 g, 26%). ^{1}H NMR (400 MHz, $CDCl_3$) δ 13.79 (1H, br s), 7.32-7.15 (4H, m), 6.56 (1H, s), 5.97 (2H, app d), 5.80 (1H, br s), 4.76 (1H, d), 4.36 (2H, br s), 4.16 (1H, br d), 3.95 (1H, d), 3.80 (3H, s), 3.55 (1H, br d), 3.01-2.74 (4H, m). ^{13}C NMR (100 MHz, $CDCl_3$) δ 160.36, 154.27, 150.66, 141.81, 134.68, 131.03, 129.62, 127.91, 127.20, 125.20, 112.53, 101.94, 94.51, 56.42, 54.16, 53.74, 51.90, 50.01, 38.75, 28.48. HRMS (ESI/Q-TOF) m/z: $[M + H]^+$ Calcd for $C_{21}H_{23}N_2O_4$ 367.1658; Found 367.1658.

2-(3-Ethoxy-4-hydroxybenzyl)-4-oxo-1,3,4,6,7,11*b*-hexahydro-*2H*-pyrazino[2,1-a]isoquinolin-2-ium chloride (25).

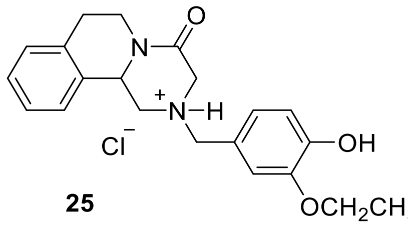

25

25 was synthesized according to the general reductive amination procedure with praziquanamine (0.1734 g, 0.857 mmol), sodium triacetoxyborohydride (0.257 g, 1.2 mmol), and ethyl *o*-vanillin (0.2135 g, 1.3 mmol). The aqueous NaOH layer was neutralized with concentrated HCl to pH 7 and then saturated sodium bicarbonate was added which formed a white precipitate. The precipitate was filtered and then recrystallized from isopropanol to yield **25** as a white solid (0.120 g, 56%). ^{1}H NMR (400 MHz, DMSO-d_6) δ 8.83 (1H, s). 7.23-7.12 (4H, m), 6.87 (1H, s), 6.74 (1H, t), 6.72 (1H, t), 4.83 (1H, dd), 4.52 (1H, dt), 4.00 (3H, q), 3.62-3.55 (2H, m), 3.41 (1H, d), 3.34 (3H, s), 3.21 (1H, d), 2.91-2.72 (3H, m), 2.29 (1H, t), 1.32 (3H, t), ^{13}C NMR (100 MHz, DMSO-d_6) δ 165.39, 146.56, 146.03, 134.95, 134.70, 128.97, 127.64, 126.67, 126.38, 124.75, 121.51, 115.23, 114.28, 63.80, 60.32, 56.25, 55.09, 54.62, 38.03, 28.18, 14.79. HRMS (ESI/Q-TOF) m/z: $[M + H]^+$ Calcd for $C_{21}H_{25}N_2O_3$ 353.1865; Found 353.1867.

2-(2-Fluorobenzyl)-4-oxo-1,3,4,6,7,11*b*-hexahydro-*2H*-pyrazino[2,1-a]isoquinolin-2-ium Chloride (26).

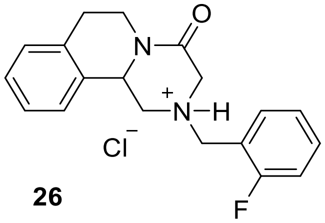

26

26 was synthesized according to the general reductive amination procedure with praziquanamine (0.202 g, 1.0 mmol), sodium triacetoxyborohydride (0.302 g, 1.4 mmol), and 2-fluorobenzaldehyde (0.15 mL, 1.4 mmol) to yield a brown-orange oil. The ethyl acetate layer from the liquid-liquid extraction was extracted with 6 M HCl (3X, 2 mL) and the combined aqueous layer was washed with EtOAc (1X, 2 mL) and then the ammonium chloride salt was extracted with $CHCl_3$ (3X, 2 mL). The combined organic layers were dried with $MgSO_4$, filtered and concentrated under reduced pressure to yield a yellow solid. The solid was recrystallized from isopropanol to yield **26** as a white solid (0.154 g, 38%). 1H NMR (400 MHz, DMSO-d_6) δ 12.43 (1H, br s), 7.79 (1H, t). 7.54 (1H, m), 7.36-7.20 (6H, m), 5.35 (1H, br d), 4.56 (1H, dt), 4.52-4.27 (3H, m), 3.82-3.62 (2H, m), 3.26 (1H, br t), 2.92-2.74 (3H, m). ^{13}C NMR (100 MHz, DMSO-d_6) *δ* 161.21 (d), 160.73, 134.75, 133.99, 132.23, 129.28, 127.35, 126.77, 124.94 (d), 124.89, 116.49, 115.87 (d), 61.99, 52.18, 51.68, 51.34, 38.03, 27.92. HRMS (ESI/Q-TOF) m/z: $[M + H]^+$ Calcd for $C_{19}H_{20}FN_2O$ 311.1560; Found 311.1566.

2-(3-Fluorobenzyl)-4-oxo-1,3,4,6,7,11*b*-hexahydro-*2H*-pyrazino[2,1-a]isoquinolin-2-ium Chloride (27).

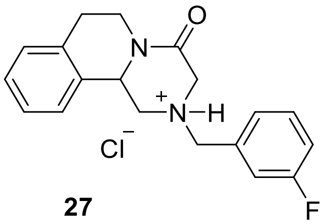

27

27 was synthesized according to the general reductive amination procedure with praziquanamine (0.1734 g, 0.857 mmol), sodium triacetoxyborohydride (0.257 g, 1.2 mmol), and 3-fluorobenzaldehyde (0.14 mL, 1.3 mmol) to yield an orange oil. The ethyl acetate layer from the liquid-liquid extraction was extracted with 2 M HCl (3X, 2 mL). The combined acidic aqueous layers were washed with EtOAc (1X, 2 mL) and then the ammonium chloride salt was extracted with chloroform (3X, 2 mL). The combined organic layers were dried over $MgSO_4$, filtered and concentrated under reduced pressure to yield a pale red solid. The solid was recrystallized from isopropanol to yield **27** as a pale red solid (43 mg, 14%). ^{1}H NMR (400 MHz, $CDCl_3$) δ 13.96 (1H, br s), 7.52 (1H, d), 7.47-7.40 (2H, m), 7.28-7.13 (4H, m), 5.78 (1H, s), 4.74 (1H, d), 4.55 (1H, d), 4.34 (2H, br s), 3.96 (1H, d), 3.62 (1H, d), 3.02-2.72 (4H, m). ^{13}C NMR (100 MHz, $CDCl_3$) δ 162.81, 159.93, 134.61, 131.37, 131.29, 130.61, 129.61, 128.01, 127.29, 125.26, 118.50, 117.70, 59.72, 54.12, 51.85, 51.05, 38.80, 28.43. HRMS (ESI/Q-TOF) m/z: $[M + H]^+$ Calcd for $C_{19}H_{20}FN_2O$ 311.1560; Found 311.1565.

2-(4-Fluorobenzyl)-4-oxo-1,3,4,6,7,11*b*-hexahydro-*2H*-pyrazino[2,1-a]isoquinolin-2-ium Chloride (28).

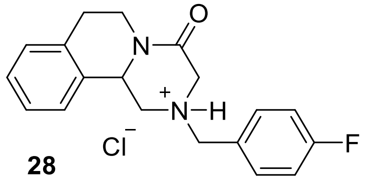

28

28 was synthesized according to the general reductive amination procedure with with praziquanamine (0.1734 g, 0.857 mmol), sodium triacetoxyborohydride (0.257 g, 1.2 mmol), 4-fluorobenzaldehyde (0.14 mL, 1.3 mmol) to yield an orange oil. The ethyl acetate layer from the liquid-liquid extraction was extracted with 2 M HCl (3X). The combined acidic aqueous layers were washed with EtOAc (1X, 2 mL) and then chloroform was added (2 mL). A precipitate formed when chloroform was added to the combined aqueous layers. The solution was vacuum filtered to yield **28** as a white solid (89.5 mg, 29%). ^{1}H NMR (400 MHz, DMSO-d_6) δ 7.71 (2H, dd), 7.35-7.21 (6H, m), 5.38 (1H, d), 4.55 (1H, dd), 4.53-4.29 (4H, m), 3.82 (1H, d), 3.58 (1H, d), 3.20 (1H, br t), 2.92-2.74 (3H, m), ^{13}C NMR (100 MHz, DMSO-d_6) *δ* 162.38 (d), 160.58, 134.78, 133.83 (d), 132.04, 129.32, 127.43, 126.81, 125.05, 115.79 (d), 57.46, 51.99, 51.54, 50.82, 38.08, 27.92. HRMS (ESI/Q-TOF) m/z: $[M + H]^+$ Calcd for $C_{19}H_{20}FN_2O$ 311.1560; Found 311.1565.

2-(2-Bromobenzyl)-4-oxo-1,3,4,6,7,11*b*-hexahydro-*2H*-pyrazino[2,1-a]isoquinolin-2-ium Chloride (29).

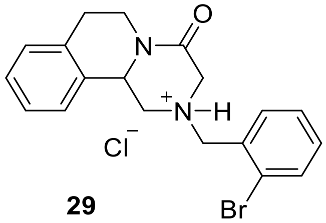

29

29 was synthesized according to the general reductive amination procedure with praziquanamine (0.173 g, 0.857 mmol), 2-bromobenzaldehyde (0.222 g, 1.2 mmol) and sodium triacetoxyborohydride (0.257 g, 1.2 mmol). The ethyl acetate layer from the liquid-liquid extraction was dried with $MgSO_4$ and filtered. Addition of 2 M HCl in ethyl acetate after the extraction formed **29** as a white, granular solid (0.2414 g, 71%). ^{1}H NMR (400 MHz, DMSO-d_6) *δ* 7.92 (1H, d), 7.75 (1H, d), 7.51 (1H, t), 7.40 (1H, t), 7.31-7.21 (4H, m), 5.30 (1H, br d), 4.55 (1H, dt), 4.55-4.18 (3H, m), 3.78 (1H, m), 3.65 (1H, d), 3.30 (1H, br s), 2.95-2.74 (3H, m). ^{13}C NMR (100 MHz, DMSO-d_6) *δ* 161.17, 134.77, 133.45, 133.23, 132.48, 131.40, 129.23, 128.23, 127.30, 126.71, 125.16, 124.96, 58.13, 52.71, 52.29, 52.01, 38.11, 27.93. HRMS (ESI/Q-TOF) m/z: $[M + H]^+$ Calcd for $C_{19}H_{20}BrN_2O$ 371.0759; Found 371.0757.

2-(3-Bromobenzyl)-4-oxo-1,3,4,6,7,11*b*-hexahydro-*2H*-pyrazino[2,1-a]isoquinolin-2-ium Chloride (30).

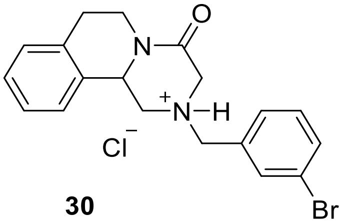

30

30 was synthesized according to the general reductive amination procedure with praziquanamine (0.173 g, 0.857 mmol), 3-bromobenzaldehyde (0.222 g, 1.2 mmol) and sodium triacetoxyborohydride (0.257 g, 1.2 mmol). The ethyl acetate layer from the liquid-liquid extraction was dried with $MgSO_4$ and filtered. Addition of 2 M HCl in ethyl acetate after the extraction formed **30** as a white, powdery solid (0.1527 g, 45%). ^{1}H NMR (400 MHz, $CDCl_3$) δ 7.92 (1H, d), 7.69-7.64 (2H, m), 7.43 (1H, t), 7.31-7.22 (4H, m), 5.30 (1H, br d), 4.55 (1H, dt), 4.55-4.29 (3H, m), 3.82 (1H, m), 3.59 (1H, d), 3.21 (1H, br s), 2.95-2.74 (3H, m). ^{13}C NMR (100 MHz, DMSO-*d*$_6$) δ 160.63, 134.78, 132.97, 132.44, 132.09, 130.92, 130.45, 129.29, 127.40, 126.78, 125.03, 121.93, 57.56, 52.25, 51.59, 51.16, 38.08, 27.90. HRMS (ESI/Q-TOF) m/z: [M + H]$^+$ Calcd for $C_{19}H_{20}BrN_2O$ 371.0759; Found 371.0757.

2-(4-Bromobenzyl)-4-oxo-1,3,4,6,7,11*b*-hexahydro-*2H*-pyrazino[2,1-a]isoquinolin-2-ium Chloride (31).

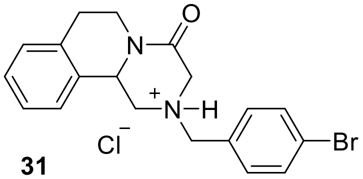

31

31 was synthesized according to the general reductive amination procedure with praziquanamine (0.173 g, 0.857 mmol), 4-bromobenzaldehyde (0.222 mL, 1.2 mmol) and sodium triacetoxyborohydride (0.257 g, 1.2 mmol). T The ethyl acetate layer from the liquid-liquid extraction was dried with Mg_2SO_4 and filtered. Addition of 2 M HCl in ethyl acetate after the extraction formed **31** as a white, granular solid (0.2993 g, 88%). ^{1}H NMR (400 MHz, DMSO-*d*$_6$) δ 7.69-7.62 (4H, m), 7.29-7.20 (4H, m), 5.41 (1H, dd), 4.29-4.34 (4H, m), 3.87 (1H, d), 3.62 (1H, d), 3.24 (1H, t), 2.92-2.72 (3H, m). ^{13}C NMR (100 MHz, DMSO-*d*$_6$) δ 160.48, 134.75, 133.72, 131.96, 131.80, 129.29, 128.32, 127.42, 126.80, 125.06, 123.35, 57.40, 51.95, 51.42, 50.78, 38.09, 27.91. HRMS (ESI/Q-TOF) m/z: [M + H]$^+$ Calcd for $C_{19}H_{20}BrN_2O$ 371.0759; Found 371.0758.

2-(2-Nitrobenzyl)-4-oxo-1,3,4,6,7,11*b*-hexahydro-*2H*-pyrazino[2,1-a]isoquinolin-2-ium Chloride (32).

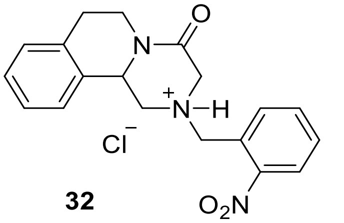

32

32 was synthesized according to the general reductive amination procedure with praziquanamine (0.202 g, 1.0 mmol), sodium triacetoxyborohydride (0.302 g, 1.4 mmol), and 2-nitrobenzaldehyde (0.218 g, 1.4 mmol) to yield a brown-orange oil. After washing the organic layer with NaOH, the organic layer was extracted with 6 M HCl (3X, 2 mL). The combined acidic aqueous layers were washed with EtOAc (1X, 2 mL). The aqueous layer was extracted with $CHCl_3$ (3X) and a solid formed in the 6M HCl layer. This solid was filtered and recrystallized from isopropanol to yield **32** as a white solid (0.2011 g, 58%). ^{1}H NMR (400 MHz, DMSO-*d6*) δ 7.95 (1H, d) 7.76-7.71 (2H, m), 7.58 (1H, m), 7.22-7.16 (2H, m), 7.11 (1H, m), 4.78 (1H, dd), 4.50 (1H, dt), 3.97 (1H, d), 3.89 (1H, d), 3.49 (1H, dd), 3.19 (1H, d), 2.97 (1H, d), 2.89-2.85 (1H, m), 2.78-2.72 (2H, m), 2.43 (1H, t). ^{13}C NMR (100 MHz, DMSO-*d6*) δ 164.90, 149.28, 134.71, 134.51, 133.12, 131.96, 131.09, 128.95, 128.80, 126.74, 126.38, 124.72, 124.45, 56.63, 56.46, 55.24, 54.45, 38.07, 28.12. HRMS (ESI/Q-TOF) m/z: $[M + H]^+$ Calcd for $C_{19}H_{20}N_3O_3$ 338.1505; Found 338.1505.

2-(3-Nitrobenzyl)-4-oxo-1,3,4,6,7,11*b*-hexahydro-*2H*-pyrazino[2,1-a]isoquinolin-2-ium Chloride (33).

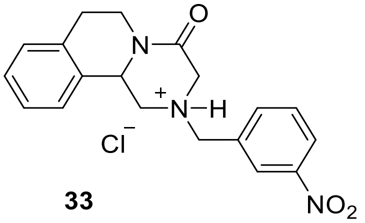

33

33 was synthesized according to the general reductive amination procedure with praziquanamine (0.1734 g, 0.857 mmol), sodium triacetoxyborohydride (0.257 g, 1.2 mmol), and 3-nitrobenzaldehyde (0.1943 g, 1.3 mmol). Addition of HCl in ethyl acetate to the ethyl acetate solution yielded a solid. The solid was recrystallized in ethanol to produce **33** as a white solid (0.105 g, 33%). ^{1}H NMR (400 MHz, DMSO-*d6*) δ 8.55 (1H, s), 8.31 (1H, d), 8.11 (1H, d) 7.77 (1H, t), 7.31-7.21 (4H, m), 5.34 (1H, d), 4.65-4.42 (3H, m) 4.34 (1H, br s), 3.81 (1H, d), 3.65 (1H, d), 3.18 (1H, br t), 2.95-2.84 (1H, m), 2.81-2.25 (2H, m). ^{13}C NMR (100 MHz, DMSO-*d6*) δ 160.83, 147.90, 137.85, 134.74, 132.21, 130.28, 129.26, 127.37, 126.73, 126.12, 125.06, 124.27, 57.36, 52.33, 51.74, 51.49, 38.08, 27.92. HRMS (ESI/Q-TOF) m/z: $[M + H]^+$ Calcd for $C_{19}H_{20}N_3O_3$ 338.1505; Found 338.1505.

2-(4-Nitrobenzyl)-4-oxo-1,3,4,6,7,11*b*-hexahydro-*2H*-pyrazino[2,1-a]isoquinolin-2-ium Chloride (34).

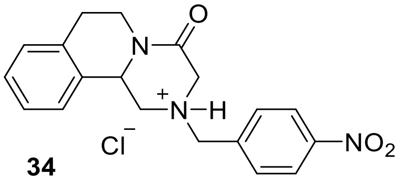

34

34 was synthesized according to the general reductive amination procedure with praziquanamine (0.1734 g, 0.857 mmol), sodium triacetoxyborohydride (0.257 g, 1.2 mmol), and 4-nitrobenzaldehyde (0.1943 g, 1.3 mmol). Addition of HCl in ethyl acetate to the ethyl acetate solution yielded a solid. The solid was recrystallized in isopropanol to produce **34** as a light brown solid (0.1640 g, 51%). ^{1}H NMR (400 MHz, DMSO-*d6*) δ 8.31 (2H, d), 7.88 (2H, d), 7.28-7.21 (4H, m), 5.25 (1H, d), 4.55 (1H, dt), 4.52-4.13 (4H, m), 3.61 (1H, br s), 3.07 (1H, br s),

2.95-2.85 (1H, m), 2.82-2.25 (2H, m). ^{13}C NMR (100 MHz, DMSO-*d$_6$*) *δ* 160.83, 147.90, 137.85, 134.74, 132.21, 130.28, 129.26, 127.37, 126.73, 126.12, 125.06, 124.27, 57.36, 52.33, 51.74, 51.51, 38.08, 27.92. HRMS (ESI/Q-TOF) m/z: [M + H]$^+$ Calcd for $C_{19}H_{20}N_3O_3$ 338.1505; Found 338.1505.

2-(Naphthalen-2-ylmethyl)-1,2,3,6,7,11*b*-hexahydro-*4H*-pyrazino[2,1-a]isoquinolin-4-one (35).

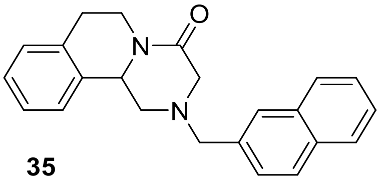

35

35 was synthesized according to the general reductive amination procedure with praziquanamine (0.1734 g, 0.857 mmol), sodium triacetoxyborohydride (0.257 g, 1.2 mmol), and 2-naphthaldehyde (0.187 g, 1.2 mmol). A solid formed in the liquid-liquid extraction with NaOH and ethyl acetate and was isolated by vacuum filtration. The solid was recrystallized in toluene to produce **35** as a white solid (0.0927 g, 31%). ^{1}H NMR (400 MHz, DMSO-*d$_6$*) *δ* 8.18 (1H, br s), 8.01 (1H, d)m 7.99-7.92 (2H, m)m 7.84 (1H, d), 7.62-7.56 (2H, m), 7.28-7.19 (4H, m), 5.46 (1H, d), 4.74-4.39 (4H, m), 3.92 (1H, br s), 3.59 (1H, d), 3.29 (1H, br s), 2.92-2.74 (3H, m). ^{13}C NMR (100 MHz, DMSO-*d$_6$*) *δ* 160.62, 134.74, 133.10, 132.51, 132.13, 131.22, 129.26, 128.39, 128.29, 128.08, 127.69, 127.34, 127.04, 126.76, 126.67, 125.05, 58.46, 52.12, 51.61, 50.93, 38.05, 27.91. HRMS (ESI/Q-TOF) m/z: [M + H]$^+$ Calcd for $C_{23}H_{23}N_2O$ 343.1810; Found 343.1812.

2-(Naphthalen-1-ylmethyl)-4-oxo-1,3,4,6,7,11*b*-hexahydro-*2H*-pyrazino[2,1-a]isoquinolin-2-ium Chloride (36).

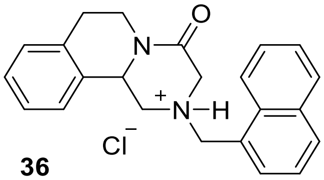

36

36 was synthesized according to the general reductive amination procedure with praziquanamine (0.1734 g, 0.857 mmol), sodium triacetoxyborohydride (0.257 g, 1.2 mmol), and 1-naphthaldehyde (0.16 mL, 1.3 mmol). The ethyl acetate layer from the liquid-liquid extraction was extracted with 2 M HCl (3X, 2 mL). The ammonium chloride salt was extracted from the combined aqueous layer with $CHCl_3$ (3X, 2 mL). The combined organic layers were dried with $MgSO_4$, filtered and concentrated under reduced pressure to yield a solid. The solid was recrystallized from ethyl acetate to yield **36** as a pale red solid (0.0929 mg, 31%). ^{1}H NMR (400 MHz, DMSO-*d$_6$*) *δ* 12.12 (1H, br s), 8.47 (1H, d), 8.07 (1H, d), 8.03 (1H, d), 7.92 (1H, br s), 7.78-7.57 (3H, m), 7.29-7.21 (4H, m), 5.36 (1H, br s), 5.05-4.76 (2H, m), 4.53 (1H, dt), 4.47 (1H, br s), 3.92 (1H, br s), 3.58 (1H, br d), 3.39 (1H, br s), 2.92-2.74 (3H, m). ^{13}C NMR (100 MHz, DMSO-*d$_6$,* δ) 160.74, 134.68, 133.45, 132.28, 132.05, 131.26, 130.34, 129.26, 128.70, 127.31, 126.90, 126.71, 126.25, 125.35, 124.90, 124.14, 55.52, 52.72, 51.82, 37.98, 27.91. HRMS (ESI/Q-TOF) m/z: [M + H]$^+$ Calcd for $C_{23}H_{23}N_2O$ 343.1810; Found 343.1816.

4-Oxo-2-(3-phenylpropyl)-1,3,4,6,7,11*b*-hexahydro-*2H*-pyrazino[2,1-a]isoquinolin-2-ium Chloride (37).

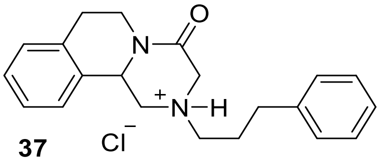

37 was synthesized according to the general reductive amination procedure with praziquanamine (0.1734 g, 0.857 mmol), sodium triacetoxyborohydride (0.257 g, 1.2 mmol), and 3-phenylpropanal (0.14 mL, 1.3 mmol) to yield an orange oil. The ethyl acetate layer from the liquid-liquid extraction was extracted with 2 M HCl (3X, 2 mL). The ammonium chloride salt was extracted from the combined aqueous layer with $CHCl_3$ (3X, 2 mL). The combined organic layers were dried with $MgSO_4$, filtered and concentrated under reduced pressure to yield a pale red solid. The solid was recrystallized from ethyl acetate to yield **37** as a white solid (0.1142 g, 37%). ^{1}H NMR (400 MHz, DMSO-*d$_6$*) δ: 12.27 (1H, br s), 7.41 (1H, dd), 7.35-7.19 (8H, m), 5.35 (1H, d), 4.56 (1H, dt), 4.39 (1H, s), 3.98 (1H, br d), 3.84 (1H, br d), 3.18 (3H, br s), 2.91 (1H, ddd), 2.80 (2H, br s), 2.66 (2H, t), 2.24-2.08 (2H, m) ^{13}C NMR (100 MHz, DMSO-*d$_6$*) δ 160.61, 140.50, 134.64, 132.03, 129.22, 128.39, 128.32, 127.39, 126.68, 126.10, 125.30, 55.49, 52.24, 51.81, 51.59, 38.07, 32.05, 27.92, 24.44. HRMS (ESI/Q-TOF) m/z: $[M + H]^+$ Calcd for $C_{21}H_{25}N_2O$ 321.1967; Found 321.1968.

4-Oxo-2-phenethyl-1,3,4,6,7,11*b*-hexahydro-*2H*-pyrazino[2,1-a]isoquinolin-2-ium Chloride (38).

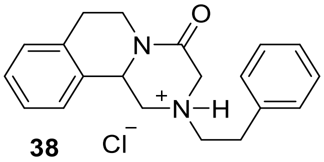

38 was synthesized according to the general reductive amination procedure with praziquanamine (0.1734 g, 0.857 mmol), sodium triacetoxyborohydride (0.257 g, 1.2 mmol), and 3-phenylpropanal (0.14 mL, 1.3 mmol) to yield an orange oil. The ethyl acetate layer from the liquid-liquid extraction was washed with 2 M HCl (3X), and a solid formed in the combined aqueous layers. The solid was filtered and recrystallized from ethyl acetate to yield **38** as a white solid (0.2129 g, 72%). ^{1}H NMR (400 MHz, DMSO-*d$_6$*) δ: 7.42-7.22 (9H, m), 5.31 (1H, br s), 4.58 (1H, dt), 4.38 (1H, br s), 3.99 (1H, br d), 3.83 (1H, br s), 3.50 (1H, br s), 3.25-3.07 (3H, m), 2.91 (1H, ddd), 2.82-2.76 (1H, m). ^{13}C NMR (100 MHz, DMSO-*d$_6$*) δ 161.09, 137.36, 134.71, 132.3, 129.26, 128.74, 128.62, 127.37, 126.70, 125.26, 56.56, 52.63, 51.88, 51.76, 38.10, 29.54, 27.98. HRMS (ESI/Q-TOF) m/z: $[M + H]^+$ Calcd for $C_{20}H_{23}N_2O$ 307.1810; Found 307.1814.

2-([1,1'-Biphenyl]-4-ylmethyl)-4-oxo-1,3,4,6,7,11*b*-hexahydro-*2H*-pyrazino[2,1-a]isoquinolin-2-ium Chloride (39).

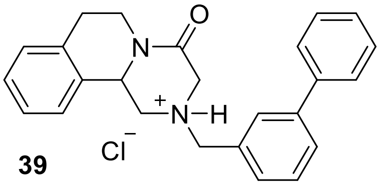

39 was synthesized according to the general reductive amination procedure with praziquanamine (0.1734 g, 0.857 mmol), sodium triacetoxyborohydride (0.257 g, 1.2 mmol), and biphenyl-3-carbaldehyde (0.1953 g, 1.3 mmol). Addition of HCl in ethyl acetate to the ethyl acetate solution yielded **39** as a white solid (0.2490 g, 71%). ^{1}H NMR (400 MHz, DMSO-d_6) δ: 8.09 (1H, s), 7.79-7.73 (3H, m), 7.65 (1H, d), 7.56 (1H, t), 7.48 (2H, t), 7.39 (1H, t), 7.31-7.21 (4H, m), 5.47 (1H, br d), 4.70-4.40 (4H, m), 3.96 (1H, d), 3.61 (1H, d), 3.34 (1H, br t), 2.94-2.74 (3H, m). ^{13}C NMR (100 MHz, DMSO-d_6) δ 160.52, 140.61, 139.43, 134.75, 131.99, 130.34, 129.95, 129.42, 129.31, 128.97, 127.81, 127.43, 126.85, 126.85, 125.00, 58.61, 52.32, 51.49, 50.91, 38.11, 27.91. HRMS (ESI/Q-TOF) m/z: $[M + H]^+$ Calcd for $C_{25}H_{25}N_2O$ 369.1967; Found 369.1962.

2-([1,1'-Biphenyl]-3-ylmethyl)-4-oxo-1,3,4,6,7,11*b*-hexahydro-*2H*-pyrazino[2,1-a]isoquinolin-2-ium Chloride (40).

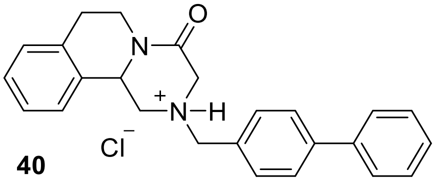

40

40 was synthesized according to the general reductive amination procedure with praziquanamine (0.1734 g, 0.857 mmol), sodium triacetoxyborohydride (0.257 g, 1.2 mmol), and biphenyl-4-carbaldehyde (0.1953 g, 1.3 mmol). Addition of HCl in ethyl acetate to the ethyl acetate solution yielded **40** as a white solid (0.2617 g, 75%). ^{1}H NMR (400 MHz, DMSO-d_6) δ: 7.77 (4H, s), 7.71 (2H, d), 7.48 (2H, t), 7.39 (2H, t), 7.33-7.21 (4H, m), 5.43 (1H, d) 4.64-4.36 (4H, m), 3.89 (1H, d), 3.65 (1H, d), 3.26 (br t), 2.95-2.74 (3H, m). ^{13}C NMR (100 MHz, DMSO-d_6) δ 160.54, 141.24, 139.25, 134.77, 132.13, 132.02, 129.29, 129.00, 127.88, 127.40, 127.02, 126.78, 125.04, 57.99, 52.09, 51.59, 50.82, 38.06, 27.90. HRMS (ESI/Q-TOF) m/z: $[M + H]^+$ Calcd for $C_{25}H_{25}N_2O$ 369.1967; Found 369.1967.

4-Oxo-2-(pyridin-2-ylmethyl)-1,3,4,6,7,11*b*-hexahydro-*2H*-pyrazino[2,1-a]isoquinolin-2-ium Chloride (41).

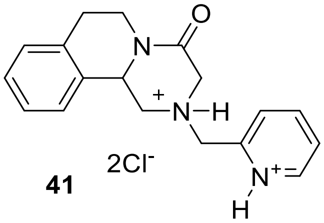

41

41 was synthesized according to the general reductive amination procedure with praziquanamine (0.1734 g, 0.857 mmol), sodium triacetoxyborohydride (0.257 g, 1.2 mmol), and 2-pyridinecarbaldehyde (0.101 mL, 1.3 mmol). Addition of HCl in ethyl acetate to the ethyl acetate solution yielded a solid. The solid was isolated by vacuum filtration. It was then in isopropanol and isolated by vacuum filtration to yield **41** as a white solid (0.1530 g, 51%). ^{1}H NMR (400 MHz, DMSO-d_6) δ 8.76 (1H, d), 8.20 (1H, td), 7.89 (1H, d), 7.70 (1H, dd), 7.28-7.21 (4H, m), 5.48 (2H, br s), 5.25 (1H, dd), 4.55 (1H, dt), 4.51 (1H, d), 4.47 (1H, d), 3.75 (2H, s), 3.12 (1H, t), 2.88 (1H, m), 2.82-2.75 (2H, m). ^{13}C NMR (100 MHz, DMSO-d_6) δ: 161.80, 150.20, 146.67, 140.98, 134.78, 132.72, 129.25, 127.28, 126.67, 126.41, 125.10, 57.58, 53.13, 53.04, 52.36, 38.09, 28.00. HRMS (ESI/Q-TOF) m/z: $[M + H]^+$ Calcd for $C_{18}H_{20}N_3O$ 294.1601; Found 294.1609.

4-Oxo-2-(pyridin-3-ylmethyl)-1,3,4,6,7,11*b*-hexahydro-*2H*-pyrazino[2,1-a]isoquinolin-2-ium Chloride (42).

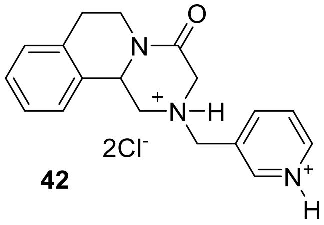

42

42 was synthesized according to the general reductive amination procedure with praziquanamine (0.1734 g, 0.857 mmol), sodium triacetoxyborohydride (0.257 g, 1.2 mmol), and 3-pyridinecarbaldehyde (0.11 mL, 1.2 mmol). Addition of HCl in ethyl acetate to the ethyl acetate solution yielded a solid. The solid was recrystallized from isopropanol to yield **42** as a white solid (0.0631 g, 18%). ^{1}H NMR (400 MHz, DMSO-*d*$_6$) δ 9.13 (1H, s), 8.95 (1H, d), 8.75 (1H, d), 8.06 (1H, dd), 7.34-7.21 (4H, m), 5.28 (1H, d), 5.10 (2H, br s), 4.58-4.47 (3H, m), 4.29 (1H, d), 3.76 (2H, s), 3.12 (1H, t), 2.96-2.74 (3H, m). ^{13}C NMR (100 MHz, DMSO-*d*$_6$) δ 161.41, 146.62, 144.84, 143.39, 134.73, 132.56, 129.26, 127.34, 126.68, 126.65, 125.17, 54.89, 52.52, 52.30, 52.15, 38.12, 27.98. HRMS (ESI/Q-TOF) m/z: [M + H]$^+$ Calcd for $C_{18}H_{20}N_3O$ 294.1601; Found 294.1611.

4-Oxo-2-(2-(trifluoromethoxy)benzyl)-1,3,4,6,7,11*b*-hexahydro-*2H*-pyrazino[2,1-a]isoquinolin-2-ium Chloride (43).

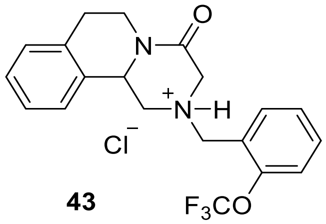

43

43 was synthesized according to the general reductive amination procedure with praziquanamine (0.202 g, 1.0 mmol), sodium triacetoxyborohydride (0.302 g, 1.4 mmol), and 2-nitrobenzaldehyde (0.218 g, 1.4 mmol) to yield a brown-orange oil. The ethyl acetate layer from the liquid-liquid extraction was extracted with 6 M HCl (3X, 2 mL). The combined acidic aqueous layers were washed with EtOAc (1X, 2 mL) and then the ammonium chloride salt was extracted with chloroform (3X, 2 mL). A solid formed in the 6M HCl layer. The solid was filtered and recrystallized from isopropanol to yield **43** as a white solid (0.025 g, 7%). ^{1}H NMR (400 MHz, DMSO-*d*$_6$) δ 7.45-7.26 (8H, m), 6.78 (1H, br s), 5.43 (1H, br s), 4.60 (1H, dt), 4.38 (1H, br s), 4.09-3.80 (3H, m), 3.26 (1H, br s), 2.98-2.78 (3H, m). ^{13}C NMR (100 MHz, DMSO-*d*$_6$, δ) 160.68, 136.01, 134.71, 132.20, 129.32, 129.00, 128.33, 127.46, 127.40, 126.78, 125.12, 64.51, 52.75, 51.66, 38.07, 27.98, 17.62. HRMS (ESI/Q-TOF) m/z: [M + H]$^+$ Calcd for $C_{20}H_{20}F_3N_2O_2$ 377.1477; Found 377.1486.

4-Oxo-2-(3-(trifluoromethoxy)benzyl)-1,3,4,6,7,11*b*-hexahydro-*2H*-pyrazino[2,1-a]isoquinolin-2-ium Chloride (44).

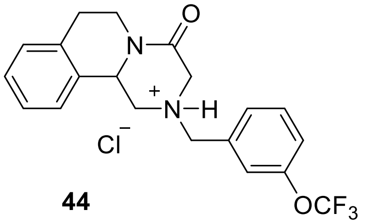

44

44 was synthesized according to the general reductive amination procedure with praziquanamine (0.1734 g, 0.857 mmol), sodium triacetoxyborohydride (0.257 g, 1.2 mmol), and 3-(trifluoromethoxy)benzaldehyde (0.184 mL, 1.285 mmol). Addition of HCl in ethyl acetate to the ethyl acetate solution yielded **44** as a white solid. (0.1786 g, 49%). ^{1}H NMR (400 MHz, DMSO-*d*$_6$) δ 12.49 (1H, br s), 7.72 (1H, s), 7.68 (1H, d), 7.62 (1H, t), 7.48 (1H, d), 7.32-7.21 (4H, m), 5.35 (1H, d), 4.55 (1H, dt), 4.55-4.28 (3H, m), 3.76 (1H, br d), 3.59 (1H, d), 3.17 (1H, br s), 2.90 (1H, m), 2.82-2.75 (2H, m). ^{13}C NMR (100 MHz, DMSO-*d*$_6$) δ 160.85, 148.37, 134.77, 132.22, 130.82, 130.37, 129.26, 127.36, 126.73, 124.95, 123.65, 121.94, 120.04 (q), 57.64, 52.38, 51.70, 51.39, 38.08, 27.90. HRMS (ESI/Q-TOF) m/z: [M + H]$^+$ Calcd for $C_{20}H_{20}F_3N_2O_2$ 377.1477; Found 377.1489.

4-Oxo-2-(4-(trifluoromethoxy)benzyl)-1,3,4,6,7,11*b*-hexahydro-*2H*-pyrazino[2,1-a]isoquinolin-2-ium Chloride (45).

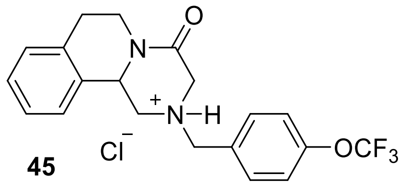

45

45 was synthesized according to the general reductive amination procedure with praziquanamine (0.2017 g, 1.0 mmol), sodium triacetoxyborohydride (0.2939 g, 1.4 mmol), and 4-trifluoromethoxy benzaldehyde (0.2 mL, 1.4 mmol). Addition of HCl in ethyl acetate to the ethyl acetate solution yielded a white solid. The solid was recrystallized from isopropanol to yield **45** as a white, powdery solid (0.21 g, 51%). ^{1}H NMR: (400 MHz MHz, DMSO-*d*$_6$) δ 7.80 (2H, d), 7.47 (2H, d), 7.31-7.21 (4H, m), 5.39 (1H, d), 4.55 (1H, dt), 4.55-4.36 (1H, m), 3.82 (1H, d), 3.60 (1H, d), 3.21 (1H, br s), 2.89 (1H, m), 2.82-2.74 (2H, m). ^{13}C NMR: (125 MHz MHz, DMSO-*d*$_6$) δ 160.59, 149.13, 134.75, 133.62, 132.08, 129.27, 127.38, 126.75, 125.03, 121.18, 119.99 (q), 57.33, 52.12, 51.56, 51.00, 38.07, 27.89. HRMS (ESI/Q-TOF) m/z: [M + H]$^+$ Calcd for $C_{20}H_{20}F_3N_2O_2$ 377.1477; Found 377.1478.

4-Oxo-2-(2-(trifluoromethyl)benzyl)-1,3,4,6,7,11*b*-hexahydro-*2H*-pyrazino[2,1-a]isoquinolin-2-ium Chloride (46).

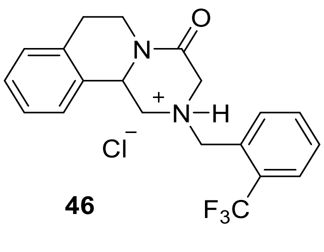

46

46 was synthesized according to the general reductive amination procedure with praziquanamine (0.2046 g, 1.0 mmol), sodium triacetoxyborohydride (0.3035 g, 1.4 mmol), and 2-trifluoromethyl benzaldehyde (0.19 mL, 1.4 mmol). Addition of aq. 2 M HCl to the ethyl acetate solution yielded a solid. The solid was recrystallized from isopropanol to yield **46** as a white solid (0.1847 g, 47%). ^{1}H NMR (400 MHz, DMSO-*d*$_6$) δ 8.21 (1H, d), 7.82 (1H, t), 7.79 (1H, d), 7.65 (1H, t), 7.28-7.18 (4H, m), 5.27 (1H, d), 4.55 (1H, dt), 4.41 (1H, d), 4.35 (1H, d), 4.04 (1H, d), 3.61 (2H, s), 3.19 (1H, s), 2.90 (1H, m), 2.84-2.76 (2H, m). ^{13}C NMR (400MHz, MHz, DMSO-*d*$_6$) δ 162.72, 134.76, 133.02, 132.82, 132.41, 129.44, 129.20, 128.03 (q), 127.27, 126.67, 126.33 (q), 124.93, 124.05 (q), 55.46, 53.47, 53.36, 52.42, 38.18, 27.98. HRMS (ESI/Q-TOF) m/z: [M + H]$^+$ Calcd for $C_{20}H_{20}F_3N_2O$ 361.1528; Found 361.1535.

4-Oxo-2-(3-(trifluoromethyl)benzyl)-1,3,4,6,7,11*b*-hexahydro-*2H*-pyrazino[2,1-a]isoquinolin-2-ium Chloride (47).

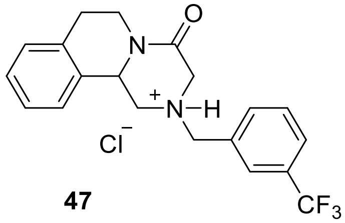

47

47 was synthesized according to the general reductive amination procedure with praziquanamine (0.2058 g, 1.0 mmol), sodium triacetoxyborohydride (0.3122 g, 1.4 mmol), and 3-trifluoromethyl benzaldehyde (0.19 mL, 1.4 mmol). Addition of aq. 2 M HCl to the ethyl acetate solution yielded a solid. The solid was recrystallized from isopropanol to yield **47** as a white solid (0.1071 g, 27% yield). ^{1}H NMR (400 MHz, DMSO-*d*$_6$) δ 8.07 (1 H, s), 7.97 (1H, d), 7.83 (1H, d), 7.71 (1H, t), 7.31-7.21 (4H, m), 5.36 (1H, d), 4.60-4.31 (3H, m), 4.55 (2H, dt), 3.82 (1H, d), 3.58 (1H, d), 3.21 (1H, br s), 2.90 (1H, m), 2.82-2.76 (2H, m). ^{13}C NMR (400 MHz, DMSO-*d*$_6$) δ 160.75, 135.45, 134.77, 132.16, 129.90, 129.42 (q), 129.28, 128.95, 128.03 (q), 127.38, 126.75, 126.24, 125.00, 124.00 (q), 57.75, 52.36, 51.66, 51.31, 38.08, 27.90. HRMS (ESI/Q-TOF) m/z: [M + H]$^+$ Calcd for $C_{20}H_{20}F_3N_2O$ 361.1528; Found 361.1527.

4-Oxo-2-(4-(trifluoromethyl)benzyl)-1,3,4,6,7,11*b*-hexahydro-*2H*-pyrazino[2,1-a]isoquinolin-2-ium Chloride (48).

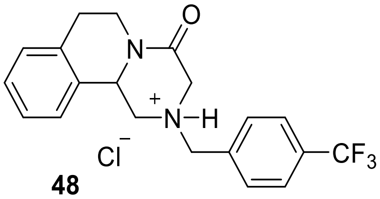

48

48 was synthesized according to the general reductive amination procedure with praziquanamine (0.2022 g, 1.0 mmol), sodium triacetoxyborohydride (0.2958 g, 1.4 mmol), and 4-(trifluoromethyl)benzaldehyde (0.19 mL, 1.4 mmol). Addition of aq. 2 M HCl to the ethyl acetate solution yielded a solid. The solid was recrystallized from isopropanol to yield **8** as a white solid (0.13g, 33%). ^{1}H NMR: (400 MHz, DMSO-*d*$_6$) δ 7.91-7.82 (4H, m), 7.27 (4H, m), 5.36 (1H, d), 4.59-4.31 (4H, m), 3.78 (2H, br d), 3.62 (1H, d), 3.18 (1H, br s), 2.89 (1H, m), 2.82-2.74 (2H, m). ^{13}C NMR: (125 MHz, DMSO-*d*$_6$) δ 160.77, 134.75, 132.19, 129.77 (q), 129.25, 127.53, 126.74, 125.65, 125.63, 125.04, 124.05 (q), 57.63, 52.31, 51.69, 51.38, 39.72,

38.06, 27.91. HRMS (ESI/Q-TOF) m/z: $[M + H]^+$ Calcd for $C_{20}H_{20}F_3N_2O$ 361.1528; Found 361.1530.

2-(3-Fluoro-4-(trifluoromethyl)benzyl)-4-oxo-1,3,4,6,7,11*b*-hexahydro-*2H*-pyrazino[2,1-a]isoquinolin-2-ium Chloride (49).

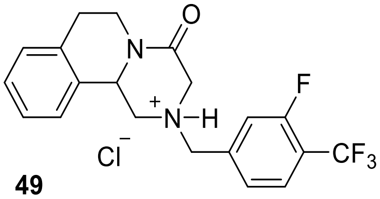

49

49 was synthesized according to the general reductive amination procedure with praziquanamine (0.2146 g, 1.0 mmol), sodium triacetoxyborohydride (0.3108 g, 1.5 mmol), and 3-fluoro-4-trifluoromethyl benzaldehyde (0.2771 g, 1.4 mmol). Addition of aq. 2 M HCl to the ethyl acetate solution yielded a solid. The solid was recrystallized from isopropanol to yield **49** as a white solid (0.1076 g, 24% yield). ^{1}H NMR (400 MHz, DMSO-d_6) δ 7.93-7.84 (2H, m), 7.67 (1H, d), 7.31-7.21 (4H, m), 5.33 (1H, d), 4.59-4.24 (4H, m), 3.79-3.65 (2H, m), 2.15 (1H, br t), 2.90 (1H, m), 2.81-2.75 (2H, m). ^{13}C NMR (400 MHz, DMSO-d_6) δ 161.97, 158.68 (d), 137.99, 134.74, 132.33, 129.24, 127.76, 127.34, 126.71, 125.11, 122.52 (q), 119.70 (d), 117.26 (q), 57.14, 52.35, 51.81, 38.10, 27.94. HRMS (ESI/Q-TOF) m/z: $[M + H]^+$ Calcd for $C_{20}H_{19}F_4N_2O$ 379.1434; Found 379.1435.

2-(2-Fluoro-5-(trifluoromethoxy)benzyl)-1,2,3,6,7,11*b*-hexahydro-*4H*-pyrazino[2,1-a]isoquinolin-4-one (50).

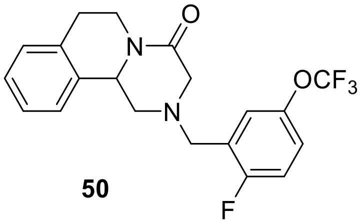

50

50 was synthesized according to the general reductive amination procedure with praziquanamine (0.2024 g, 1.0 mmol), sodium triacetoxyborohydride (0.2994 g, 1.4 mmol), and 2-methoxy-5-trifluoromethoxy benzaldehyde (0.3112 g, 1.4 mmol). The dried ethyl acetate layer was condensed under reduced pressure. The residue was purified by column chromatography on silica gel (Hexanes:1% NH_3 in Isopropanol, 0-20%). The product fractions were combined and condensed under reduced pressure. Ethyl acetate was added to the residue followed by HCl in ethyl acetate. Hexanes was added and the solution was kept at 0 °C overnight to yield **50** as a pale yellow solid (0.1815 g, 42% yield). ^{1}H NMR (400 MHz, $CDCl_3$) δ 7.36 (1H, dd), 7.23-7.19 (2H, m), 7.18-7.13 (2H, m), 7.11 (1H, t), 7.06 (1H, dd), 4.89 (1H, dd), 4.80 (1H, m), 3.73 (1H, d), 3.68 (1H, d), 3.58-3.48 (2H, m), 3.03 (1H, d), 2.99-2.86 (2H, m), 2.75 (1H, m), 2.44 (1H, dd). ^{13}C NMR (400 MHz, $CDCl_3$) δ 165.90, 159.17 (d), 145.04, 134.91, 133.99, 129.28, 127.04, 126.56, 125.55 (d), 124.33, 123.53 (d), 121.87 (d), 120.34 (q), 116.60 (d), 56.73, 55.96, 55.43, 53.56, 38.65, 28.68. HRMS (ESI/Q-TOF) m/z: $[M + H]^+$ Calcd for $C_{20}H_{19}F_4N_2O_2$ 395.1383; Found 395.1390.

2-(2-Methyl-4-(trifluoromethoxy)benzyl)-4-oxo-1,3,4,6,7,11*b*-hexahydro-*2H*-pyrazino[2,1-a]isoquinolin-2-ium Chloride (51).

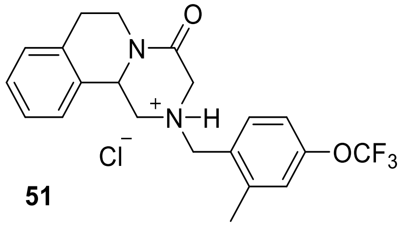

51

51 was synthesized according to the general reductive amination procedure with praziquanamine (0.2024 g, 1.0 mmol), sodium triacetoxyborohydride (0.2971 g, 1.4 mmol), and 2-methyl-4-(trifluoromethoxy)benzaldehyde (0.2868 mL, 1.4 mmol). Addition of HCl in ethyl acetate to the ethyl acetate solution yielded **51** as a white solid (0.1686 g, 39%). ^{1}H NMR (400 MHz, $CDCl_3$) δ 7.98 (1H, d), 7.30-7.27 (2H, m), 7.22-7.12 (4H, m), 5.85 (1H, br s), 4.78 (1H, d), 4.54 (1H, d), 4.39 (1H, d), 4.29 (1H, d), 4.03 (1H, d), 3.50 (1H, d), 3.04-2.86 (4H, m). ^{13}C NMR (100 MHz, $CDCl_3$) δ 159.59, 150.52, 140.64, 134.66, 134.32, 130.52, 129.75, 128.15, 127.35, 125.17, 124.06, 123.38, 120.27 (q), 119.37, 56.23, 54.25, 51.67, 50.85, 38.87, 28.46, 20.40. HRMS (ESI/Q-TOF) m/z: $[M + H]^+$ Calcd for $C_{21}H_{22}F_3N_2O_2$ 391.1628; Found 391.1637.

2-(2-Methoxy-4-(trifluoromethoxy)benzyl)-4-oxo-1,3,4,6,7,11*b*-hexahydro-*2H*-pyrazino[2,1-a]isoquinolin-2-ium Chloride (52).

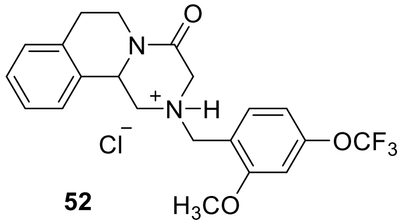

52

52 was synthesized according to the general reductive amination procedure with praziquanamine (0.2027 g, 1.0 mmol), sodium triacetoxyborohydride (0.3006 g, 1.4 mmol), and 2-methoxy-4-trifluoromethoxybenzaldehyde (0.3185 g, 1.4 mmol). The dried ethyl acetate layer was condensed under reduced pressure. The residue was purified by column chromatography on silica gel (Hexanes:1% NH_3 in Isopropanol, 0-20%). The product fractions were combined and condensed under reduced pressure. Ethyl acetate was added to the residue followed by HCl in ethyl acetate. Hexanes was added and the solution was kept at 0 °C overnight to yield **52** as a white solid (0.3801 g, 86% yield). ^{1}H NMR (400 MHz, $CDCl_3$) δ 7.77 (1H, d), 7.33-7.21 (4H, m), 7.13 (1H, d), 7.05 (1H, d), 5.38 (1H, br d), 4.56 (1H, dt), 4.51-4.32 (3H, m), 3.89 (3H, s), 3.82 (1H, d), 3.66 (1H, d), 3.28 (1H, br s), 2.93-2.74 (3H, m). ^{13}C NMR (100 MHz, DMSO-d_6) δ 160.64, 159.53, 150.56, 134.86, 134.76, 132.21, 129.28, 127.37, 126.77, 125.05, 120.01 (q), 112.26, 105.17, 56.35, 52.43, 52.11, 51.63, 51.21, 38.04, 27.92. HRMS (ESI/Q-TOF) m/z: $[M + H]^+$ Calcd for $C_{21}H_{22}F_3N_2O_3$ 407.1577; Found 407.1583.

2-(4-Methoxy-3-(trifluoromethoxy)benzyl)-4-oxo-1,3,4,6,7,11*b*-hexahydro-*2H*-pyrazino[2,1-a]isoquinolin-2-ium Chloride (53).

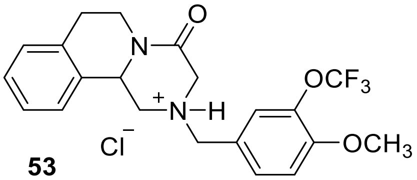

53

53 was synthesized according to the general reductive amination procedure with praziquanamine (0.2038 g, 1.0 mmol), sodium triacetoxyborohydride (0.2955 g, 1.4 mmol), and 4-methoxy-3-trifluoromethoxy benzaldehyde (0.3100 g, 1.4 mmol). The ethyl acetate layer was washed with HCl (2M). Solid formed in the ethyl acetate layer, which was vacuum filtered to yield **53** a white solid (0.0771 g, 17% yield). ^{1}H NMR (400 MHz, $CDCl_3$) δ 7.75 (1H, s), 7.67 (1H, d), 7.34 (1H, d), 7.29-7.21 (4H, m), 5.39 (1H, d), 4.58-4.31 (4H, m), 3.89 (3H, s), 3.85 (1H, d), 3.60 (1H, d), 3.20 (1H, br s), 2.93-2.75 (3H, m). ^{13}C NMR (400 MHz, DMSO-d_6) δ 160.55, 152.49, 136.50, 134.77, 132.27, 132.04, 129.29, 127.40, 126.76, 126.00, 124.99, 124.07, 121.30, 120.24 (q), 113.85, 57.17, 56.26, 51.87, 51.52, 50.69, 38.08, 27.90. HRMS (ESI/Q-TOF) m/z: [M + H]$^+$ Calcd for $C_{21}H_{22}F_3N_2O_3$ 407.1577; Found 407.1584.

Single-Crystal X-ray Crystallography

Single crystal growth. Crystals of **4** suitable for single-crystal X-ray diffraction were grown by slow evaporation of a solution in a mixture of ethyl acetate and isopropanol at room temperature.

Single Crystal X-Ray Diffraction and Structural Refinement. The diffraction data on single crystals were collected at 298 K using a Bruker D8 Venture IμS microfocus dual-source diffractometer equipped with a PHOTON II CPAD detector and an Oxford cryogenic system. Monochromatic Cu Kα (λ= 1.54178 Å) radiation was used in the data collection using phi (φ) and omega (ω) scan strategies. Cell measurement, data collection, integration, scaling, and absorption correction were performed using SADABS programs incorporated in APEX6.[5] The structure was solved using SHELXT[6] and refined using SHELXL[7] programs, both implemented in OLEX2.[8] The non-hydrogen atoms were located in successive difference Fourier syntheses and refined with anisotropic thermal parameters. The CH hydrogen atoms were placed at calculated positions and refined using a riding model with appropriate HFIX commands and U_{iso} = 1.2 x U_{eqiv}. NH hydrogen atom was located in different Fourier syntheses (NH, 1.2 x U_{eqiv}), and their positions were refined independently. The program OLEX2 was used for molecular images. Crystallographic data is summarized in Table S1. Crystallographic data for the crystal structure has been deposited with the Cambridge Crystallographic Data Centre as supplementary publication no. CCDC 2531889. Copies of the data can be obtained free of charge on application to CCDC, 12 Union Road, Cambridge CB2 1EZ, U.K. (fax: (+44)1223-336-033; e-mail: deposit@ccdc.cam.ac.uk; www: http://www.ccdc.cam.ac.uk).

Table S1. The data collection, processing, and refinement statistics of **4**.

**Compound**	**4**
**Crystal Data**	
CCDC	
Chemical Formula	$C_{19}H_{21}ClN_2O$
Formula Weight	328.83
Crystal system	Triclinic
Space group	P-1
a [Å]	9.3942(8)
b [Å]	10.3425(8)
c [Å]	10.4094(8)
α [deg]	100.038(3)
β [deg]	105.575(3)
γ [deg]	112.531(3)
V [Å^3]	855.09(12)
Z, Z’	2, 1
D_{calc} (g cm^{-3})	1.277
μ (mm^{-1}), rad. type	1.54178, Cu Kα
F_{000}	348
temp (K)	298
Crystal form, color	Block, colorless
Crystal size, mm	0.127 x 0.177 x 0.233
**Data Collection**	
Diffractometer	Bruker D8 Venture
T_{min}/T_{max}	0.651/0.784
No. of refls. (meas., uniq., and obs.)	29848, 3655, 3244
R_{int}	0.0398
Θ_{max} (°)	79.22
**Refinement**	
R/R^2_{ω} (obs data)	0.0428/0.1124
R/R^2_{ω} (all data)	0.0481/0.1187
S	1.104
No. of refls.	3655
No. of parameters	296
$\Delta\rho_{max/min}$ (e·Å^{-3})	0.18/-0.21

References

(1) Wang, W. L.; Song, L. J.; Chen, X.; Yin, X. R.; Fan, W. H.; Wang, G. P.; Yu, C. X.; Feng, B. Synthesis and SAR Studies of Praziquantel Derivatives with Activity against Schistosoma Japonicum. *Molecules* **2013**, *18* (8), 9163–9178. https://doi.org/10.3390/molecules18089163.

(2) Sadhu, P. S.; Kumar, S. N.; Chandrasekharam, M.; Pica-Mattoccia, L.; Cioli, D.; Rao, V. J. Synthesis of New Praziquantel Analogues: Potential Candidates for the Treatment of Schistosomiasis. *Bioorganic & Medicinal Chemistry Letters* **2012**, *22* (2), 1103–1106. https://doi.org/10.1016/j.bmcl.2011.11.108.

(3) Tang, H.; Zheng, C.; Lv, J.; Wu, J.; Li, Y.; Yang, H.; Fu, B.; Li, C.; Zhou, Y.; Zhu, J. Synthesis and Antifungal Activities in Vitro of Novel Pyrazino [2,1-a] Isoquinolin Derivatives. *Bioorganic & Medicinal Chemistry Letters* **2010**, *20* (3), 979–982. https://doi.org/10.1016/j.bmcl.2009.12.050.

(4) Arnáiz, F. J. A Convenient Way to Generate Hydrogen Chloride in the Freshman Lab. *Journal of Chemical Education* **1995**, *72* (12), 1139. https://doi.org/10.1021/ed072p1139.

(5) APEX6 Version 2025.6-0 Data Collection (Includes SAINT V8.40B, SADABS 2016/2, and XPREP Version 2014/2), 2024.

(6) Sheldrick, G. M. SHELXT – Integrated Space-Group and Crystal-Structure Determination. *Acta Cryst A* **2015**, *71* (1), 3–8. https://doi.org/10.1107/S2053273314026370.

(7) Sheldrick, G. M. Crystal Structure Refinement with SHELXL. *Acta Cryst C* **2015**, *71* (1), 3–8. https://doi.org/10.1107/S2053229614024218.

(8) Dolomanov, O. V.; Bourhis, L. J.; Gildea, R. J.; Howard, J. a. K.; Puschmann, H. OLEX2: A Complete Structure Solution, Refinement and Analysis Program. *J Appl Cryst* **2009**, *42* (2), 339–341. https://doi.org/10.1107/S0021889808042726.

Figure S4: 1H NMR Spectrum of Compound 4.

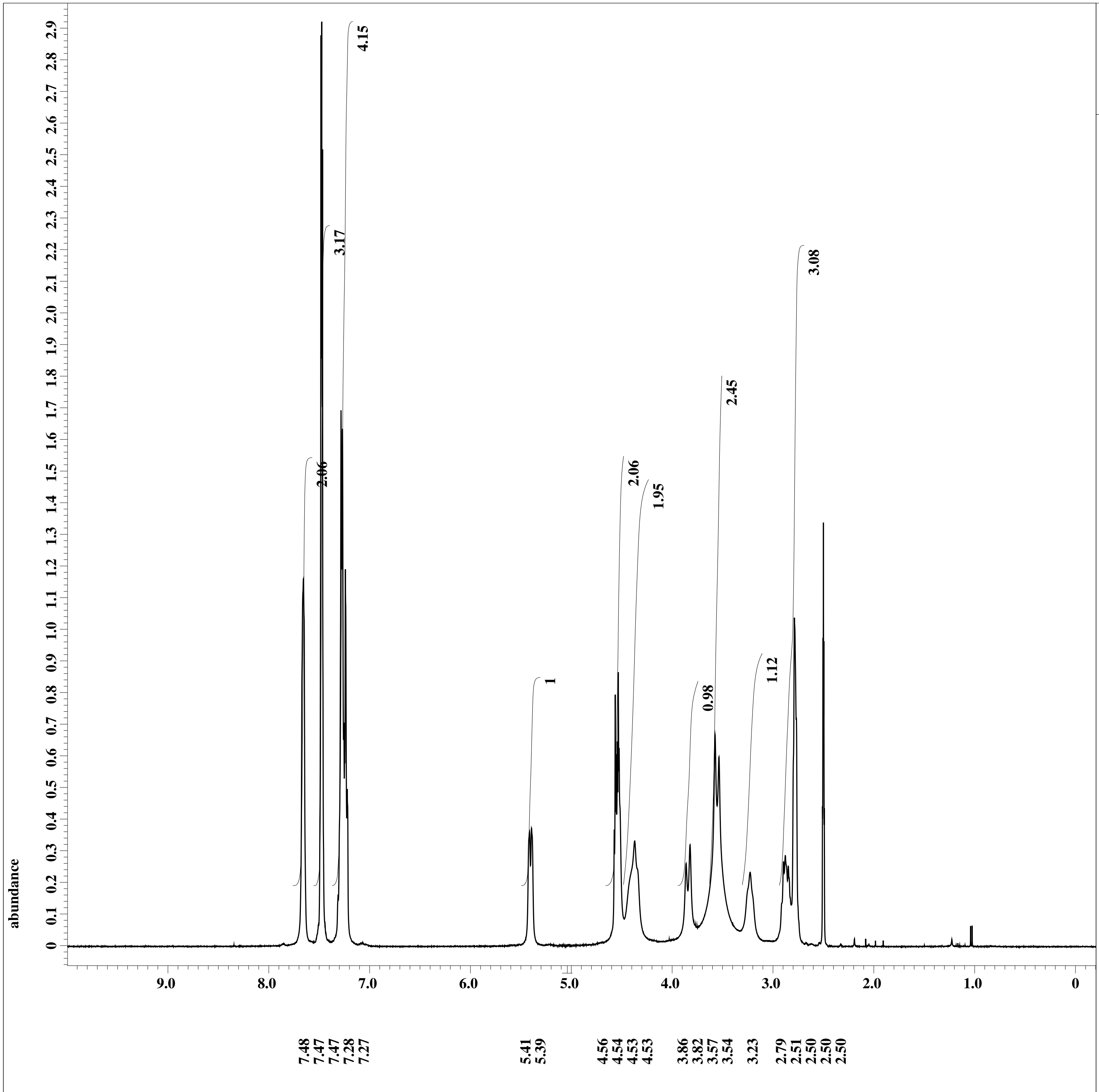

Filename	= TAR-2-011 crystals-4.
Author	= russell research stud
Experiment	= single_pulse.ex2
Sample_id	= S#542601
Solvent	= DMSO-D6
Creation_time	= 9-FEB-2026 13:54:54
Revision_time	= 10-FEB-2026 11:54:42
Current_time	= 10-FEB-2026 11:55:20
Comment	= single_pulse
Data_format	= 1D COMPLEX
Dim_size	= 13107
Dim_title	= 1H
Dim_units	= [ppm]
Dimensions	= X
Site	= ECS 400
Spectrometer	= JNM-ECS400
Field_strength	= 9.389766[T] (400[MHz]
X_acq_duration	= 2.18365952[s]
X_domain	= 1H
X_freq	= 399.78219838[MHz]
X_offset	= 5[ppm]
X_points	= 16384
X_prescans	= 1
X_resolution	= 0.45794685[Hz]
X_sweep	= 7.5030012[kHz]
Irr_domain	= 1H
Irr_freq	= 399.78219838[MHz]
Irr_offset	= 5[ppm]
Tri_domain	= 1H
Tri_freq	= 399.78219838[MHz]
Tri_offset	= 5[ppm]
Clipped	= FALSE
Mod_return	= 1
Scans	= 8
Total_scans	= 8
X_90_width	= 14[us]
X_acq_time	= 2.18365952[s]
X_angle	= 45[deg]
X_atn	= 3[dB]
X_pulse	= 7[us]
Irr_mode	= Off
Tri_mode	= Off
Dante_presat	= FALSE
Initial_wait	= 1[s]
Recvr_gain	= 38
Relaxation_delay	= 5[s]
Repetition_time	= 7.18365952[s]
Temp_get	= 20.5[dC]

Figure S5: 1H NMR Spectrum of Compound 4.

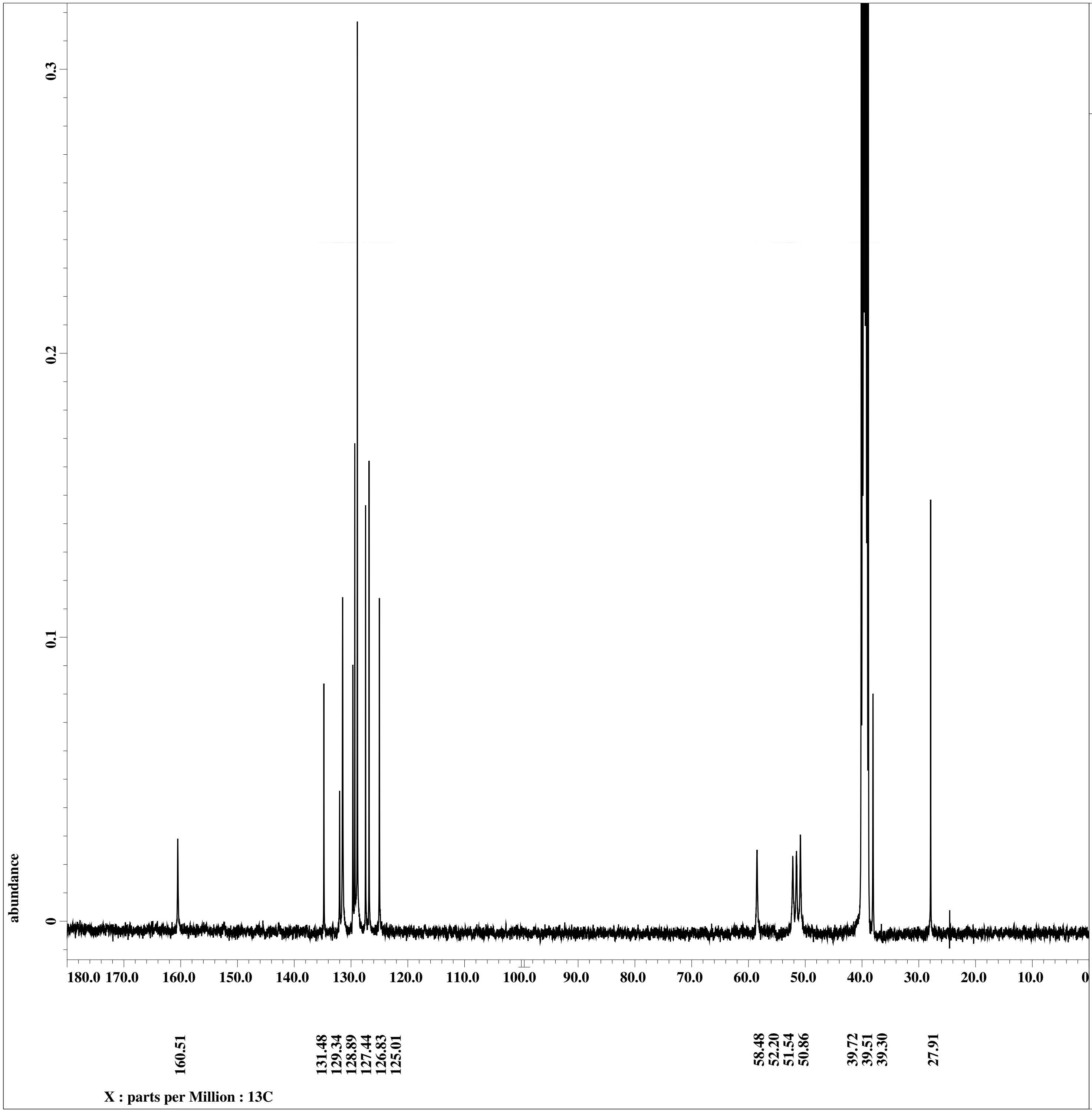

Filename	= TAR-2-011 crystals 13
Author	= russell research stud
Experiment	= single_pulse_dec
Sample_id	= S#546141
Solvent	= DMSO-D6
Creation_time	= 10-FEB-2026 02:34:36
Revision_time	= 10-FEB-2026 11:57:13
Current_time	= 10-FEB-2026 11:57:49
Comment	= single pulse decouple
Data_format	= 1D COMPLEX
Dim_size	= 26214
Dim_title	= 13C
Dim_units	= [ppm]
Dimensions	= X
Site	= ECS 400
Spectrometer	= JNM-ECS400
Field_strength	= 9.389766[T] (400[MHz]
X_acq_duration	= 1.04333312[s]
X_domain	= 13C
X_freq	= 100.52530333[MHz]
X_offset	= 100[ppm]
X_points	= 32768
X_prescans	= 4
X_resolution	= 0.95846665[Hz]
X_sweep	= 31.40703518[kHz]
Irr_domain	= 1H
Irr_freq	= 399.78219838[MHz]
Irr_offset	= 5[ppm]
Clipped	= FALSE
Mod_return	= 1
Scans	= 4096
Total_scans	= 4096
X_90_width	= 9.82623[us]
X_acq_time	= 1.04333312[s]
X_angle	= 30[deg]
X_atn	= 5.8[dB]
X_pulse	= 3.27541[us]
Irr_atn_dec	= 21[dB]
Irr_atn_noe	= 21[dB]
Irr_noise	= WALTZ
Decoupling	= TRUE
Initial_wait	= 1[s]
Noe	= TRUE
Noe_time	= 10[s]
Recvr_gain	= 60
Relaxation_delay	= 10[s]
Repetition_time	= 11.04333312[s]
Temp_get	= 21.3[dC]

Figure S6: 1H NMR Spectrum of Compound 5.

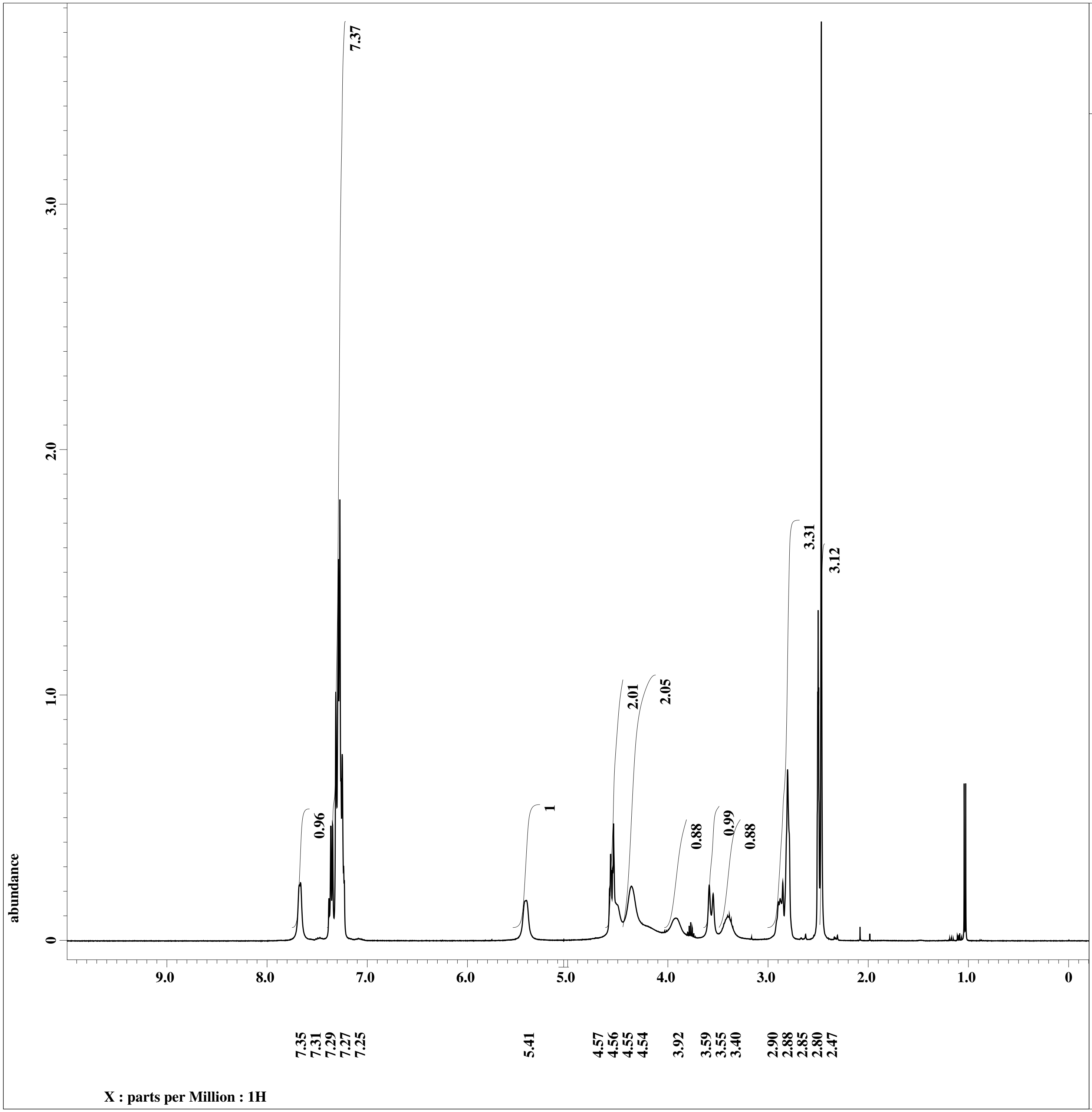


```
Filename          = BDG-II-073 (DMSO) 1H
Author            = chemlabs
Experiment        = single_pulse.ex2
Sample_id         = 001
Solvent           = DMSO-D6
Creation_time     = 22-JUL-2017 19:56:59
Revision_time     = 19-JUL-2021 10:26:36
Current_time      = 19-JUL-2021 10:26:51

Comment           = single_pulse
Data_format       = 1D COMPLEX
Dim_size          = 13107
Dim_title         = 1H
Dim_units         = [ppm]
Dimensions        = X
Site              = ECS 400
Spectrometer      = JNM-ECS400

Field_strength    = 9.389766[T] (400[MHz]
X_acq_duration    = 2.18365952[s]
X_domain          = 1H
X_freq            = 399.78219838[MHz]
X_offset          = 5[ppm]
X_points          = 16384
X_prescans        = 1
X_resolution      = 0.45794685[Hz]
X_sweep           = 7.5030012[kHz]
Irr_domain        = 1H
Irr_freq          = 399.78219838[MHz]
Irr_offset        = 5[ppm]
Tri_domain        = 1H
Tri_freq          = 399.78219838[MHz]
Tri_offset        = 5[ppm]
Clipped           = FALSE
Mod_return        = 1
Scans             = 8
Total_scans       = 8

X_90_width        = 14.3[us]
X_acq_time        = 2.18365952[s]
X_angle           = 45[deg]
X_atn             = 3[dB]
X_pulse           = 7.15[us]
Irr_mode          = Off
Tri_mode          = Off
Dante_presat      = FALSE
Initial_wait      = 1[s]
Recvr_gain        = 34
Relaxation_delay  = 5[s]
Repetition_time   = 7.18365952[s]
Temp_get          = 23.9[dC]
```

Figure S7: 13C NMR Spectrum of Compound 5.

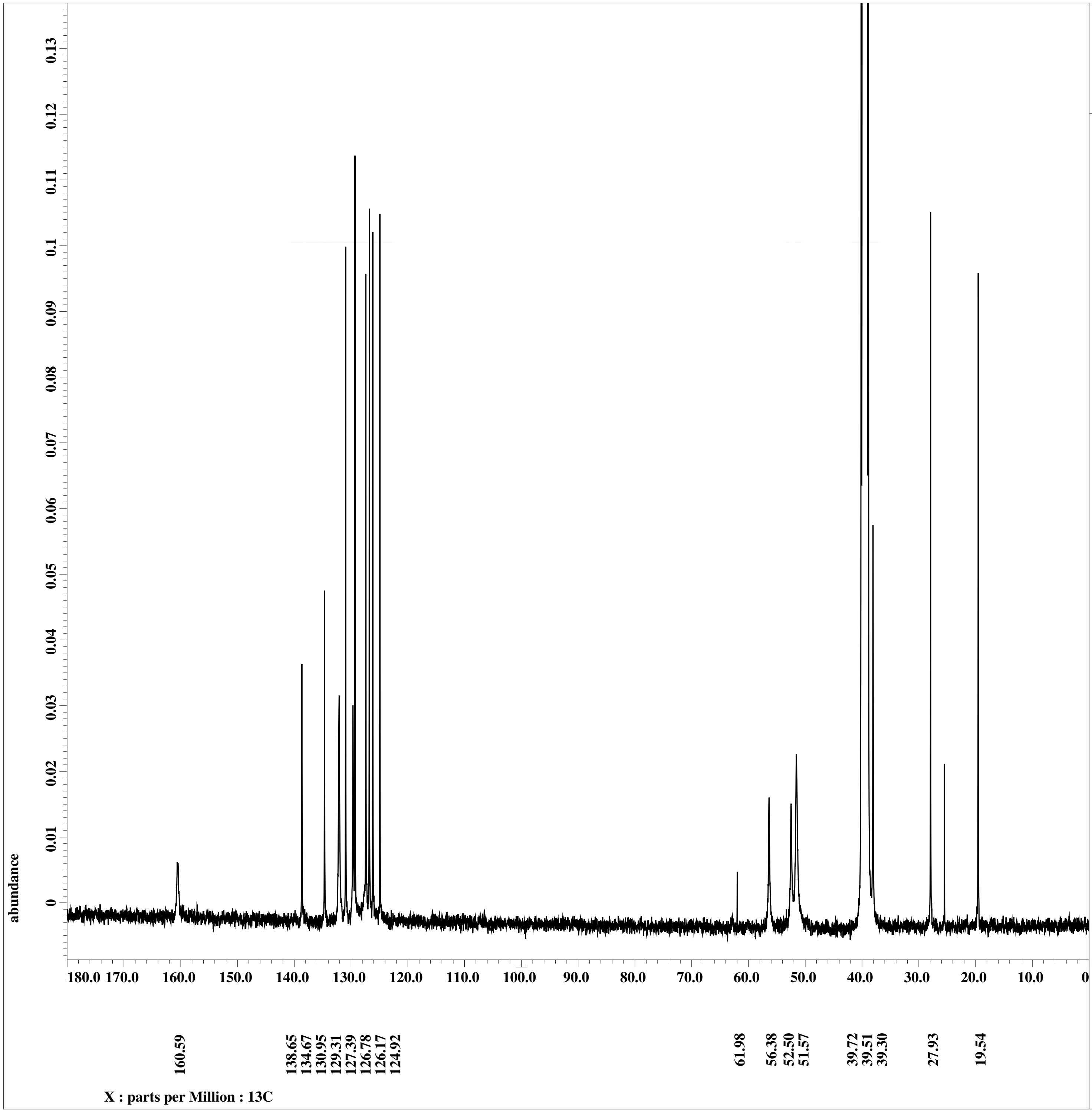

Filename	= BDG-II-073 (DMSO) 13C
Author	= chemlabs
Experiment	= single_pulse_dec
Sample_id	= 001
Solvent	= DMSO-D6
Creation_time	= 23-JUL-2017 07:53:30
Revision_time	= 19-JUL-2021 10:30:40
Current_time	= 19-JUL-2021 10:37:01
Comment	= single pulse decouple
Data_format	= 1D COMPLEX
Dim_size	= 26214
Dim_title	= 13C
Dim_units	= [ppm]
Dimensions	= X
Site	= ECS 400
Spectrometer	= JNM-ECS400
Field_strength	= 9.389766[T] (400[MHz]
X_acq_duration	= 1.04333312[s]
X_domain	= 13C
X_freq	= 100.52530333[MHz]
X_offset	= 100[ppm]
X_points	= 32768
X_prescans	= 4
X_resolution	= 0.95846665[Hz]
X_sweep	= 31.40703518[kHz]
Irr_domain	= 1H
Irr_freq	= 399.78219838[MHz]
Irr_offset	= 5[ppm]
Clipped	= FALSE
Mod_return	= 1
Scans	= 14097
Total_scans	= 14097
X_90_width	= 9.82623[us]
X_acq_time	= 1.04333312[s]
X_angle	= 30[deg]
X_atn	= 5.8[dB]
X_pulse	= 3.27541[us]
Irr_atn_dec	= 21[dB]
Irr_atn_noe	= 21[dB]
Irr_noise	= WALTZ
Decoupling	= TRUE
Initial_wait	= 1[s]
Noe	= TRUE
Noe_time	= 2[s]
Recvr_gain	= 60
Relaxation_delay	= 2[s]
Repetition_time	= 3.04333312[s]
Temp_get	= 24.2[dC]

Figure S8: 1H NMR Spectrum of Compound 6.

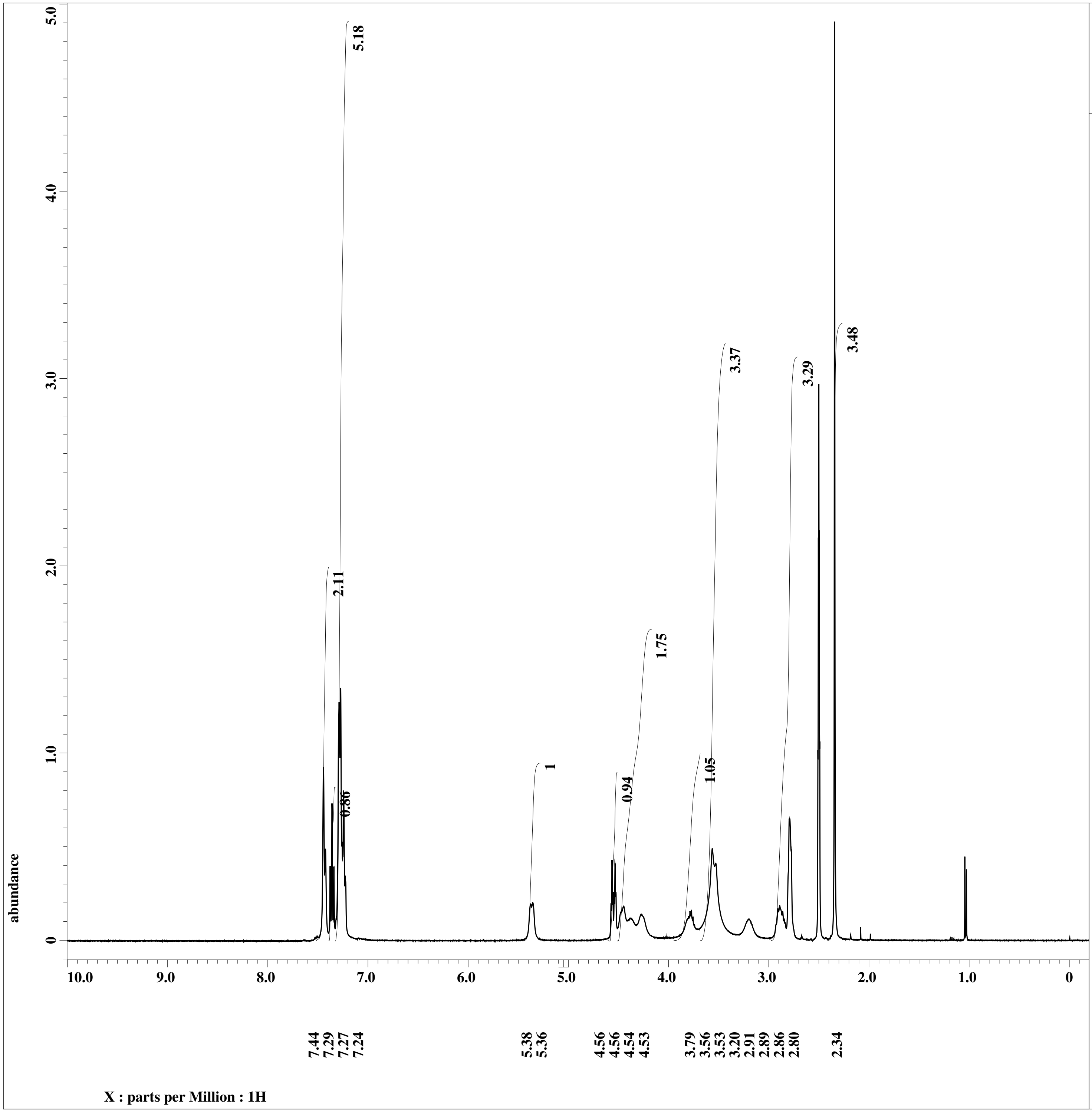

Filename	= SMW-1-011 crystals co
Author	= chemlabs
Experiment	= single_pulse.ex2
Sample_id	= 001
Solvent	= DMSO-D6
Creation_time	= 18-JUL-2017 15:38:11
Revision_time	= 19-JUL-2021 10:46:08
Current_time	= 19-JUL-2021 10:50:35
Comment	= single_pulse
Data_format	= 1D COMPLEX
Dim_size	= 13107
Dim_title	= 1H
Dim_units	= [ppm]
Dimensions	= X
Site	= ECS 400
Spectrometer	= JNM-ECS400
Field_strength	= 9.389766[T] (400[MHz]
X_acq_duration	= 2.18365952[s]
X_domain	= 1H
X_freq	= 399.78219838[MHz]
X_offset	= 5[ppm]
X_points	= 16384
X_prescans	= 1
X_resolution	= 0.45794685[Hz]
X_sweep	= 7.5030012[kHz]
Irr_domain	= 1H
Irr_freq	= 399.78219838[MHz]
Irr_offset	= 5[ppm]
Tri_domain	= 1H
Tri_freq	= 399.78219838[MHz]
Tri_offset	= 5[ppm]
Clipped	= FALSE
Mod_return	= 1
Scans	= 8
Total_scans	= 8
X_90_width	= 14.3[us]
X_acq_time	= 2.18365952[s]
X_angle	= 45[deg]
X_atn	= 3[dB]
X_pulse	= 7.15[us]
Irr_mode	= Off
Tri_mode	= Off
Dante_presat	= FALSE
Initial_wait	= 1[s]
Recvr_gain	= 42
Relaxation_delay	= 5[s]
Repetition_time	= 7.18365952[s]
Temp_get	= 24.5[dC]

Figure S9: 13C NMR Spectrum of Compound 6.

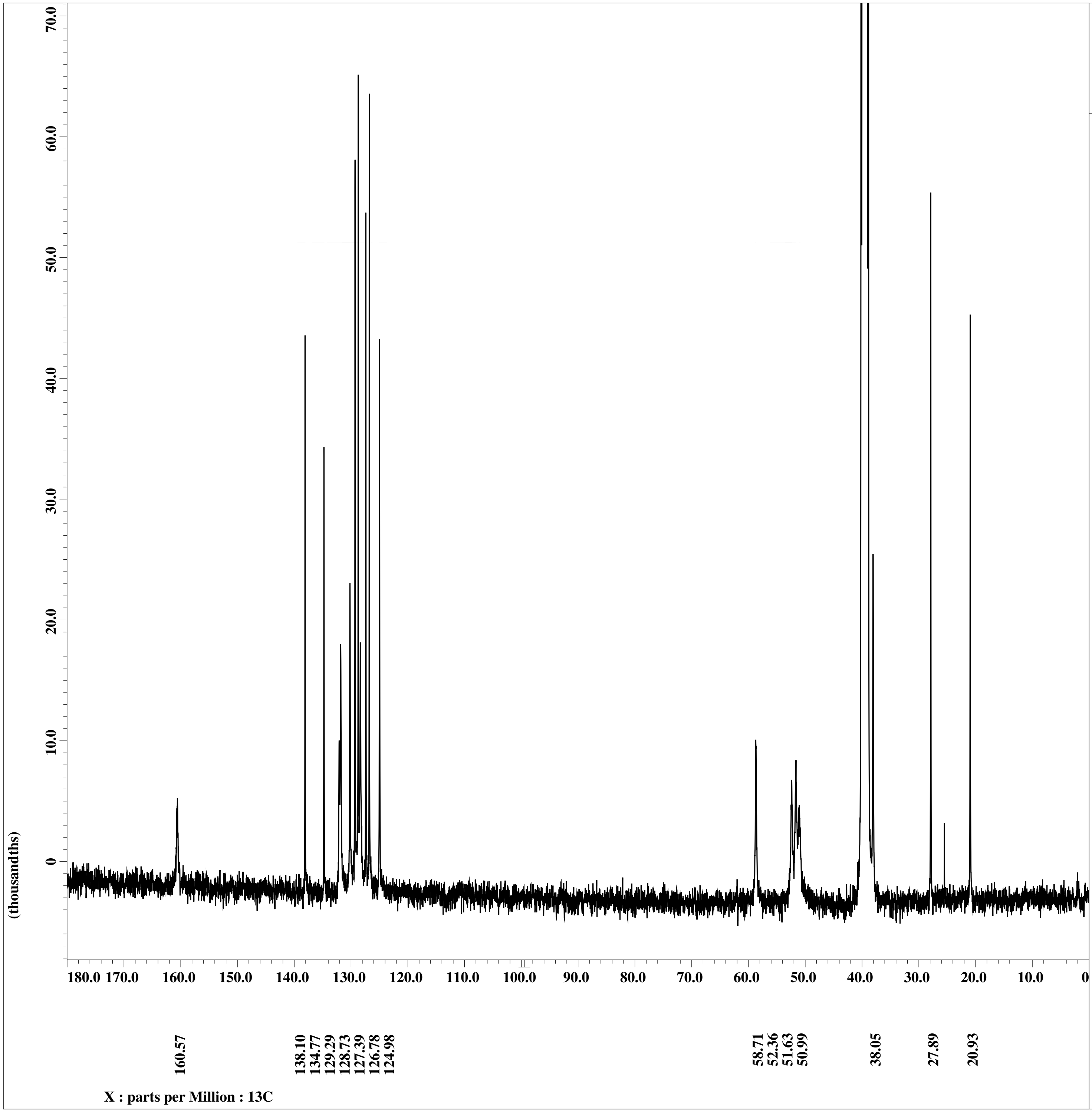

Filename	= SMW-1-011 crystals co
Author	= chemlabs
Experiment	= single_pulse_dec
Sample_id	= 001
Solvent	= DMSO-D6
Creation_time	= 19-JUL-2017 05:31:29
Revision_time	= 19-JUL-2021 10:53:47
Current_time	= 19-JUL-2021 10:58:11
Comment	= single pulse decouple
Data_format	= 1D COMPLEX
Dim_size	= 26214
Dim_title	= 13C
Dim_units	= [ppm]
Dimensions	= X
Site	= ECS 400
Spectrometer	= JNM-ECS400
Field_strength	= 9.389766[T] (400[MHz]
X_acq_duration	= 1.04333312[s]
X_domain	= 13C
X_freq	= 100.52530333[MHz]
X_offset	= 100[ppm]
X_points	= 32768
X_prescans	= 4
X_resolution	= 0.95846665[Hz]
X_sweep	= 31.40703518[kHz]
Irr_domain	= 1H
Irr_freq	= 399.78219838[MHz]
Irr_offset	= 5[ppm]
Clipped	= TRUE
Mod_return	= 1
Scans	= 16384
Total_scans	= 16384
X_90_width	= 9.4712[us]
X_acq_time	= 1.04333312[s]
X_angle	= 30[deg]
X_atn	= 5.8[dB]
X_pulse	= 3.15706667[us]
Irr_atn_dec	= 21[dB]
Irr_atn_noe	= 21[dB]
Irr_noise	= WALTZ
Decoupling	= TRUE
Initial_wait	= 1[s]
Noe	= TRUE
Noe_time	= 2[s]
Recvr_gain	= 60
Relaxation_delay	= 2[s]
Repetition_time	= 3.04333312[s]
Temp_get	= 24.1[dC]

Figure S10: 1H NMR Spectrum of Compound 7.

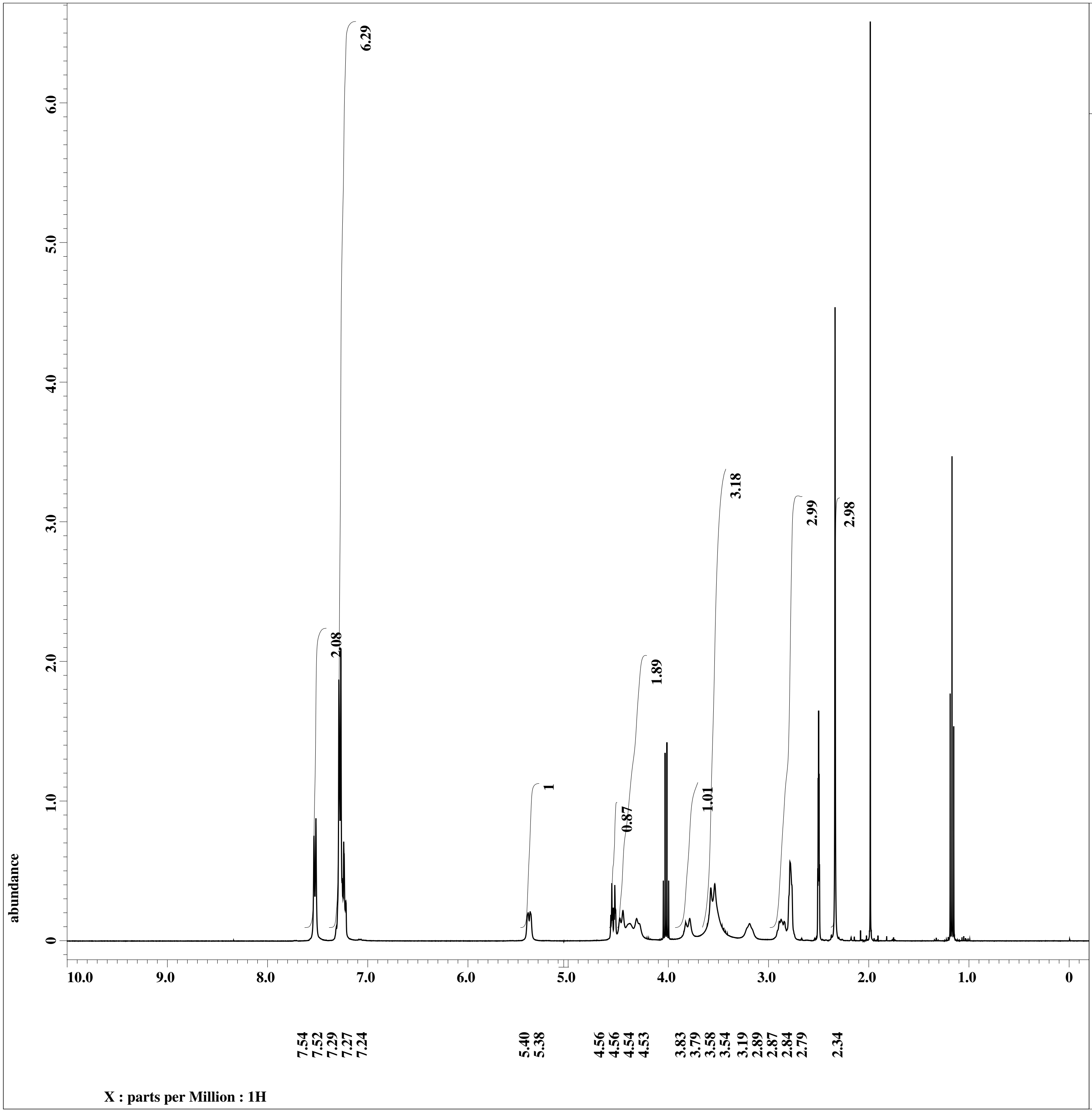


```
Filename          = BDG-II-103 (DMSO)-4.j
Author            = chemlabs
Experiment        = single_pulse.ex2
Sample_id         = 001
Solvent           = DMSO-D6
Creation_time     = 29-JUN-2016 16:32:27
Revision_time     = 19-JUL-2021 11:07:14
Current_time      = 19-JUL-2021 11:08:04

Comment           = single_pulse
Data_format       = 1D COMPLEX
Dim_size          = 13107
Dim_title         = 1H
Dim_units         = [ppm]
Dimensions        = X
Site              = ECS 400
Spectrometer      = JNM-ECS400

Field_strength    = 9.389766[T] (400[MHz]
X_acq_duration    = 2.18365952[s]
X_domain          = 1H
X_freq            = 399.78219838[MHz]
X_offset          = 5[ppm]
X_points          = 16384
X_prescans        = 1
X_resolution      = 0.45794685[Hz]
X_sweep           = 7.5030012[kHz]
Irr_domain        = 1H
Irr_freq          = 399.78219838[MHz]
Irr_offset        = 5[ppm]
Tri_domain        = 1H
Tri_freq          = 399.78219838[MHz]
Tri_offset        = 5[ppm]
Clipped           = FALSE
Mod_return        = 1
Scans             = 8
Total_scans       = 8

X_90_width        = 12.8[us]
X_acq_time        = 2.18365952[s]
X_angle           = 45[deg]
X_atn             = 3[dB]
X_pulse           = 6.4[us]
Irr_mode          = Off
Tri_mode          = Off
Dante_presat      = FALSE
Initial_wait      = 1[s]
Recvr_gain        = 36
Relaxation_delay  = 5[s]
Repetition_time   = 7.18365952[s]
Temp_get          = 22.3[dC]
```

Figure S11: 13C NMR Spectrum of Compound 7.

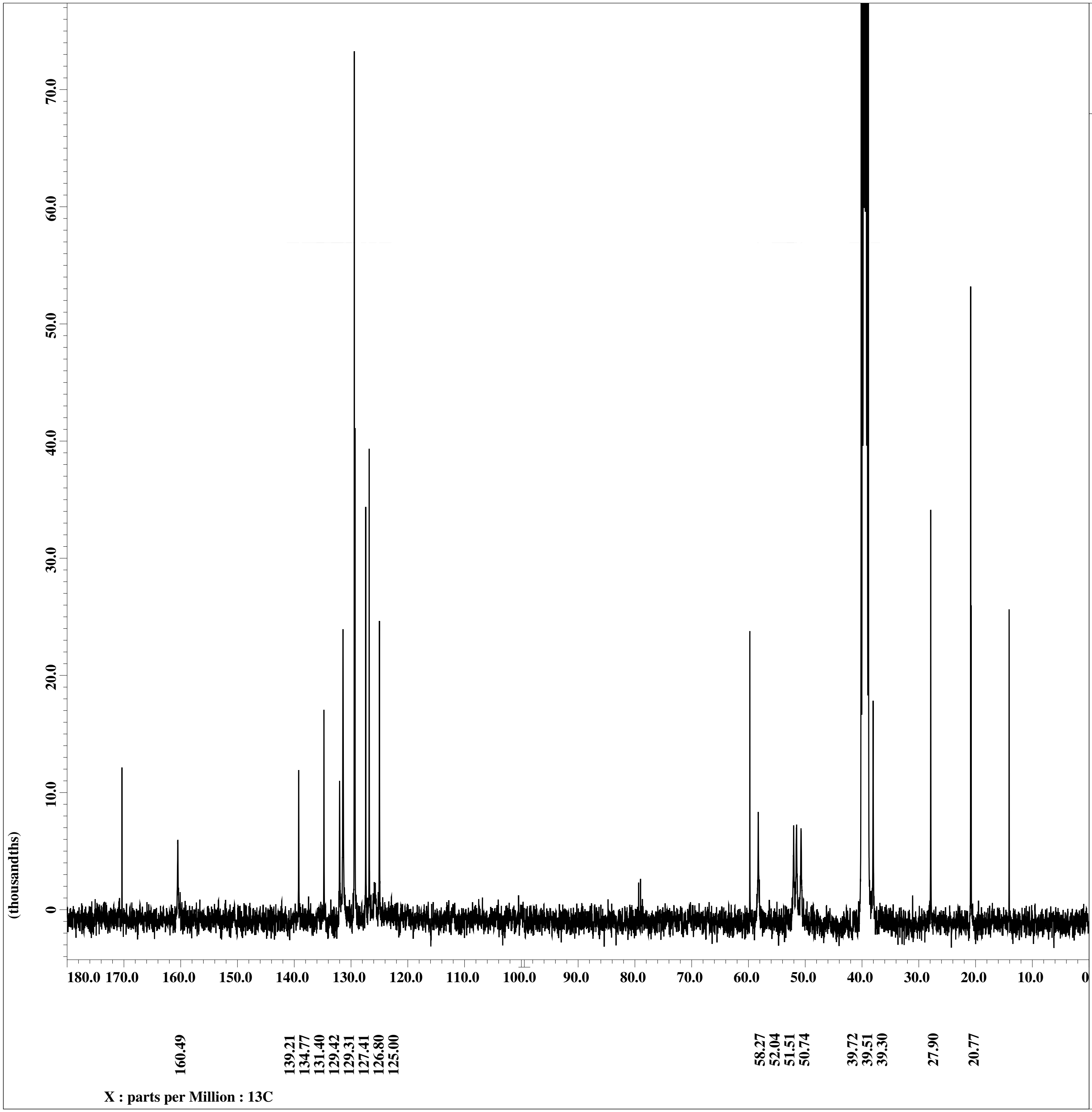


```
Filename          = BDG-II-103 C13 long (
Author            = chemlabs
Experiment        = single_pulse_dec
Sample_id         = 001
Solvent           = DMSO-D6
Creation_time     =  7-JUL-2016 00:51:37
Revision_time     = 21-JUL-2021 13:22:17
Current_time      = 21-JUL-2021 13:23:05

Comment           = single pulse decouple
Data_format       = 1D COMPLEX
Dim_size          = 26214
Dim_title         = 13C
Dim_units         = [ppm]
Dimensions        = X
Site              = ECS 400
Spectrometer      = JNM-ECS400

Field_strength    = 9.389766[T] (400[MHz]
X_acq_duration    = 1.04333312[s]
X_domain          = 13C
X_freq            = 100.52530333[MHz]
X_offset          = 100[ppm]
X_points          = 32768
X_prescans        = 4
X_resolution      = 0.95846665[Hz]
X_sweep           = 31.40703518[kHz]
Irr_domain        = 1H
Irr_freq          = 399.78219838[MHz]
Irr_offset        = 5[ppm]
Clipped           = FALSE
Mod_return        = 1
Scans             = 8192
Total_scans       = 8192

X_90_width        = 9.5[us]
X_acq_time        = 1.04333312[s]
X_angle           = 30[deg]
X_atn             = 6[dB]
X_pulse           = 3.16666667[us]
Irr_atn_dec       = 21[dB]
Irr_atn_noe       = 21[dB]
Irr_noise         = WALTZ
Decoupling        = TRUE
Initial_wait      = 1[s]
Noe               = TRUE
Noe_time          = 2[s]
Recvr_gain        = 60
Relaxation_delay  = 2[s]
Repetition_time   = 3.04333312[s]
Temp_get          = 23.6[dC]
```

Figure S12: 1H NMR Spectrum of Compound 8.

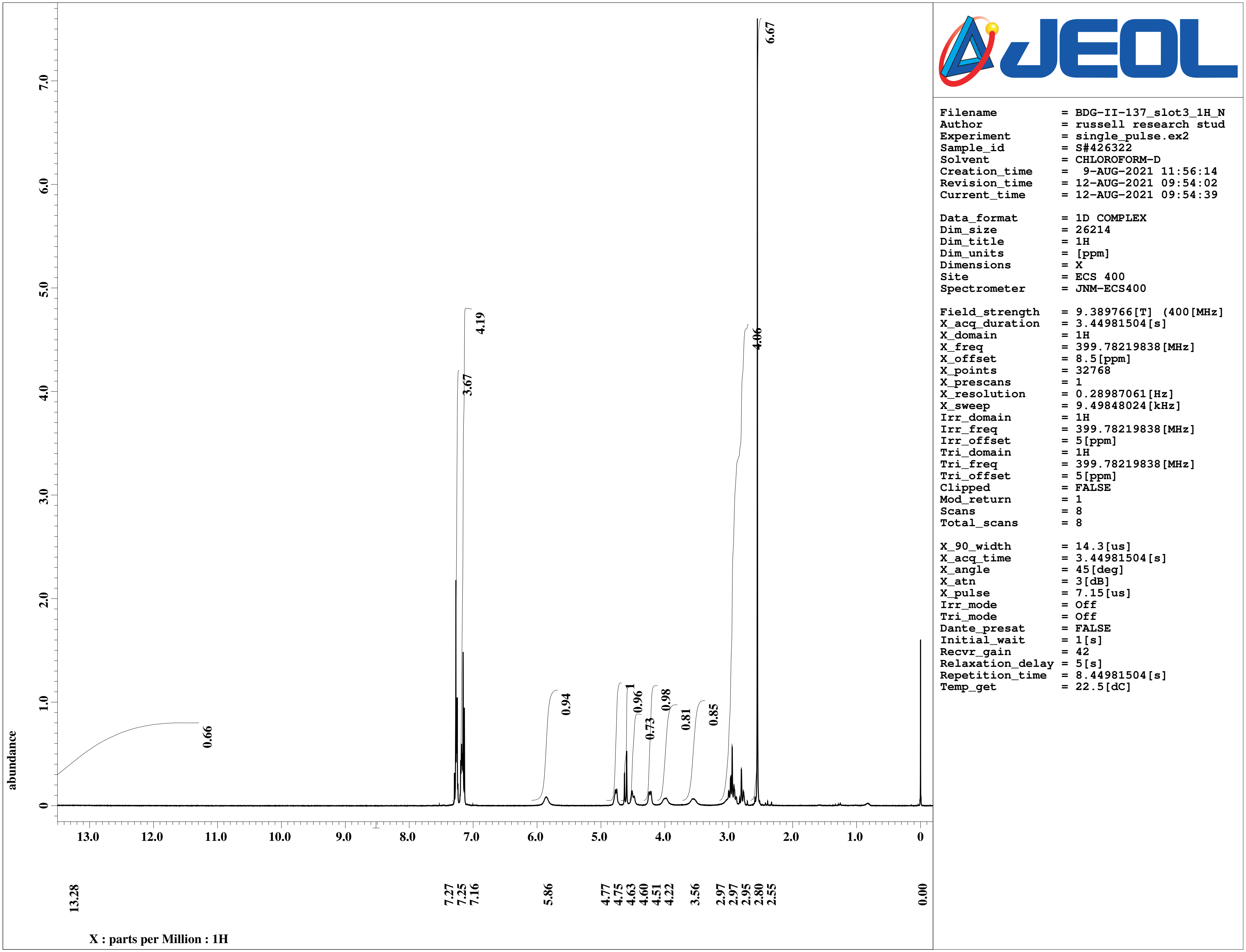

Figure S13: 13C NMR Spectrum of Compound 8.

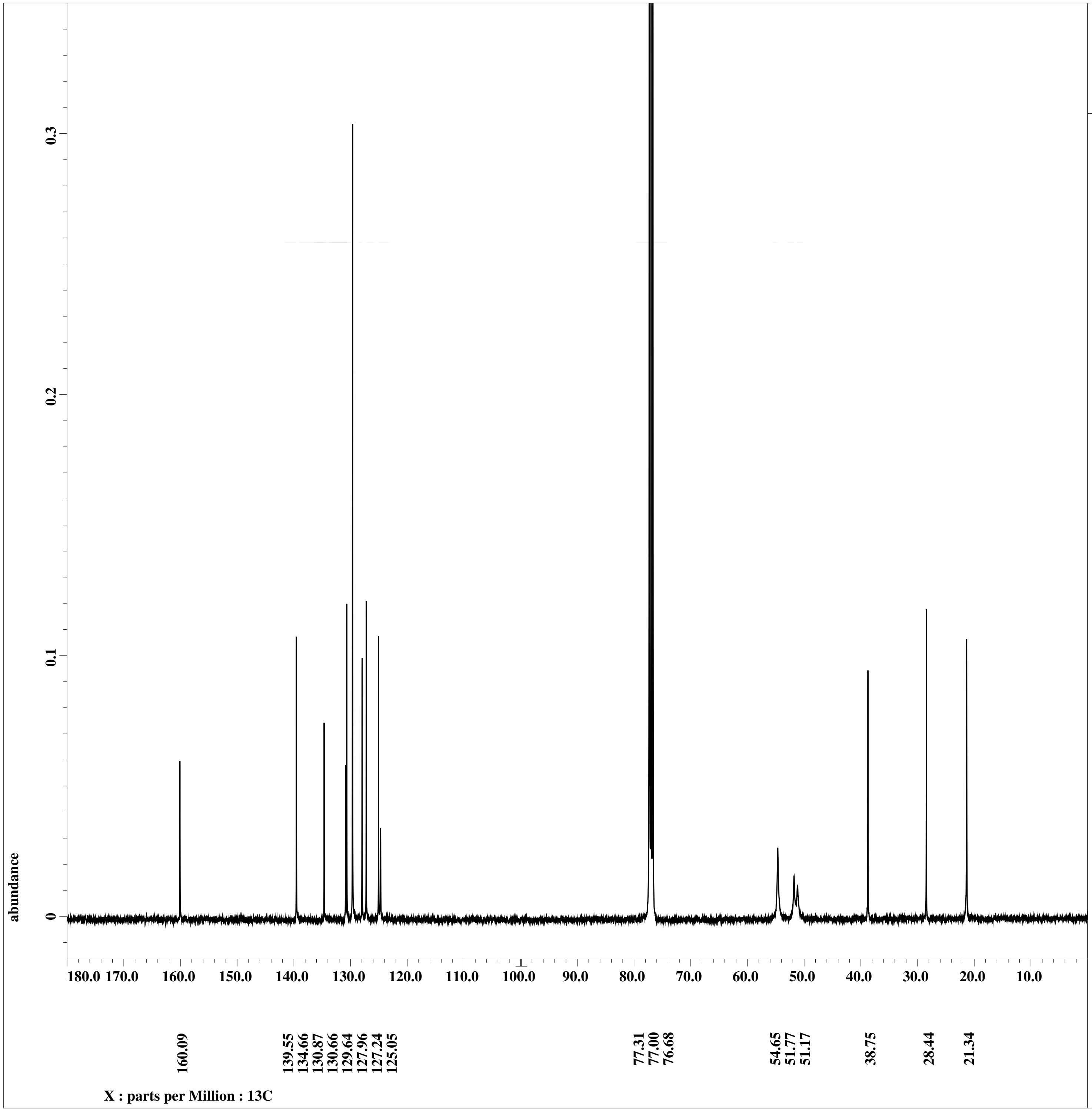

Filename	= BDG-II-137_slot3_13C_
Author	= russell research stud
Experiment	= single_pulse_dec
Sample_id	= S#78660
Solvent	= CHLOROFORM-D
Creation_time	= 10-AUG-2021 16:05:52
Revision_time	= 12-AUG-2021 09:57:47
Current_time	= 12-AUG-2021 10:01:50
Data_format	= 1D COMPLEX
Dim_size	= 26214
Dim_title	= 13C
Dim_units	= [ppm]
Dimensions	= X
Site	= ECS 400
Spectrometer	= JNM-ECS400
Field_strength	= 9.389766[T] (400[MHz]
X_acq_duration	= 1.04333312[s]
X_domain	= 13C
X_freq	= 100.52530333[MHz]
X_offset	= 100[ppm]
X_points	= 32768
X_prescans	= 4
X_resolution	= 0.95846665[Hz]
X_sweep	= 31.40703518[kHz]
Irr_domain	= 1H
Irr_freq	= 399.78219838[MHz]
Irr_offset	= 5[ppm]
Clipped	= FALSE
Mod_return	= 1
Scans	= 16384
Total_scans	= 16384
X_90_width	= 9.82623[us]
X_acq_time	= 1.04333312[s]
X_angle	= 30[deg]
X_atn	= 5.8[dB]
X_pulse	= 3.27541[us]
Irr_atn_dec	= 21[dB]
Irr_atn_noe	= 21[dB]
Irr_noise	= WALTZ
Decoupling	= TRUE
Initial_wait	= 1[s]
Noe	= TRUE
Noe_time	= 2[s]
Recvr_gain	= 60
Relaxation_delay	= 2[s]
Repetition_time	= 3.04333312[s]
Temp_get	= 23.6[dC]

Figure S14: 1H NMR Spectrum of Compound 9.

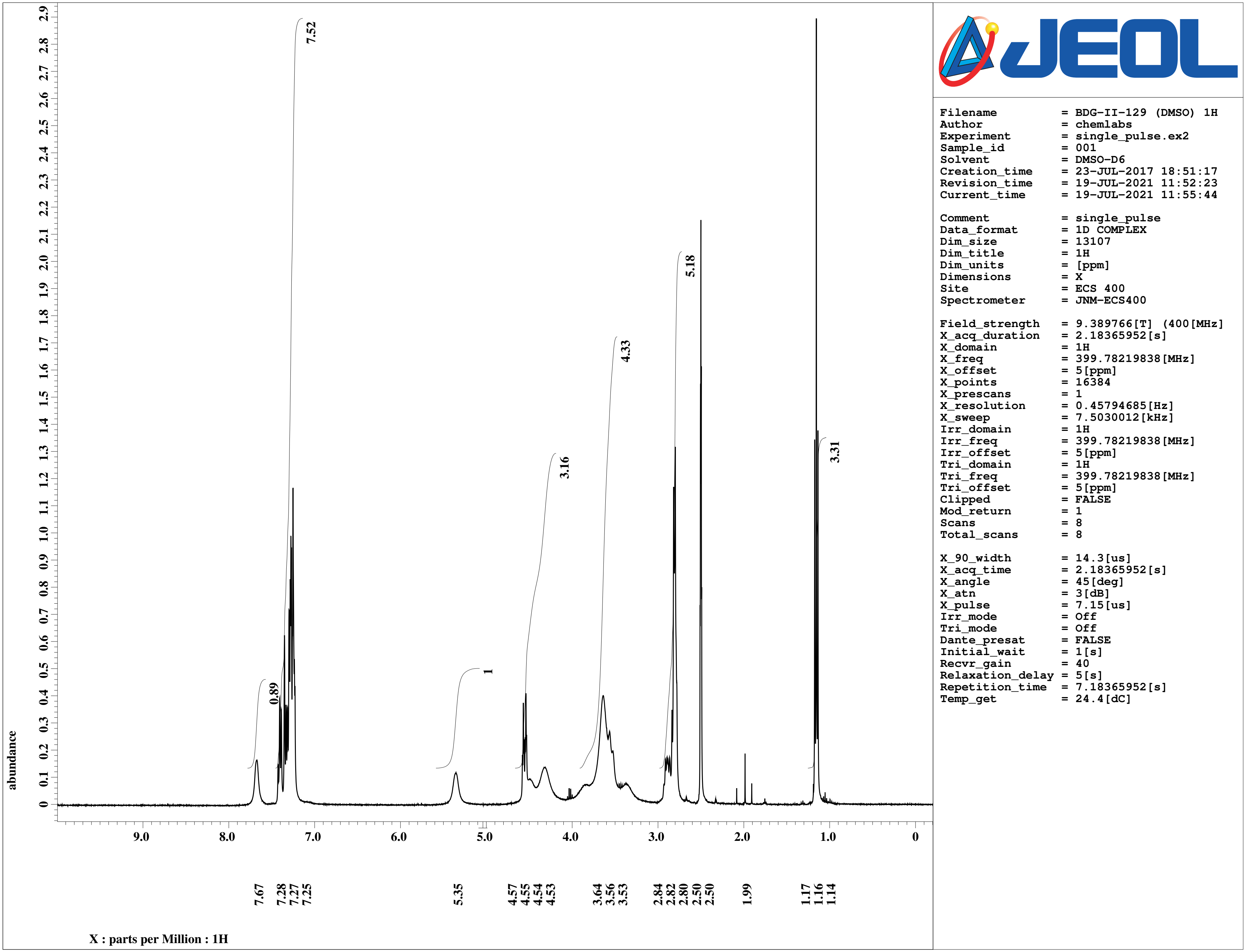

Figure S15: 13C NMR Spectrum of Compound 9.

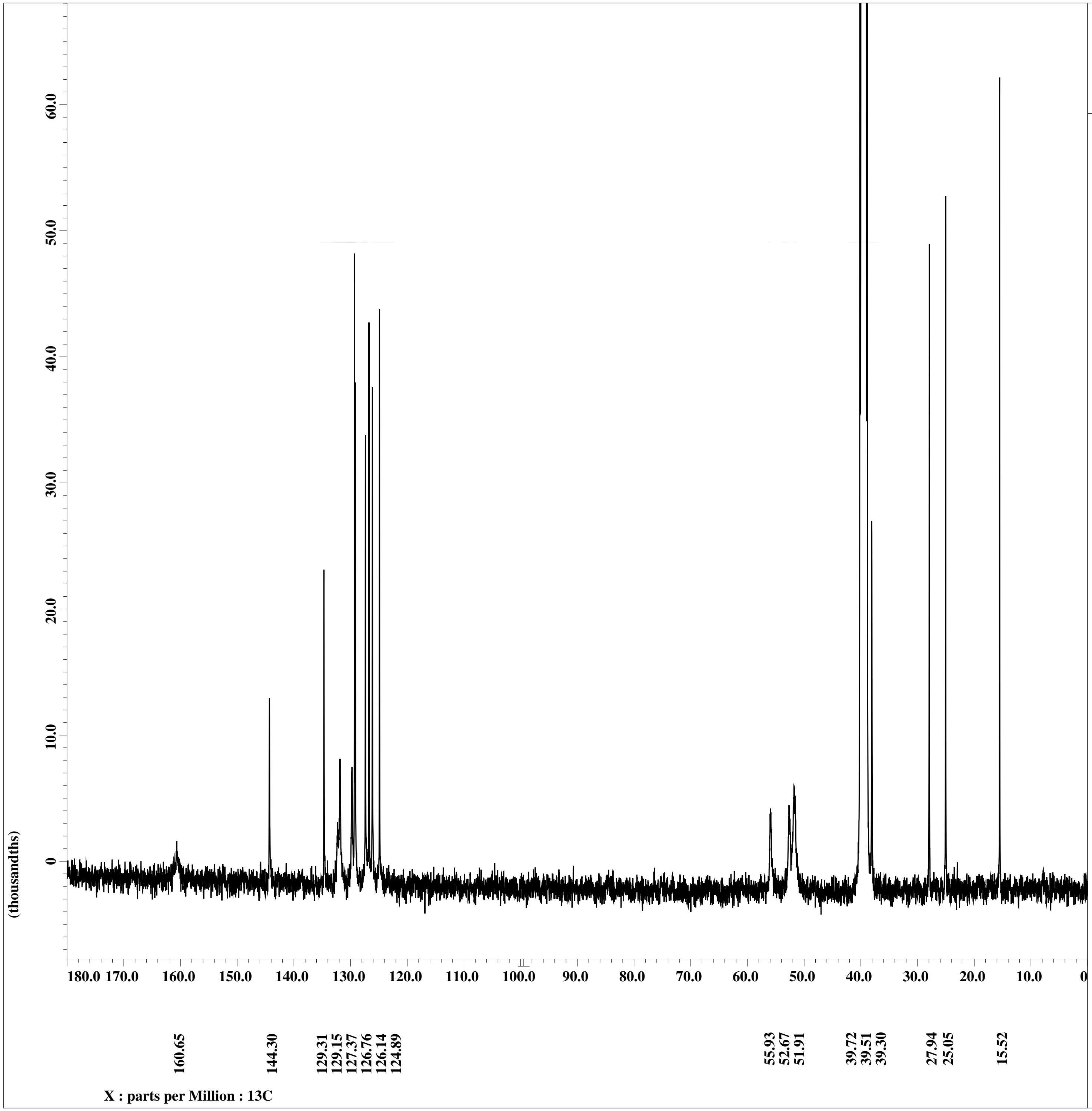

JEOL

```
Filename          = BDG-II-129 (DMSO) 13C
Author            = chemlabs
Experiment        = single_pulse_dec
Sample_id         = 001
Solvent           = DMSO-D6
Creation_time     = 24-JUL-2017 07:09:58
Revision_time     = 22-JUL-2021 11:29:47
Current_time      = 22-JUL-2021 11:29:51

Comment           = single pulse decouple
Data_format       = 1D COMPLEX
Dim_size          = 26214
Dim_title         = 13C
Dim_units         = [ppm]
Dimensions        = X
Site              = ECS 400
Spectrometer      = JNM-ECS400

Field_strength    = 9.389766[T] (400[MHz]
X_acq_duration    = 1.04333312[s]
X_domain          = 13C
X_freq            = 100.52530333[MHz]
X_offset          = 100[ppm]
X_points          = 32768
X_prescans        = 4
X_resolution      = 0.95846665[Hz]
X_sweep           = 31.40703518[kHz]
Irr_domain        = 1H
Irr_freq          = 399.78219838[MHz]
Irr_offset        = 5[ppm]
Clipped           = FALSE
Mod_return        = 1
Scans             = 14552
Total_scans       = 14552

X_90_width        = 9.82623[us]
X_acq_time        = 1.04333312[s]
X_angle           = 30[deg]
X_atn             = 5.8[dB]
X_pulse           = 3.27541[us]
Irr_atn_dec       = 21[dB]
Irr_atn_noe       = 21[dB]
Irr_noise         = WALTZ
Decoupling        = TRUE
Initial_wait      = 1[s]
Noe               = TRUE
Noe_time          = 2[s]
Recvr_gain        = 60
Relaxation_delay  = 2[s]
Repetition_time   = 3.04333312[s]
Temp_get          = 24.2[dC]
```

Figure S16: 1H NMR Spectrum of Compound 10.

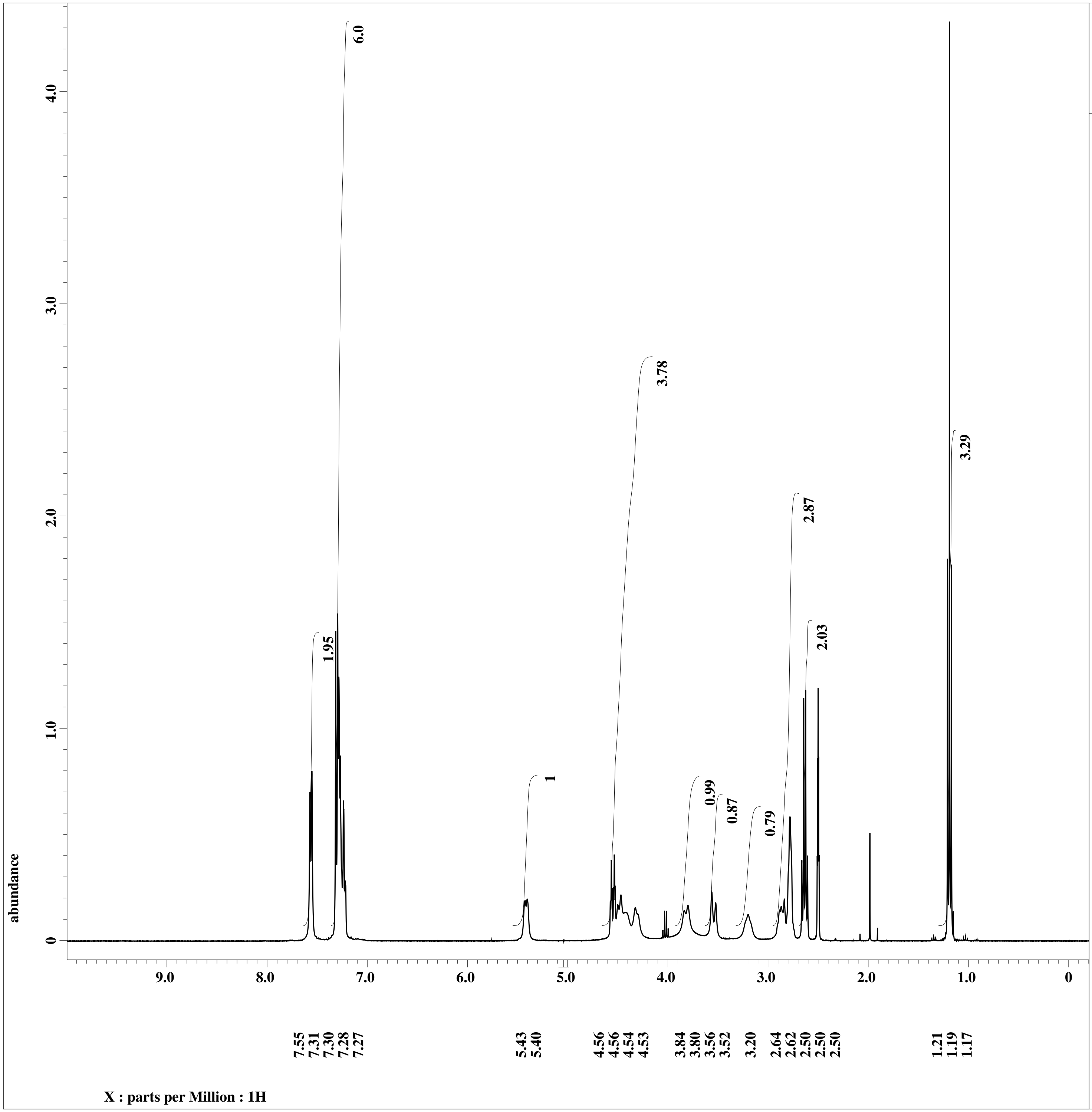

JEOL

```
Filename           = BDG-II-119(DMSO) 1H N
Author             = chemlabs
Experiment         = single_pulse.ex2
Sample_id          = 001
Solvent            = DMSO-D6
Creation_time      = 24-JUL-2017 08:15:14
Revision_time      = 19-JUL-2021 13:09:10
Current_time       = 19-JUL-2021 13:11:23

Comment            = single_pulse
Data_format        = 1D COMPLEX
Dim_size           = 13107
Dim_title          = 1H
Dim_units          = [ppm]
Dimensions         = X
Site               = ECS 400
Spectrometer       = JNM-ECS400

Field_strength     = 9.389766[T] (400[MHz]
X_acq_duration     = 2.18365952[s]
X_domain           = 1H
X_freq             = 399.78219838[MHz]
X_offset           = 5[ppm]
X_points           = 16384
X_prescans         = 1
X_resolution       = 0.45794685[Hz]
X_sweep            = 7.5030012[kHz]
Irr_domain         = 1H
Irr_freq           = 399.78219838[MHz]
Irr_offset         = 5[ppm]
Tri_domain         = 1H
Tri_freq           = 399.78219838[MHz]
Tri_offset         = 5[ppm]
Clipped            = FALSE
Mod_return         = 1
Scans              = 8
Total_scans        = 8

X_90_width         = 14.3[us]
X_acq_time         = 2.18365952[s]
X_angle            = 45[deg]
X_atn              = 3[dB]
X_pulse            = 7.15[us]
Irr_mode           = Off
Tri_mode           = Off
Dante_presat       = FALSE
Initial_wait       = 1[s]
Recvr_gain         = 34
Relaxation_delay   = 5[s]
Repetition_time    = 7.18365952[s]
Temp_get           = 23.8[dC]
```

Figure S17: 13C NMR Spectrum of Compound 10.

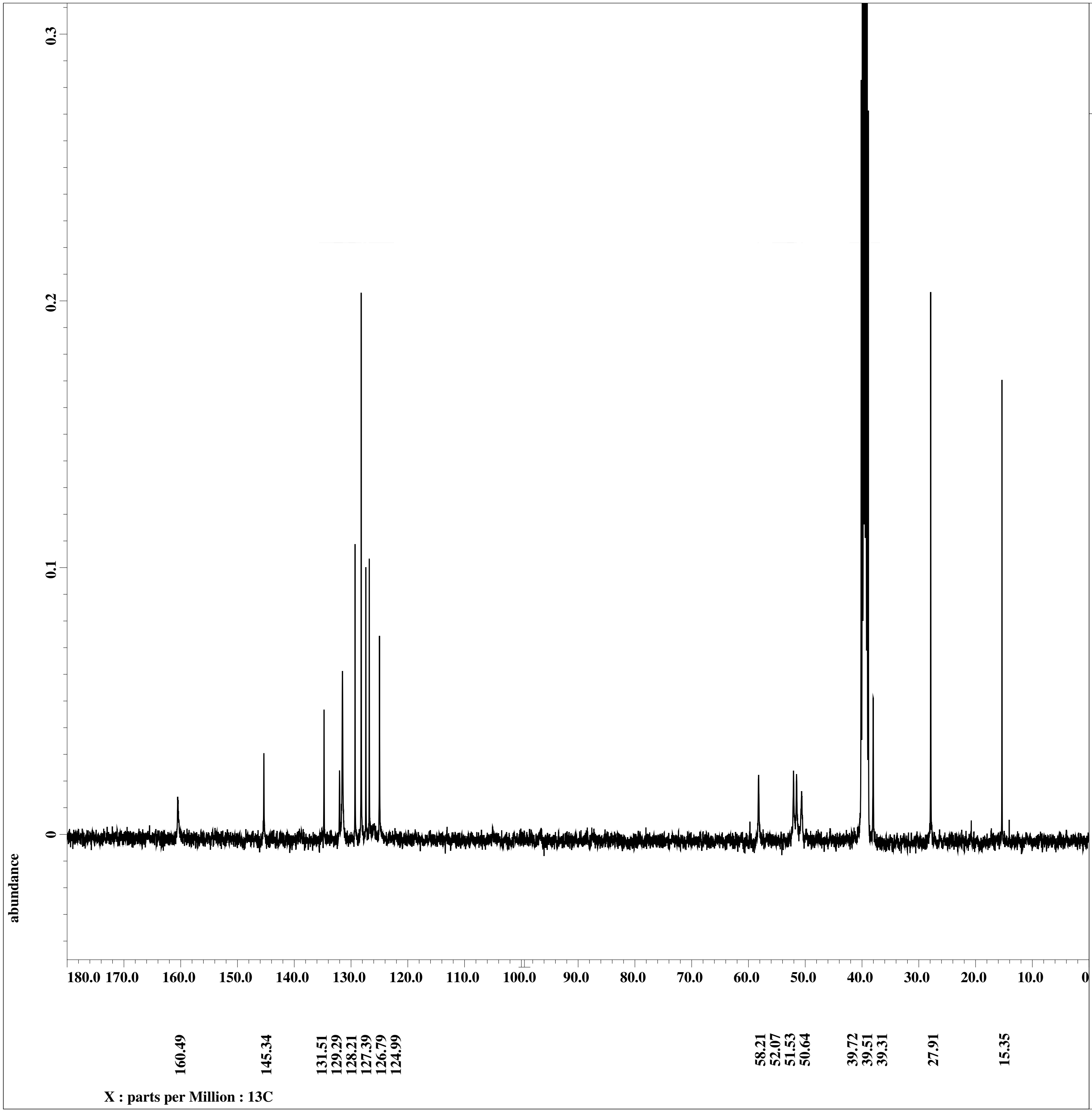


```
Filename          = BDG-II-119 (DMSO) 13C
Author            = chemlabs
Experiment        = single_pulse_dec
Sample_id         = 001
Solvent           = DMSO-D6
Creation_time     = 24-JUL-2017 09:55:49
Revision_time     = 19-JUL-2021 13:19:03
Current_time      = 19-JUL-2021 13:22:51

Comment           = single pulse decouple
Data_format       = 1D COMPLEX
Dim_size          = 26214
Dim_title         = 13C
Dim_units         = [ppm]
Dimensions        = X
Site              = ECS 400
Spectrometer      = JNM-ECS400

Field_strength    = 9.389766[T] (400[MHz]
X_acq_duration    = 1.04333312[s]
X_domain          = 13C
X_freq            = 100.52530333[MHz]
X_offset          = 100[ppm]
X_points          = 32768
X_prescans        = 4
X_resolution      = 0.95846665[Hz]
X_sweep           = 31.40703518[kHz]
Irr_domain        = 1H
Irr_freq          = 399.78219838[MHz]
Irr_offset        = 5[ppm]
Clipped           = FALSE
Mod_return        = 1
Scans             = 1965
Total_scans       = 1965

X_90_width        = 9.82623[us]
X_acq_time        = 1.04333312[s]
X_angle           = 30[deg]
X_atn             = 5.8[dB]
X_pulse           = 3.27541[us]
Irr_atn_dec       = 21[dB]
Irr_atn_noe       = 21[dB]
Irr_noise         = WALTZ
Decoupling        = TRUE
Initial_wait      = 1[s]
Noe               = TRUE
Noe_time          = 2[s]
Recvr_gain        = 60
Relaxation_delay  = 2[s]
Repetition_time   = 3.04333312[s]
Temp_get          = 24.2[dC]
```

Figure S18: 1H NMR Spectrum of Compound 11.

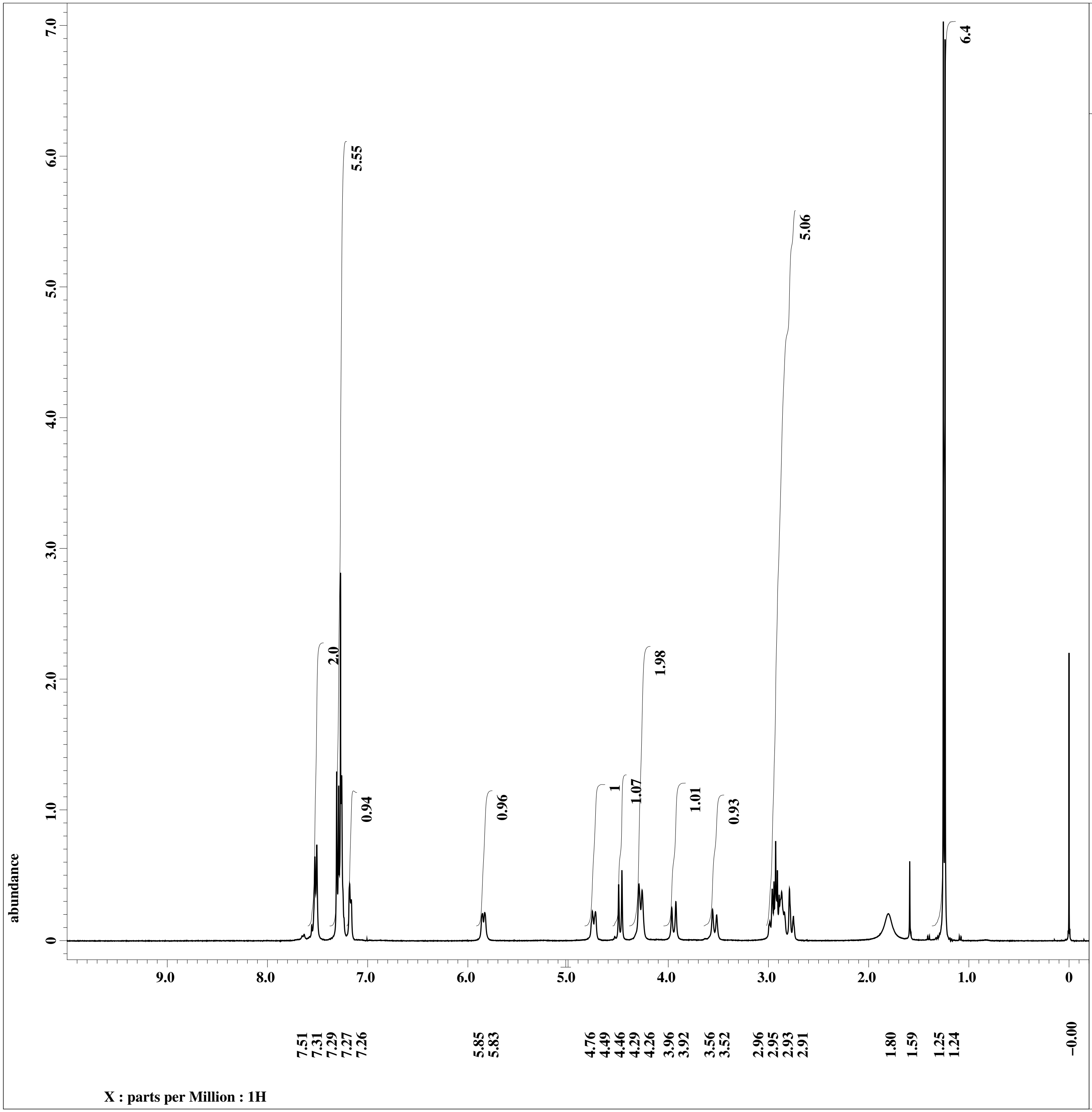


```
Filename          = SMZ-1-107 1H NMR-4.jd
Author            = chemlabs
Experiment        = single_pulse.ex2
Sample_id         = 001
Solvent           = CHLOROFORM-D
Creation_time     = 31-MAY-2019 13:04:30
Revision_time     = 19-JUL-2021 13:44:07
Current_time      = 19-JUL-2021 13:51:47

Comment           = single_pulse
Data_format       = 1D COMPLEX
Dim_size          = 13107
Dim_title         = 1H
Dim_units         = [ppm]
Dimensions        = X
Site              = ECS 400
Spectrometer      = JNM-ECS400

Field_strength    = 9.389766[T] (400[MHz]
X_acq_duration    = 2.18365952[s]
X_domain          = 1H
X_freq            = 399.78219838[MHz]
X_offset          = 5[ppm]
X_points          = 16384
X_prescans        = 1
X_resolution      = 0.45794685[Hz]
X_sweep           = 7.5030012[kHz]
Irr_domain        = 1H
Irr_freq          = 399.78219838[MHz]
Irr_offset        = 5[ppm]
Tri_domain        = 1H
Tri_freq          = 399.78219838[MHz]
Tri_offset        = 5[ppm]
Clipped           = FALSE
Mod_return        = 1
Scans             = 8
Total_scans       = 8

X_90_width        = 14.3[us]
X_acq_time        = 2.18365952[s]
X_angle           = 45[deg]
X_atn             = 3[dB]
X_pulse           = 7.15[us]
Irr_mode          = Off
Tri_mode          = Off
Dante_presat      = FALSE
Initial_wait      = 1[s]
Recvr_gain        = 42
Relaxation_delay  = 5[s]
Repetition_time   = 7.18365952[s]
Temp_get          = 21.3[dC]
```

Figure S19: 13C NMR Spectrum of Compound 11.

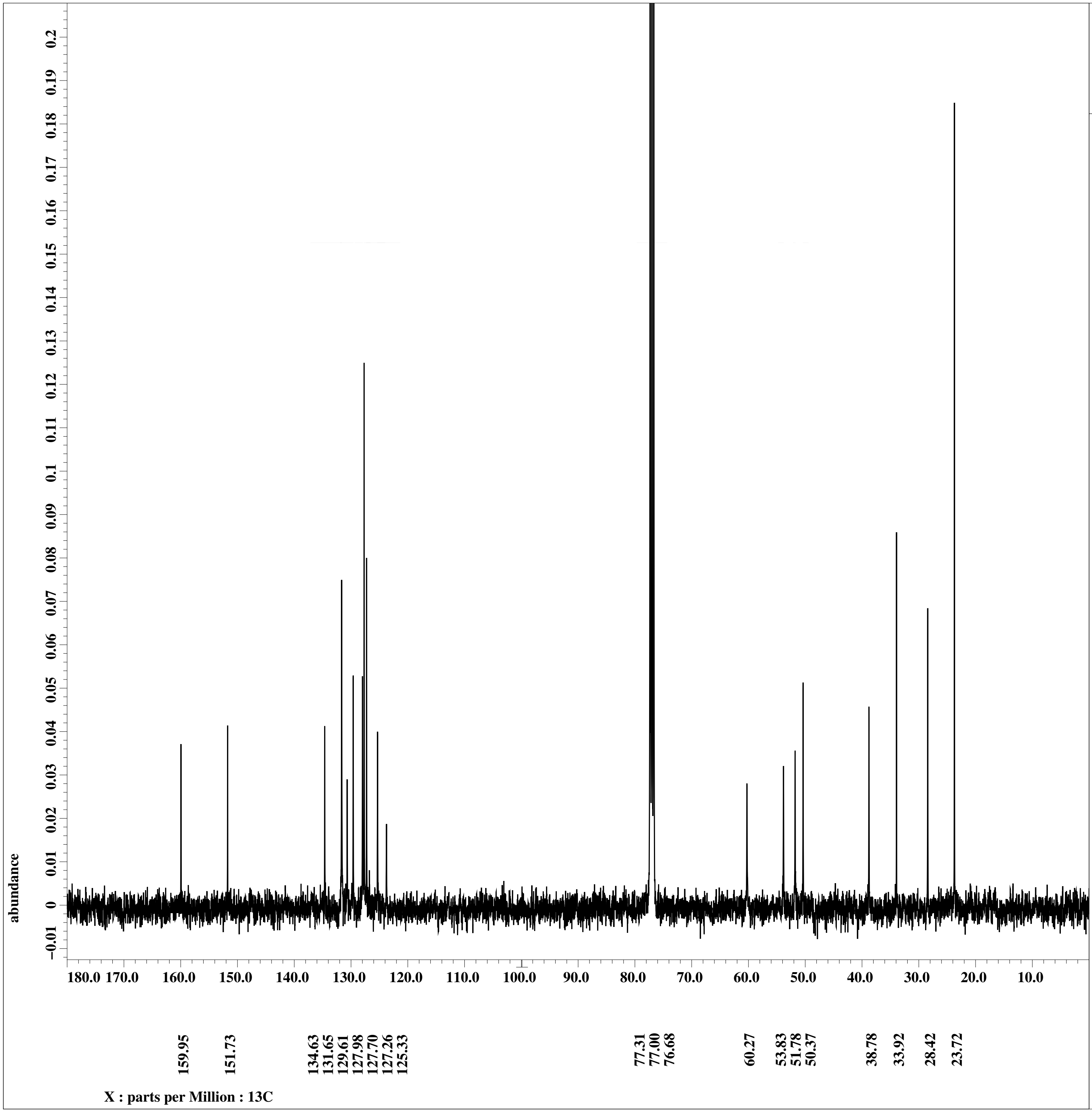

Filename	= SMZ-1-107 13C NMR-4.j
Author	= chemlabs
Experiment	= single_pulse_dec
Sample_id	= 001
Solvent	= CHLOROFORM-D
Creation_time	= 31-MAY-2019 13:58:20
Revision_time	= 19-JUL-2021 13:48:23
Current_time	= 19-JUL-2021 13:51:13
Comment	= single pulse decouple
Data_format	= 1D COMPLEX
Dim_size	= 26214
Dim_title	= 13C
Dim_units	= [ppm]
Dimensions	= X
Site	= ECS 400
Spectrometer	= JNM-ECS400
Field_strength	= 9.389766[T] (400[MHz]
X_acq_duration	= 1.04333312[s]
X_domain	= 13C
X_freq	= 100.52530333[MHz]
X_offset	= 100[ppm]
X_points	= 32768
X_prescans	= 4
X_resolution	= 0.95846665[Hz]
X_sweep	= 31.40703518[kHz]
Irr_domain	= 1H
Irr_freq	= 399.78219838[MHz]
Irr_offset	= 5[ppm]
Clipped	= FALSE
Mod_return	= 1
Scans	= 1044
Total_scans	= 1044
X_90_width	= 9.82623[us]
X_acq_time	= 1.04333312[s]
X_angle	= 30[deg]
X_atn	= 5.8[dB]
X_pulse	= 3.27541[us]
Irr_atn_dec	= 21[dB]
Irr_atn_noe	= 21[dB]
Irr_noise	= WALTZ
Decoupling	= TRUE
Initial_wait	= 1[s]
Noe	= TRUE
Noe_time	= 2[s]
Recvr_gain	= 60
Relaxation_delay	= 2[s]
Repetition_time	= 3.04333312[s]
Temp_get	= 21.6[dC]

Figure S20: 1H NMR Spectrum of Compound 12.

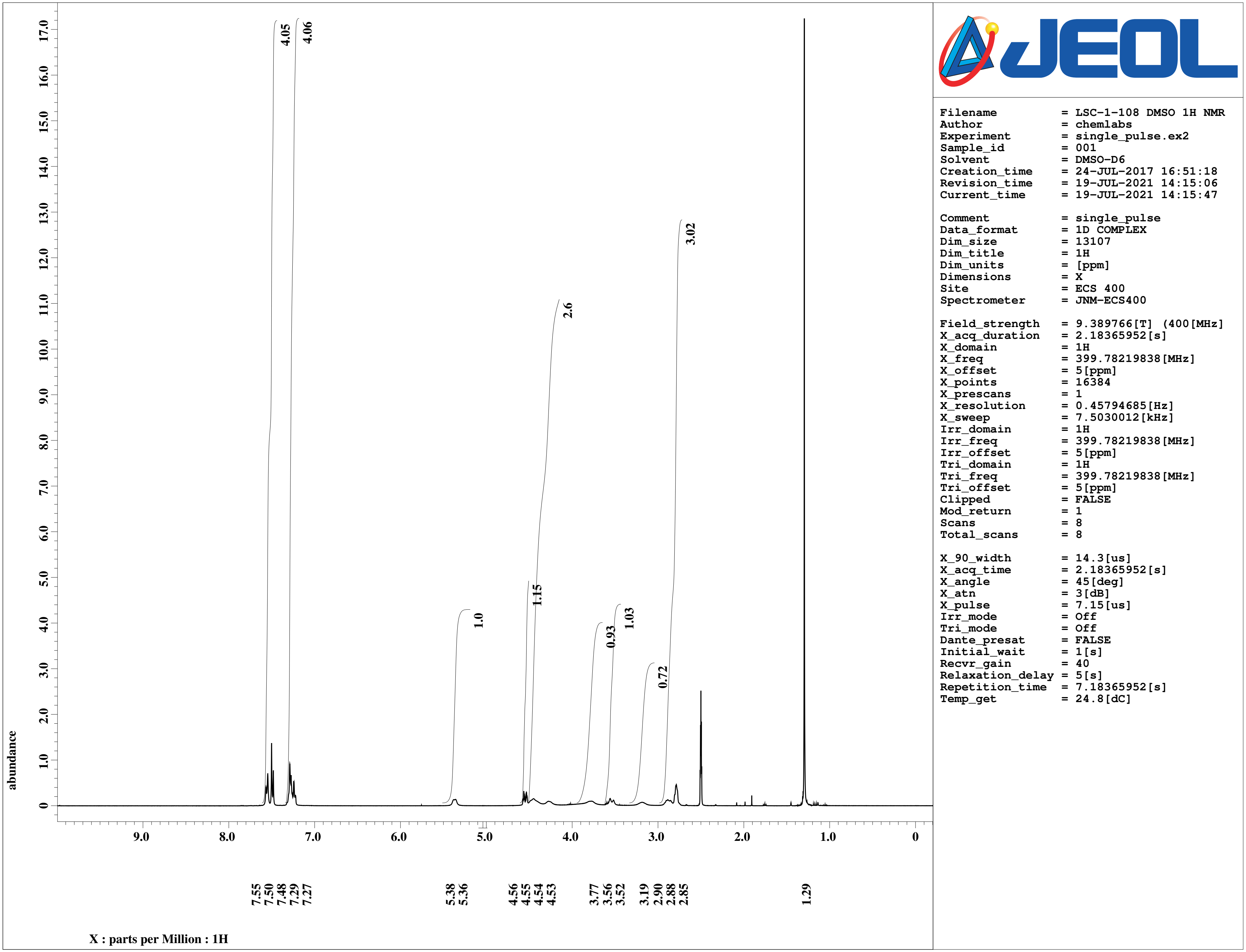

Figure S21: 13C NMR Spectrum of Compound 12.

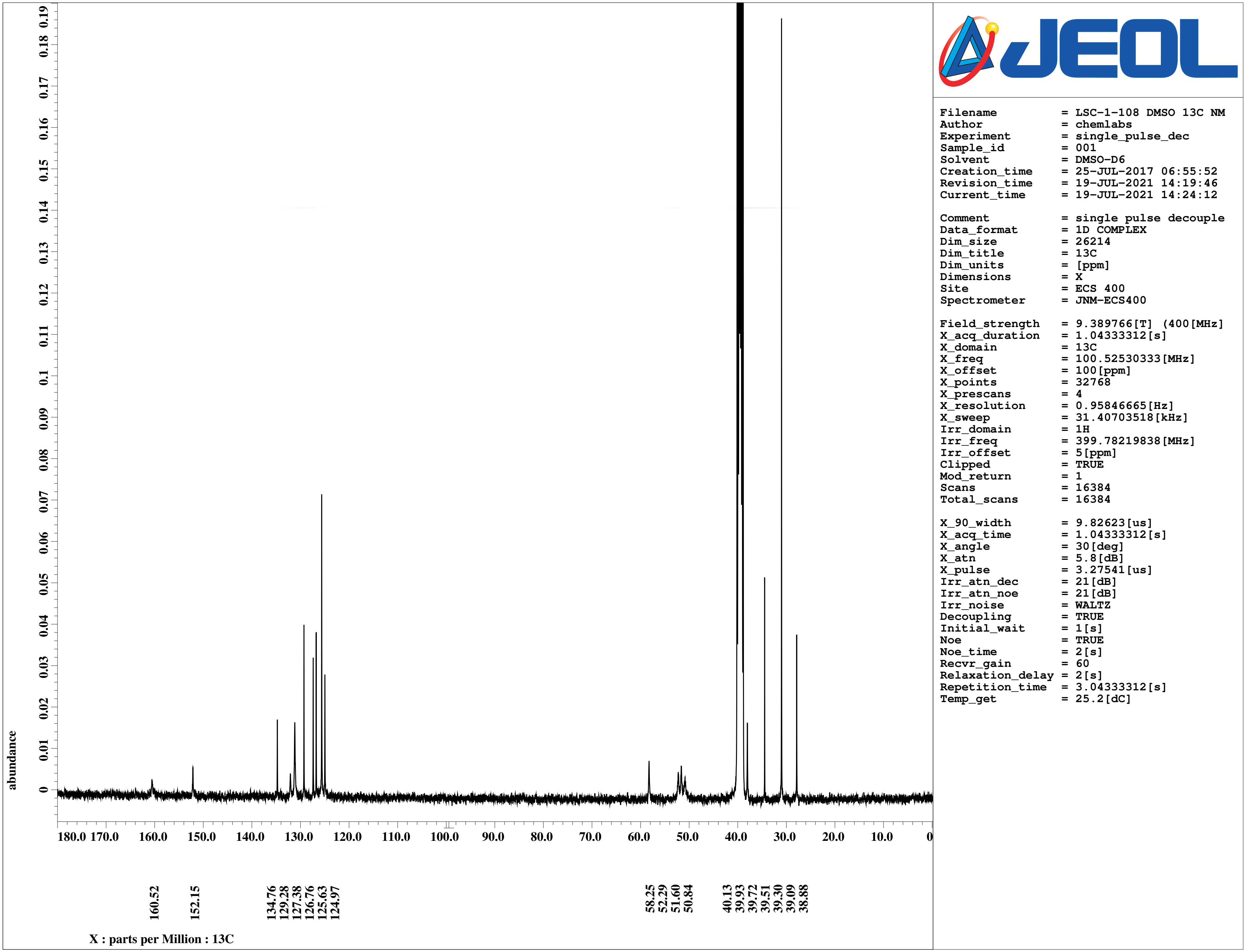

Figure S22: 1H NMR Spectrum of Compound 13.

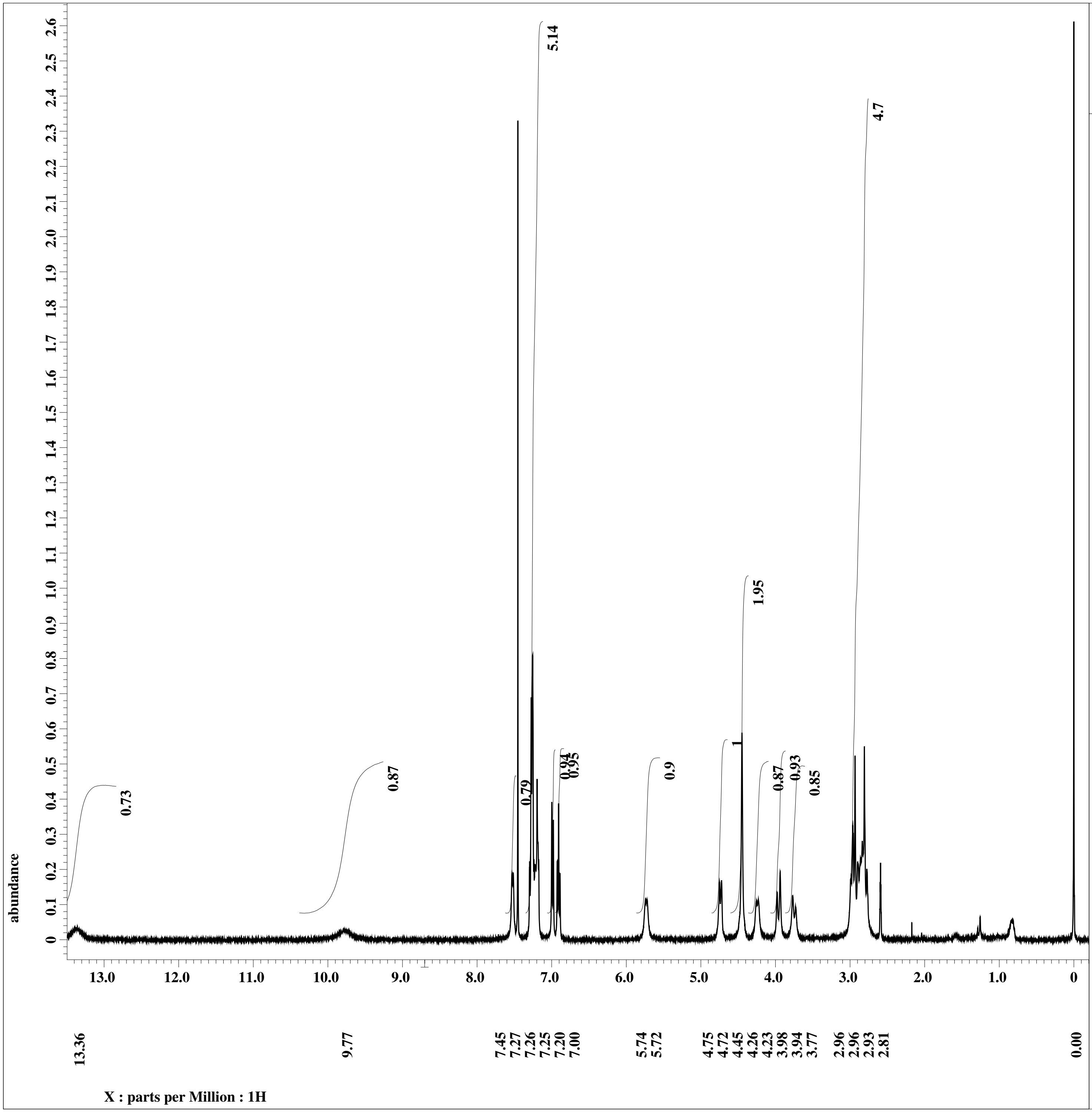


```
Filename          = BDG-II-085_slot4_1H_N
Author            = russell research stud
Experiment        = single_pulse.ex2
Sample_id         = S#429882
Solvent           = CHLOROFORM-D
Creation_time     =  9-AUG-2021 12:01:36
Revision_time     = 12-AUG-2021 10:13:34
Current_time      = 12-AUG-2021 10:13:52

Data_format       = 1D COMPLEX
Dim_size          = 26214
Dim_title         = 1H
Dim_units         = [ppm]
Dimensions        = X
Site              = ECS 400
Spectrometer      = JNM-ECS400

Field_strength    = 9.389766[T] (400[MHz]
X_acq_duration    = 3.44981504[s]
X_domain          = 1H
X_freq            = 399.78219838[MHz]
X_offset          = 8.5[ppm]
X_points          = 32768
X_prescans        = 1
X_resolution      = 0.28987061[Hz]
X_sweep           = 9.49848024[kHz]
Irr_domain        = 1H
Irr_freq          = 399.78219838[MHz]
Irr_offset        = 5[ppm]
Tri_domain        = 1H
Tri_freq          = 399.78219838[MHz]
Tri_offset        = 5[ppm]
Clipped           = FALSE
Mod_return        = 1
Scans             = 8
Total_scans       = 8

X_90_width        = 14.3[us]
X_acq_time        = 3.44981504[s]
X_angle           = 45[deg]
X_atn             = 3[dB]
X_pulse           = 7.15[us]
Irr_mode          = Off
Tri_mode          = Off
Dante_presat      = FALSE
Initial_wait      = 1[s]
Recvr_gain        = 50
Relaxation_delay  = 5[s]
Repetition_time   = 8.44981504[s]
Temp_get          = 22.6[dC]
```

Figure S23: 13C NMR Spectrum of Compound 13.

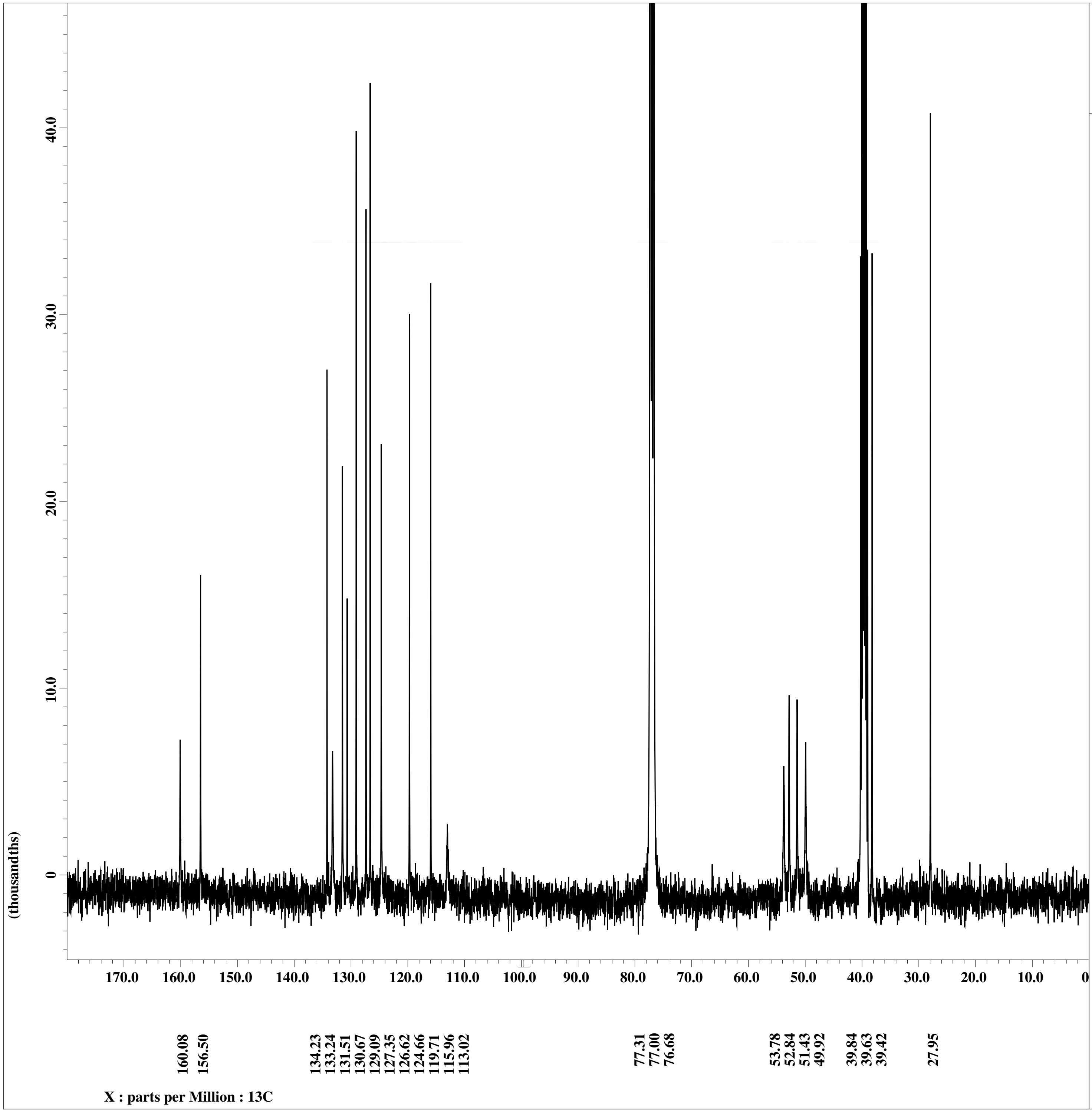


```
Filename           = BDG-II-085_slot4_13C_
Author             = russell research stud
Experiment         = single_pulse_dec
Sample_id          = S#580766
Solvent            = CHLOROFORM-D
Creation_time      = 11-AUG-2021 06:02:45
Revision_time      = 12-AUG-2021 10:14:57
Current_time       = 12-AUG-2021 10:20:44

Data_format        = 1D COMPLEX
Dim_size           = 26214
Dim_title          = 13C
Dim_units          = [ppm]
Dimensions         = X
Site               = ECS 400
Spectrometer       = JNM-ECS400

Field_strength     = 9.389766[T] (400[MHz]
X_acq_duration     = 1.04333312[s]
X_domain           = 13C
X_freq             = 100.52530333[MHz]
X_offset           = 100[ppm]
X_points           = 32768
X_prescans         = 4
X_resolution       = 0.95846665[Hz]
X_sweep            = 31.40703518[kHz]
Irr_domain         = 1H
Irr_freq           = 399.78219838[MHz]
Irr_offset         = 5[ppm]
Clipped            = FALSE
Mod_return         = 1
Scans              = 16384
Total_scans        = 16384

X_90_width         = 9.82623[us]
X_acq_time         = 1.04333312[s]
X_angle            = 30[deg]
X_atn              = 5.8[dB]
X_pulse            = 3.27541[us]
Irr_atn_dec        = 21[dB]
Irr_atn_noe        = 21[dB]
Irr_noise          = WALTZ
Decoupling         = TRUE
Initial_wait       = 1[s]
Noe                = TRUE
Noe_time           = 2[s]
Recvr_gain         = 60
Relaxation_delay   = 2[s]
Repetition_time    = 3.04333312[s]
Temp_get           = 23.1[dC]
```

Figure S24: 1H NMR Spectrum of Compound 14.

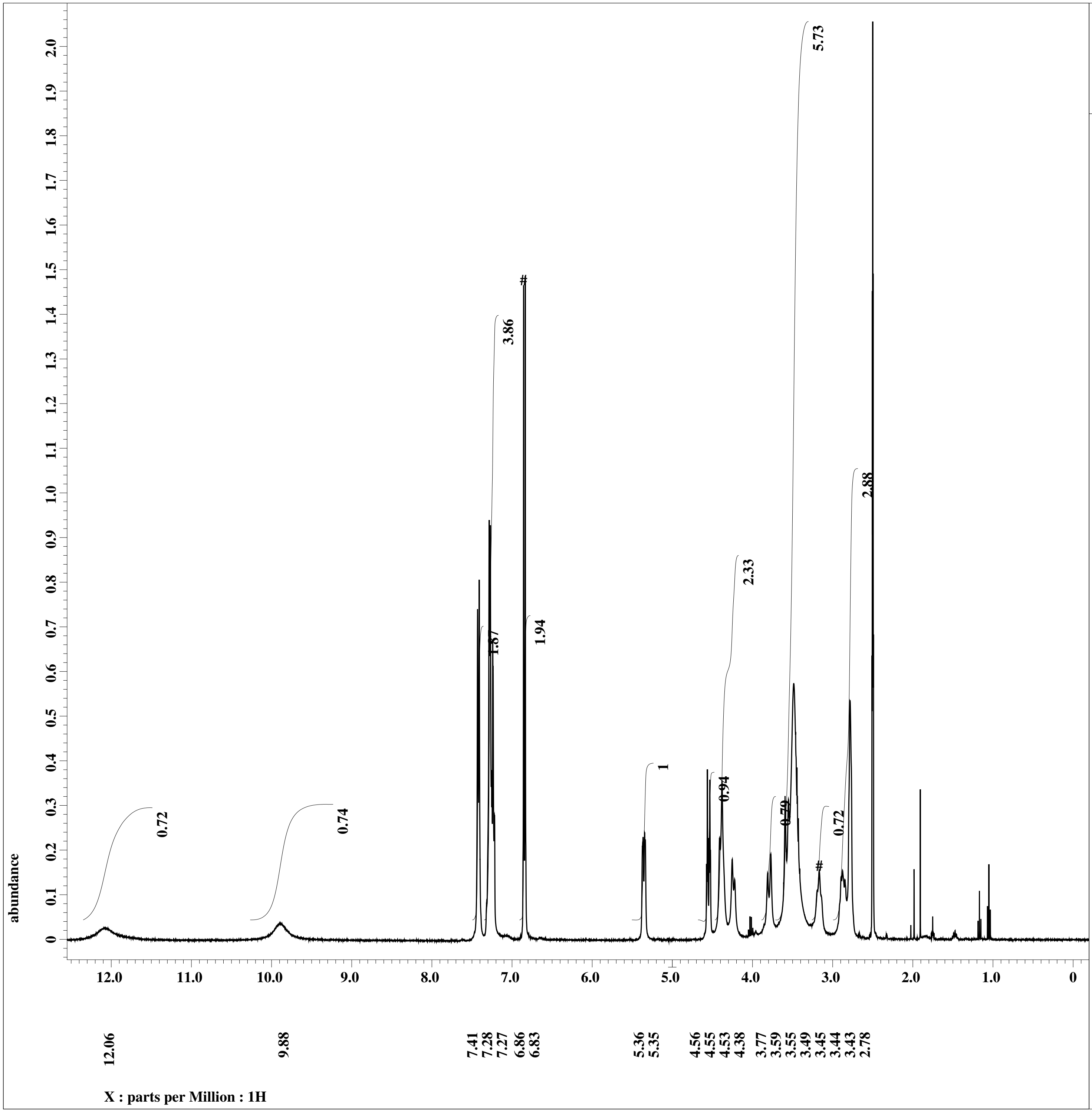


```
Filename          = BDG-II-083 DMSO-4.jdf
Author            = chemlabs
Experiment        = single_pulse.ex2
Sample_id         = 001
Solvent           = DMSO-D6
Creation_time     = 20-JUN-2016 09:50:39
Revision_time     = 16-JUL-2021 12:01:51
Current_time      = 16-JUL-2021 12:02:20

Comment           = single_pulse
Data_format       = 1D COMPLEX
Dim_size          = 13107
Dim_title         = 1H
Dim_units         = [ppm]
Dimensions        = X
Site              = ECS 400
Spectrometer      = JNM-ECS400

Field_strength    = 9.389766[T] (400[MHz]
X_acq_duration    = 2.18365952[s]
X_domain          = 1H
X_freq            = 399.78219838[MHz]
X_offset          = 5[ppm]
X_points          = 16384
X_prescans        = 1
X_resolution      = 0.45794685[Hz]
X_sweep           = 7.5030012[kHz]
Irr_domain        = 1H
Irr_freq          = 399.78219838[MHz]
Irr_offset        = 5[ppm]
Tri_domain        = 1H
Tri_freq          = 399.78219838[MHz]
Tri_offset        = 5[ppm]
Clipped           = FALSE
Mod_return        = 1
Scans             = 8
Total_scans       = 8

X_90_width        = 12.8[us]
X_acq_time        = 2.18365952[s]
X_angle           = 45[deg]
X_atn             = 3[dB]
X_pulse           = 6.4[us]
Irr_mode          = Off
Tri_mode          = Off
Dante_presat      = FALSE
Initial_wait      = 1[s]
Recvr_gain        = 38
Relaxation_delay  = 5[s]
Repetition_time   = 7.18365952[s]
Temp_get          = 22.4[dC]
```

Figure S25: 13C NMR Spectrum of Compound 14.

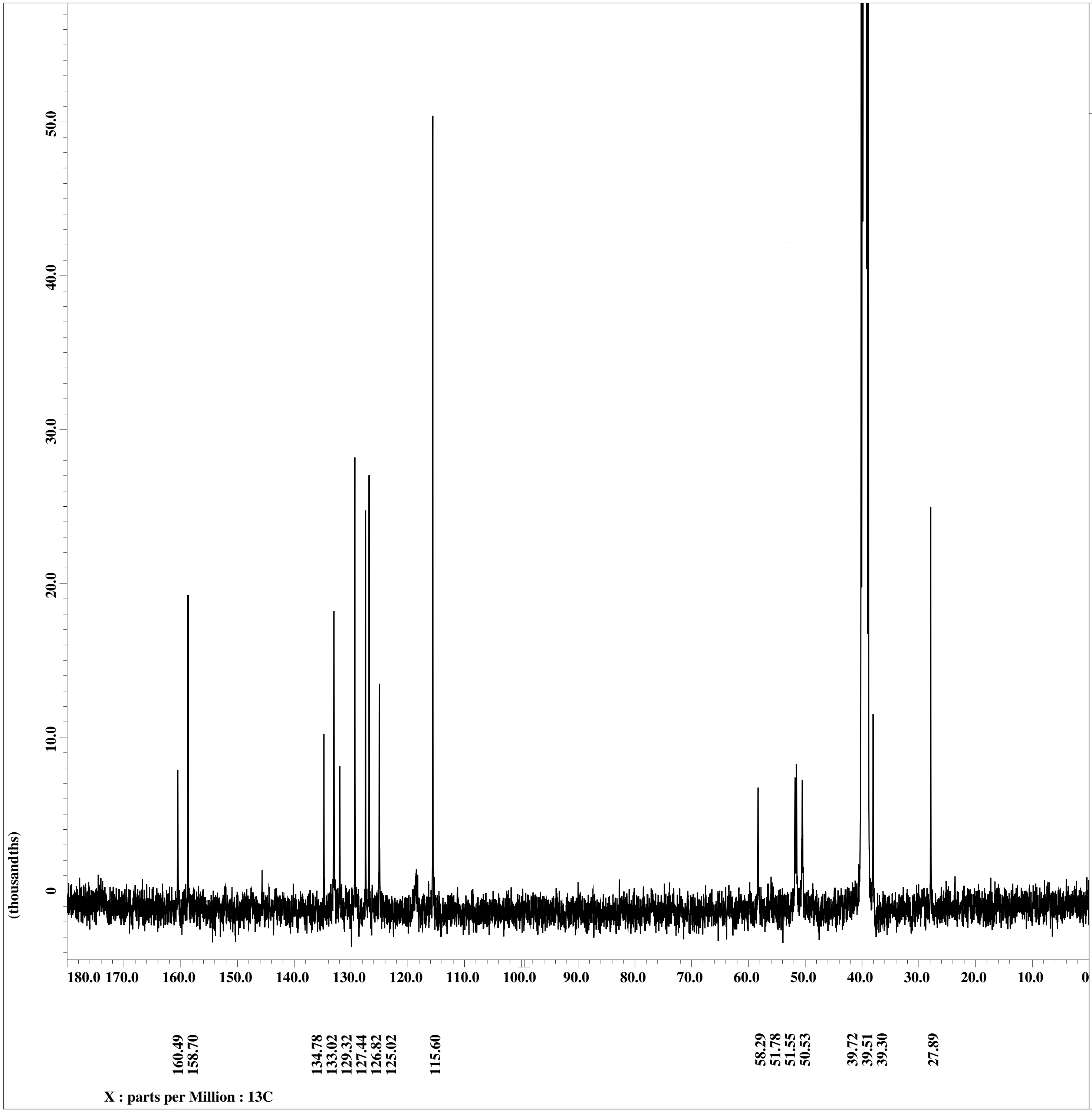

Filename	= BDG-II-083 C13 long (
Author	= chemlabs
Experiment	= single_pulse_dec
Sample_id	= 001
Solvent	= DMSO-D6
Creation_time	= 27-JUN-2016 23:58:09
Revision_time	= 22-JUL-2021 11:32:12
Current_time	= 22-JUL-2021 11:32:39
Comment	= single pulse decouple
Data_format	= 1D COMPLEX
Dim_size	= 26214
Dim_title	= 13C
Dim_units	= [ppm]
Dimensions	= X
Site	= ECS 400
Spectrometer	= JNM-ECS400
Field_strength	= 9.389766[T] (400[MHz]
X_acq_duration	= 1.04333312[s]
X_domain	= 13C
X_freq	= 100.52530333[MHz]
X_offset	= 100[ppm]
X_points	= 32768
X_prescans	= 4
X_resolution	= 0.95846665[Hz]
X_sweep	= 31.40703518[kHz]
Irr_domain	= 1H
Irr_freq	= 399.78219838[MHz]
Irr_offset	= 5[ppm]
Clipped	= FALSE
Mod_return	= 1
Scans	= 8192
Total_scans	= 8192
X_90_width	= 9.5[us]
X_acq_time	= 1.04333312[s]
X_angle	= 30[deg]
X_atn	= 6[dB]
X_pulse	= 3.16666667[us]
Irr_atn_dec	= 21[dB]
Irr_atn_noe	= 21[dB]
Irr_noise	= WALTZ
Decoupling	= TRUE
Initial_wait	= 1[s]
Noe	= TRUE
Noe_time	= 2[s]
Recvr_gain	= 60
Relaxation_delay	= 2[s]
Repetition_time	= 3.04333312[s]
Temp_get	= 23.4[dC]

Figure S26: 1H NMR Spectrum of Compound 15.

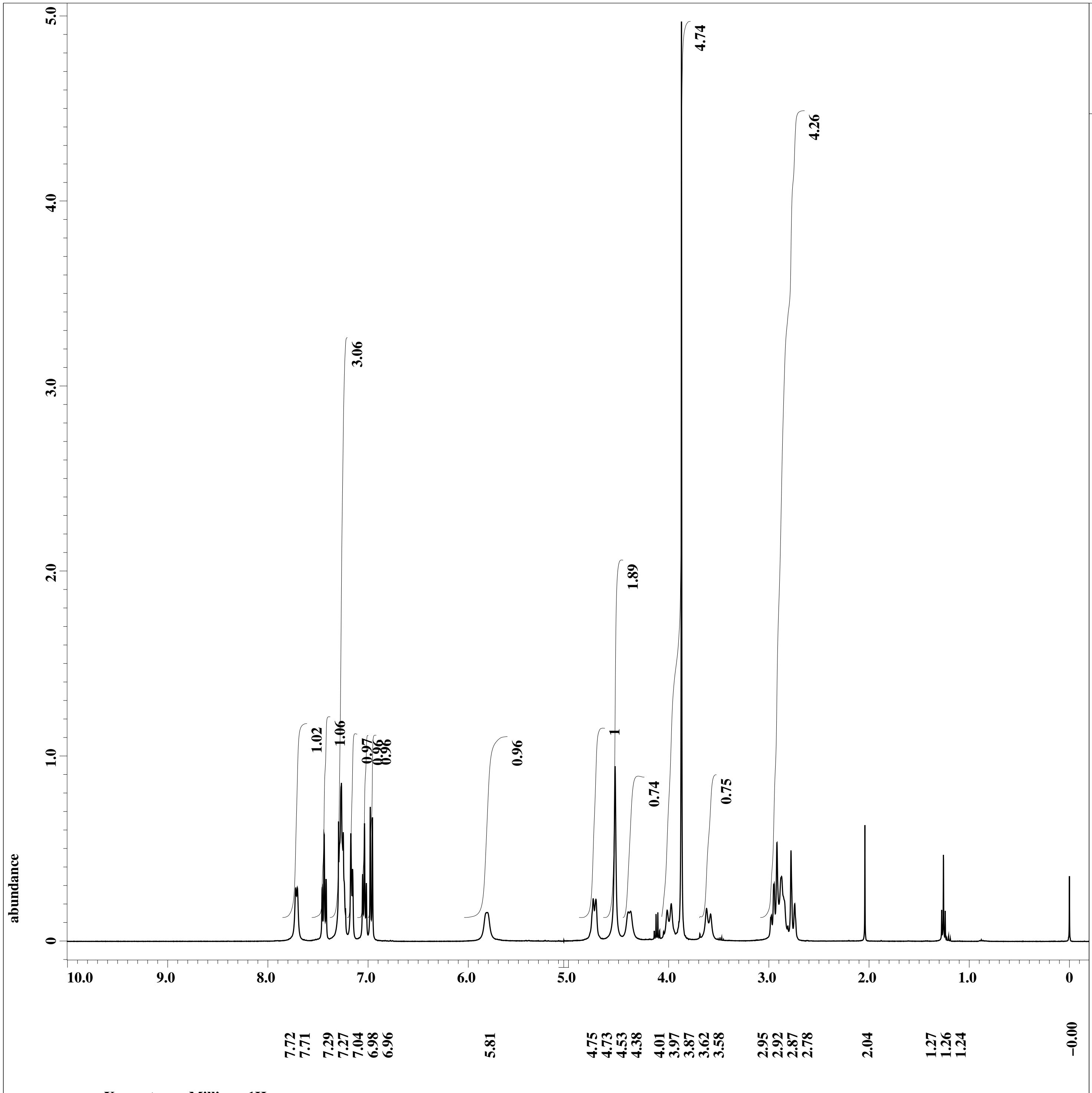

Filename	= BDG-II-117 CdCl3 1H-7
Author	= chemlabs
Experiment	= single_pulse.ex2
Sample_id	= 001
Solvent	= CHLOROFORM-D
Creation_time	= 28-JUL-2017 12:55:55
Revision_time	= 17-JUL-2020 14:09:54
Current_time	= 17-JUL-2020 14:20:24
Comment	= single_pulse
Data_format	= 1D COMPLEX
Dim_size	= 13107
Dim_title	= 1H
Dim_units	= [ppm]
Dimensions	= X
Site	= ECS 400
Spectrometer	= JNM-ECS400
Field_strength	= 9.389766[T] (400[MHz]
X_acq_duration	= 2.18365952[s]
X_domain	= 1H
X_freq	= 399.78219838[MHz]
X_offset	= 5[ppm]
X_points	= 16384
X_prescans	= 1
X_resolution	= 0.45794685[Hz]
X_sweep	= 7.5030012[kHz]
Irr_domain	= 1H
Irr_freq	= 399.78219838[MHz]
Irr_offset	= 5[ppm]
Tri_domain	= 1H
Tri_freq	= 399.78219838[MHz]
Tri_offset	= 5[ppm]
Clipped	= FALSE
Mod_return	= 1
Scans	= 8
Total_scans	= 8
X_90_width	= 14.3[us]
X_acq_time	= 2.18365952[s]
X_angle	= 45[deg]
X_atn	= 3[dB]
X_pulse	= 7.15[us]
Irr_mode	= Off
Tri_mode	= Off
Dante_presat	= FALSE
Initial_wait	= 1[s]
Recvr_gain	= 32
Relaxation_delay	= 5[s]
Repetition_time	= 7.18365952[s]
Temp_get	= 24.7[dC]

Figure S27: 13C NMR Spectrum of Compound 15.

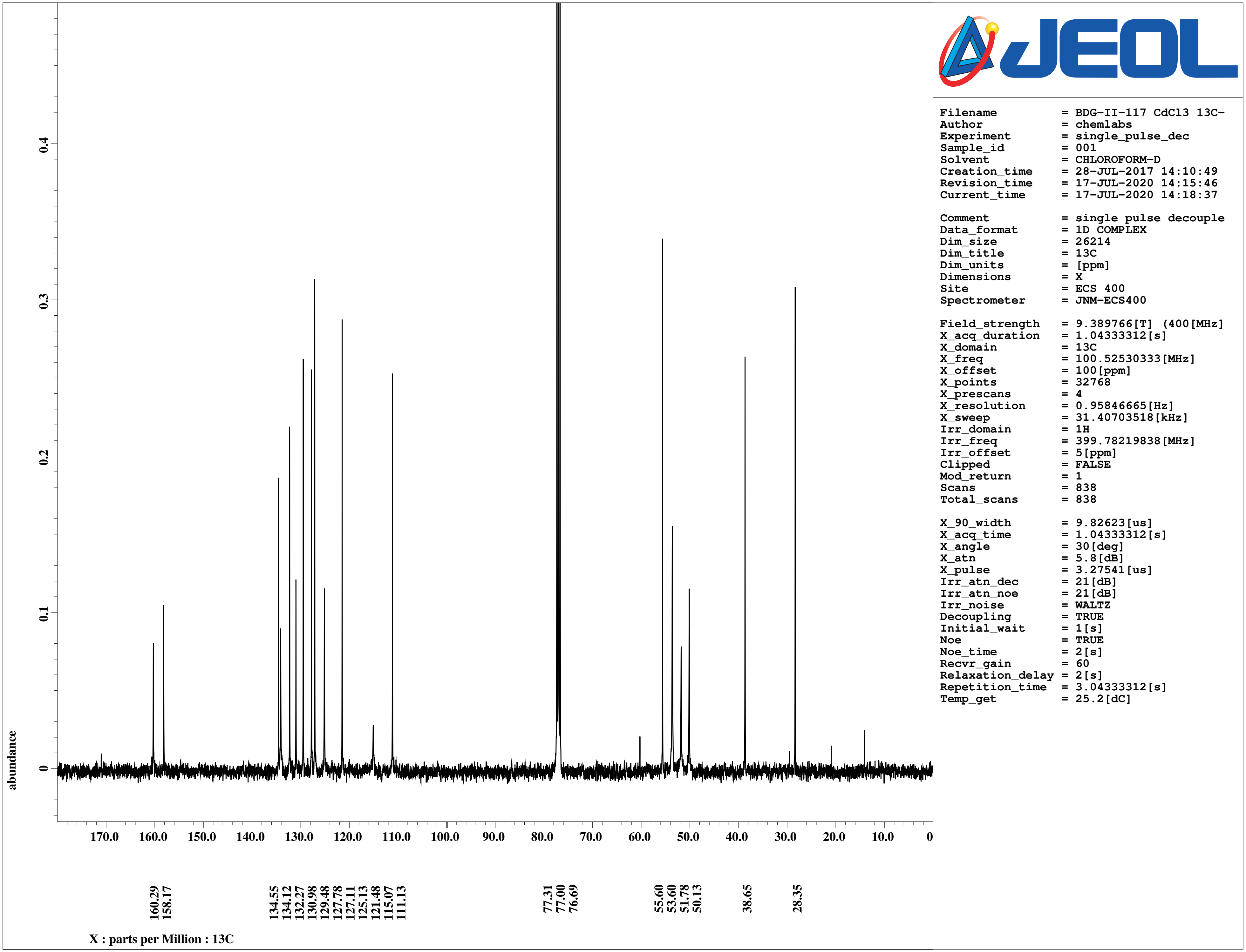

Figure S28: 1H NMR Spectrum of Compound 16.

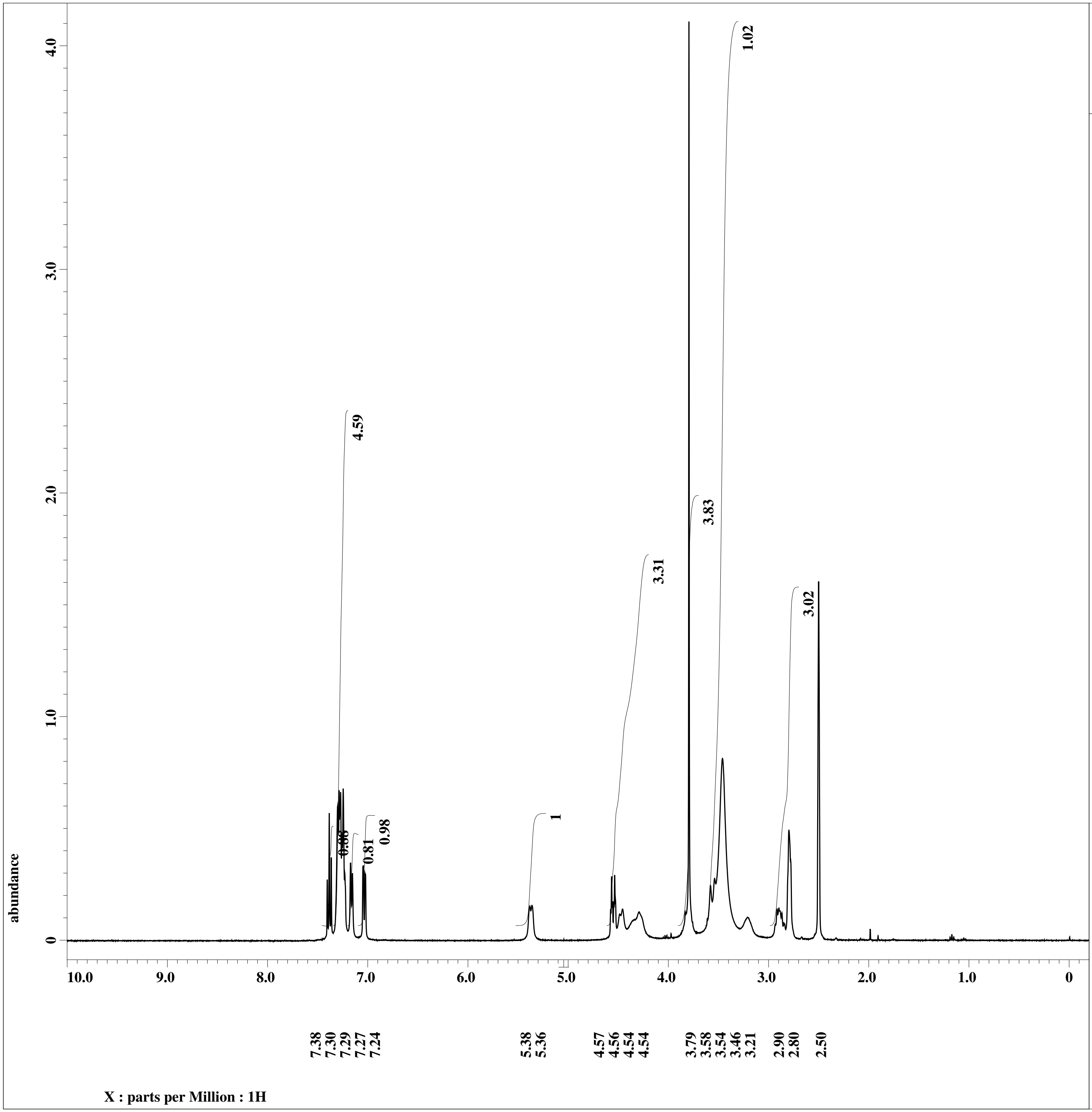

Filename	= BDG-II-099 (DMSO)-4.j
Author	= chemlabs
Experiment	= single_pulse.ex2
Sample_id	= 001
Solvent	= DMSO-D6
Creation_time	= 29-JUN-2016 10:20:42
Revision_time	= 16-JUL-2021 11:30:56
Current_time	= 16-JUL-2021 11:33:06
Comment	= single_pulse
Data_format	= 1D COMPLEX
Dim_size	= 13107
Dim_title	= 1H
Dim_units	= [ppm]
Dimensions	= X
Site	= ECS 400
Spectrometer	= JNM-ECS400
Field_strength	= 9.389766[T] (400[MHz]
X_acq_duration	= 2.18365952[s]
X_domain	= 1H
X_freq	= 399.78219838[MHz]
X_offset	= 5[ppm]
X_points	= 16384
X_prescans	= 1
X_resolution	= 0.45794685[Hz]
X_sweep	= 7.5030012[kHz]
Irr_domain	= 1H
Irr_freq	= 399.78219838[MHz]
Irr_offset	= 5[ppm]
Tri_domain	= 1H
Tri_freq	= 399.78219838[MHz]
Tri_offset	= 5[ppm]
Clipped	= FALSE
Mod_return	= 1
Scans	= 8
Total_scans	= 8
X_90_width	= 12.8[us]
X_acq_time	= 2.18365952[s]
X_angle	= 45[deg]
X_atn	= 3[dB]
X_pulse	= 6.4[us]
Irr_mode	= Off
Tri_mode	= Off
Dante_presat	= FALSE
Initial_wait	= 1[s]
Recvr_gain	= 40
Relaxation_delay	= 5[s]
Repetition_time	= 7.18365952[s]
Temp_get	= 23.1[dC]

Figure S29: 13C NMR Spectrum of Compound 16.

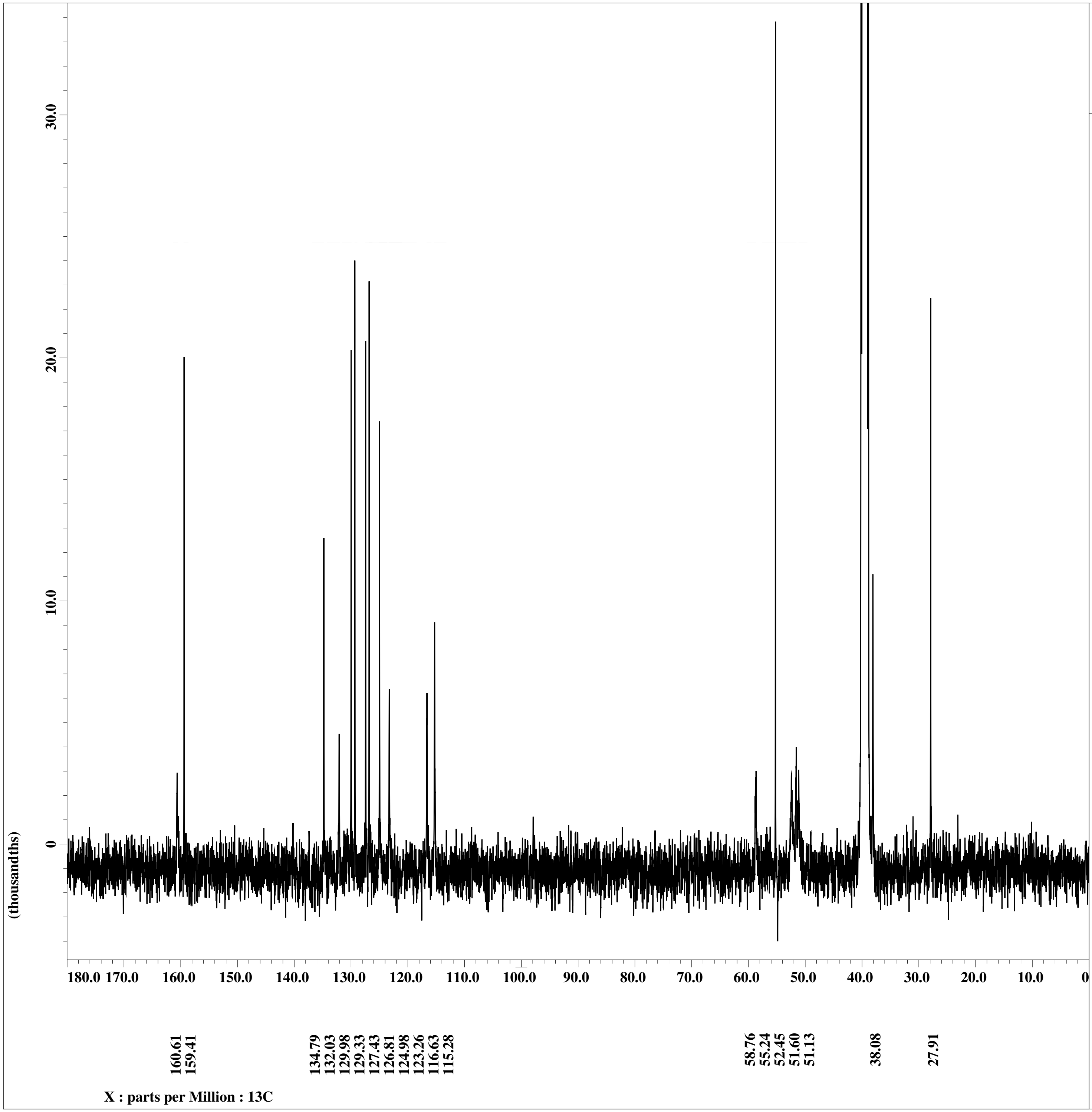


```
Filename          = BDG-II-099 C13 long (
Author            = chemlabs
Experiment        = single_pulse_dec
Sample_id         = 001
Solvent           = DMSO-D6
Creation_time     =  6-JUL-2016 01:54:30
Revision_time     = 16-JUL-2021 11:19:27
Current_time      = 16-JUL-2021 11:22:47

Comment           = single pulse decouple
Data_format       = 1D COMPLEX
Dim_size          = 26214
Dim_title         = 13C
Dim_units         = [ppm]
Dimensions        = X
Site              = ECS 400
Spectrometer      = JNM-ECS400

Field_strength    = 9.389766[T] (400[MHz]
X_acq_duration    = 1.04333312[s]
X_domain          = 13C
X_freq            = 100.52530333[MHz]
X_offset          = 100[ppm]
X_points          = 32768
X_prescans        = 4
X_resolution      = 0.95846665[Hz]
X_sweep           = 31.40703518[kHz]
Irr_domain        = 1H
Irr_freq          = 399.78219838[MHz]
Irr_offset        = 5[ppm]
Clipped           = FALSE
Mod_return        = 1
Scans             = 8192
Total_scans       = 8192

X_90_width        = 9.5[us]
X_acq_time        = 1.04333312[s]
X_angle           = 30[deg]
X_atn             = 6[dB]
X_pulse           = 3.16666667[us]
Irr_atn_dec       = 21[dB]
Irr_atn_noe       = 21[dB]
Irr_noise         = WALTZ
Decoupling        = TRUE
Initial_wait      = 1[s]
Noe               = TRUE
Noe_time          = 2[s]
Recvr_gain        = 60
Relaxation_delay  = 2[s]
Repetition_time   = 3.04333312[s]
Temp_get          = 23.9[dC]
```

Figure S30: 1H NMR Spectrum of Compound 17.

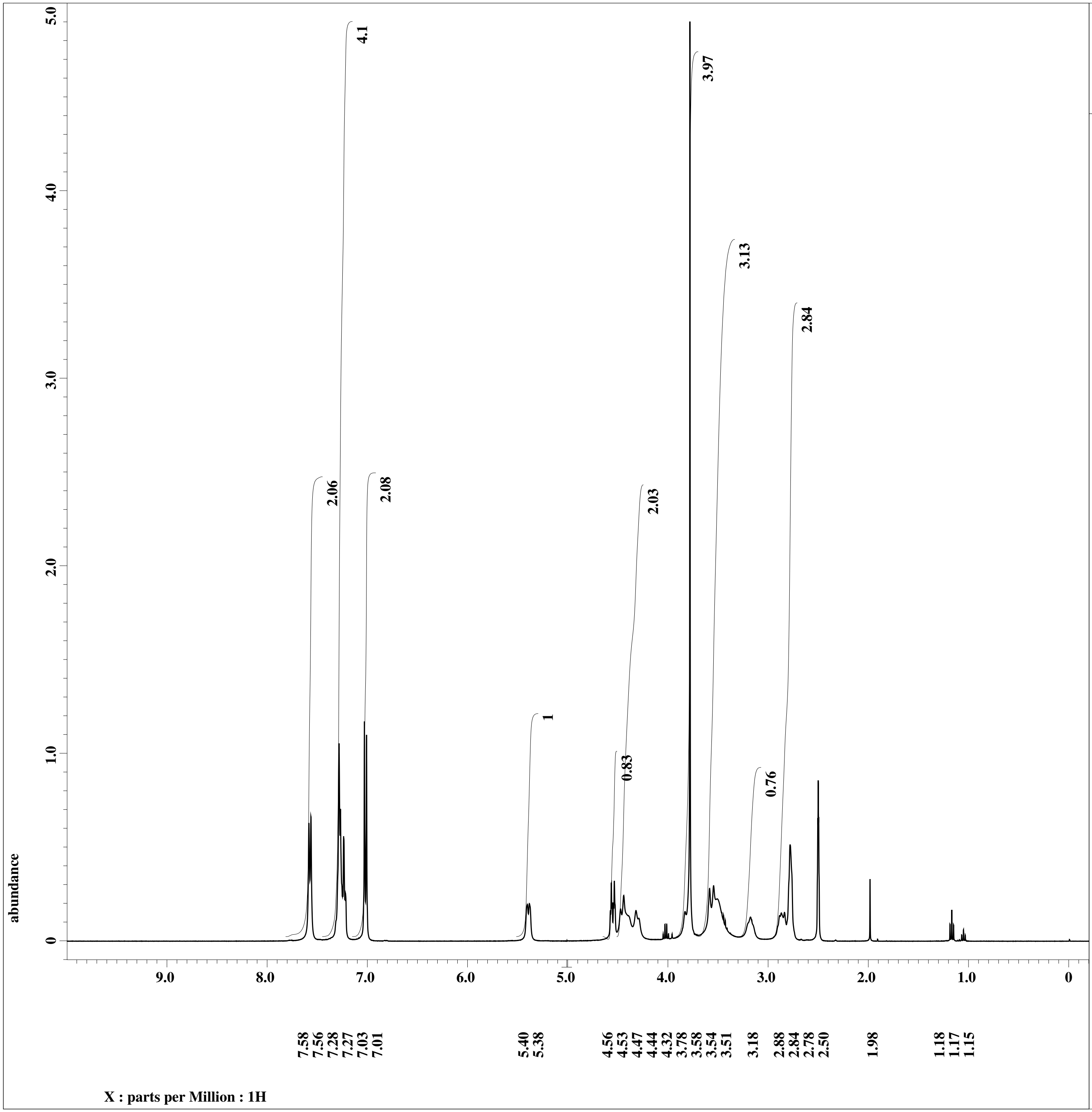

Filename	=	TAR-I-001 1H NMR Work
Author	=	russell research stud
Experiment	=	single_pulse.ex2
Sample_id	=	S#860467
Solvent	=	DMSO-D6
Creation_time	=	6-OCT-2019 21:28:38
Revision_time	=	12-JUN-2025 12:04:24
Current_time	=	12-JUN-2025 12:04:35
Data_format	=	1D COMPLEX
Dim_size	=	13107
Dim_title	=	1H
Dim_units	=	[ppm]
Dimensions	=	X
Site	=	ECS 400
Spectrometer	=	JNM-ECS400
Field_strength	=	9.389766[T] (400[MHz]
X_acq_duration	=	2.18365952[s]
X_domain	=	1H
X_freq	=	399.78219838[MHz]
X_offset	=	5[ppm]
X_points	=	16384
X_prescans	=	1
X_resolution	=	0.45794685[Hz]
X_sweep	=	7.5030012[kHz]
Irr_domain	=	1H
Irr_freq	=	399.78219838[MHz]
Irr_offset	=	5[ppm]
Tri_domain	=	1H
Tri_freq	=	399.78219838[MHz]
Tri_offset	=	5[ppm]
Clipped	=	FALSE
Mod_return	=	1
Scans	=	8
Total_scans	=	8
X_90_width	=	14.3[us]
X_acq_time	=	2.18365952[s]
X_angle	=	45[deg]
X_atn	=	3[dB]
X_pulse	=	7.15[us]
Irr_mode	=	Off
Tri_mode	=	Off
Dante_presat	=	FALSE
Initial_wait	=	1[s]
Recvr_gain	=	32
Relaxation_delay	=	5[s]
Repetition_time	=	7.18365952[s]
Temp_get	=	22.2[dC]

Figure S31: 13C NMR Spectrum of Compound 17.

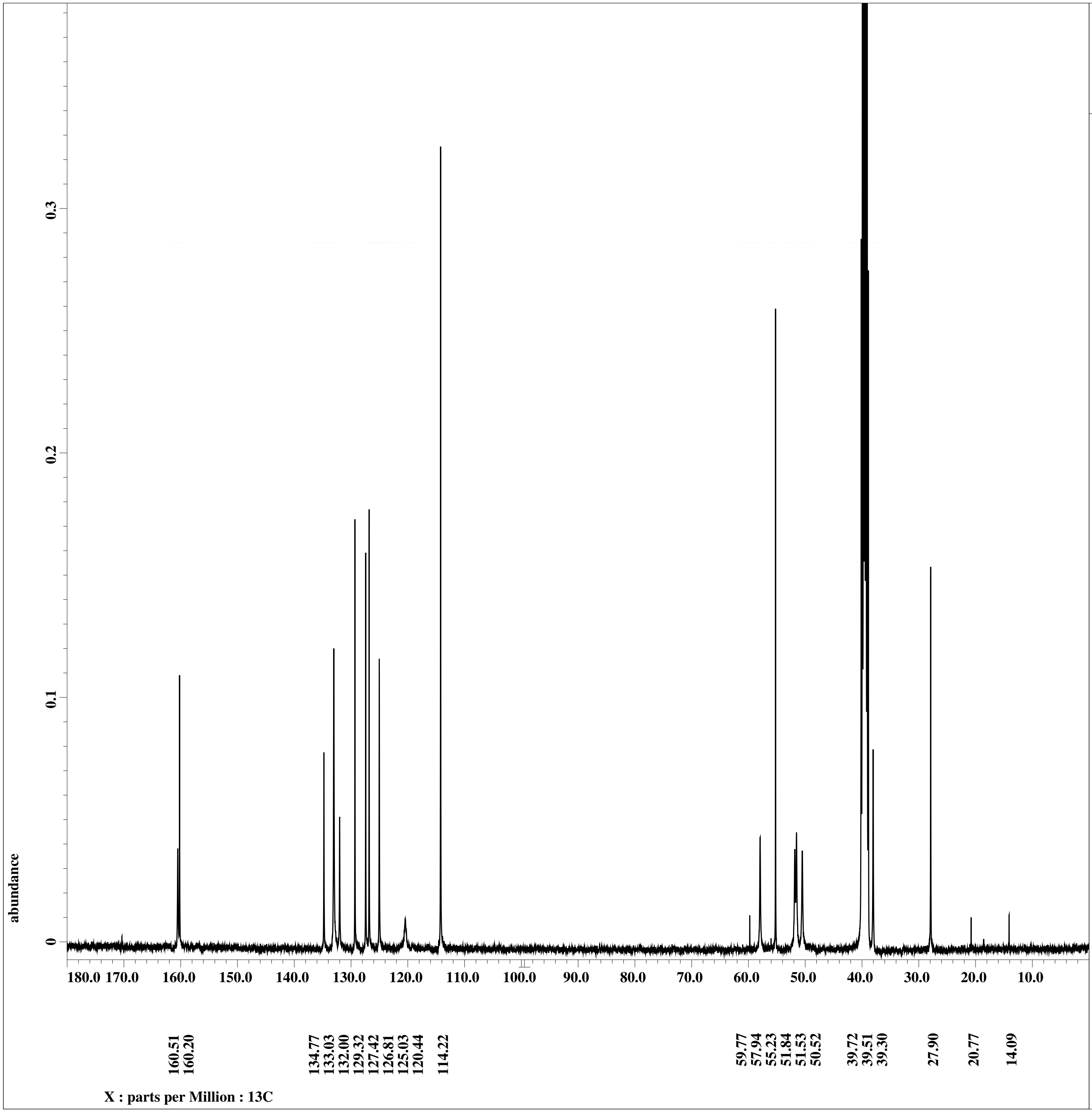

Filename	= TAR-I-001 13C NMR Wor
Author	= russell research stud
Experiment	= single_pulse_dec
Sample_id	= S#863197
Solvent	= DMSO-D6
Creation_time	= 7-OCT-2019 06:29:10
Revision_time	= 21-OCT-2019 09:28:44
Current_time	= 12-JUN-2025 12:06:16
Data_format	= 1D COMPLEX
Dim_size	= 26214
Dim_title	= 13C
Dim_units	= [ppm]
Dimensions	= X
Site	= ECS 400
Spectrometer	= JNM-ECS400
Field_strength	= 9.389766[T] (400[MHz]
X_acq_duration	= 1.04333312[s]
X_domain	= 13C
X_freq	= 100.52530333[MHz]
X_offset	= 100[ppm]
X_points	= 32768
X_prescans	= 4
X_resolution	= 0.95846665[Hz]
X_sweep	= 31.40703518[kHz]
Irr_domain	= 1H
Irr_freq	= 399.78219838[MHz]
Irr_offset	= 5[ppm]
Clipped	= FALSE
Mod_return	= 1
Scans	= 10593.0
Total_scans	= 10593.0
X_90_width	= 9.82623[us]
X_acq_time	= 1.04333312[s]
X_angle	= 30[deg]
X_atn	= 5.8[dB]
X_pulse	= 3.27541[us]
Irr_atn_dec	= 21[dB]
Irr_atn_noe	= 21[dB]
Irr_noise	= WALTZ
Decoupling	= TRUE
Initial_wait	= 1[s]
Noe	= TRUE
Noe_time	= 2[s]
Recvr_gain	= 60
Relaxation_delay	= 2[s]
Repetition_time	= 3.04333312[s]
Temp_get	= 22.6[dC]

Figure S32: 1H NMR Spectrum of Compound 18.

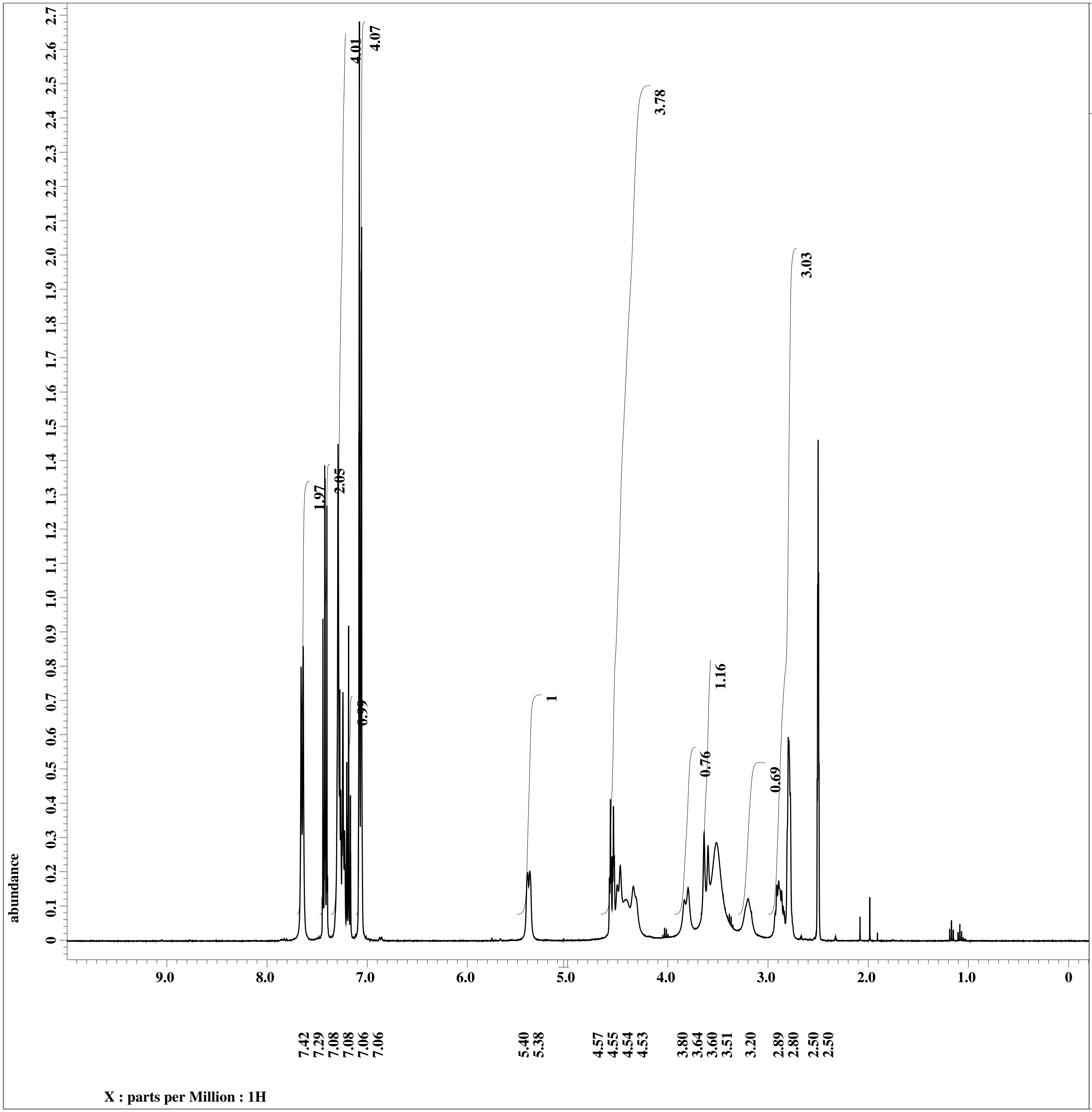

Filename	= BDG-II-131 (DMSO) 1H
Author	= chemlabs
Experiment	= single_pulse.ex2
Sample_id	= 001
Solvent	= DMSO-D6
Creation_time	= 22-JUL-2017 08:46:24
Revision_time	= 19-JUL-2021 14:57:12
Current_time	= 19-JUL-2021 14:58:19
Comment	= single_pulse
Data_format	= 1D COMPLEX
Dim_size	= 13107
Dim_title	= 1H
Dim_units	= [ppm]
Dimensions	= X
Site	= ECS 400
Spectrometer	= JNM-ECS400
Field_strength	= 9.389766[T] (400[MHz]
X_acq_duration	= 2.18365952[s]
X_domain	= 1H
X_freq	= 399.78219838[MHz]
X_offset	= 5[ppm]
X_points	= 16384
X_prescans	= 1
X_resolution	= 0.45794685[Hz]
X_sweep	= 7.5030012[kHz]
Irr_domain	= 1H
Irr_freq	= 399.78219838[MHz]
Irr_offset	= 5[ppm]
Tri_domain	= 1H
Tri_freq	= 399.78219838[MHz]
Tri_offset	= 5[ppm]
Clipped	= FALSE
Mod_return	= 1
Scans	= 8
Total_scans	= 8
X_90_width	= 14.3[us]
X_acq_time	= 2.18365952[s]
X_angle	= 45[deg]
X_atn	= 3[dB]
X_pulse	= 7.15[us]
Irr_mode	= Off
Tri_mode	= Off
Dante_presat	= FALSE
Initial_wait	= 1[s]
Recvr_gain	= 36
Relaxation_delay	= 5[s]
Repetition_time	= 7.18365952[s]
Temp_get	= 23.4[dC]

Figure S33: 13C NMR Spectrum of Compound 18.

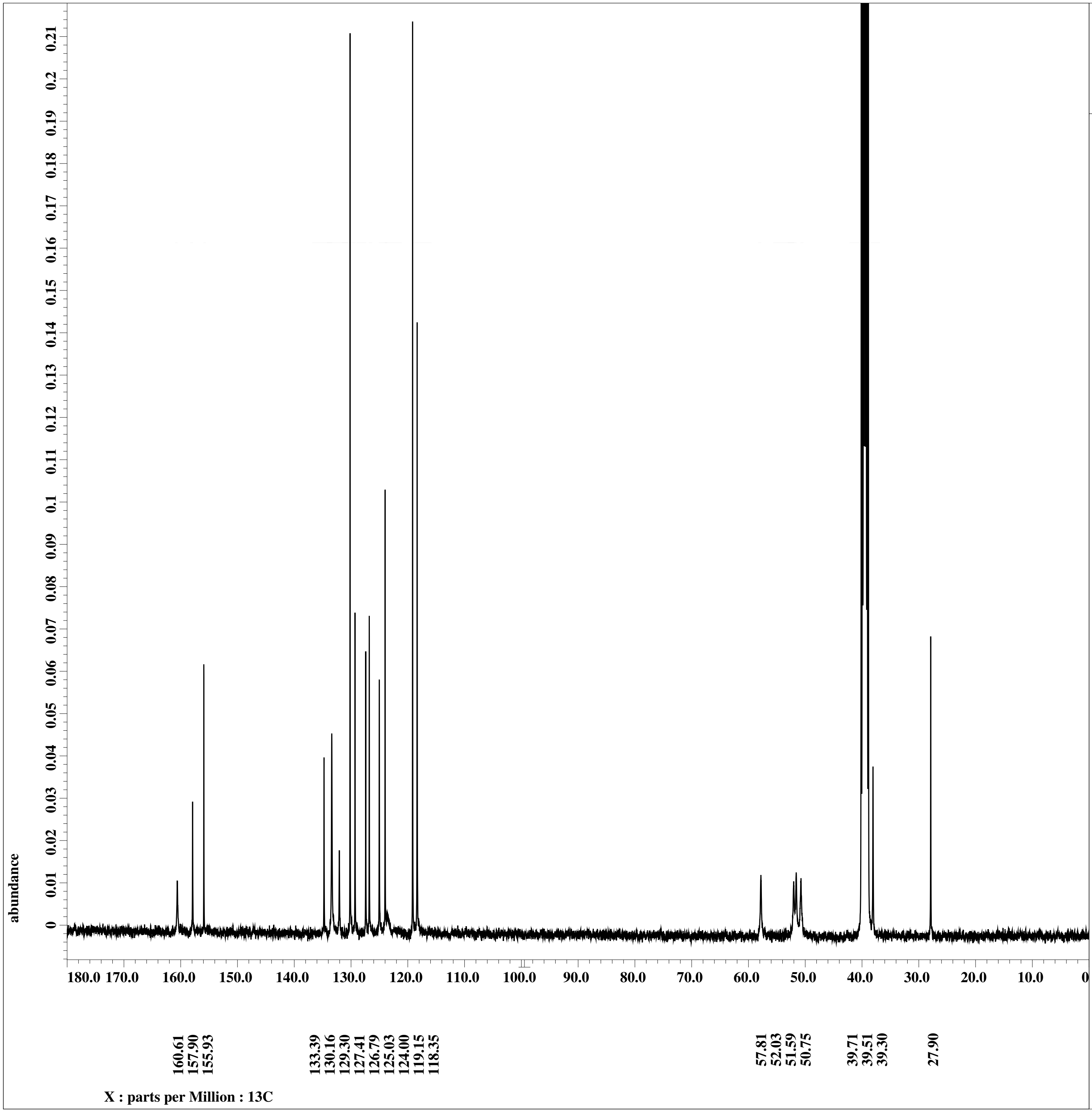


```
Filename           = BDG-II-131 (DMSO) 13C
Author             = chemlabs
Experiment         = single_pulse_dec
Sample_id          = 001
Solvent            = DMSO-D6
Creation_time      = 22-JUL-2017 19:50:54
Revision_time      = 19-JUL-2021 14:59:17
Current_time       = 19-JUL-2021 15:07:15

Comment            = single pulse decouple
Data_format        = 1D COMPLEX
Dim_size           = 26214
Dim_title          = 13C
Dim_units          = [ppm]
Dimensions         = X
Site               = ECS 400
Spectrometer       = JNM-ECS400

Field_strength     = 9.389766[T] (400[MHz]
X_acq_duration     = 1.04333312[s]
X_domain           = 13C
X_freq             = 100.52530333[MHz]
X_offset           = 100[ppm]
X_points           = 32768
X_prescans         = 4
X_resolution       = 0.95846665[Hz]
X_sweep            = 31.40703518[kHz]
Irr_domain         = 1H
Irr_freq           = 399.78219838[MHz]
Irr_offset         = 5[ppm]
Clipped            = FALSE
Mod_return         = 1
Scans              = 13076
Total_scans        = 13076

X_90_width         = 9.82623[us]
X_acq_time         = 1.04333312[s]
X_angle            = 30[deg]
X_atn              = 5.8[dB]
X_pulse            = 3.27541[us]
Irr_atn_dec        = 21[dB]
Irr_atn_noe        = 21[dB]
Irr_noise          = WALTZ
Decoupling         = TRUE
Initial_wait       = 1[s]
Noe                = TRUE
Noe_time           = 2[s]
Recvr_gain         = 60
Relaxation_delay   = 2[s]
Repetition_time    = 3.04333312[s]
Temp_get           = 24.2[dC]
```

Figure S34: 1H NMR Spectrum of Compound 19.

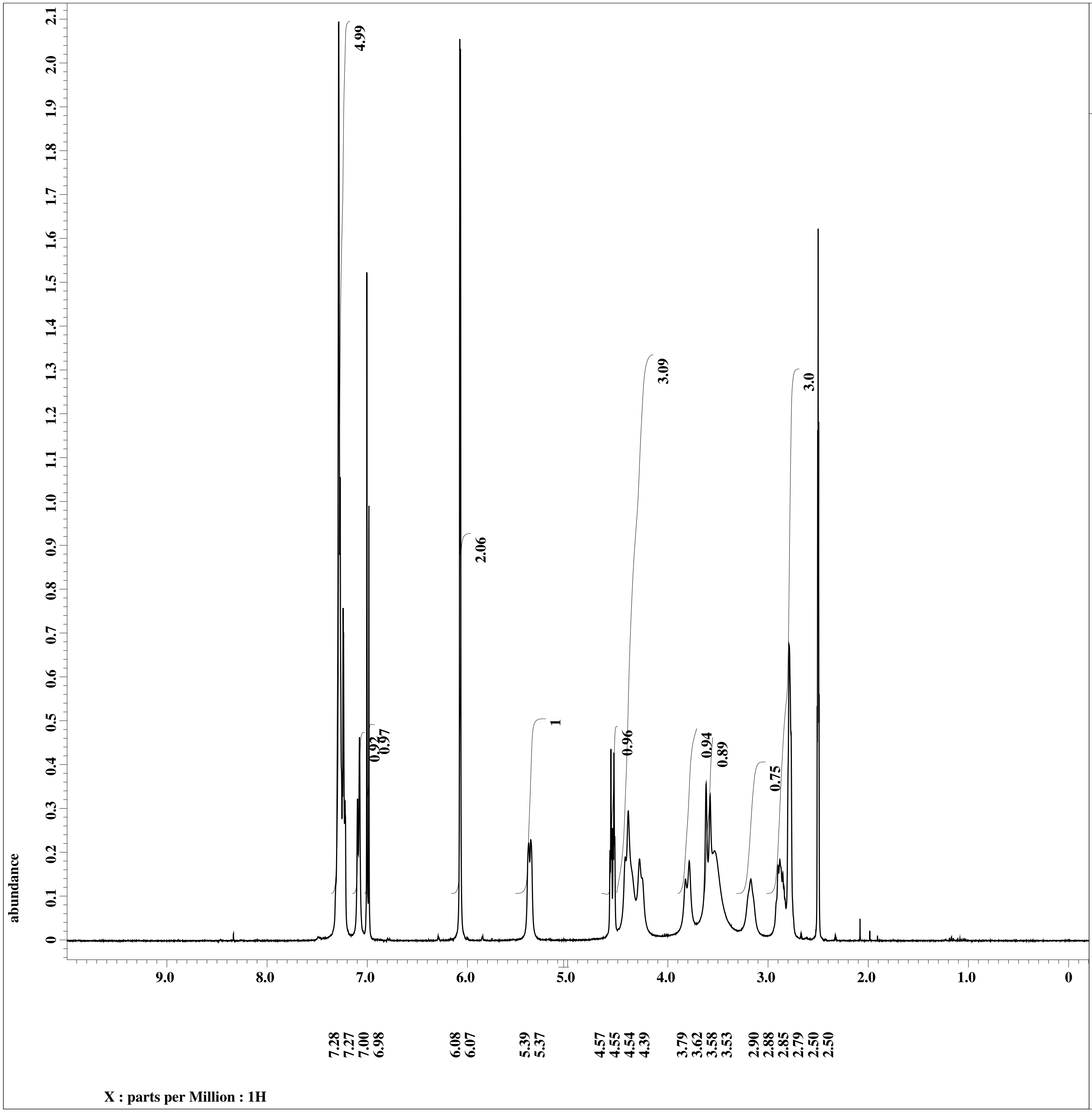


```
Filename           = LSC-1-091 DMSO 1H NMR
Author             = chemlabs
Experiment         = single_pulse.ex2
Sample_id          = 001
Solvent            = DMSO-D6
Creation_time      = 24-JUL-2017 11:29:39
Revision_time      = 19-JUL-2021 15:13:46
Current_time       = 19-JUL-2021 15:18:01

Comment            = single_pulse
Data_format        = 1D COMPLEX
Dim_size           = 13107
Dim_title          = 1H
Dim_units          = [ppm]
Dimensions         = X
Site               = ECS 400
Spectrometer       = JNM-ECS400

Field_strength     = 9.389766[T] (400[MHz]
X_acq_duration     = 2.18365952[s]
X_domain           = 1H
X_freq             = 399.78219838[MHz]
X_offset           = 5[ppm]
X_points           = 16384
X_prescans         = 1
X_resolution       = 0.45794685[Hz]
X_sweep            = 7.5030012[kHz]
Irr_domain         = 1H
Irr_freq           = 399.78219838[MHz]
Irr_offset         = 5[ppm]
Tri_domain         = 1H
Tri_freq           = 399.78219838[MHz]
Tri_offset         = 5[ppm]
Clipped            = FALSE
Mod_return         = 1
Scans              = 8
Total_scans        = 8

X_90_width         = 14.3[us]
X_acq_time         = 2.18365952[s]
X_angle            = 45[deg]
X_atn              = 3[dB]
X_pulse            = 7.15[us]
Irr_mode           = Off
Tri_mode           = Off
Dante_presat       = FALSE
Initial_wait       = 1[s]
Recvr_gain         = 36
Relaxation_delay   = 5[s]
Repetition_time    = 7.18365952[s]
Temp_get           = 24[dC]
```

Figure S35: 13C NMR Spectrum of Compound 19.

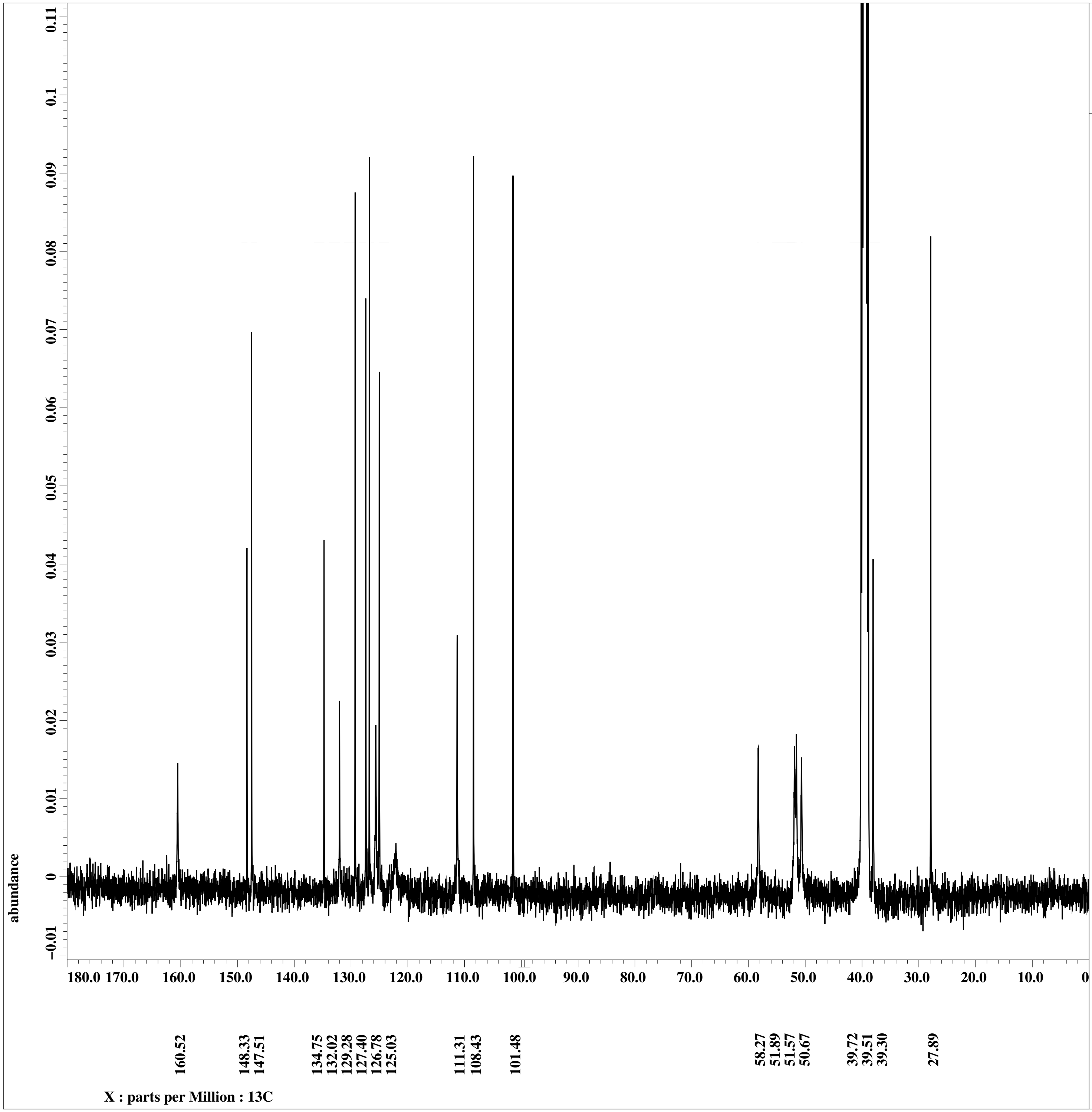

Filename = LSC-1-091 DMSO 13C NM
Author = chemlabs
Experiment = single_pulse_dec
Sample_id = 001
Solvent = DMSO-D6
Creation_time = 24-JUL-2017 14:06:27
Revision_time = 19-JUL-2021 15:19:09
Current_time = 19-JUL-2021 15:22:16

Comment = single pulse decouple
Data_format = 1D COMPLEX
Dim_size = 26214
Dim_title = 13C
Dim_units = [ppm]
Dimensions = X
Site = ECS 400
Spectrometer = JNM-ECS400

Field_strength = 9.389766[T] (400[MHz]
X_acq_duration = 1.04333312[s]
X_domain = 13C
X_freq = 100.52530333[MHz]
X_offset = 100[ppm]
X_points = 32768
X_prescans = 4
X_resolution = 0.95846665[Hz]
X_sweep = 31.40703518[kHz]
Irr_domain = 1H
Irr_freq = 399.78219838[MHz]
Irr_offset = 5[ppm]
Clipped = FALSE
Mod_return = 1
Scans = 3081
Total_scans = 3081

X_90_width = 9.82623[us]
X_acq_time = 1.04333312[s]
X_angle = 30[deg]
X_atn = 5.8[dB]
X_pulse = 3.27541[us]
Irr_atn_dec = 21[dB]
Irr_atn_noe = 21[dB]
Irr_noise = WALTZ
Decoupling = TRUE
Initial_wait = 1[s]
Noe = TRUE
Noe_time = 2[s]
Recvr_gain = 60
Relaxation_delay = 2[s]
Repetition_time = 3.04333312[s]
Temp_get = 25[dC]

Figure S36: 1H NMR Spectrum of Compound 20.

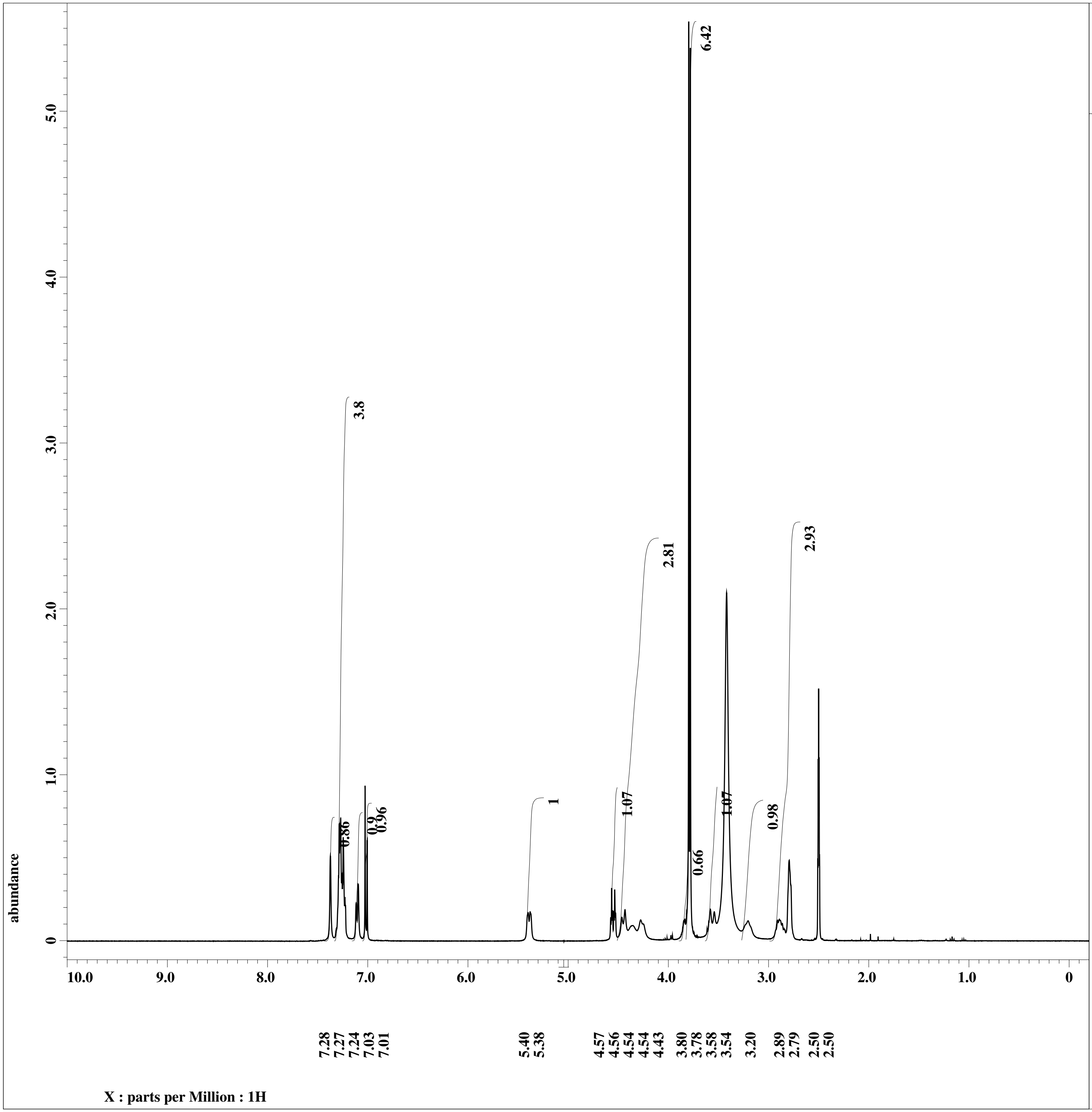

Filename	= BDG-II-081 in DMSO a-
Author	= chemlabs
Experiment	= single_pulse.ex2
Sample_id	= 001
Solvent	= DMSO-D6
Creation_time	= 17-JUN-2016 11:19:59
Revision_time	= 21-JUL-2021 10:10:02
Current_time	= 21-JUL-2021 10:10:42
Comment	= single_pulse
Data_format	= 1D COMPLEX
Dim_size	= 13107
Dim_title	= 1H
Dim_units	= [ppm]
Dimensions	= X
Site	= ECS 400
Spectrometer	= JNM-ECS400
Field_strength	= 9.389766[T] (400[MHz]
X_acq_duration	= 2.18365952[s]
X_domain	= 1H
X_freq	= 399.78219838[MHz]
X_offset	= 5[ppm]
X_points	= 16384
X_prescans	= 1
X_resolution	= 0.45794685[Hz]
X_sweep	= 7.5030012[kHz]
Irr_domain	= 1H
Irr_freq	= 399.78219838[MHz]
Irr_offset	= 5[ppm]
Tri_domain	= 1H
Tri_freq	= 399.78219838[MHz]
Tri_offset	= 5[ppm]
Clipped	= FALSE
Mod_return	= 1
Scans	= 8
Total_scans	= 8
X_90_width	= 12.8[us]
X_acq_time	= 2.18365952[s]
X_angle	= 45[deg]
X_atn	= 3[dB]
X_pulse	= 6.4[us]
Irr_mode	= Off
Tri_mode	= Off
Dante_presat	= FALSE
Initial_wait	= 1[s]
Recvr_gain	= 36
Relaxation_delay	= 5[s]
Repetition_time	= 7.18365952[s]
Temp_get	= 22.9[dC]

Figure S37: 13C NMR Spectrum of Compound 20.

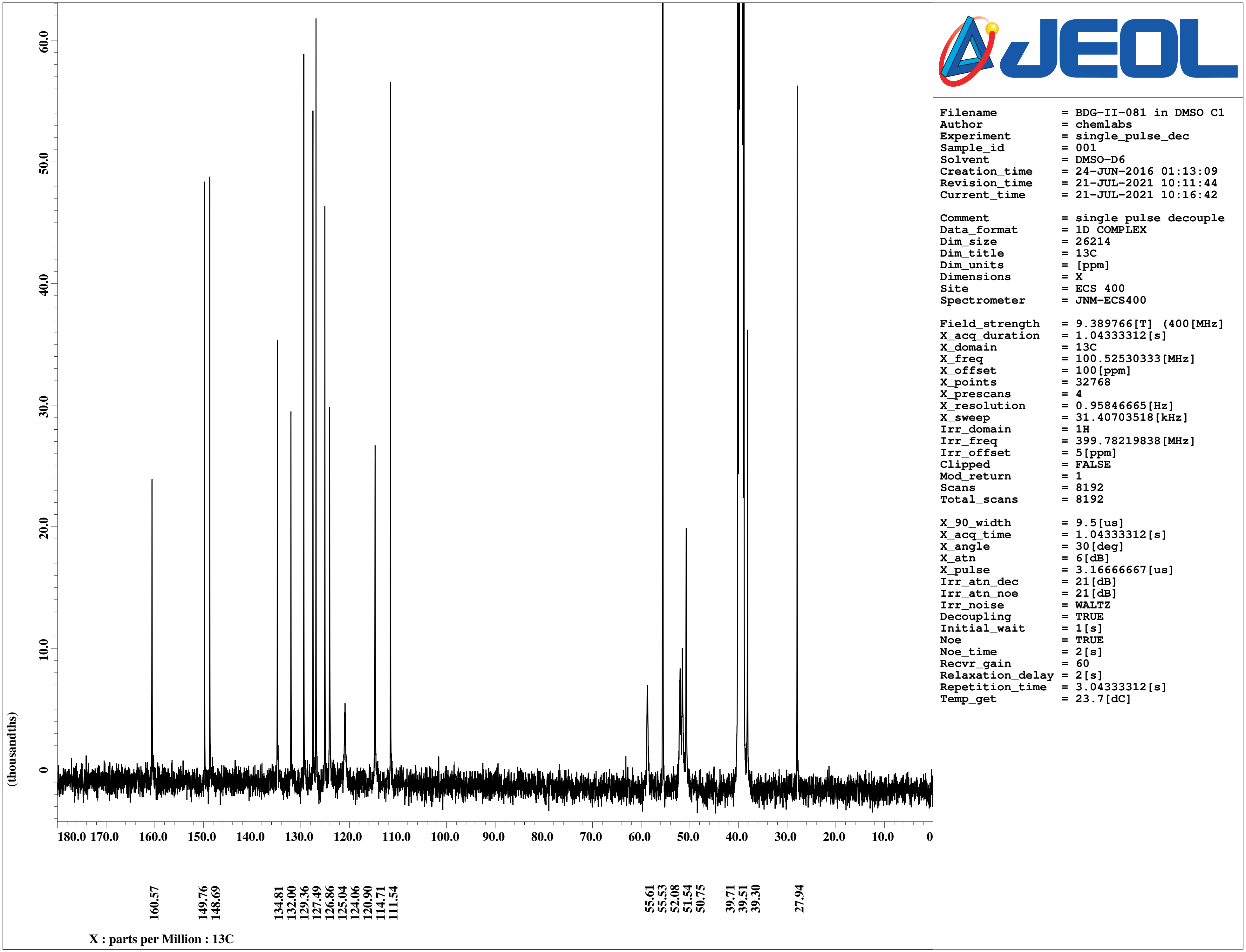

Figure S38: 1H NMR Spectrum of Compound 21.

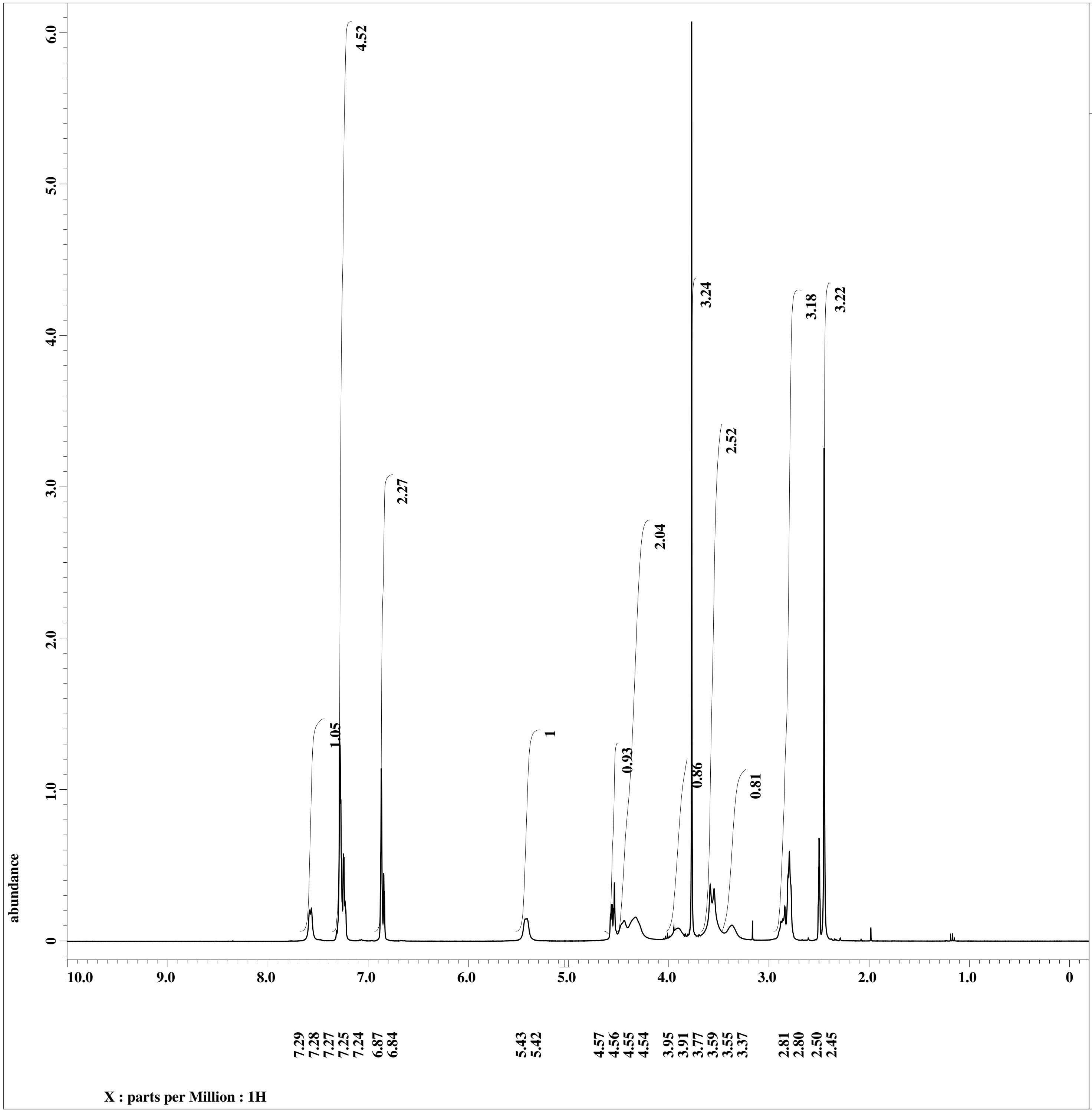


```
Filename           = BDG-II-135 DMSO 1H NM
Author             = chemlabs
Experiment         = single_pulse.ex2
Sample_id          = 001
Solvent            = DMSO-D6
Creation_time      = 27-JUL-2017 08:23:31
Revision_time      = 21-JUL-2021 10:22:53
Current_time       = 21-JUL-2021 10:26:51

Comment            = single_pulse
Data_format        = 1D COMPLEX
Dim_size           = 13107
Dim_title          = 1H
Dim_units          = [ppm]
Dimensions         = X
Site               = ECS 400
Spectrometer       = JNM-ECS400

Field_strength     = 9.389766[T] (400[MHz]
X_acq_duration     = 2.18365952[s]
X_domain           = 1H
X_freq             = 399.78219838[MHz]
X_offset           = 5[ppm]
X_points           = 16384
X_prescans         = 1
X_resolution       = 0.45794685[Hz]
X_sweep            = 7.5030012[kHz]
Irr_domain         = 1H
Irr_freq           = 399.78219838[MHz]
Irr_offset         = 5[ppm]
Tri_domain         = 1H
Tri_freq           = 399.78219838[MHz]
Tri_offset         = 5[ppm]
Clipped            = FALSE
Mod_return         = 1
Scans              = 8
Total_scans        = 8

X_90_width         = 14.3[us]
X_acq_time         = 2.18365952[s]
X_angle            = 45[deg]
X_atn              = 3[dB]
X_pulse            = 7.15[us]
Irr_mode           = Off
Tri_mode           = Off
Dante_presat       = FALSE
Initial_wait       = 1[s]
Recvr_gain         = 30
Relaxation_delay   = 5[s]
Repetition_time    = 7.18365952[s]
Temp_get           = 24.1[dC]
```

Figure S39: 13C NMR Spectrum of Compound 21.

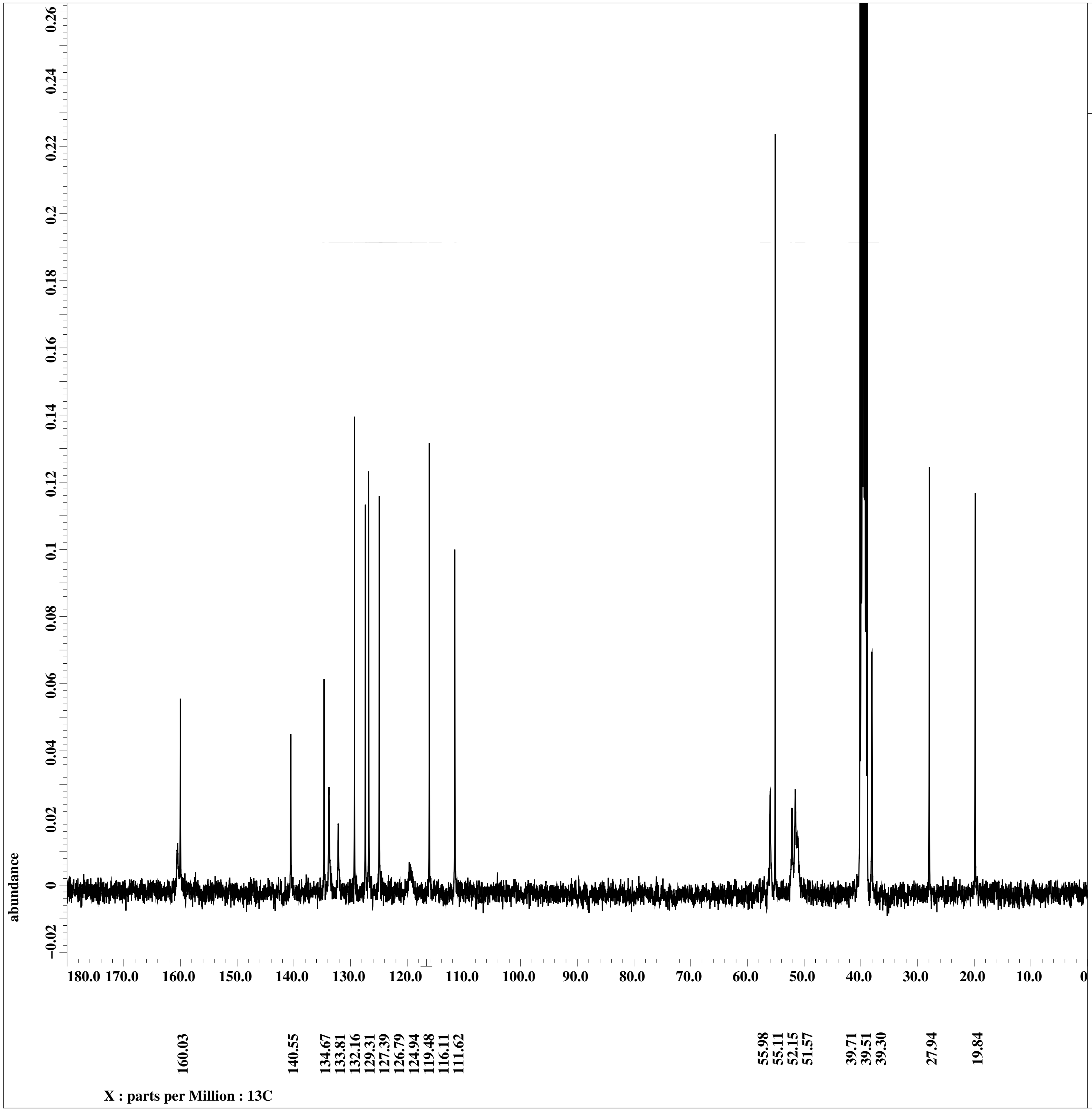


```
Filename          = BDG-II-135 DMSO 13C N
Author            = chemlabs
Experiment        = single_pulse_dec
Sample_id         = 001
Solvent           = DMSO-D6
Creation_time     = 27-JUL-2017 09:36:33
Revision_time     = 21-JUL-2021 10:34:02
Current_time      = 21-JUL-2021 10:37:49

Comment           = single pulse decouple
Data_format       = 1D COMPLEX
Dim_size          = 26214
Dim_title         = 13C
Dim_units         = [ppm]
Dimensions        = X
Site              = ECS 400
Spectrometer      = JNM-ECS400

Field_strength    = 9.389766[T] (400[MHz]
X_acq_duration    = 1.04333312[s]
X_domain          = 13C
X_freq            = 100.52530333[MHz]
X_offset          = 100[ppm]
X_points          = 32768
X_prescans        = 4
X_resolution      = 0.95846665[Hz]
X_sweep           = 31.40703518[kHz]
Irr_domain        = 1H
Irr_freq          = 399.78219838[MHz]
Irr_offset        = 5[ppm]
Clipped           = FALSE
Mod_return        = 1
Scans             = 1400
Total_scans       = 1400

X_90_width        = 9.82623[us]
X_acq_time        = 1.04333312[s]
X_angle           = 30[deg]
X_atn             = 5.8[dB]
X_pulse           = 3.27541[us]
Irr_atn_dec       = 21[dB]
Irr_atn_noe       = 21[dB]
Irr_noise         = WALTZ
Decoupling        = TRUE
Initial_wait      = 1[s]
Noe               = TRUE
Noe_time          = 2[s]
Recvr_gain        = 60
Relaxation_delay  = 2[s]
Repetition_time   = 3.04333312[s]
Temp_get          = 24.5[dC]
```

Figure S40: 1H NMR Spectrum of Compound 22.

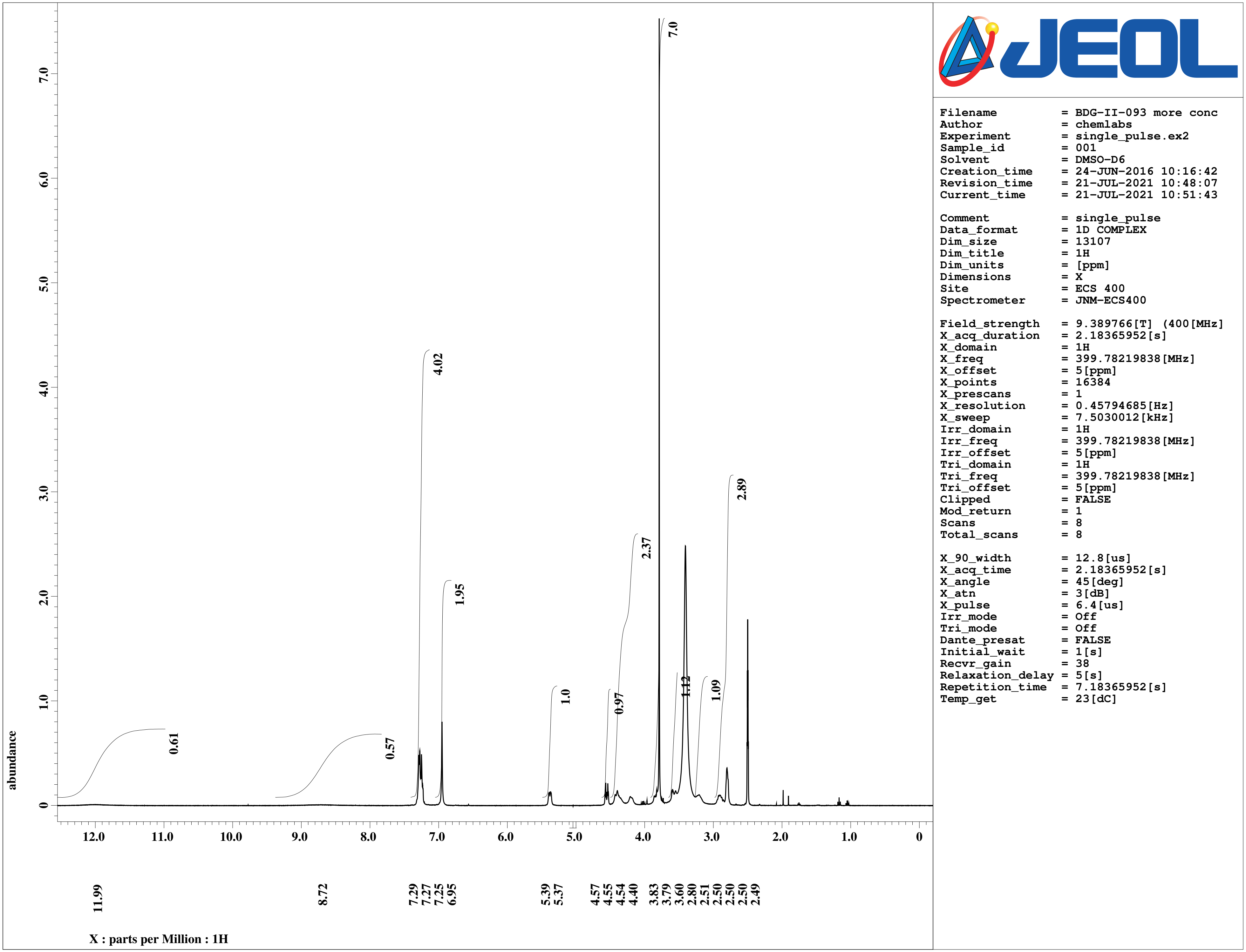

Figure S41: 13C NMR Spectrum of Compound 22.

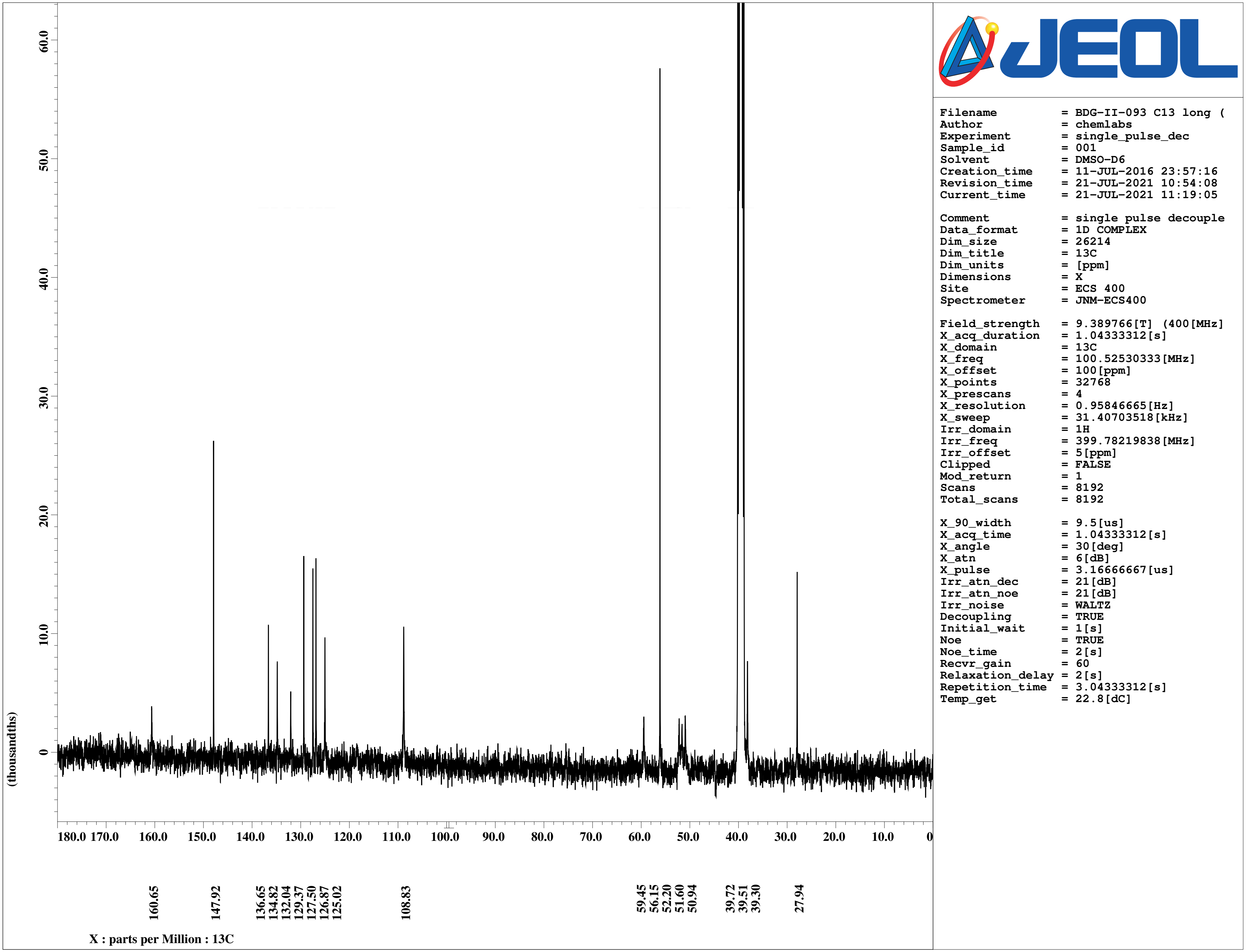

Figure S42: 1H NMR Spectrum of Compound 23.

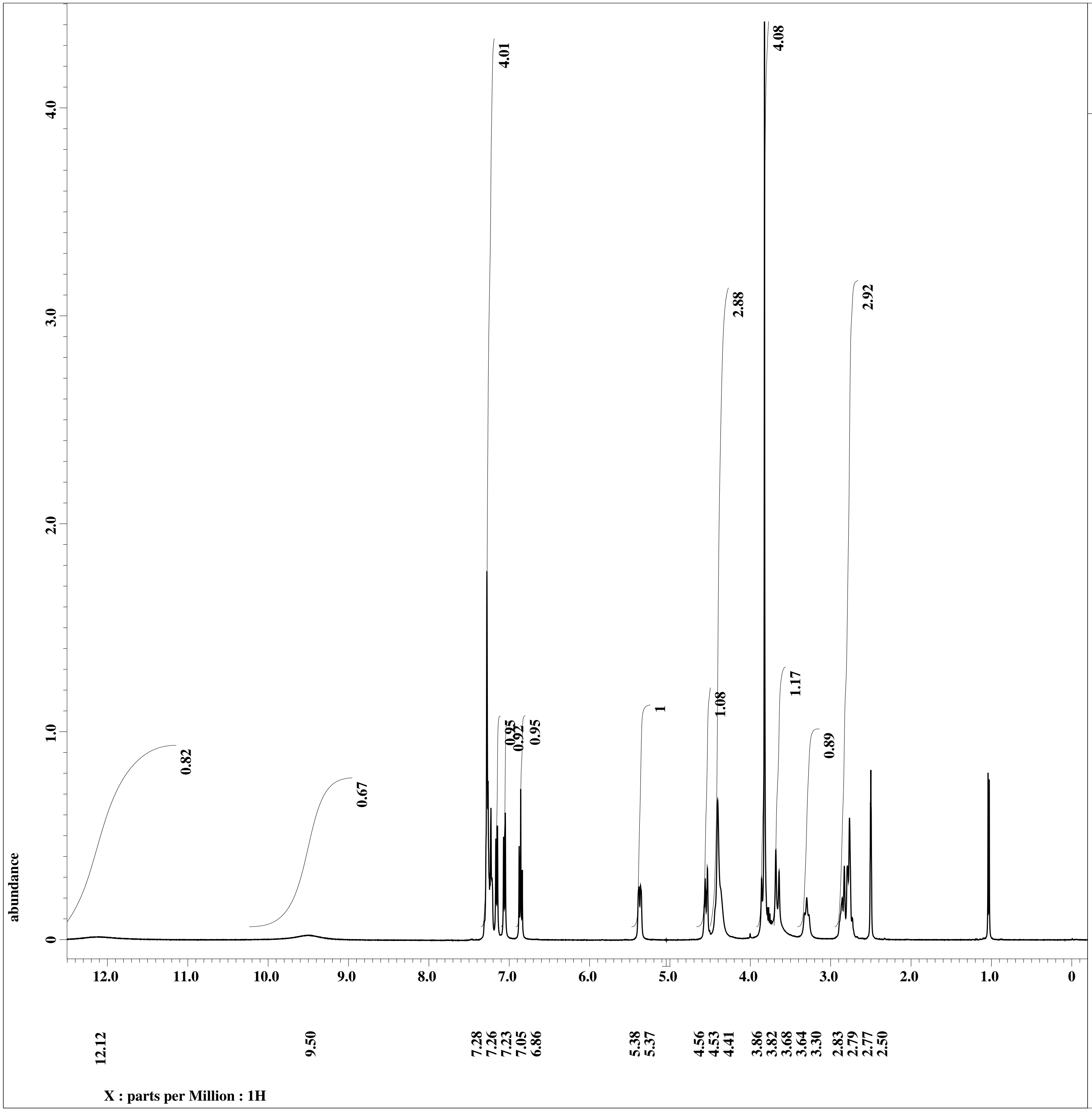

Filename	= BDG-II-091 recrystall
Author	= chemlabs
Experiment	= single_pulse.ex2
Sample_id	= 001
Solvent	= DMSO-D6
Creation_time	= 24-JUN-2016 11:07:14
Revision_time	= 21-JUL-2021 11:14:38
Current_time	= 21-JUL-2021 11:18:22
Comment	= single_pulse
Data_format	= 1D COMPLEX
Dim_size	= 13107
Dim_title	= 1H
Dim_units	= [ppm]
Dimensions	= X
Site	= ECS 400
Spectrometer	= JNM-ECS400
Field_strength	= 9.389766[T] (400[MHz]
X_acq_duration	= 2.18365952[s]
X_domain	= 1H
X_freq	= 399.78219838[MHz]
X_offset	= 5[ppm]
X_points	= 16384
X_prescans	= 1
X_resolution	= 0.45794685[Hz]
X_sweep	= 7.5030012[kHz]
Irr_domain	= 1H
Irr_freq	= 399.78219838[MHz]
Irr_offset	= 5[ppm]
Tri_domain	= 1H
Tri_freq	= 399.78219838[MHz]
Tri_offset	= 5[ppm]
Clipped	= FALSE
Mod_return	= 1
Scans	= 8
Total_scans	= 8
X_90_width	= 12.8[us]
X_acq_time	= 2.18365952[s]
X_angle	= 45[deg]
X_atn	= 3[dB]
X_pulse	= 6.4[us]
Irr_mode	= Off
Tri_mode	= Off
Dante_presat	= FALSE
Initial_wait	= 1[s]
Recvr_gain	= 34
Relaxation_delay	= 5[s]
Repetition_time	= 7.18365952[s]
Temp_get	= 23[dC]

Figure S43: 13C NMR Spectrum of Compound 23.

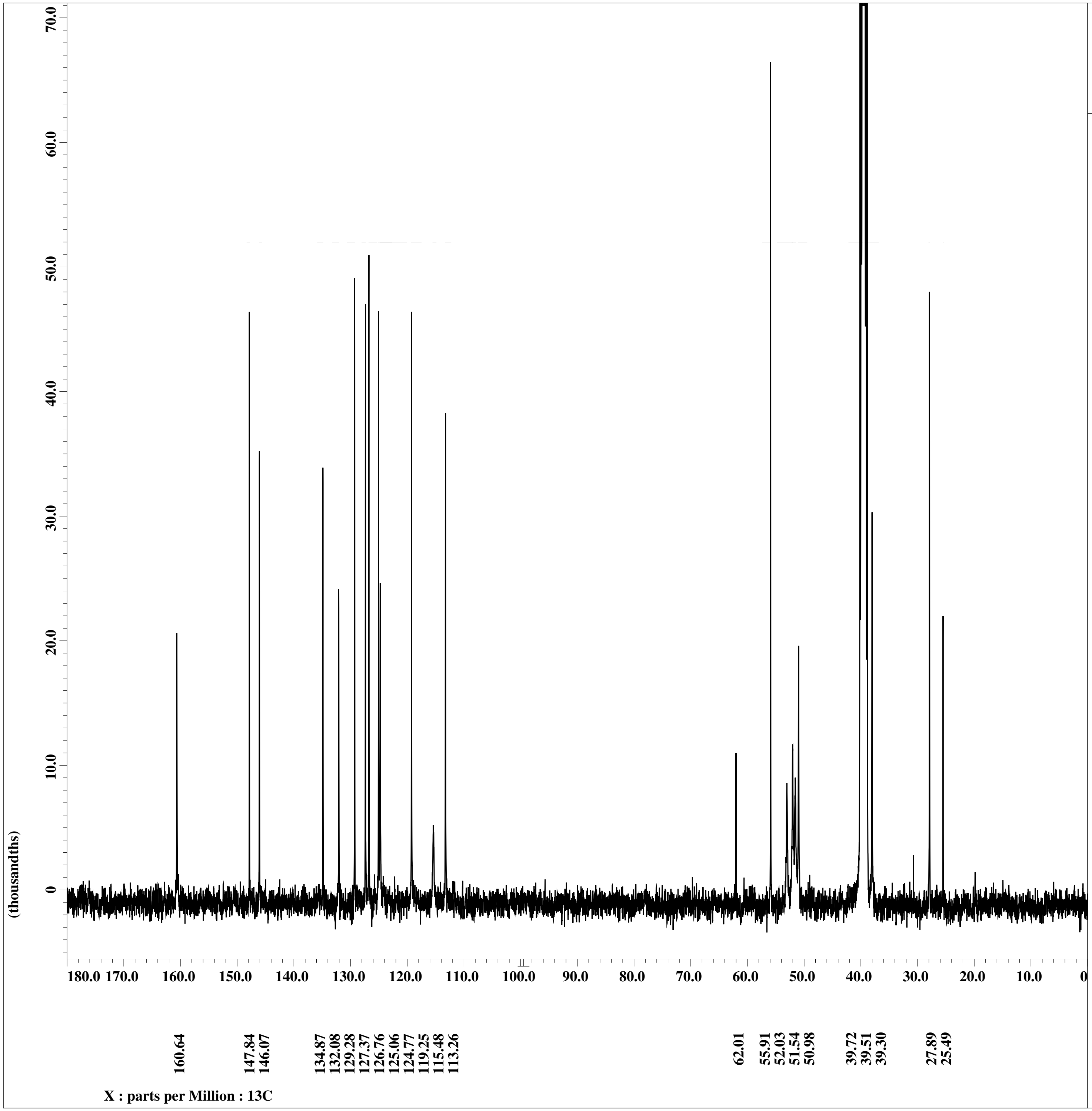


```
Filename          = BDG-II-091 recrystall
Author            = chemlabs
Experiment        = single_pulse_dec
Sample_id         = 001
Solvent           = DMSO-D6
Creation_time     = 30-JUN-2016 00:26:41
Revision_time     = 21-JUL-2021 11:20:41
Current_time      = 21-JUL-2021 11:25:35

Comment           = single pulse decouple
Data_format       = 1D COMPLEX
Dim_size          = 26214
Dim_title         = 13C
Dim_units         = [ppm]
Dimensions        = X
Site              = ECS 400
Spectrometer      = JNM-ECS400

Field_strength    = 9.389766[T] (400[MHz]
X_acq_duration    = 1.04333312[s]
X_domain          = 13C
X_freq            = 100.52530333[MHz]
X_offset          = 100[ppm]
X_points          = 32768
X_prescans        = 4
X_resolution      = 0.95846665[Hz]
X_sweep           = 31.40703518[kHz]
Irr_domain        = 1H
Irr_freq          = 399.78219838[MHz]
Irr_offset        = 5[ppm]
Clipped           = FALSE
Mod_return        = 1
Scans             = 8192
Total_scans       = 8192

X_90_width        = 9.5[us]
X_acq_time        = 1.04333312[s]
X_angle           = 30[deg]
X_atn             = 6[dB]
X_pulse           = 3.16666667[us]
Irr_atn_dec       = 21[dB]
Irr_atn_noe       = 21[dB]
Irr_noise         = WALTZ
Decoupling        = TRUE
Initial_wait      = 1[s]
Noe               = TRUE
Noe_time          = 2[s]
Recvr_gain        = 60
Relaxation_delay  = 2[s]
Repetition_time   = 3.04333312[s]
Temp_get          = 23.3[dC]
```

Figure S44: 1H NMR Spectrum of Compound 24.

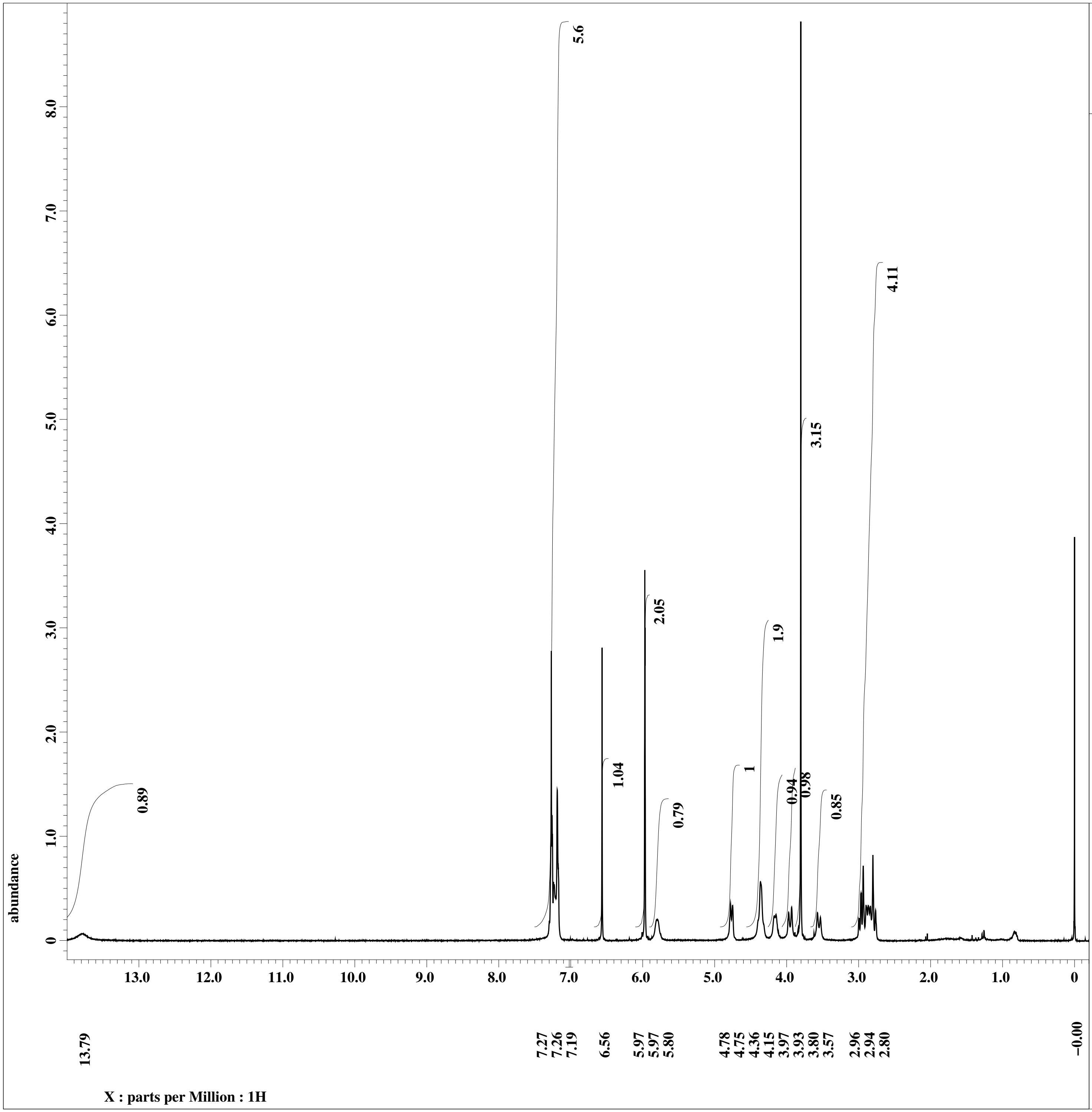


```
Filename          = LSC-1-136_slot6_1H_NM
Author            = russell research stud
Experiment        = single_pulse.ex2
Sample_id         = S#518344
Solvent           = CHLOROFORM-D
Creation_time     = 27-JUL-2021 14:28:14
Revision_time     = 12-AUG-2021 10:24:35
Current_time      = 12-AUG-2021 10:29:42

Data_format       = 1D COMPLEX
Dim_size          = 13107
Dim_title         = 1H
Dim_units         = [ppm]
Dimensions        = X
Site              = ECS 400
Spectrometer      = JNM-ECS400

Field_strength    = 9.389766[T] (400[MHz]
X_acq_duration    = 2.04734464[s]
X_domain          = 1H
X_freq            = 399.78219838[MHz]
X_offset          = 7[ppm]
X_points          = 16384
X_prescans        = 1
X_resolution      = 0.48843755[Hz]
X_sweep           = 8.00256082[kHz]
Irr_domain        = 1H
Irr_freq          = 399.78219838[MHz]
Irr_offset        = 5[ppm]
Tri_domain        = 1H
Tri_freq          = 399.78219838[MHz]
Tri_offset        = 5[ppm]
Clipped           = FALSE
Mod_return        = 1
Scans             = 8
Total_scans       = 8

X_90_width        = 14.3[us]
X_acq_time        = 2.04734464[s]
X_angle           = 45[deg]
X_atn             = 3[dB]
X_pulse           = 7.15[us]
Irr_mode          = Off
Tri_mode          = Off
Dante_presat      = FALSE
Initial_wait      = 1[s]
Recvr_gain        = 46
Relaxation_delay  = 5[s]
Repetition_time   = 7.04734464[s]
Temp_get          = 24.2[dC]
```

Figure S45: 13C NMR Spectrum of Compound 24.

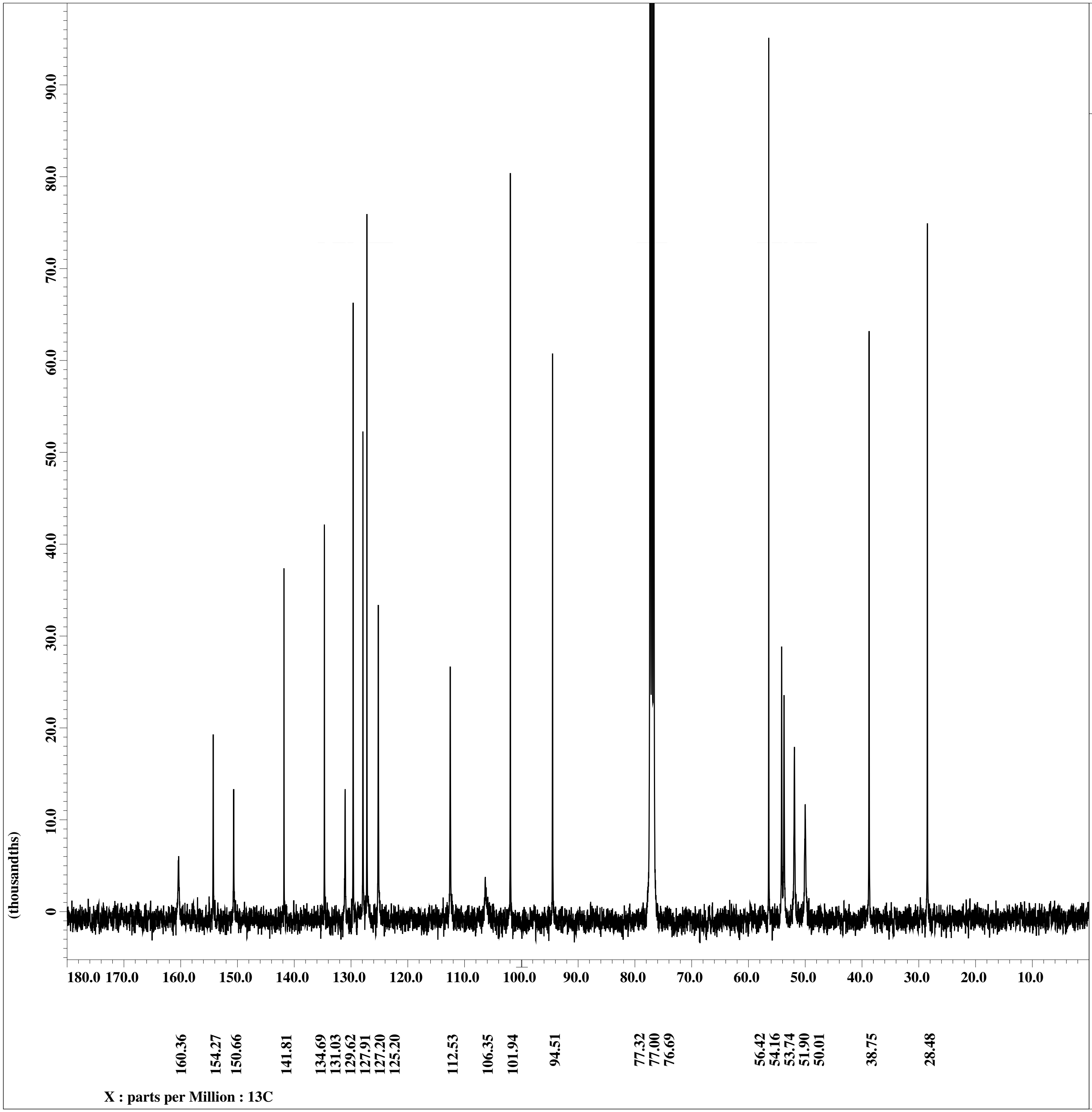

Filename	= LSC-1-136_slot6_13C_N
Author	= russell research stud
Experiment	= single_pulse_dec
Sample_id	= S#519534
Solvent	= CHLOROFORM-D
Creation_time	= 27-JUL-2021 21:24:19
Revision_time	= 12-AUG-2021 10:30:47
Current_time	= 12-AUG-2021 10:35:16
Data_format	= 1D COMPLEX
Dim_size	= 26214
Dim_title	= 13C
Dim_units	= [ppm]
Dimensions	= X
Site	= ECS 400
Spectrometer	= JNM-ECS400
Field_strength	= 9.389766[T] (400[MHz]
X_acq_duration	= 1.04333312[s]
X_domain	= 13C
X_freq	= 100.52530333[MHz]
X_offset	= 100[ppm]
X_points	= 32768
X_prescans	= 4
X_resolution	= 0.95846665[Hz]
X_sweep	= 31.40703518[kHz]
Irr_domain	= 1H
Irr_freq	= 399.78219838[MHz]
Irr_offset	= 5[ppm]
Clipped	= FALSE
Mod_return	= 1
Scans	= 8192
Total_scans	= 8192
X_90_width	= 9.82623[us]
X_acq_time	= 1.04333312[s]
X_angle	= 30[deg]
X_atn	= 5.8[dB]
X_pulse	= 3.27541[us]
Irr_atn_dec	= 21[dB]
Irr_atn_noe	= 21[dB]
Irr_noise	= WALTZ
Decoupling	= TRUE
Initial_wait	= 1[s]
Noe	= TRUE
Noe_time	= 2[s]
Recvr_gain	= 60
Relaxation_delay	= 2[s]
Repetition_time	= 3.04333312[s]
Temp_get	= 24.6[dC]

Figure S46: 1H NMR Spectrum of Compound 25.

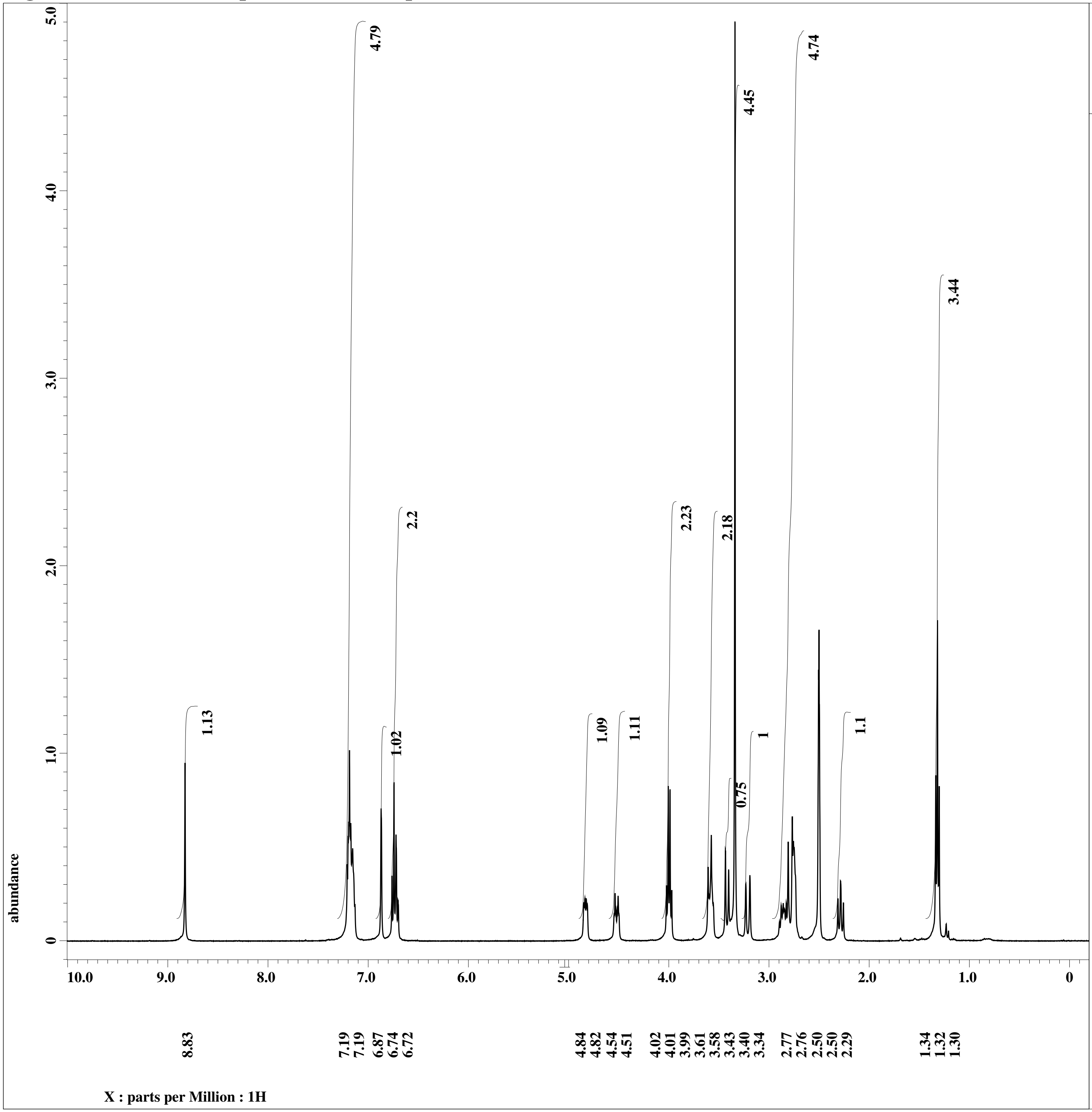

Filename	= BDG-II-115 1H NMR Wor
Author	= chemlabs
Experiment	= single_pulse.ex2
Sample_id	= BDG-II-115
Solvent	= DMSO-D6
Creation_time	= 2-SEP-2019 22:56:05
Revision_time	= 21-JUL-2021 11:51:33
Current_time	= 21-JUL-2021 11:56:22
Data_format	= 1D COMPLEX
Dim_size	= 13107
Dim_title	= 1H
Dim_units	= [ppm]
Dimensions	= X
Site	= ECS 400
Spectrometer	= JNM-ECS400
Field_strength	= 9.389766[T] (400[MHz]
X_acq_duration	= 2.18365952[s]
X_domain	= 1H
X_freq	= 399.78219838[MHz]
X_offset	= 5[ppm]
X_points	= 16384
X_prescans	= 1
X_resolution	= 0.45794685[Hz]
X_sweep	= 7.5030012[kHz]
Irr_domain	= 1H
Irr_freq	= 399.78219838[MHz]
Irr_offset	= 5[ppm]
Tri_domain	= 1H
Tri_freq	= 399.78219838[MHz]
Tri_offset	= 5[ppm]
Clipped	= FALSE
Mod_return	= 1
Scans	= 16
Total_scans	= 16
X_90_width	= 14.3[us]
X_acq_time	= 2.18365952[s]
X_angle	= 45[deg]
X_atn	= 3[dB]
X_pulse	= 7.15[us]
Irr_mode	= Off
Tri_mode	= Off
Dante_presat	= FALSE
Initial_wait	= 1[s]
Recvr_gain	= 40
Relaxation_delay	= 4[s]
Repetition_time	= 6.18365952[s]
Temp_get	= 22.5[dC]

Figure S47: 13C NMR Spectrum of Compound 25.

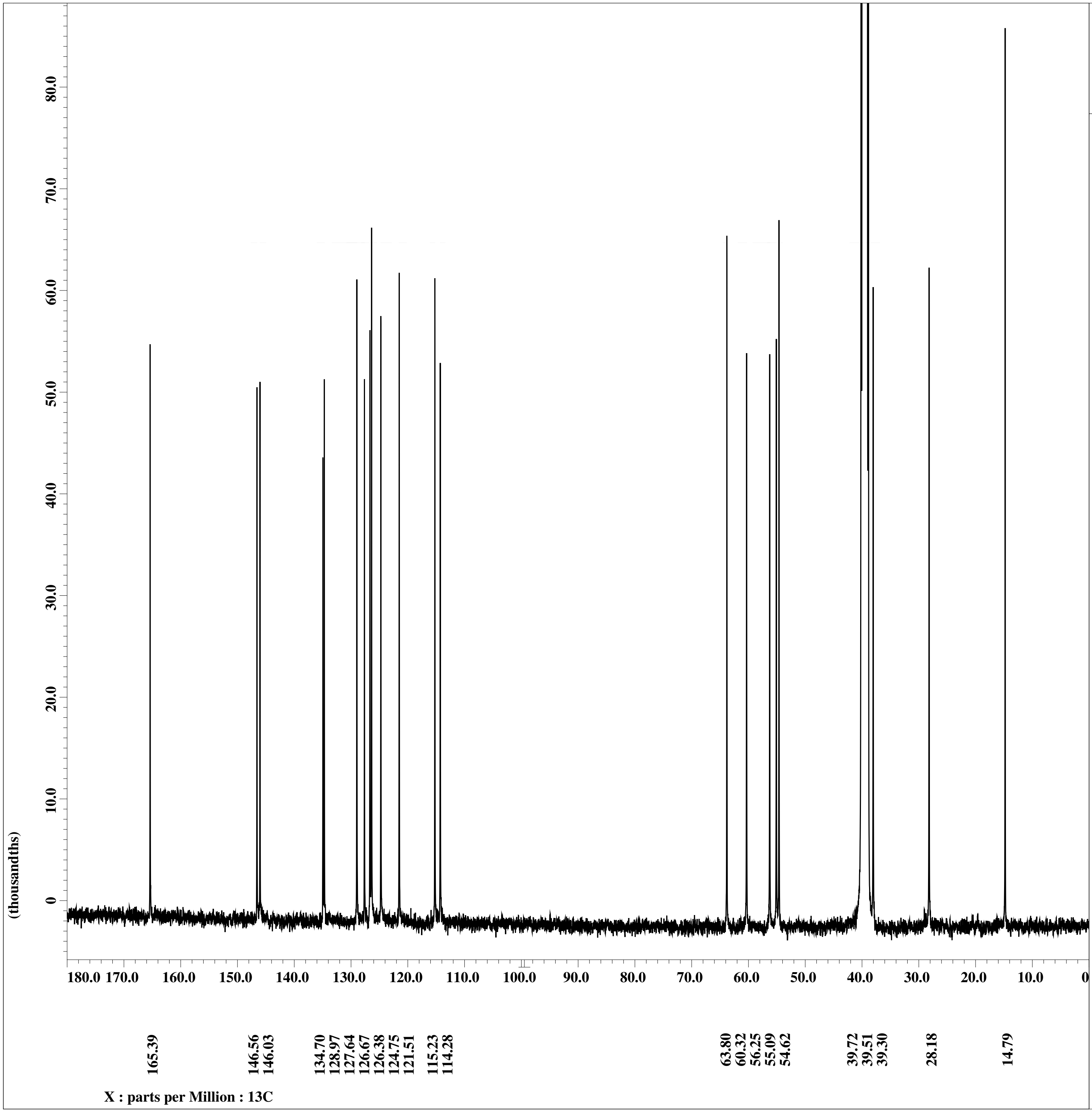

Filename	= BDG-II-115 13C NMR Wo
Author	= chemlabs
Experiment	= single_pulse_dec
Sample_id	= BDG-II-115
Solvent	= DMSO-D6
Creation_time	= 4-SEP-2019 02:38:45
Revision_time	= 21-JUL-2021 11:57:07
Current_time	= 21-JUL-2021 12:00:35
Data_format	= 1D COMPLEX
Dim_size	= 26214
Dim_title	= 13C
Dim_units	= [ppm]
Dimensions	= X
Site	= ECS 400
Spectrometer	= JNM-ECS400
Field_strength	= 9.389766[T] (400[MHz]
X_acq_duration	= 1.04333312[s]
X_domain	= 13C
X_freq	= 100.52530333[MHz]
X_offset	= 100[ppm]
X_points	= 32768
X_prescans	= 4
X_resolution	= 0.95846665[Hz]
X_sweep	= 31.40703518[kHz]
Irr_domain	= 1H
Irr_freq	= 399.78219838[MHz]
Irr_offset	= 5[ppm]
Clipped	= FALSE
Mod_return	= 1
Scans	= 32768
Total_scans	= 32768
X_90_width	= 9.82623[us]
X_acq_time	= 1.04333312[s]
X_angle	= 30[deg]
X_atn	= 5.8[dB]
X_pulse	= 3.27541[us]
Irr_atn_dec	= 21[dB]
Irr_atn_noe	= 21[dB]
Irr_noise	= WALTZ
Decoupling	= TRUE
Initial_wait	= 1[s]
Noe	= TRUE
Noe_time	= 2[s]
Recvr_gain	= 60
Relaxation_delay	= 2[s]
Repetition_time	= 3.04333312[s]
Temp_get	= 22.7[dC]

Figure S48: 1H NMR Spectrum of Compound 26.

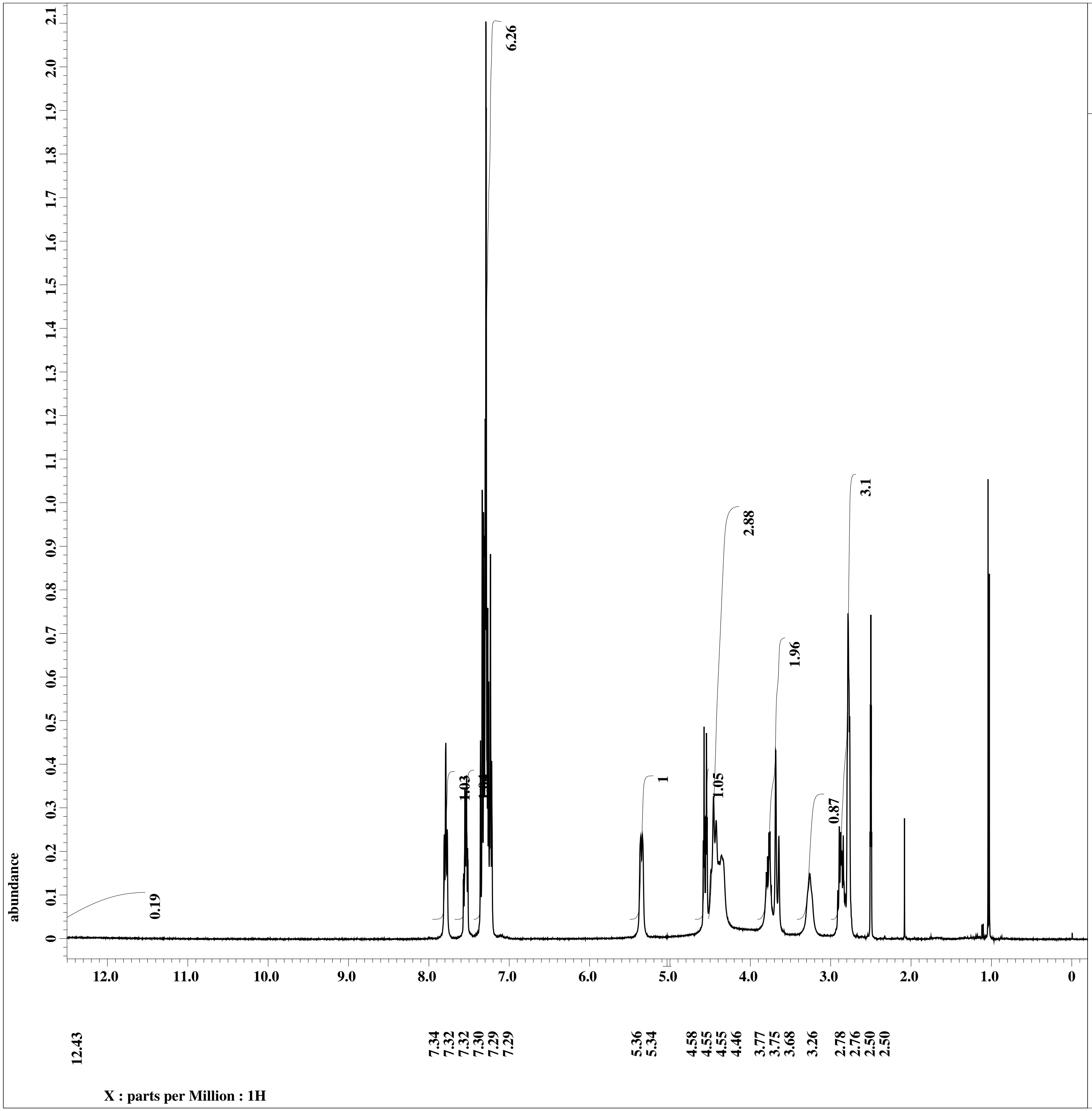


```
Filename          = TAR-2-002 1H NMR-5.jd
Author            = chemlabs
Experiment        = single_pulse.ex2
Sample_id         = S#528192
Solvent           = DMSO-D6
Creation_time     = 12-AUG-2021 14:45:31
Revision_time     = 21-SEP-2021 09:08:49
Current_time      = 21-SEP-2021 09:16:17

Comment           = single_pulse
Data_format       = 1D COMPLEX
Dim_size          = 13107
Dim_title         = 1H
Dim_units         = [ppm]
Dimensions        = X
Site              = ECS 400
Spectrometer      = JNM-ECS400

Field_strength    = 9.389766[T] (400[MHz]
X_acq_duration    = 2.18365952[s]
X_domain          = 1H
X_freq            = 399.78219838[MHz]
X_offset          = 5[ppm]
X_points          = 16384
X_prescans        = 1
X_resolution      = 0.45794685[Hz]
X_sweep           = 7.5030012[kHz]
Irr_domain        = 1H
Irr_freq          = 399.78219838[MHz]
Irr_offset        = 5[ppm]
Tri_domain        = 1H
Tri_freq          = 399.78219838[MHz]
Tri_offset        = 5[ppm]
Clipped           = FALSE
Mod_return        = 1
Scans             = 8
Total_scans       = 8

X_90_width        = 14.3[us]
X_acq_time        = 2.18365952[s]
X_angle           = 45[deg]
X_atn             = 3[dB]
X_pulse           = 7.15[us]
Irr_mode          = Off
Tri_mode          = Off
Dante_presat      = FALSE
Initial_wait      = 1[s]
Recvr_gain        = 36
Relaxation_delay  = 5[s]
Repetition_time   = 7.18365952[s]
Temp_get          = 23.5[dC]
```

Figure S49: 13C NMR Spectrum of Compound 26.

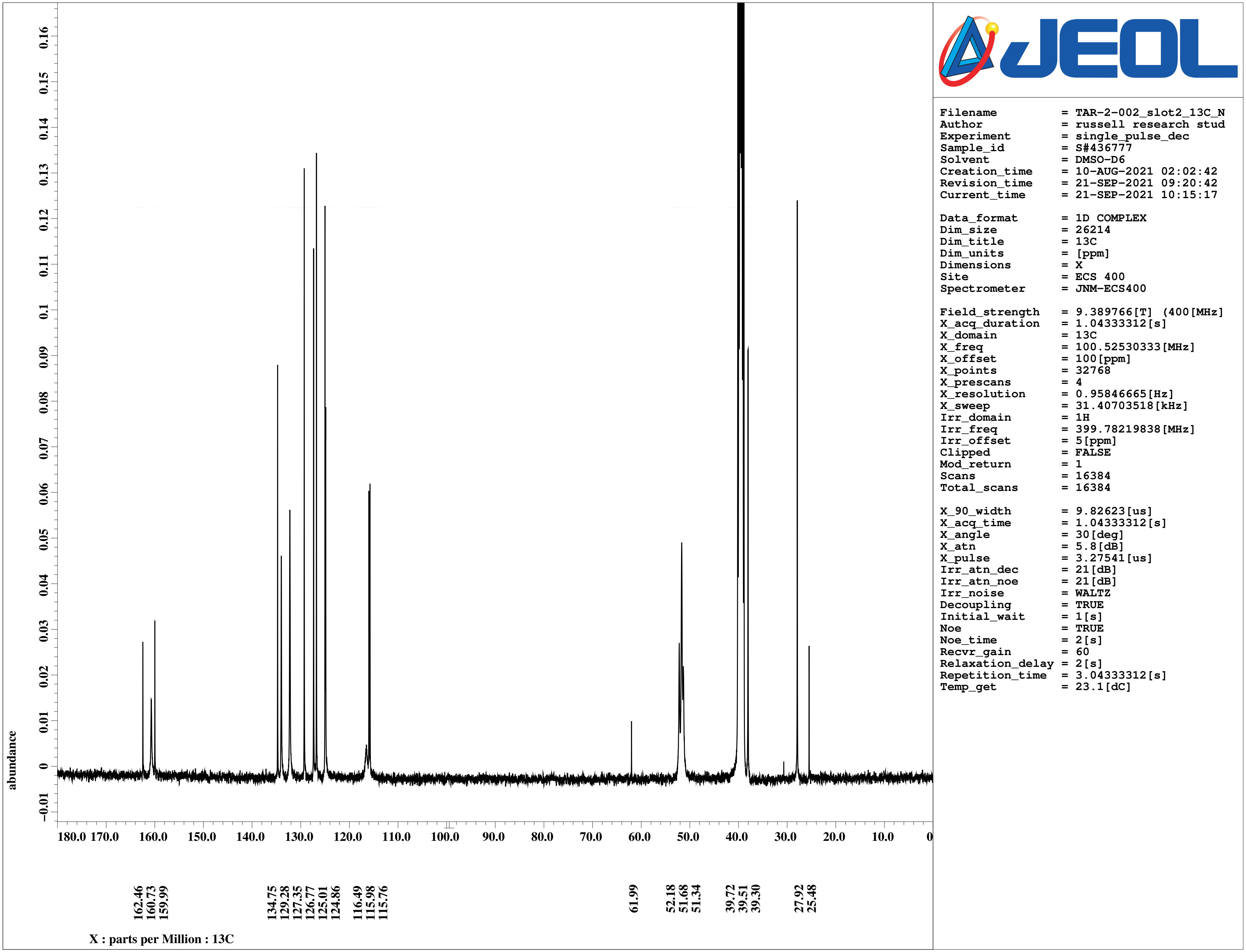

Figure S50: 1H NMR Spectrum of Compound 27.

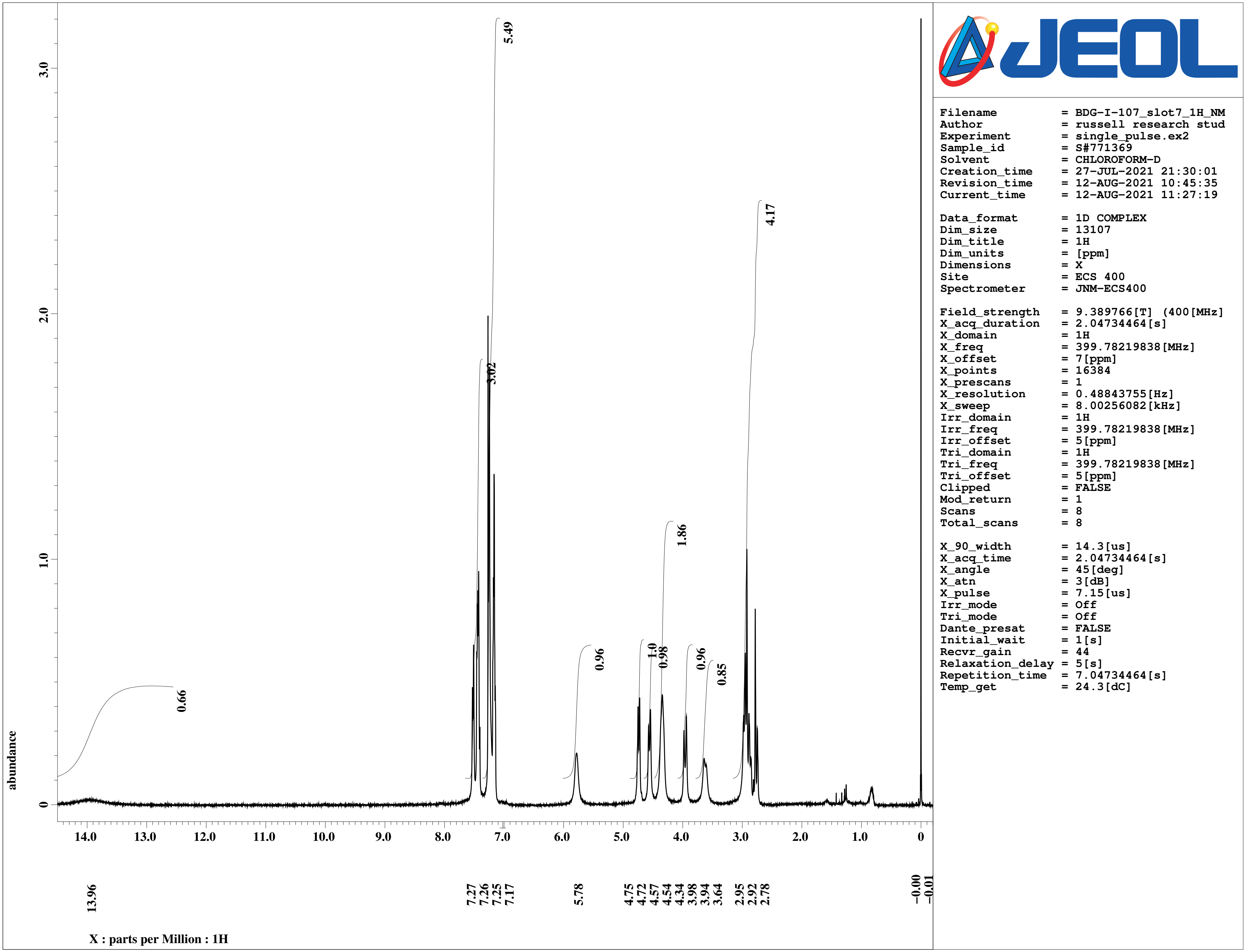

Figure S51: 13C NMR Spectrum of Compound 27.

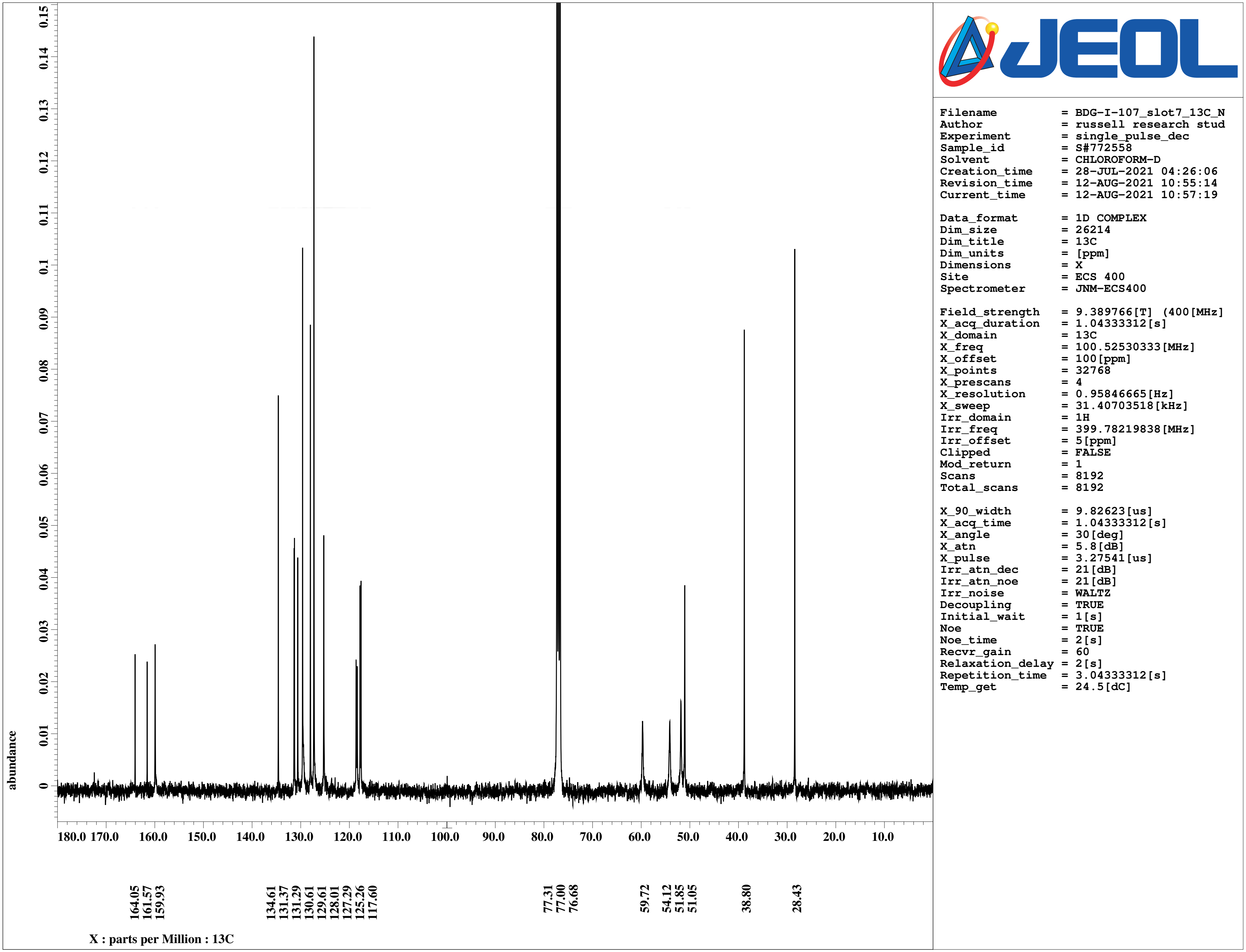

Figure S52: 1H NMR Spectrum of Compound 28.

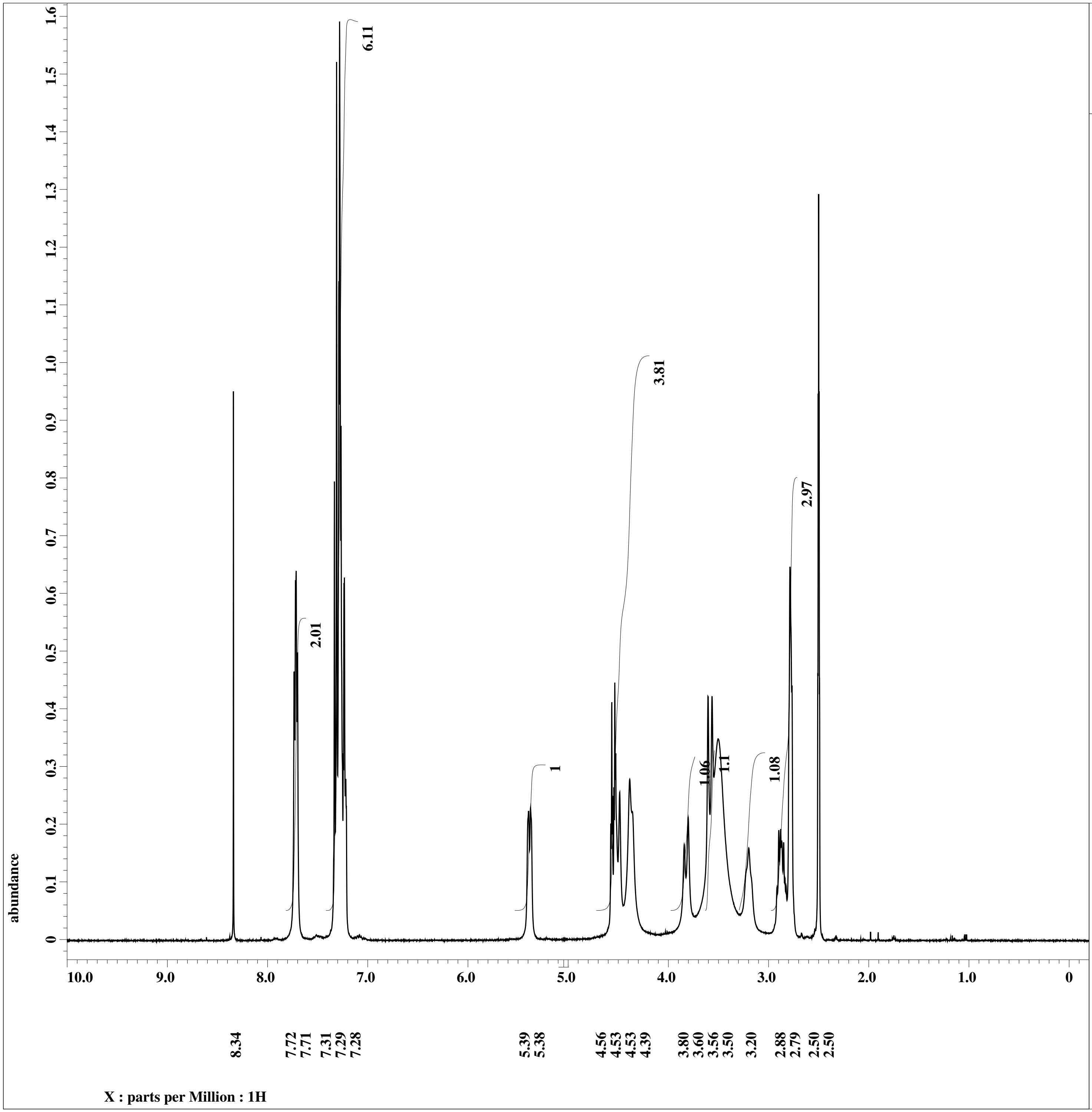


```
Filename            = BDG-II-113 (DMSO)-5.j
Author              = chemlabs
Experiment          = single_pulse.ex2
Sample_id           = 001
Solvent             = DMSO-D6
Creation_time       =  6-JUL-2016 14:57:58
Revision_time       = 21-JUL-2021 13:19:02
Current_time        = 21-JUL-2021 13:19:08

Comment             = single_pulse
Data_format         = 1D COMPLEX
Dim_size            = 13107
Dim_title           = 1H
Dim_units           = [ppm]
Dimensions          = X
Site                = ECS 400
Spectrometer        = JNM-ECS400

Field_strength      = 9.389766[T] (400[MHz]
X_acq_duration      = 2.18365952[s]
X_domain            = 1H
X_freq              = 399.78219838[MHz]
X_offset            = 5[ppm]
X_points            = 16384
X_prescans          = 1
X_resolution        = 0.45794685[Hz]
X_sweep             = 7.5030012[kHz]
Irr_domain          = 1H
Irr_freq            = 399.78219838[MHz]
Irr_offset          = 5[ppm]
Tri_domain          = 1H
Tri_freq            = 399.78219838[MHz]
Tri_offset          = 5[ppm]
Clipped             = FALSE
Mod_return          = 1
Scans               = 8
Total_scans         = 8

X_90_width          = 12.8[us]
X_acq_time          = 2.18365952[s]
X_angle             = 45[deg]
X_atn               = 3[dB]
X_pulse             = 6.4[us]
Irr_mode            = Off
Tri_mode            = Off
Dante_presat        = FALSE
Initial_wait        = 1[s]
Recvr_gain          = 36
Relaxation_delay    = 5[s]
Repetition_time     = 7.18365952[s]
Temp_get            = 22.2[dC]
```

Figure S53: 13C NMR Spectrum of Compound 28.

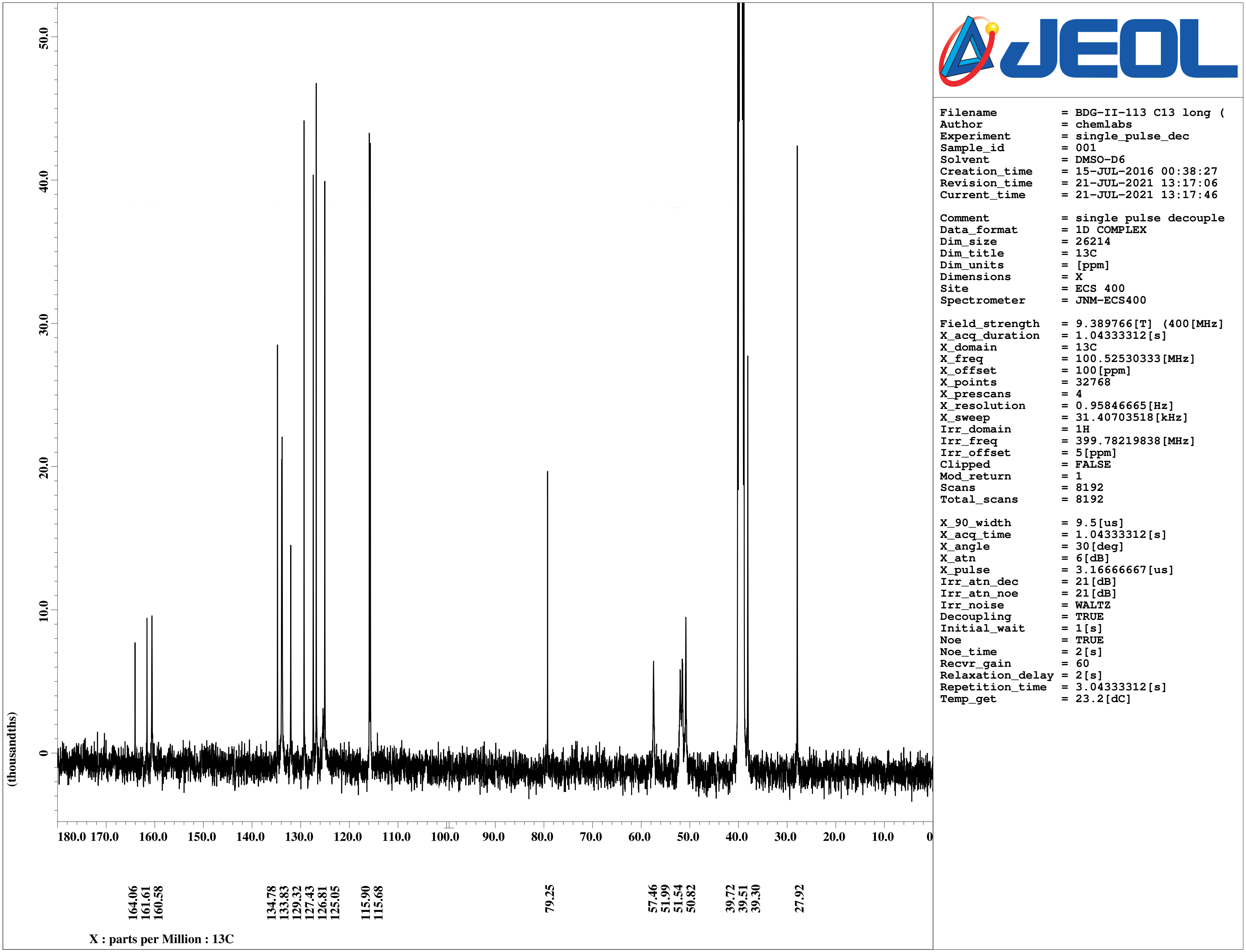

Figure S54: 1H NMR Spectrum of Compound 29.

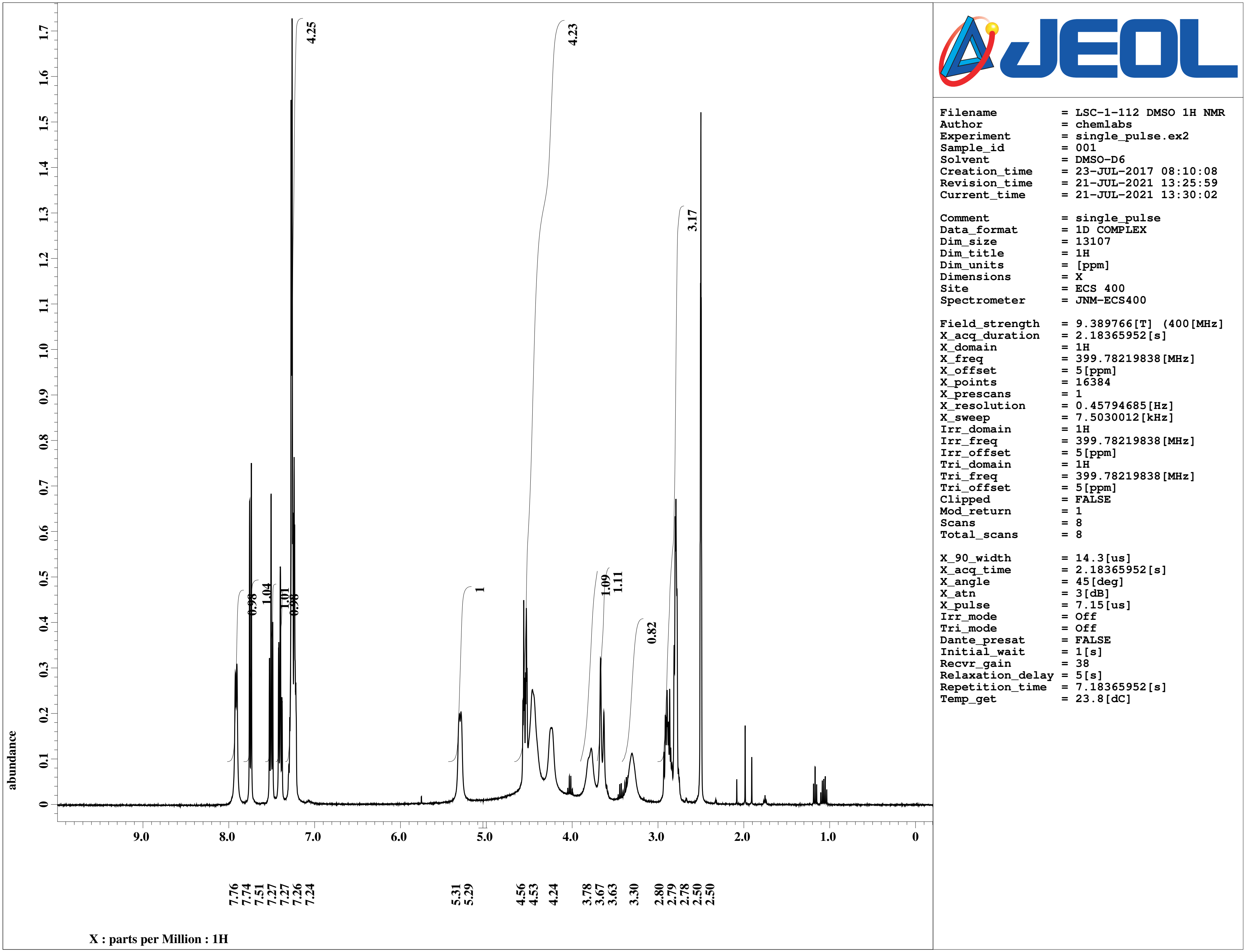

Figure S55: 13C NMR Spectrum of Compound 29.

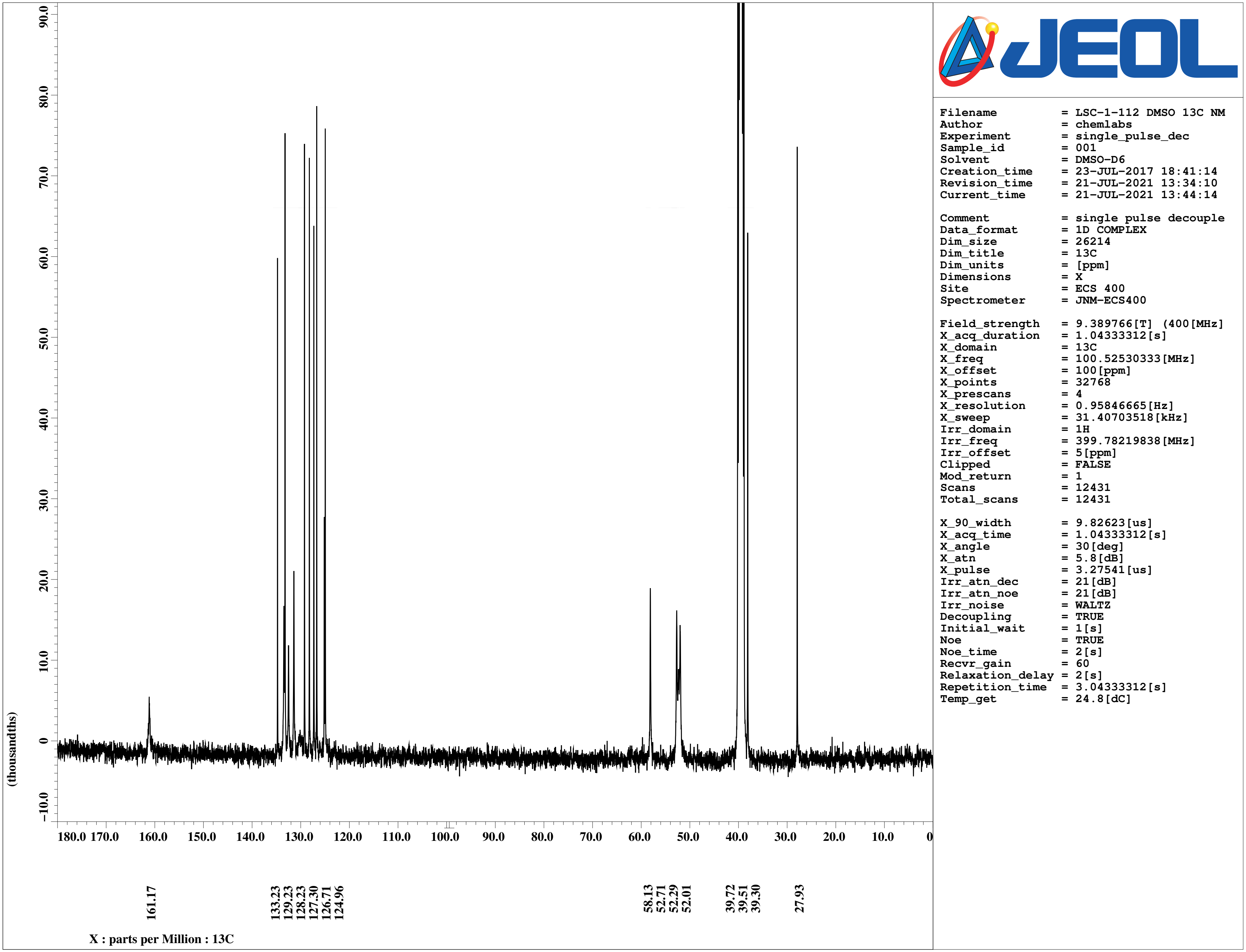

Figure S56: 1H NMR Spectrum of Compound 30.

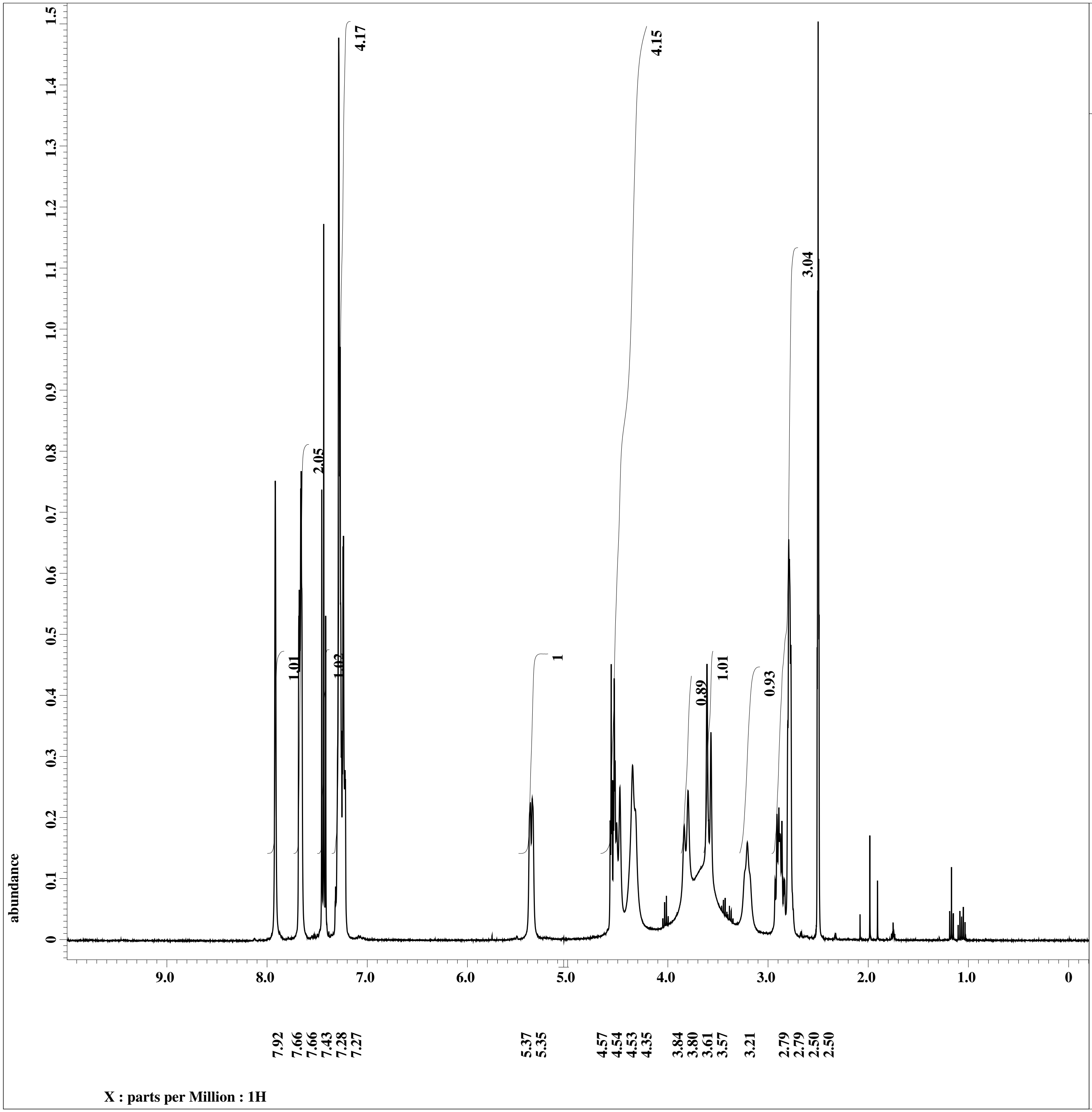


```
Filename          = LSC-1-114 DMSO 1H NMR
Author            = chemlabs
Experiment        = single_pulse.ex2
Sample_id         = 001
Solvent           = DMSO-D6
Creation_time     = 21-JUL-2017 15:55:19
Revision_time     = 21-JUL-2021 13:51:46
Current_time      = 21-JUL-2021 13:52:57

Comment           = single_pulse
Data_format       = 1D COMPLEX
Dim_size          = 13107
Dim_title         = 1H
Dim_units         = [ppm]
Dimensions        = X
Site              = ECS 400
Spectrometer      = JNM-ECS400

Field_strength    = 9.389766[T] (400[MHz]
X_acq_duration    = 2.18365952[s]
X_domain          = 1H
X_freq            = 399.78219838[MHz]
X_offset          = 5[ppm]
X_points          = 16384
X_prescans        = 1
X_resolution      = 0.45794685[Hz]
X_sweep           = 7.5030012[kHz]
Irr_domain        = 1H
Irr_freq          = 399.78219838[MHz]
Irr_offset        = 5[ppm]
Tri_domain        = 1H
Tri_freq          = 399.78219838[MHz]
Tri_offset        = 5[ppm]
Clipped           = FALSE
Mod_return        = 1
Scans             = 8
Total_scans       = 8

X_90_width        = 14.3[us]
X_acq_time        = 2.18365952[s]
X_angle           = 45[deg]
X_atn             = 3[dB]
X_pulse           = 7.15[us]
Irr_mode          = Off
Tri_mode          = Off
Dante_presat      = FALSE
Initial_wait      = 1[s]
Recvr_gain        = 36
Relaxation_delay  = 5[s]
Repetition_time   = 7.18365952[s]
Temp_get          = 23.7[dC]
```

Figure S57: 13C NMR Spectrum of Compound 30.

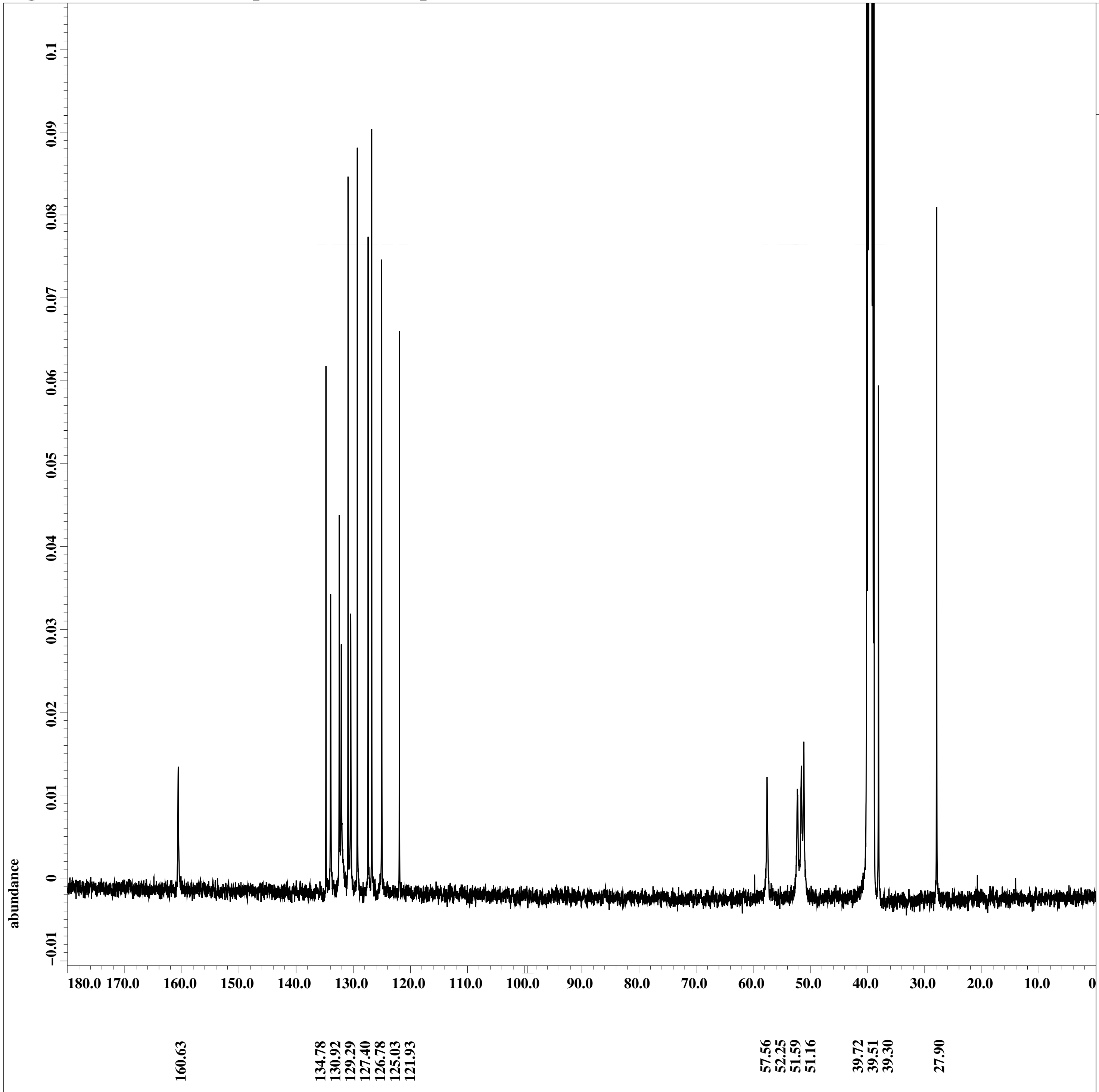

JEOL

```
Filename          = LSC-1-114 DMSO 13C NM
Author            = chemlabs
Experiment        = single_pulse_dec
Sample_id         = 001
Solvent           = DMSO-D6
Creation_time     = 22-JUL-2017 05:47:29
Revision_time     = 21-JUL-2021 13:56:12
Current_time      = 21-JUL-2021 13:57:13

Comment           = single pulse decouple
Data_format       = 1D COMPLEX
Dim_size          = 26214
Dim_title         = 13C
Dim_units         = [ppm]
Dimensions        = X
Site              = ECS 400
Spectrometer      = JNM-ECS400

Field_strength    = 9.389766[T] (400[MHz]
X_acq_duration    = 1.04333312[s]
X_domain          = 13C
X_freq            = 100.52530333[MHz]
X_offset          = 100[ppm]
X_points          = 32768
X_prescans        = 4
X_resolution      = 0.95846665[Hz]
X_sweep           = 31.40703518[kHz]
Irr_domain        = 1H
Irr_freq          = 399.78219838[MHz]
Irr_offset        = 5[ppm]
Clipped           = TRUE
Mod_return        = 1
Scans             = 16384
Total_scans       = 16384

X_90_width        = 9.82623[us]
X_acq_time        = 1.04333312[s]
X_angle           = 30[deg]
X_atn             = 5.8[dB]
X_pulse           = 3.27541[us]
Irr_atn_dec       = 21[dB]
Irr_atn_noe       = 21[dB]
Irr_noise         = WALTZ
Decoupling        = TRUE
Initial_wait      = 1[s]
Noe               = TRUE
Noe_time          = 2[s]
Recvr_gain        = 60
Relaxation_delay  = 2[s]
Repetition_time   = 3.04333312[s]
Temp_get          = 24.1[dC]
```

Figure S58: 1H NMR Spectrum of Compound 31.

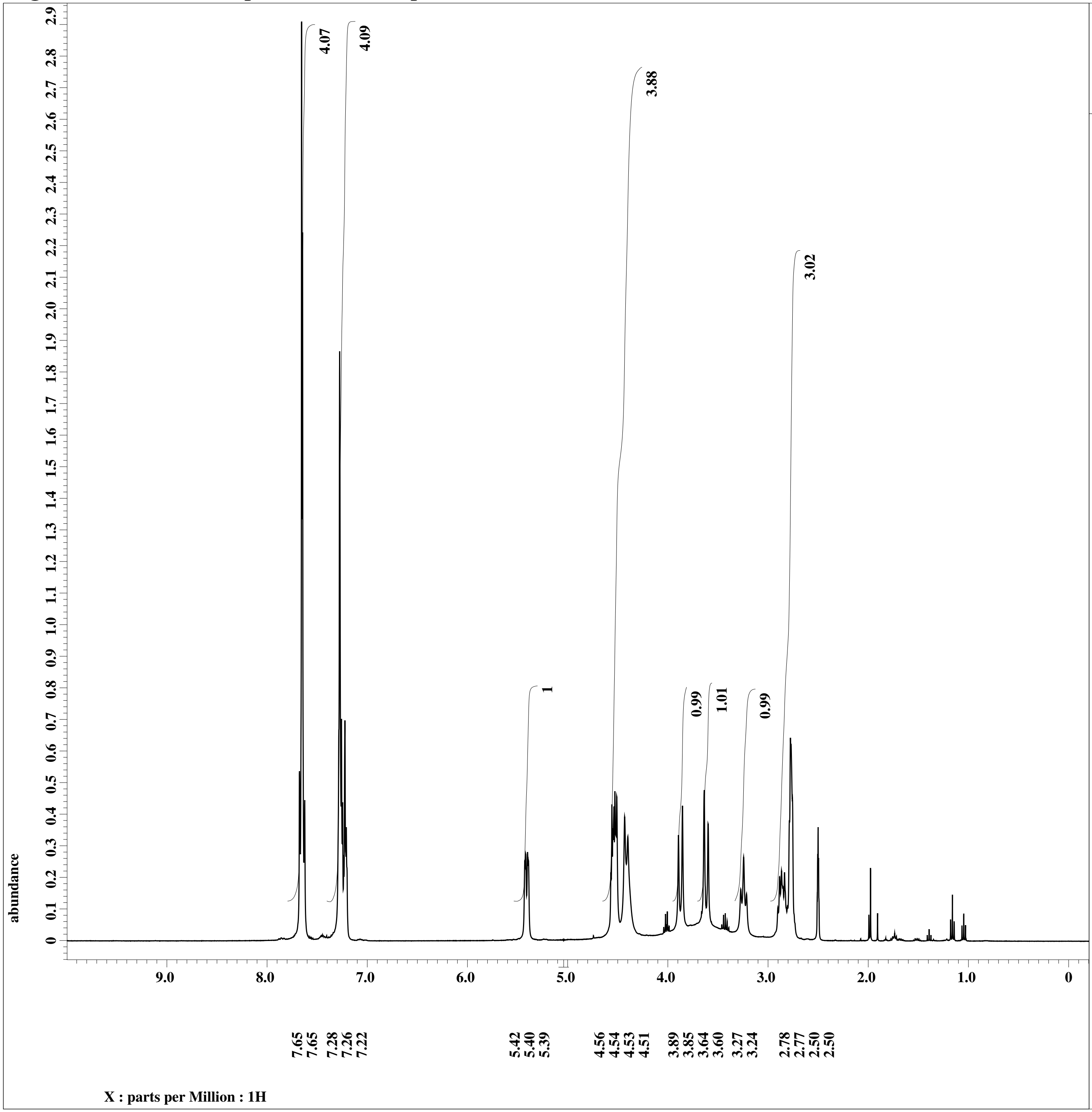


```
Filename          = LSC-1-116 DMSO 1H-4.j
Author            = chemlabs
Experiment        = single_pulse.ex2
Sample_id         = 001
Solvent           = DMSO-D6
Creation_time     = 28-JUL-2017 07:27:31
Revision_time     = 21-JUL-2021 14:01:09
Current_time      = 21-JUL-2021 14:58:02

Comment           = single_pulse
Data_format       = 1D COMPLEX
Dim_size          = 13107
Dim_title         = 1H
Dim_units         = [ppm]
Dimensions        = X
Site              = ECS 400
Spectrometer      = JNM-ECS400

Field_strength    = 9.389766[T] (400[MHz]
X_acq_duration    = 2.18365952[s]
X_domain          = 1H
X_freq            = 399.78219838[MHz]
X_offset          = 5[ppm]
X_points          = 16384
X_prescans        = 1
X_resolution      = 0.45794685[Hz]
X_sweep           = 7.5030012[kHz]
Irr_domain        = 1H
Irr_freq          = 399.78219838[MHz]
Irr_offset        = 5[ppm]
Tri_domain        = 1H
Tri_freq          = 399.78219838[MHz]
Tri_offset        = 5[ppm]
Clipped           = FALSE
Mod_return        = 1
Scans             = 8
Total_scans       = 8

X_90_width        = 14.3[us]
X_acq_time        = 2.18365952[s]
X_angle           = 45[deg]
X_atn             = 3[dB]
X_pulse           = 7.15[us]
Irr_mode          = Off
Tri_mode          = Off
Dante_presat      = FALSE
Initial_wait      = 1[s]
Recvr_gain        = 26
Relaxation_delay  = 5[s]
Repetition_time   = 7.18365952[s]
Temp_get          = 23.5[dC]
```

Figure S59: 13C NMR Spectrum of Compound 31.

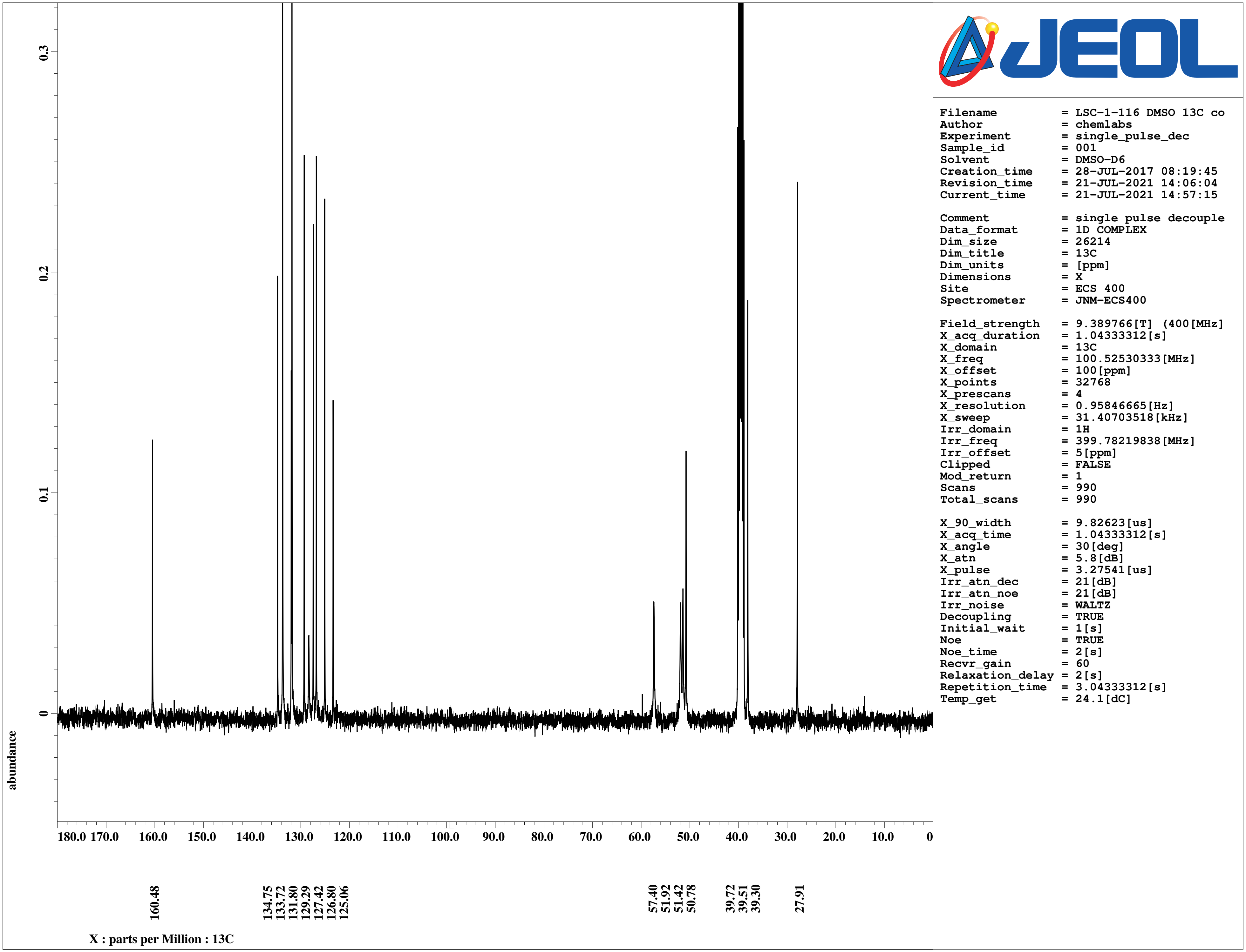

Figure S60: 1H NMR Spectrum of Compound 32.

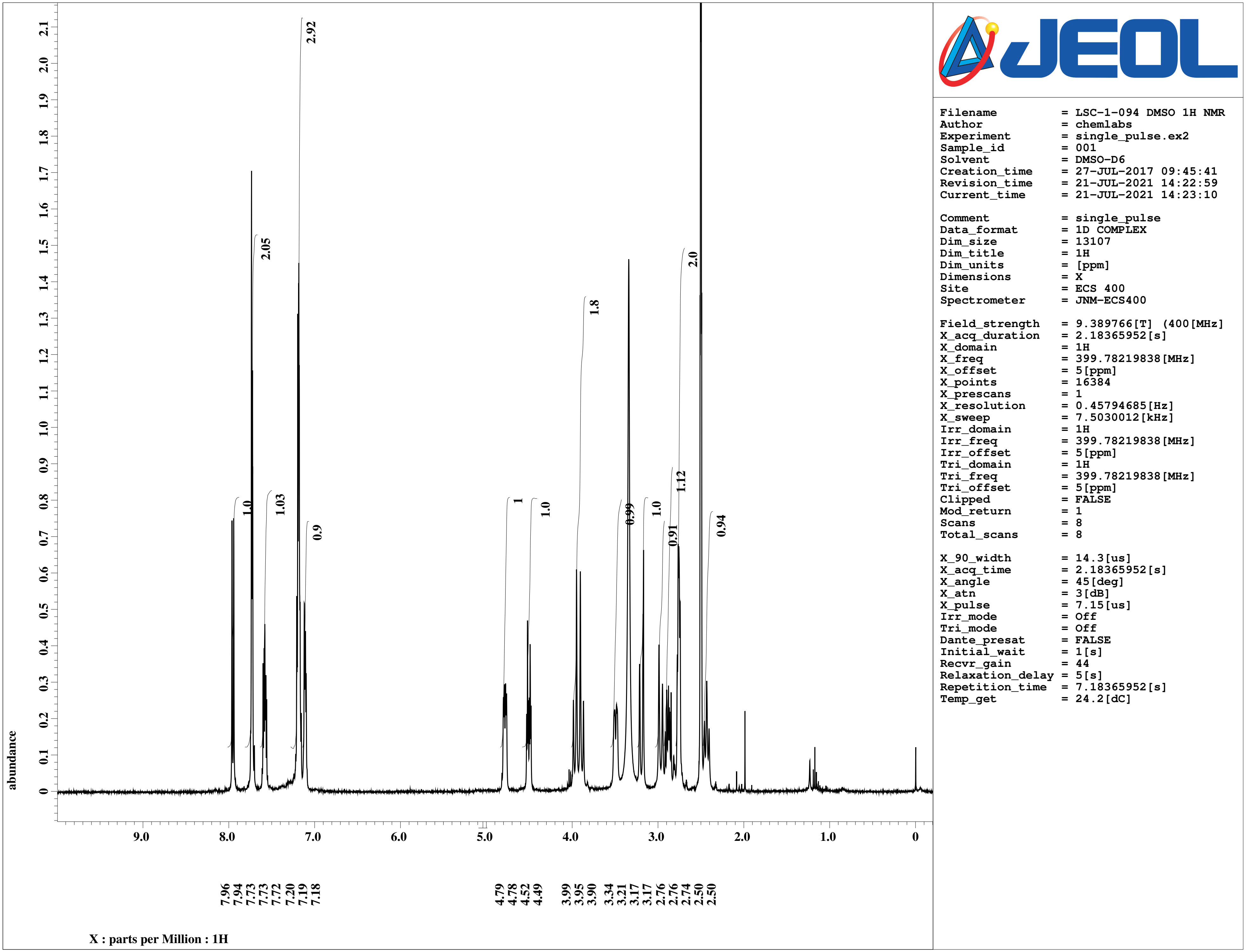

Figure S61: 13C NMR Spectrum of Compound 32.

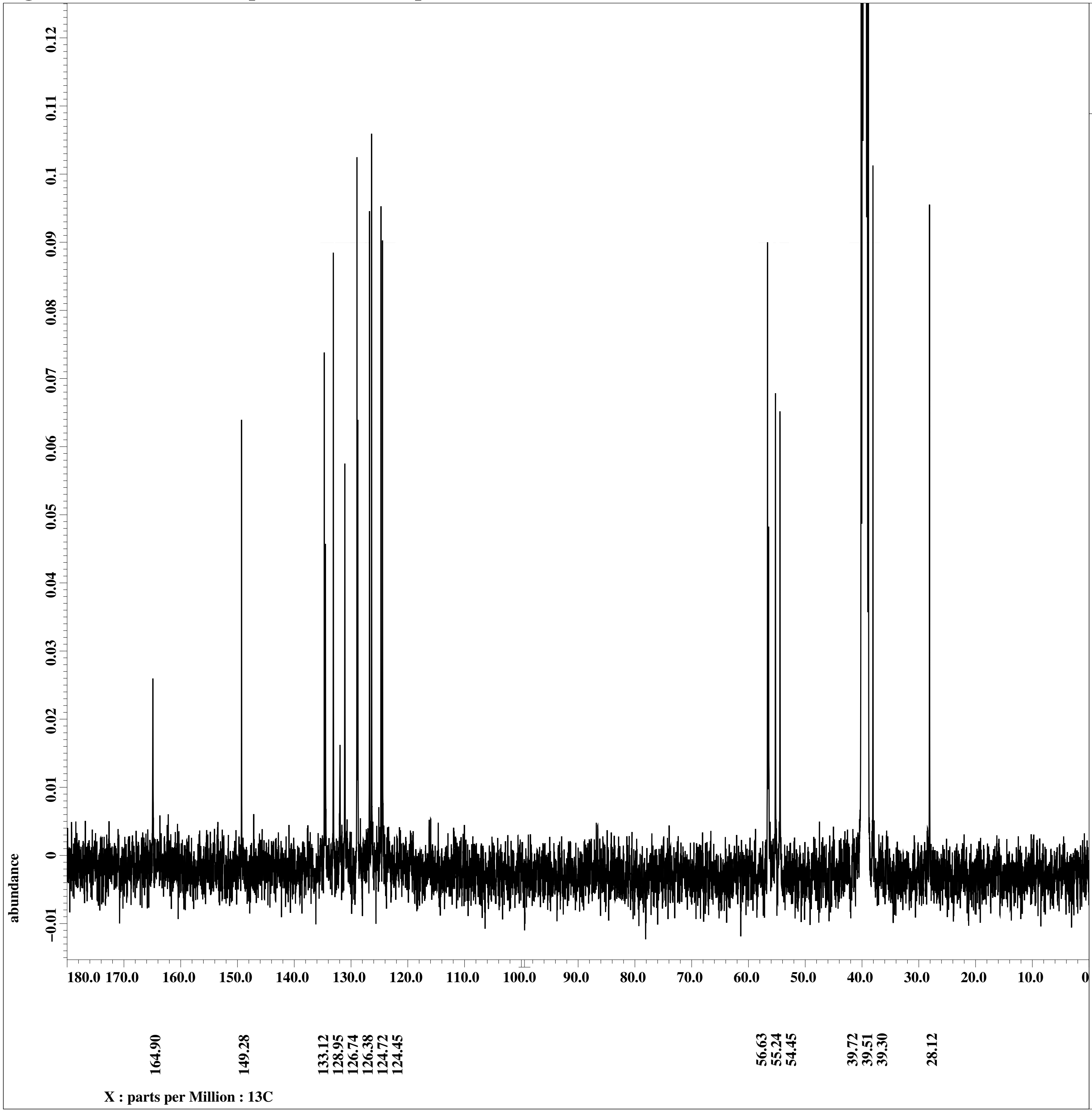


```
Filename          = LSC-1-094 DMSO 1H-5.j
Author            = chemlabs
Experiment        = single_pulse_dec
Sample_id         = 001
Solvent           = DMSO-D6
Creation_time     = 27-JUL-2017 13:42:20
Revision_time     = 21-JUL-2021 14:24:20
Current_time      = 21-JUL-2021 14:30:22

Comment           = single pulse decouple
Data_format       = 1D COMPLEX
Dim_size          = 26214
Dim_title         = 13C
Dim_units         = [ppm]
Dimensions        = X
Site              = ECS 400
Spectrometer      = JNM-ECS400

Field_strength    = 9.389766[T] (400[MHz]
X_acq_duration    = 1.04333312[s]
X_domain          = 13C
X_freq            = 100.52530333[MHz]
X_offset          = 100[ppm]
X_points          = 32768
X_prescans        = 4
X_resolution      = 0.95846665[Hz]
X_sweep           = 31.40703518[kHz]
Irr_domain        = 1H
Irr_freq          = 399.78219838[MHz]
Irr_offset        = 5[ppm]
Clipped           = FALSE
Mod_return        = 1
Scans             = 712
Total_scans       = 712

X_90_width        = 9.82623[us]
X_acq_time        = 1.04333312[s]
X_angle           = 30[deg]
X_atn             = 5.8[dB]
X_pulse           = 3.27541[us]
Irr_atn_dec       = 21[dB]
Irr_atn_noe       = 21[dB]
Irr_noise         = WALTZ
Decoupling        = TRUE
Initial_wait      = 1[s]
Noe               = TRUE
Noe_time          = 2[s]
Recvr_gain        = 60
Relaxation_delay  = 2[s]
Repetition_time   = 3.04333312[s]
Temp_get          = 25.4[dC]
```

Figure S62: 1H NMR Spectrum of Compound 33.

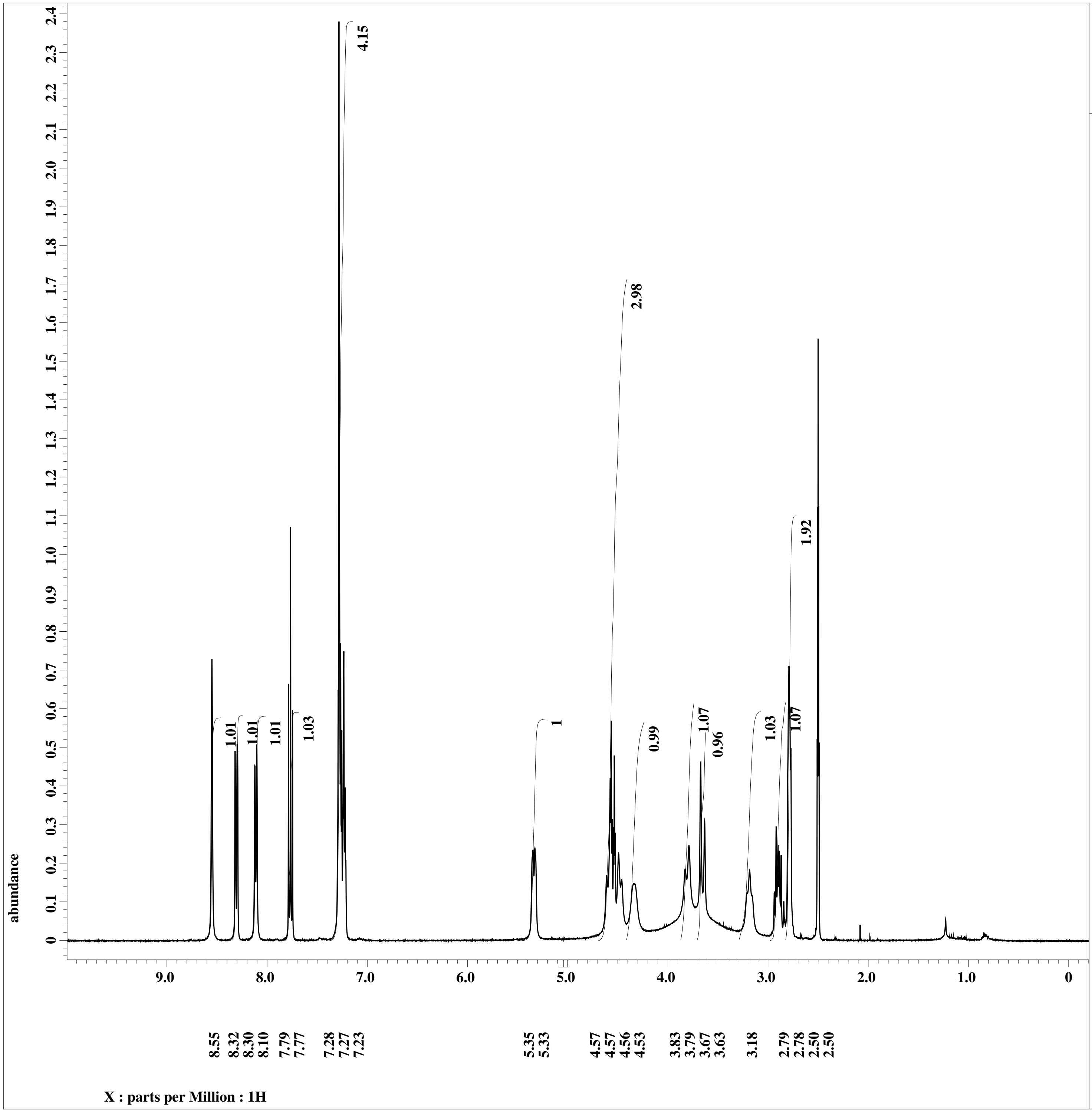

Filename	= LSC-1-061 DMSO 1H NMR
Author	= chemlabs
Experiment	= single_pulse.ex2
Sample_id	= 001
Solvent	= DMSO-D6
Creation_time	= 25-JUL-2017 07:32:00
Revision_time	= 21-JUL-2021 14:36:35
Current_time	= 21-JUL-2021 14:38:37
Comment	= single_pulse
Data_format	= 1D COMPLEX
Dim_size	= 13107
Dim_title	= 1H
Dim_units	= [ppm]
Dimensions	= X
Site	= ECS 400
Spectrometer	= JNM-ECS400
Field_strength	= 9.389766[T] (400[MHz]
X_acq_duration	= 2.18365952[s]
X_domain	= 1H
X_freq	= 399.78219838[MHz]
X_offset	= 5[ppm]
X_points	= 16384
X_prescans	= 1
X_resolution	= 0.45794685[Hz]
X_sweep	= 7.5030012[kHz]
Irr_domain	= 1H
Irr_freq	= 399.78219838[MHz]
Irr_offset	= 5[ppm]
Tri_domain	= 1H
Tri_freq	= 399.78219838[MHz]
Tri_offset	= 5[ppm]
Clipped	= FALSE
Mod_return	= 1
Scans	= 8
Total_scans	= 8
X_90_width	= 14.3[us]
X_acq_time	= 2.18365952[s]
X_angle	= 45[deg]
X_atn	= 3[dB]
X_pulse	= 7.15[us]
Irr_mode	= Off
Tri_mode	= Off
Dante_presat	= FALSE
Initial_wait	= 1[s]
Recvr_gain	= 36
Relaxation_delay	= 5[s]
Repetition_time	= 7.18365952[s]
Temp_get	= 24.9[dC]

Figure S63: 13C NMR Spectrum of Compound 33.

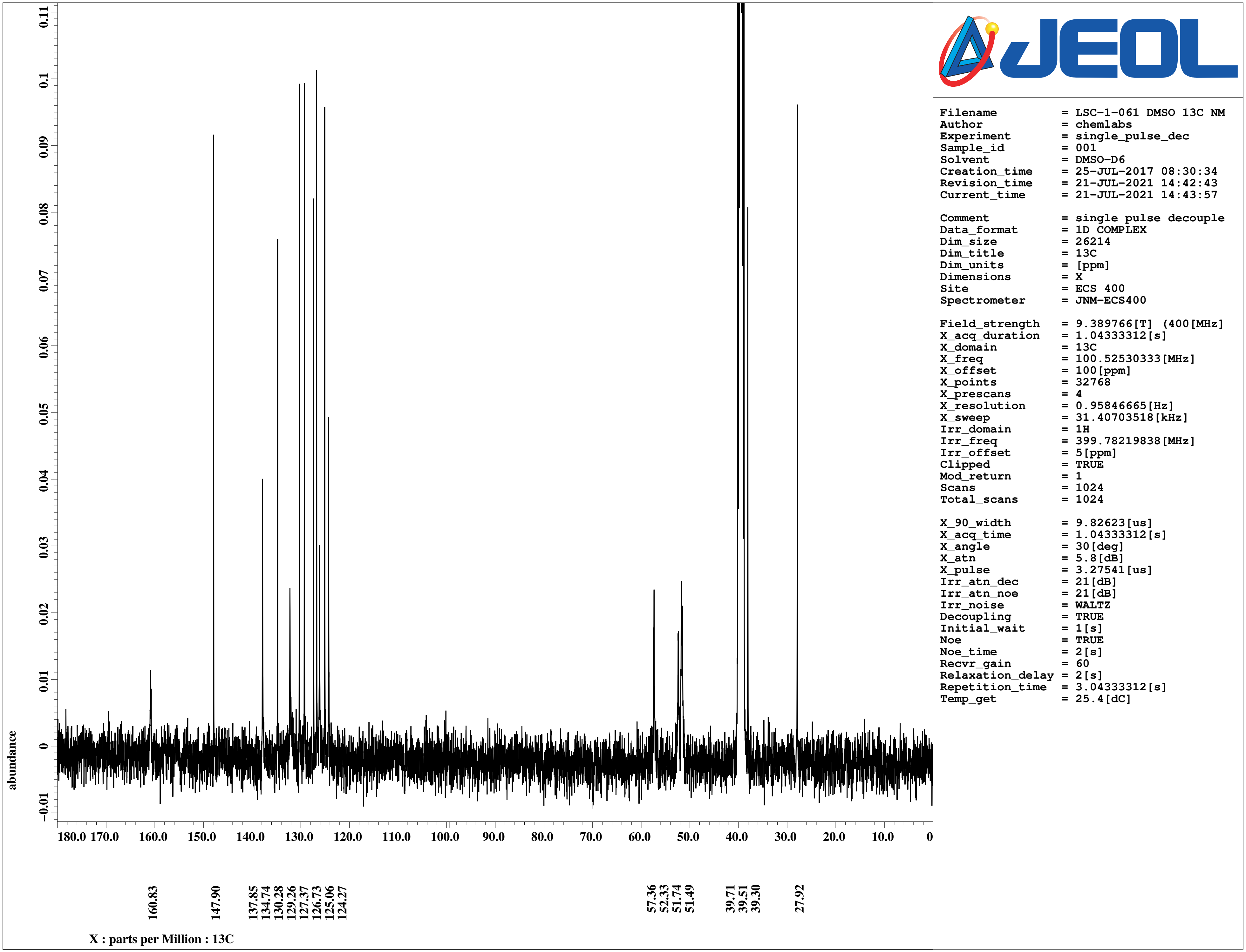

Figure S64: 1H NMR Spectrum of Compound 34.

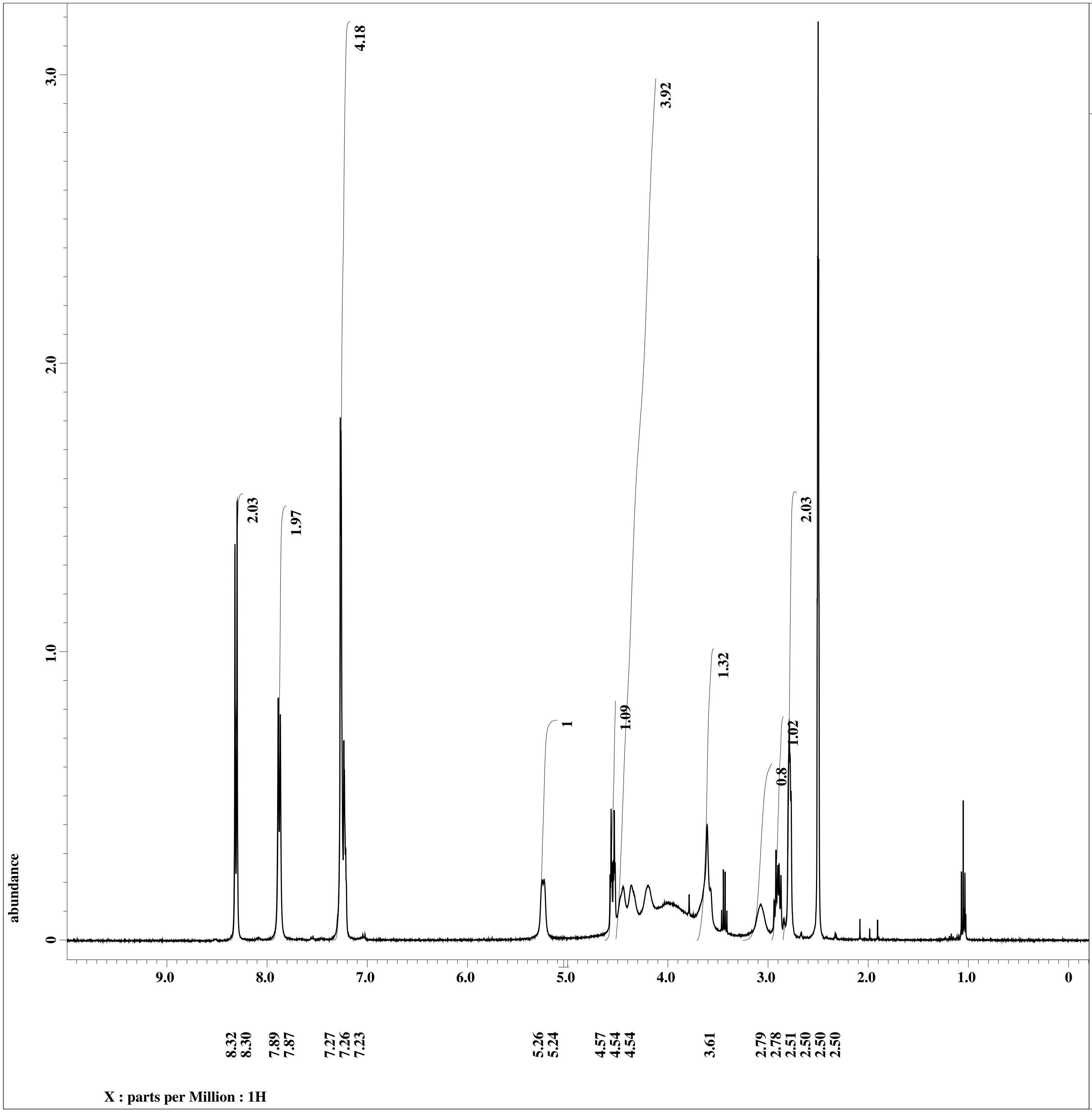

Filename	=	LSC-1-067 DMSO 1H NMR
Author	=	chemlabs
Experiment	=	single_pulse.ex2
Sample_id	=	001
Solvent	=	DMSO-D6
Creation_time	=	26-JUL-2017 08:49:37
Revision_time	=	21-JUL-2021 14:51:41
Current_time	=	21-JUL-2021 14:51:49
Comment	=	single_pulse
Data_format	=	1D COMPLEX
Dim_size	=	13107
Dim_title	=	1H
Dim_units	=	[ppm]
Dimensions	=	X
Site	=	ECS 400
Spectrometer	=	JNM-ECS400
Field_strength	=	9.389766[T] (400[MHz]
X_acq_duration	=	2.18365952[s]
X_domain	=	1H
X_freq	=	399.78219838[MHz]
X_offset	=	5[ppm]
X_points	=	16384
X_prescans	=	1
X_resolution	=	0.45794685[Hz]
X_sweep	=	7.5030012[kHz]
Irr_domain	=	1H
Irr_freq	=	399.78219838[MHz]
Irr_offset	=	5[ppm]
Tri_domain	=	1H
Tri_freq	=	399.78219838[MHz]
Tri_offset	=	5[ppm]
Clipped	=	FALSE
Mod_return	=	1
Scans	=	8
Total_scans	=	8
X_90_width	=	14.3[us]
X_acq_time	=	2.18365952[s]
X_angle	=	45[deg]
X_atn	=	3[dB]
X_pulse	=	7.15[us]
Irr_mode	=	Off
Tri_mode	=	Off
Dante_presat	=	FALSE
Initial_wait	=	1[s]
Recvr_gain	=	44
Relaxation_delay	=	5[s]
Repetition_time	=	7.18365952[s]
Temp_get	=	23.6[dC]

Figure S65: 13C NMR Spectrum of Compound 34.

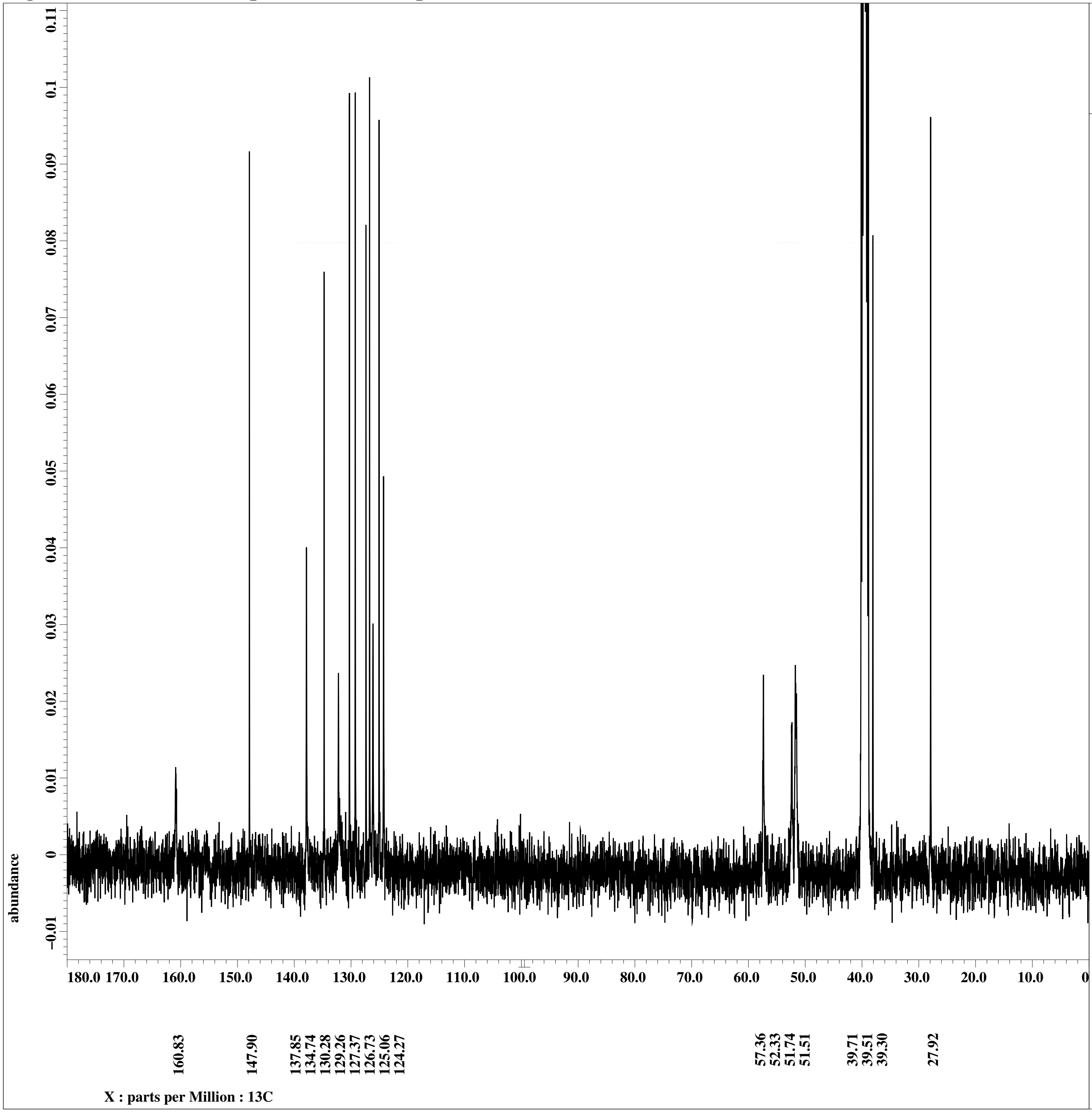


```
Filename          = LSC-1-061 DMSO 13C NM
Author            = chemlabs
Experiment        = single_pulse_dec
Sample_id         = 001
Solvent           = DMSO-D6
Creation_time     = 25-JUL-2017 08:30:34
Revision_time     = 21-JUL-2021 14:54:52
Current_time      = 21-JUL-2021 14:56:02

Comment           = single pulse decouple
Data_format       = 1D COMPLEX
Dim_size          = 26214
Dim_title         = 13C
Dim_units         = [ppm]
Dimensions        = X
Site              = ECS 400
Spectrometer      = JNM-ECS400

Field_strength    = 9.389766[T] (400[MHz]
X_acq_duration    = 1.04333312[s]
X_domain          = 13C
X_freq            = 100.52530333[MHz]
X_offset          = 100[ppm]
X_points          = 32768
X_prescans        = 4
X_resolution      = 0.95846665[Hz]
X_sweep           = 31.40703518[kHz]
Irr_domain        = 1H
Irr_freq          = 399.78219838[MHz]
Irr_offset        = 5[ppm]
Clipped           = TRUE
Mod_return        = 1
Scans             = 1024
Total_scans       = 1024

X_90_width        = 9.82623[us]
X_acq_time        = 1.04333312[s]
X_angle           = 30[deg]
X_atn             = 5.8[dB]
X_pulse           = 3.27541[us]
Irr_atn_dec       = 21[dB]
Irr_atn_noe       = 21[dB]
Irr_noise         = WALTZ
Decoupling        = TRUE
Initial_wait      = 1[s]
Noe               = TRUE
Noe_time          = 2[s]
Recvr_gain        = 60
Relaxation_delay  = 2[s]
Repetition_time   = 3.04333312[s]
Temp_get          = 25.4[dC]
```

Figure S66: 1H NMR Spectrum of Compound 35.

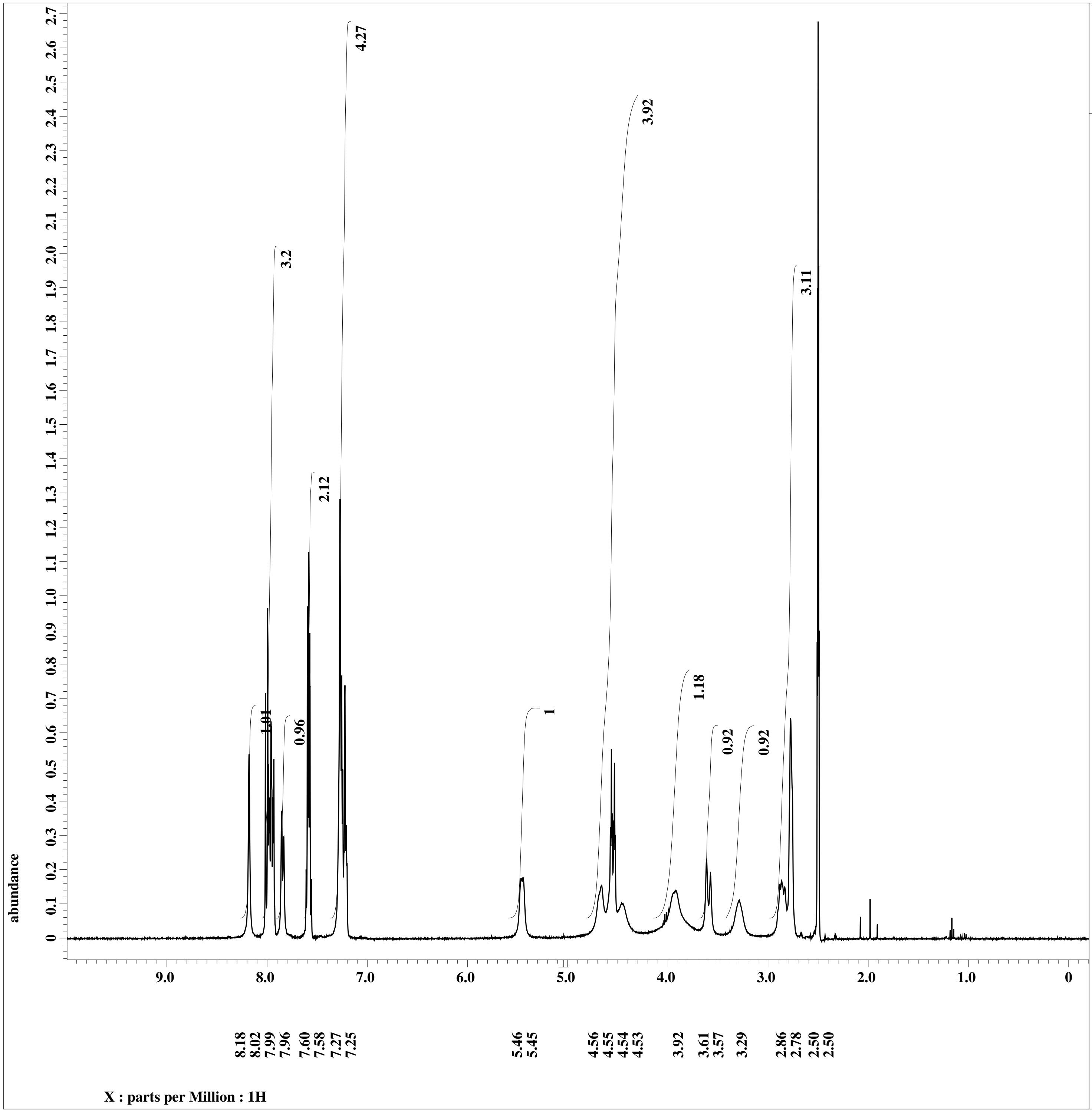

Filename	=	LSC-1-130 DMSO 1H NMR
Author	=	chemlabs
Experiment	=	single_pulse.ex2
Sample_id	=	001
Solvent	=	DMSO-D6
Creation_time	=	25-JUL-2017 08:43:04
Revision_time	=	21-JUL-2021 15:05:45
Current_time	=	21-JUL-2021 15:07:54
Comment	=	single_pulse
Data_format	=	1D COMPLEX
Dim_size	=	13107
Dim_title	=	1H
Dim_units	=	[ppm]
Dimensions	=	X
Site	=	ECS 400
Spectrometer	=	JNM-ECS400
Field_strength	=	9.389766[T] (400[MHz]
X_acq_duration	=	2.18365952[s]
X_domain	=	1H
X_freq	=	399.78219838[MHz]
X_offset	=	5[ppm]
X_points	=	16384
X_prescans	=	1
X_resolution	=	0.45794685[Hz]
X_sweep	=	7.5030012[kHz]
Irr_domain	=	1H
Irr_freq	=	399.78219838[MHz]
Irr_offset	=	5[ppm]
Tri_domain	=	1H
Tri_freq	=	399.78219838[MHz]
Tri_offset	=	5[ppm]
Clipped	=	FALSE
Mod_return	=	1
Scans	=	8
Total_scans	=	8
X_90_width	=	14.3[us]
X_acq_time	=	2.18365952[s]
X_angle	=	45[deg]
X_atn	=	3[dB]
X_pulse	=	7.15[us]
Irr_mode	=	Off
Tri_mode	=	Off
Dante_presat	=	FALSE
Initial_wait	=	1[s]
Recvr_gain	=	40
Relaxation_delay	=	5[s]
Repetition_time	=	7.18365952[s]
Temp_get	=	25.2[dC]

Figure S67: 13C NMR Spectrum of Compound 35.

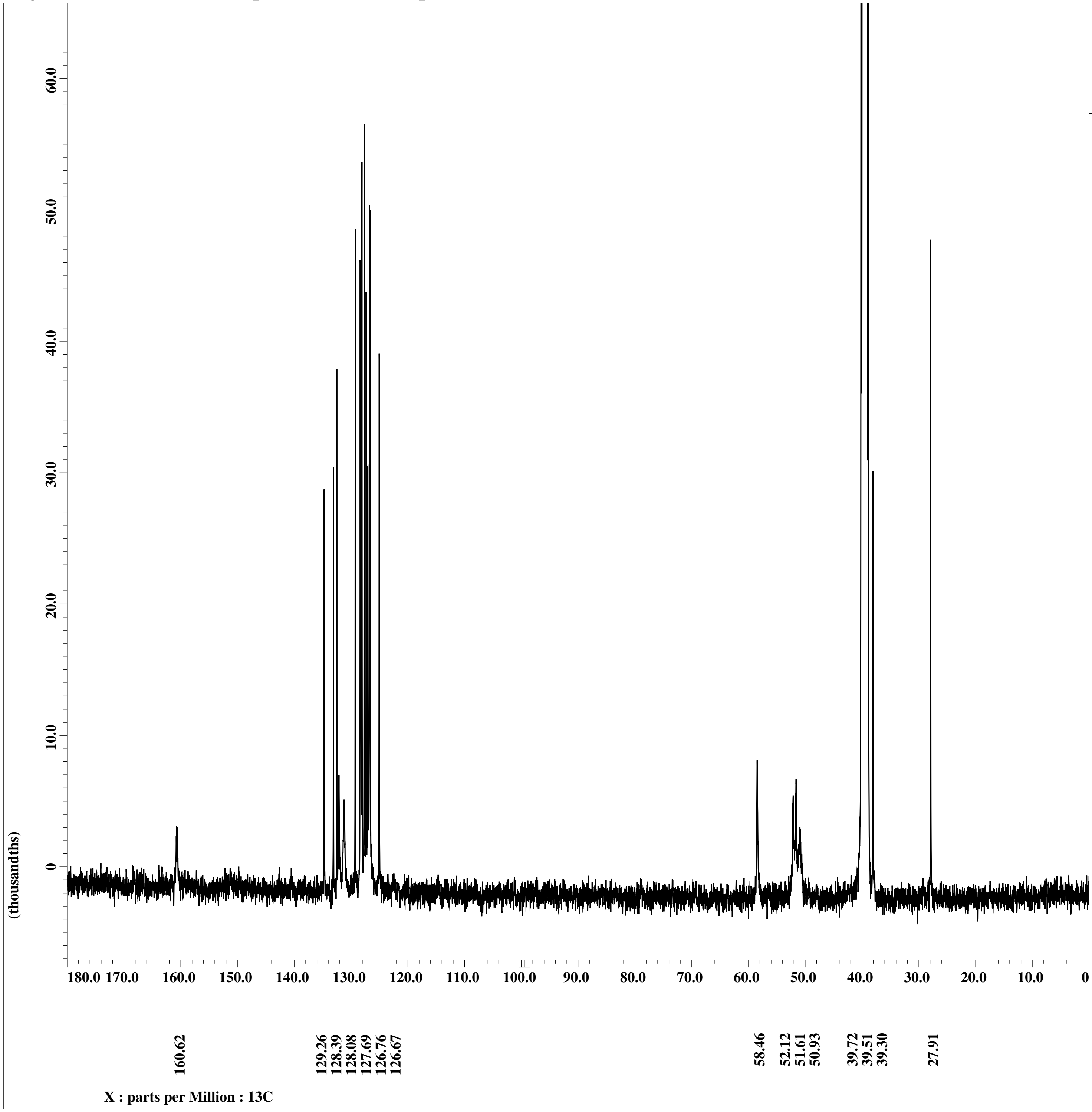


```
Filename           = LSC-1-130 DMSO 13C NM
Author             = chemlabs
Experiment         = single_pulse_dec
Sample_id          = 001
Solvent            = DMSO-D6
Creation_time      = 26-JUL-2017 04:26:03
Revision_time      = 21-JUL-2021 15:14:38
Current_time       = 21-JUL-2021 15:14:55

Comment            = single pulse decouple
Data_format        = 1D COMPLEX
Dim_size           = 26214
Dim_title          = 13C
Dim_units          = [ppm]
Dimensions         = X
Site               = ECS 400
Spectrometer       = JNM-ECS400

Field_strength     = 9.389766[T] (400[MHz]
X_acq_duration     = 1.04333312[s]
X_domain           = 13C
X_freq             = 100.52530333[MHz]
X_offset           = 100[ppm]
X_points           = 32768
X_prescans         = 4
X_resolution       = 0.95846665[Hz]
X_sweep            = 31.40703518[kHz]
Irr_domain         = 1H
Irr_freq           = 399.78219838[MHz]
Irr_offset         = 5[ppm]
Clipped            = FALSE
Mod_return         = 1
Scans              = 16384
Total_scans        = 16384

X_90_width         = 9.82623[us]
X_acq_time         = 1.04333312[s]
X_angle            = 30[deg]
X_atn              = 5.8[dB]
X_pulse            = 3.27541[us]
Irr_atn_dec        = 21[dB]
Irr_atn_noe        = 21[dB]
Irr_noise          = WALTZ
Decoupling         = TRUE
Initial_wait       = 1[s]
Noe                = TRUE
Noe_time           = 2[s]
Recvr_gain         = 60
Relaxation_delay   = 2[s]
Repetition_time    = 3.04333312[s]
Temp_get           = 24.7[dC]
```

Figure S68: 1H NMR Spectrum of Compound 36.

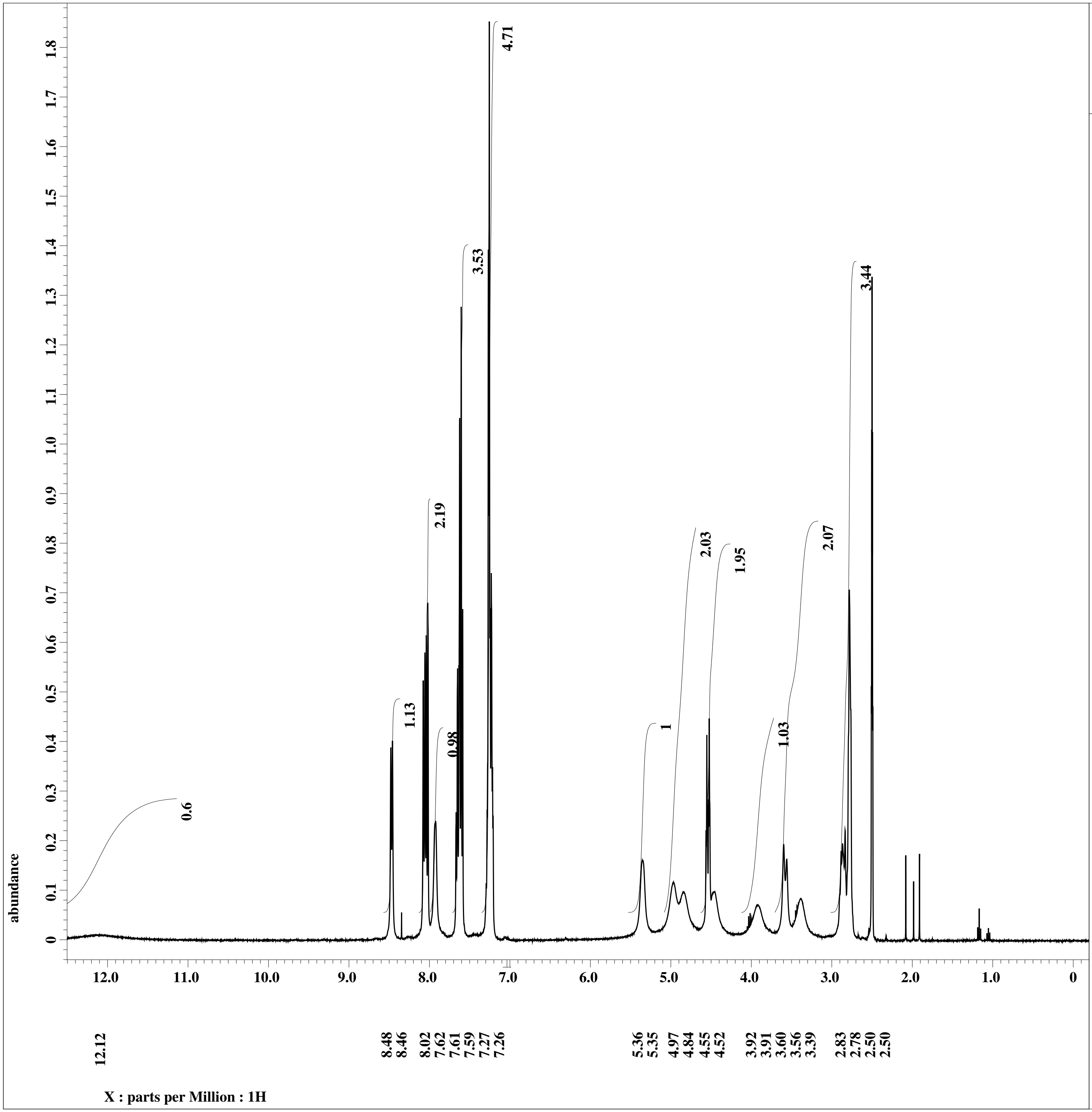

Filename = LSC-I-152_slot5_1H_NM
Author = russell research stud
Experiment = single_pulse.ex2
Sample_id = S#620429
Solvent = DMSO-D6
Creation_time = 23-JUL-2021 17:18:12
Revision_time = 26-JUL-2021 10:02:40
Current_time = 26-JUL-2021 10:03:04

Data_format = 1D COMPLEX
Dim_size = 13107
Dim_title = 1H
Dim_units = [ppm]
Dimensions = X
Site = ECS 400
Spectrometer = JNM-ECS400

Field_strength = 9.389766[T] (400[MHz]
X_acq_duration = 2.04734464[s]
X_domain = 1H
X_freq = 399.78219838[MHz]
X_offset = 7[ppm]
X_points = 16384
X_prescans = 1
X_resolution = 0.48843755[Hz]
X_sweep = 8.00256082[kHz]
Irr_domain = 1H
Irr_freq = 399.78219838[MHz]
Irr_offset = 5[ppm]
Tri_domain = 1H
Tri_freq = 399.78219838[MHz]
Tri_offset = 5[ppm]
Clipped = FALSE
Mod_return = 1
Scans = 8
Total_scans = 8

X_90_width = 14.3[us]
X_acq_time = 2.04734464[s]
X_angle = 45[deg]
X_atn = 3[dB]
X_pulse = 7.15[us]
Irr_mode = Off
Tri_mode = Off
Dante_presat = FALSE
Initial_wait = 1[s]
Recvr_gain = 36
Relaxation_delay = 5[s]
Repetition_time = 7.04734464[s]
Temp_get = 26.9[dC]

Figure S69: 13C NMR Spectrum of Compound 36.

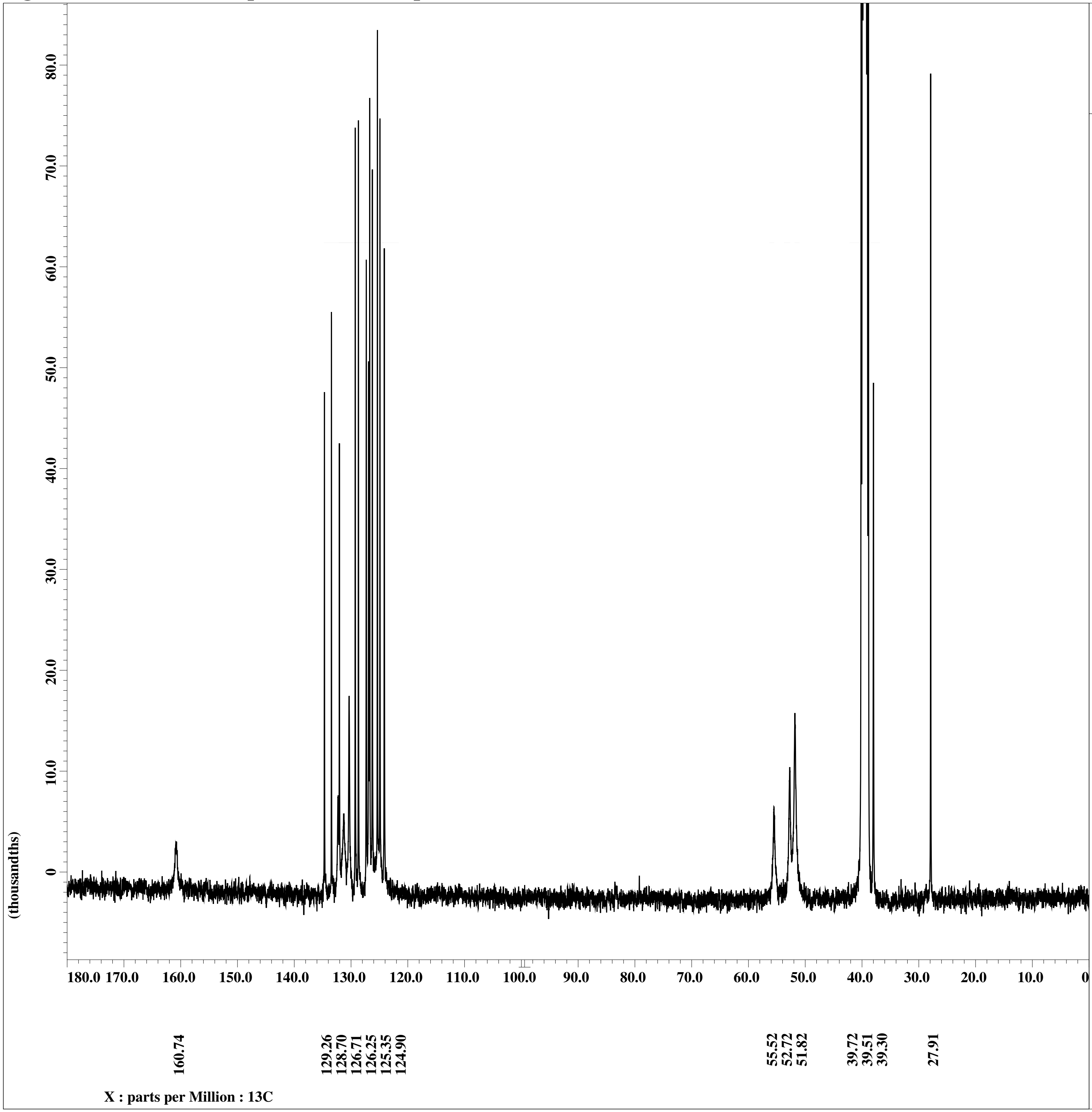

JEOL

```
Filename           = LSC-I-152_slot5_13C_N
Author             = russell research stud
Experiment         = single_pulse_dec
Sample_id          = S#621634
Solvent            = DMSO-D6
Creation_time      = 24-JUL-2021 07:09:49
Revision_time      = 26-JUL-2021 10:09:32
Current_time       = 26-JUL-2021 10:09:38

Data_format        = 1D COMPLEX
Dim_size           = 26214
Dim_title          = 13C
Dim_units          = [ppm]
Dimensions         = X
Site               = ECS 400
Spectrometer       = JNM-ECS400

Field_strength     = 9.389766[T] (400[MHz]
X_acq_duration     = 1.04333312[s]
X_domain           = 13C
X_freq             = 100.52530333[MHz]
X_offset           = 100[ppm]
X_points           = 32768
X_prescans         = 4
X_resolution       = 0.95846665[Hz]
X_sweep            = 31.40703518[kHz]
Irr_domain         = 1H
Irr_freq           = 399.78219838[MHz]
Irr_offset         = 5[ppm]
Clipped            = FALSE
Mod_return         = 1
Scans              = 16384
Total_scans        = 16384

X_90_width         = 9.82623[us]
X_acq_time         = 1.04333312[s]
X_angle            = 30[deg]
X_atn              = 5.8[dB]
X_pulse            = 3.27541[us]
Irr_atn_dec        = 21[dB]
Irr_atn_noe        = 21[dB]
Irr_noise          = WALTZ
Decoupling         = TRUE
Initial_wait       = 1[s]
Noe                = TRUE
Noe_time           = 2[s]
Recvr_gain         = 60
Relaxation_delay   = 2[s]
Repetition_time    = 3.04333312[s]
Temp_get           = 25.9[dC]
```

Figure S70: 1H NMR Spectrum of Compound 37.

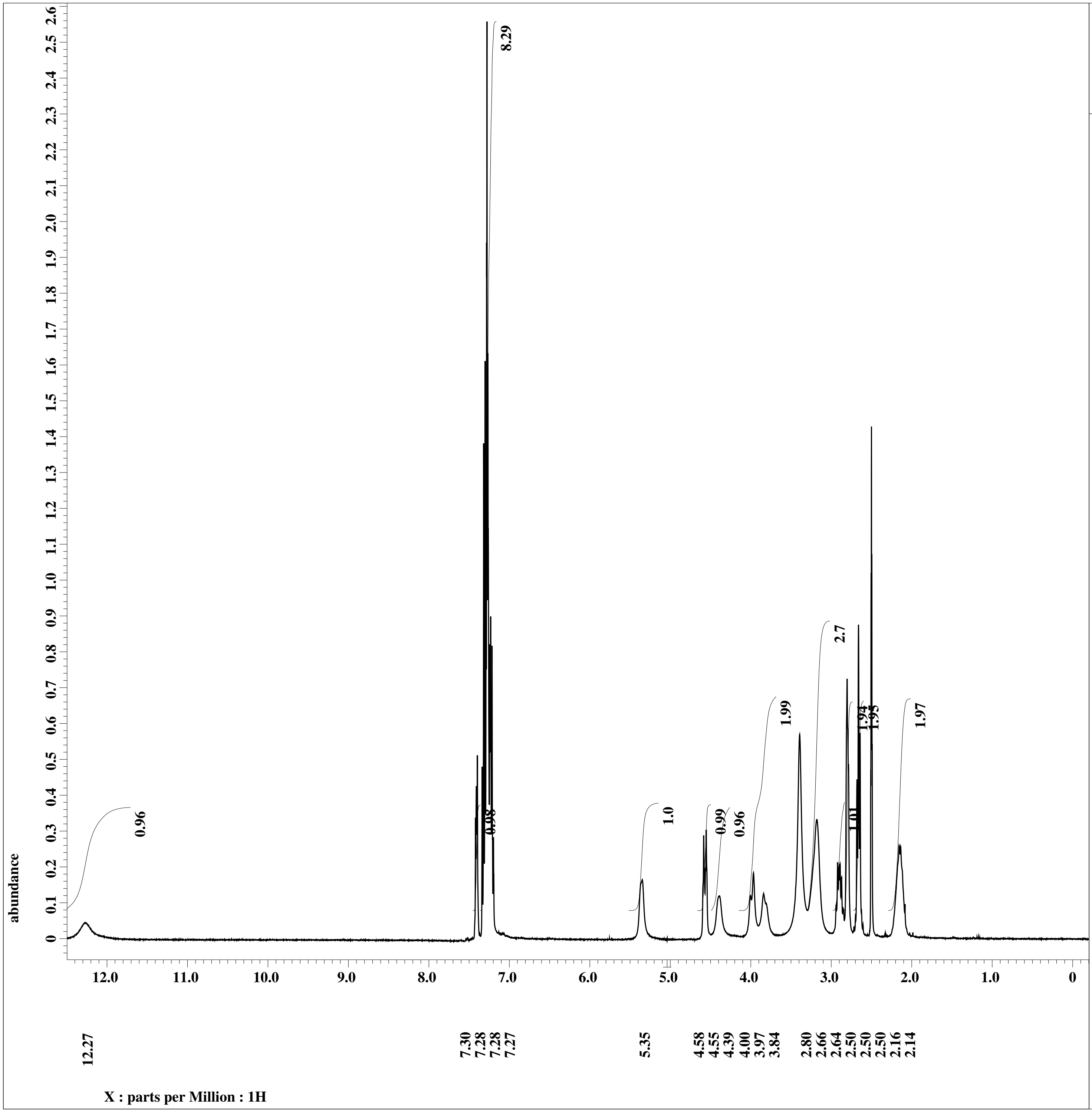


```
Filename          = LSC-1-092 DMSO 1H-4.j
Author            = chemlabs
Experiment        = single_pulse.ex2
Sample_id         = 001
Solvent           = DMSO-D6
Creation_time     = 27-JUL-2017 11:21:26
Revision_time     = 22-JUL-2021 08:32:59
Current_time      = 22-JUL-2021 08:39:26

Comment           = single_pulse
Data_format       = 1D COMPLEX
Dim_size          = 13107
Dim_title         = 1H
Dim_units         = [ppm]
Dimensions        = X
Site              = ECS 400
Spectrometer      = JNM-ECS400

Field_strength    = 9.389766[T] (400[MHz]
X_acq_duration    = 2.18365952[s]
X_domain          = 1H
X_freq            = 399.78219838[MHz]
X_offset          = 5[ppm]
X_points          = 16384
X_prescans        = 1
X_resolution      = 0.45794685[Hz]
X_sweep           = 7.5030012[kHz]
Irr_domain        = 1H
Irr_freq          = 399.78219838[MHz]
Irr_offset        = 5[ppm]
Tri_domain        = 1H
Tri_freq          = 399.78219838[MHz]
Tri_offset        = 5[ppm]
Clipped           = FALSE
Mod_return        = 1
Scans             = 8
Total_scans       = 8

X_90_width        = 14.3[us]
X_acq_time        = 2.18365952[s]
X_angle           = 45[deg]
X_atn             = 3[dB]
X_pulse           = 7.15[us]
Irr_mode          = Off
Tri_mode          = Off
Dante_presat      = FALSE
Initial_wait      = 1[s]
Recvr_gain        = 36
Relaxation_delay  = 5[s]
Repetition_time   = 7.18365952[s]
Temp_get          = 24.7[dC]
```

Figure S71: 13C NMR Spectrum of Compound 37.

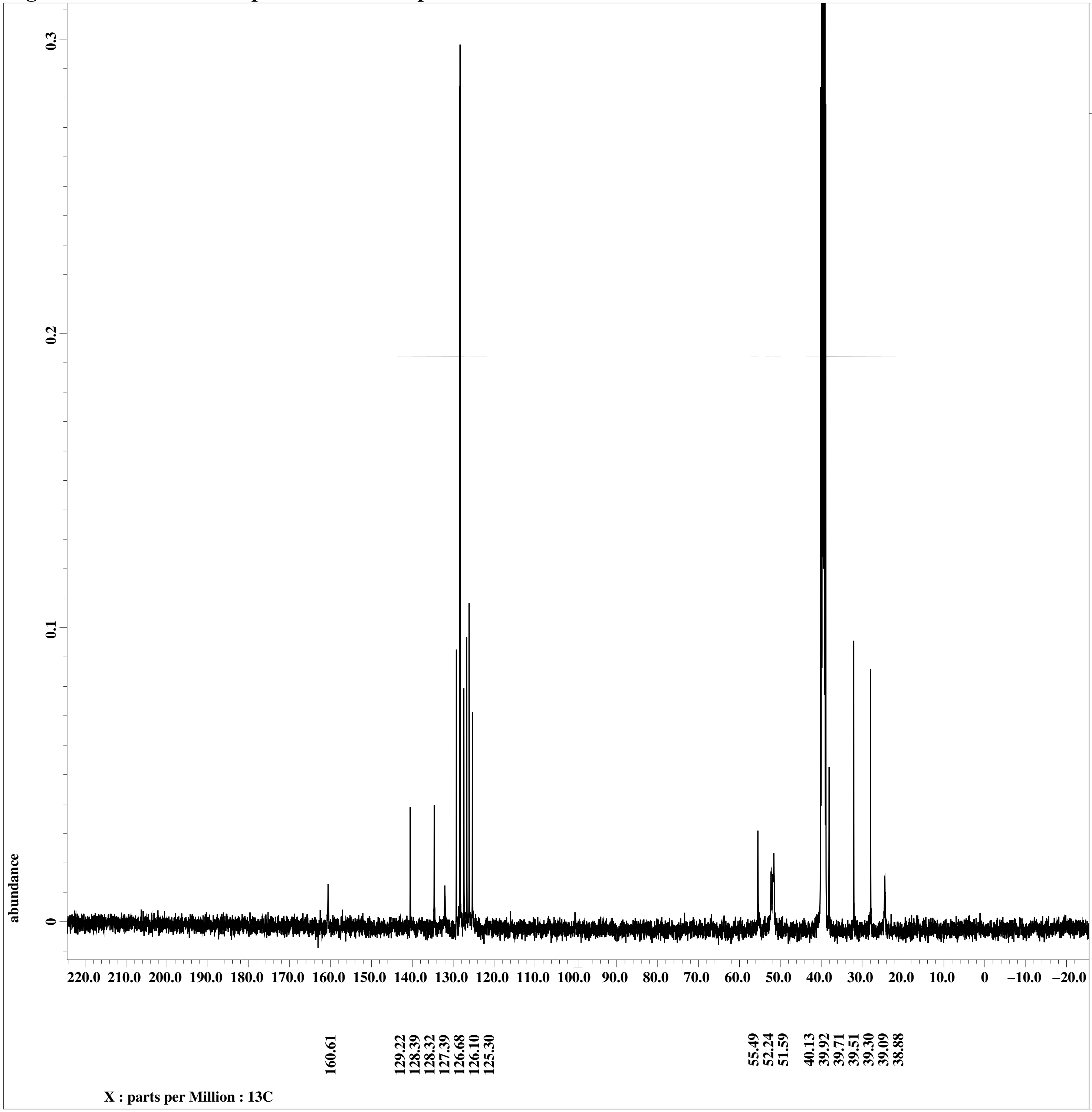

Filename	= LSC-1-092 DMSO 13C -3
Author	= chemlabs
Experiment	= single_pulse_dec
Sample_id	= 001
Solvent	= DMSO-D6
Creation_time	= 27-JUL-2017 12:52:21
Revision_time	= 22-JUL-2021 08:43:03
Current_time	= 22-JUL-2021 08:46:06
Comment	= single pulse decouple
Data_format	= 1D COMPLEX
Dim_size	= 26214
Dim_title	= 13C
Dim_units	= [ppm]
Dimensions	= X
Site	= ECS 400
Spectrometer	= JNM-ECS400
Field_strength	= 9.389766[T] (400[MHz]
X_acq_duration	= 1.04333312[s]
X_domain	= 13C
X_freq	= 100.52530333[MHz]
X_offset	= 100[ppm]
X_points	= 32768
X_prescans	= 4
X_resolution	= 0.95846665[Hz]
X_sweep	= 31.40703518[kHz]
Irr_domain	= 1H
Irr_freq	= 399.78219838[MHz]
Irr_offset	= 5[ppm]
Clipped	= FALSE
Mod_return	= 1
Scans	= 1709
Total_scans	= 1709
X_90_width	= 9.82623[us]
X_acq_time	= 1.04333312[s]
X_angle	= 30[deg]
X_atn	= 5.8[dB]
X_pulse	= 3.27541[us]
Irr_atn_dec	= 21[dB]
Irr_atn_noe	= 21[dB]
Irr_noise	= WALTZ
Decoupling	= TRUE
Initial_wait	= 1[s]
Noe	= TRUE
Noe_time	= 2[s]
Recvr_gain	= 60
Relaxation_delay	= 2[s]
Repetition_time	= 3.04333312[s]
Temp_get	= 25.2[dC]

Figure S72: 1H NMR Spectrum of Compound 38.

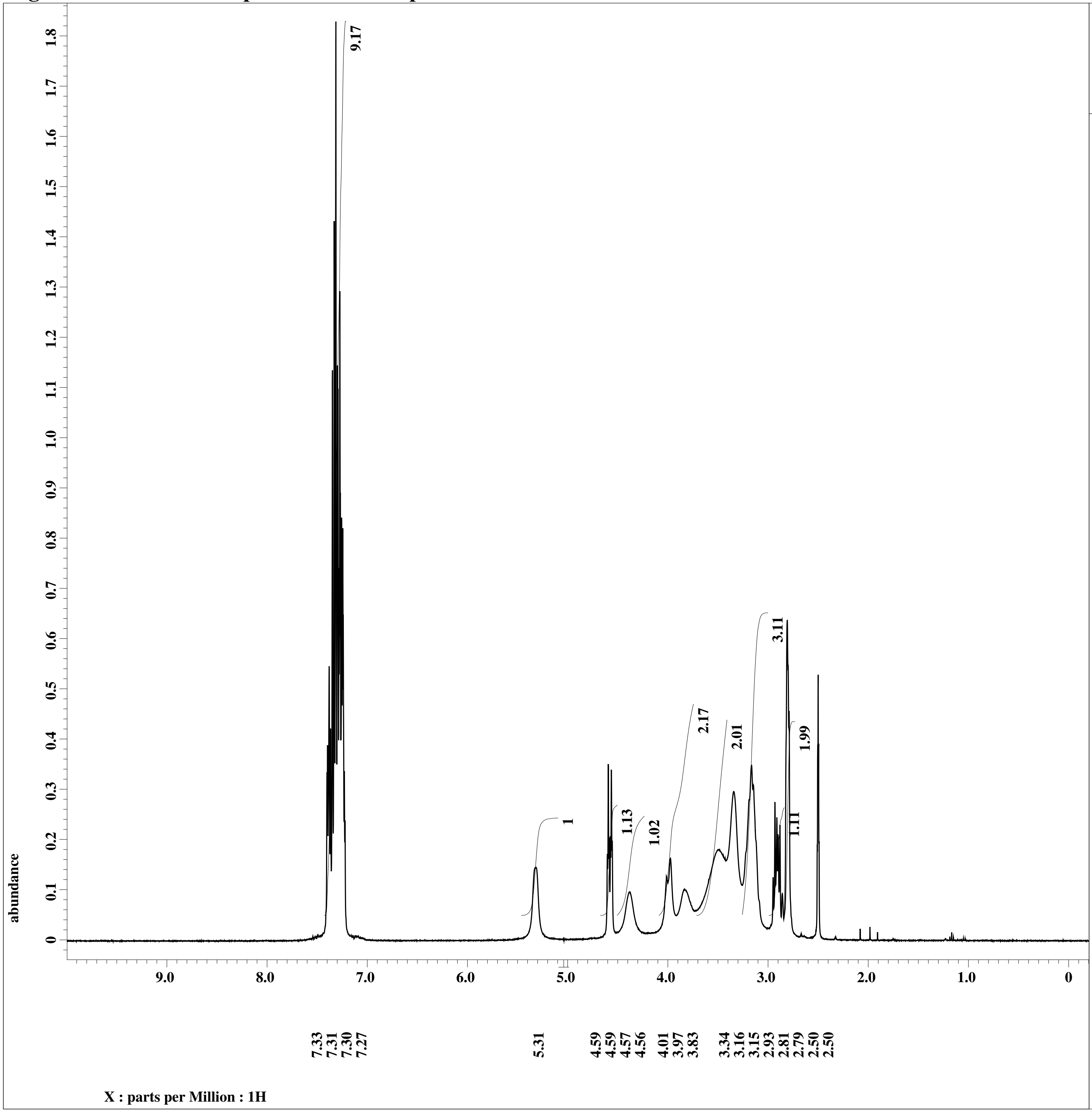


```
Filename          = LSC-1-100 DMSO 1H-9.j
Author            = chemlabs
Experiment        = single_pulse.ex2
Sample_id         = 001
Solvent           = DMSO-D6
Creation_time     = 28-JUL-2017 09:57:06
Revision_time     = 22-JUL-2021 08:52:30
Current_time      = 22-JUL-2021 08:56:23

Comment           = single_pulse
Data_format       = 1D COMPLEX
Dim_size          = 13107
Dim_title         = 1H
Dim_units         = [ppm]
Dimensions        = X
Site              = ECS 400
Spectrometer      = JNM-ECS400

Field_strength    = 9.389766[T] (400[MHz]
X_acq_duration    = 2.18365952[s]
X_domain          = 1H
X_freq            = 399.78219838[MHz]
X_offset          = 5[ppm]
X_points          = 16384
X_prescans        = 1
X_resolution      = 0.45794685[Hz]
X_sweep           = 7.5030012[kHz]
Irr_domain        = 1H
Irr_freq          = 399.78219838[MHz]
Irr_offset        = 5[ppm]
Tri_domain        = 1H
Tri_freq          = 399.78219838[MHz]
Tri_offset        = 5[ppm]
Clipped           = FALSE
Mod_return        = 1
Scans             = 8
Total_scans       = 8

X_90_width        = 14.3[us]
X_acq_time        = 2.18365952[s]
X_angle           = 45[deg]
X_atn             = 3[dB]
X_pulse           = 7.15[us]
Irr_mode          = Off
Tri_mode          = Off
Dante_presat      = FALSE
Initial_wait      = 1[s]
Recvr_gain        = 34
Relaxation_delay  = 5[s]
Repetition_time   = 7.18365952[s]
Temp_get          = 24[dC]
```

Figure S73: 13C NMR Spectrum of Compound 38.

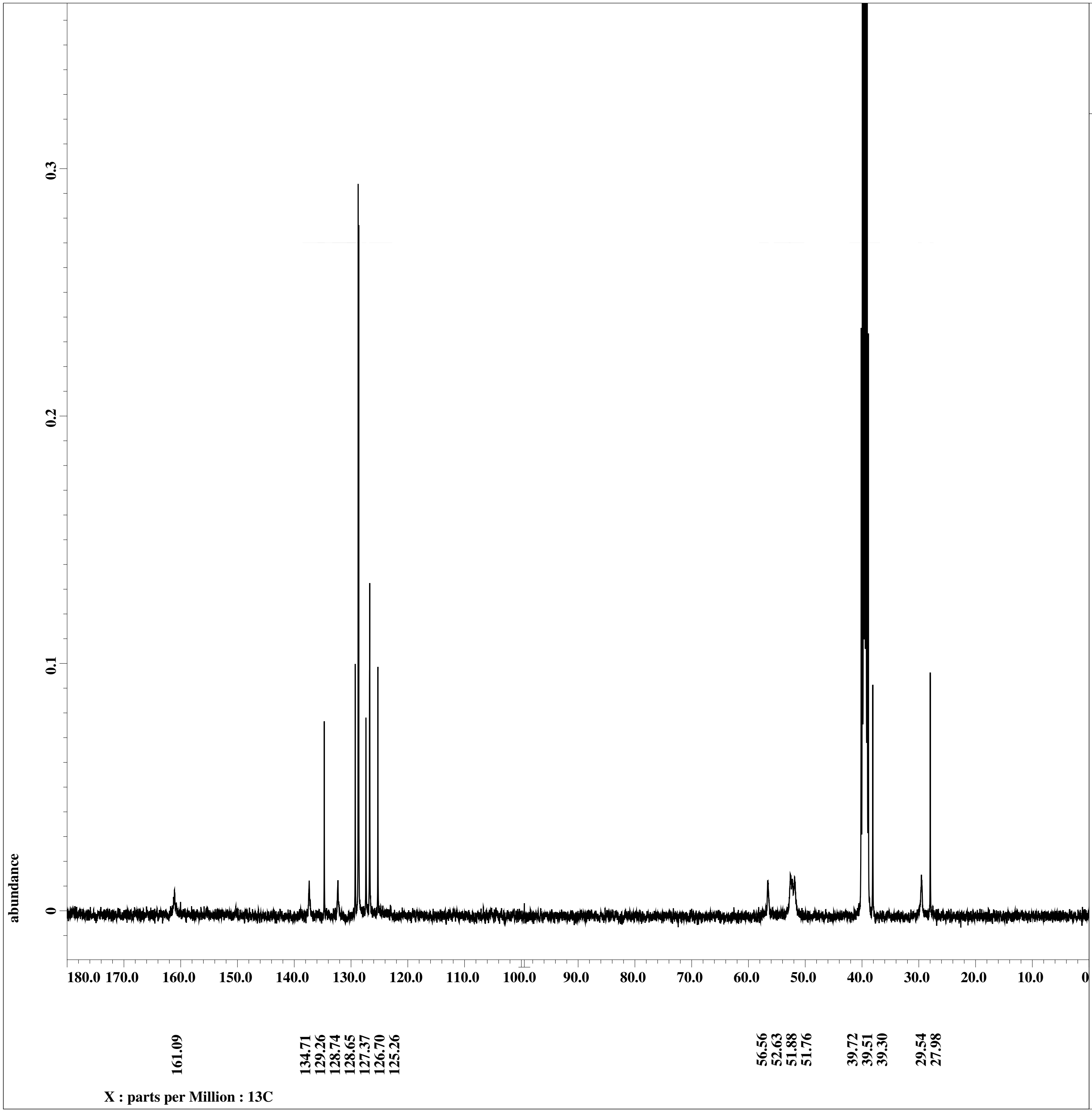

Filename	=	LSC-1-100 DMSO 13C-3.
Author	=	chemlabs
Experiment	=	single_pulse_dec
Sample_id	=	001
Solvent	=	DMSO-D6
Creation_time	=	28-JUL-2017 12:40:23
Revision_time	=	22-JUL-2021 08:58:19
Current_time	=	22-JUL-2021 09:01:02
Comment	=	single pulse decouple
Data_format	=	1D COMPLEX
Dim_size	=	26214
Dim_title	=	13C
Dim_units	=	[ppm]
Dimensions	=	X
Site	=	ECS 400
Spectrometer	=	JNM-ECS400
Field_strength	=	9.389766[T] (400[MHz]
X_acq_duration	=	1.04333312[s]
X_domain	=	13C
X_freq	=	100.52530333[MHz]
X_offset	=	100[ppm]
X_points	=	32768
X_prescans	=	4
X_resolution	=	0.95846665[Hz]
X_sweep	=	31.40703518[kHz]
Irr_domain	=	1H
Irr_freq	=	399.78219838[MHz]
Irr_offset	=	5[ppm]
Clipped	=	TRUE
Mod_return	=	1
Scans	=	3190
Total_scans	=	3190
X_90_width	=	9.82623[us]
X_acq_time	=	1.04333312[s]
X_angle	=	30[deg]
X_atn	=	5.8[dB]
X_pulse	=	3.27541[us]
Irr_atn_dec	=	21[dB]
Irr_atn_noe	=	21[dB]
Irr_noise	=	WALTZ
Decoupling	=	TRUE
Initial_wait	=	1[s]
Noe	=	TRUE
Noe_time	=	2[s]
Recvr_gain	=	60
Relaxation_delay	=	2[s]
Repetition_time	=	3.04333312[s]
Temp_get	=	25[dC]

Figure S74: 1H NMR Spectrum of Compound 39.

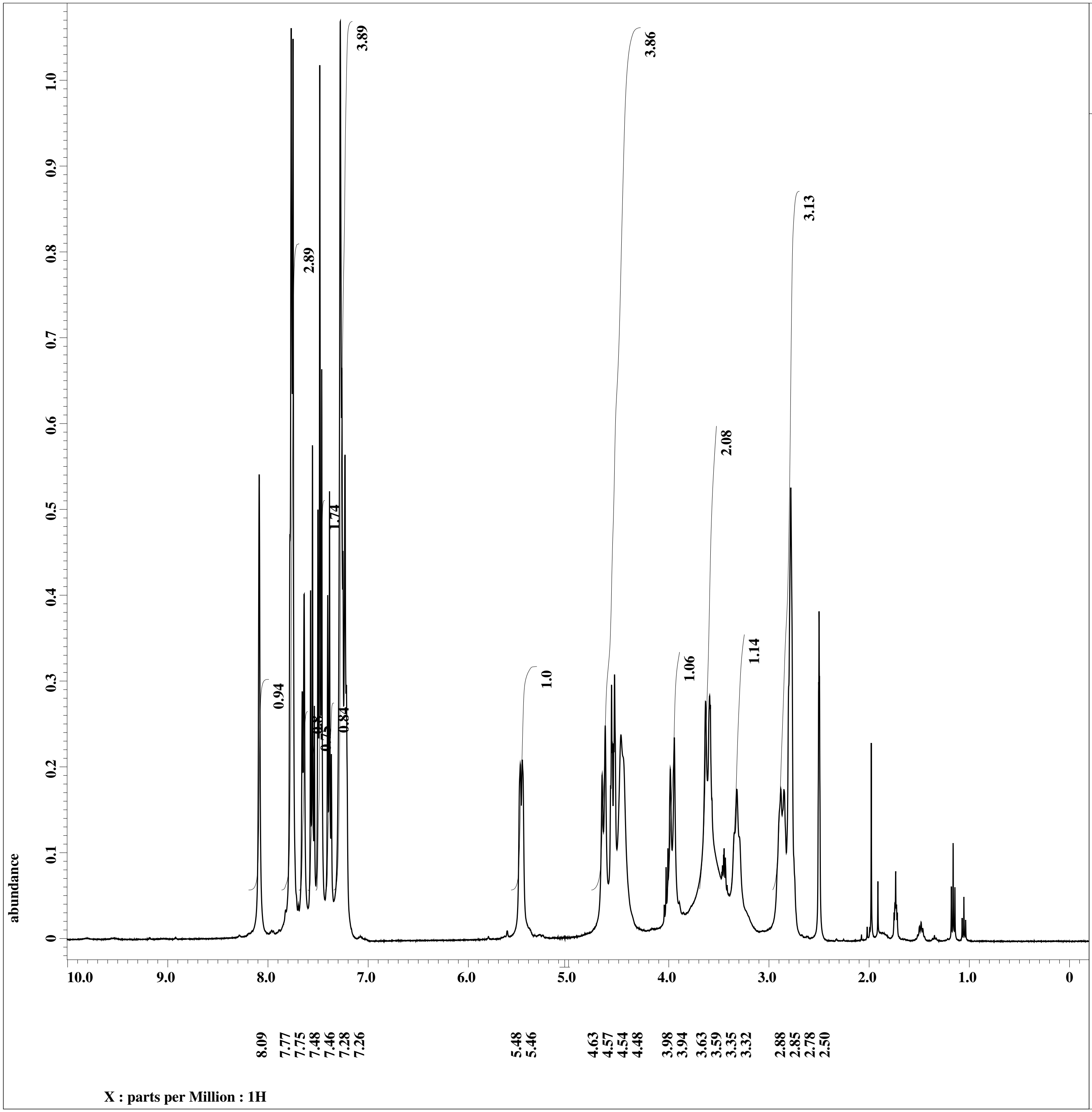

Filename	= LSC-1-098 DMSO 1H-4.j
Author	= chemlabs
Experiment	= single_pulse.ex2
Sample_id	= 001
Solvent	= DMSO-D6
Creation_time	= 27-JUL-2017 14:39:08
Revision_time	= 22-JUL-2021 09:09:10
Current_time	= 22-JUL-2021 09:12:00
Comment	= single_pulse
Data_format	= 1D COMPLEX
Dim_size	= 13107
Dim_title	= 1H
Dim_units	= [ppm]
Dimensions	= X
Site	= ECS 400
Spectrometer	= JNM-ECS400
Field_strength	= 9.389766[T] (400[MHz]
X_acq_duration	= 2.18365952[s]
X_domain	= 1H
X_freq	= 399.78219838[MHz]
X_offset	= 5[ppm]
X_points	= 16384
X_prescans	= 1
X_resolution	= 0.45794685[Hz]
X_sweep	= 7.5030012[kHz]
Irr_domain	= 1H
Irr_freq	= 399.78219838[MHz]
Irr_offset	= 5[ppm]
Tri_domain	= 1H
Tri_freq	= 399.78219838[MHz]
Tri_offset	= 5[ppm]
Clipped	= FALSE
Mod_return	= 1
Scans	= 8
Total_scans	= 8
X_90_width	= 14.3[us]
X_acq_time	= 2.18365952[s]
X_angle	= 45[deg]
X_atn	= 3[dB]
X_pulse	= 7.15[us]
Irr_mode	= Off
Tri_mode	= Off
Dante_presat	= FALSE
Initial_wait	= 1[s]
Recvr_gain	= 28
Relaxation_delay	= 5[s]
Repetition_time	= 7.18365952[s]
Temp_get	= 24.4[dC]

Figure S75: 13C NMR Spectrum of Compound 39.

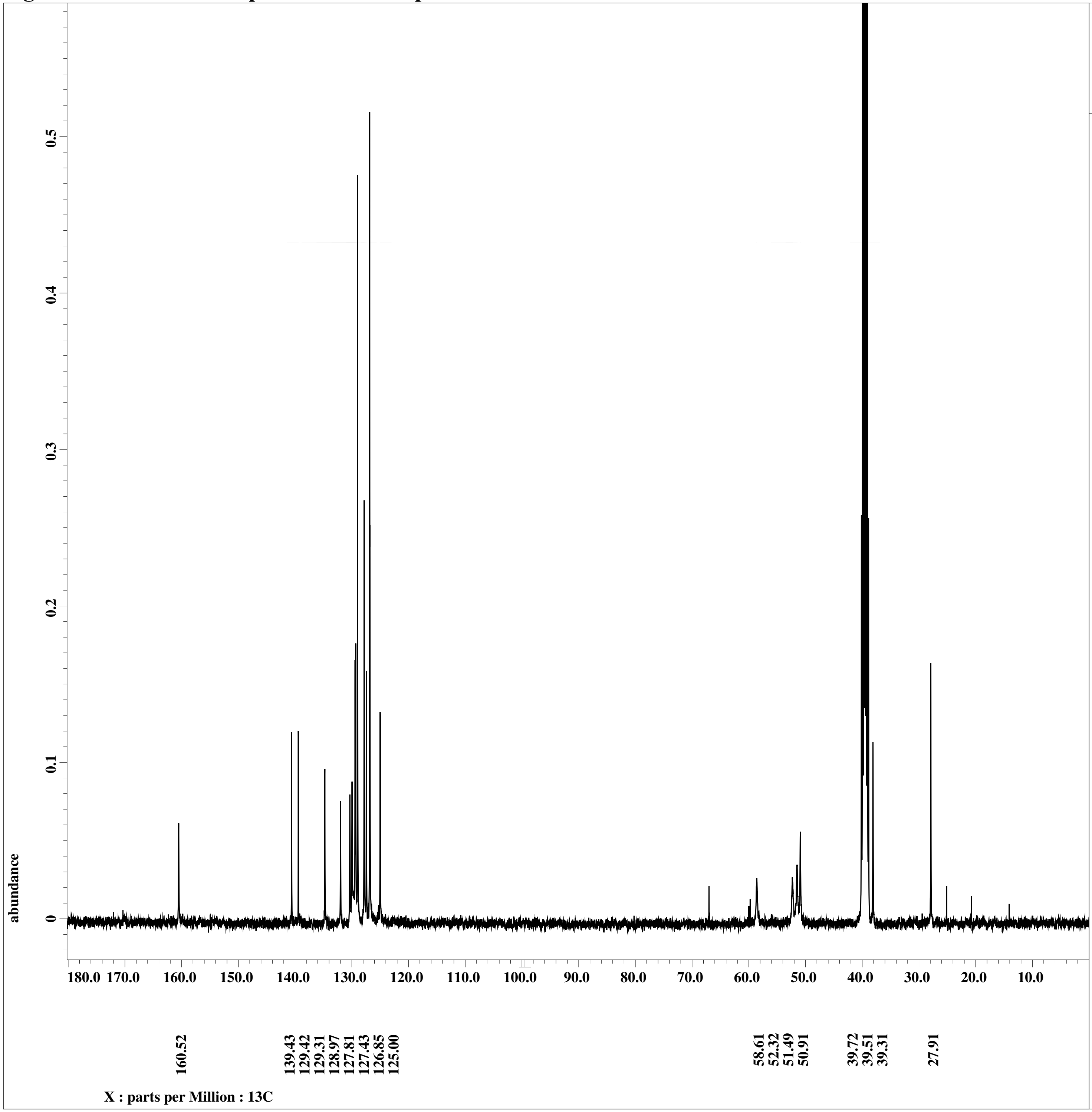


```
Filename          = LSC-1-098 DMSO 13C co
Author            = chemlabs
Experiment        = single_pulse_dec
Sample_id         = 001
Solvent           = DMSO-D6
Creation_time     = 27-JUL-2017 16:06:12
Revision_time     = 23-JUL-2020 16:27:23
Current_time      = 22-JUL-2021 09:17:01

Comment           = single pulse decouple
Data_format       = 1D COMPLEX
Dim_size          = 26214
Dim_title         = 13C
Dim_units         = [ppm]
Dimensions        = X
Site              = ECS 400
Spectrometer      = JNM-ECS400

Field_strength    = 9.389766[T] (400[MHz]
X_acq_duration    = 1.04333312[s]
X_domain          = 13C
X_freq            = 100.52530333[MHz]
X_offset          = 100[ppm]
X_points          = 32768
X_prescans        = 4
X_resolution      = 0.95846665[Hz]
X_sweep           = 31.40703518[kHz]
Irr_domain        = 1H
Irr_freq          = 399.78219838[MHz]
Irr_offset        = 5[ppm]
Clipped           = FALSE
Mod_return        = 1
Scans             = 1673
Total_scans       = 1673

X_90_width        = 9.82623[us]
X_acq_time        = 1.04333312[s]
X_angle           = 30[deg]
X_atn             = 5.8[dB]
X_pulse           = 3.27541[us]
Irr_atn_dec       = 21[dB]
Irr_atn_noe       = 21[dB]
Irr_noise         = WALTZ
Decoupling        = TRUE
Initial_wait      = 1[s]
Noe               = TRUE
Noe_time          = 2[s]
Recvr_gain        = 60
Relaxation_delay  = 2[s]
Repetition_time   = 3.04333312[s]
Temp_get          = 25.2[dC]
```

Figure S76: 1H NMR Spectrum of Compound 40.

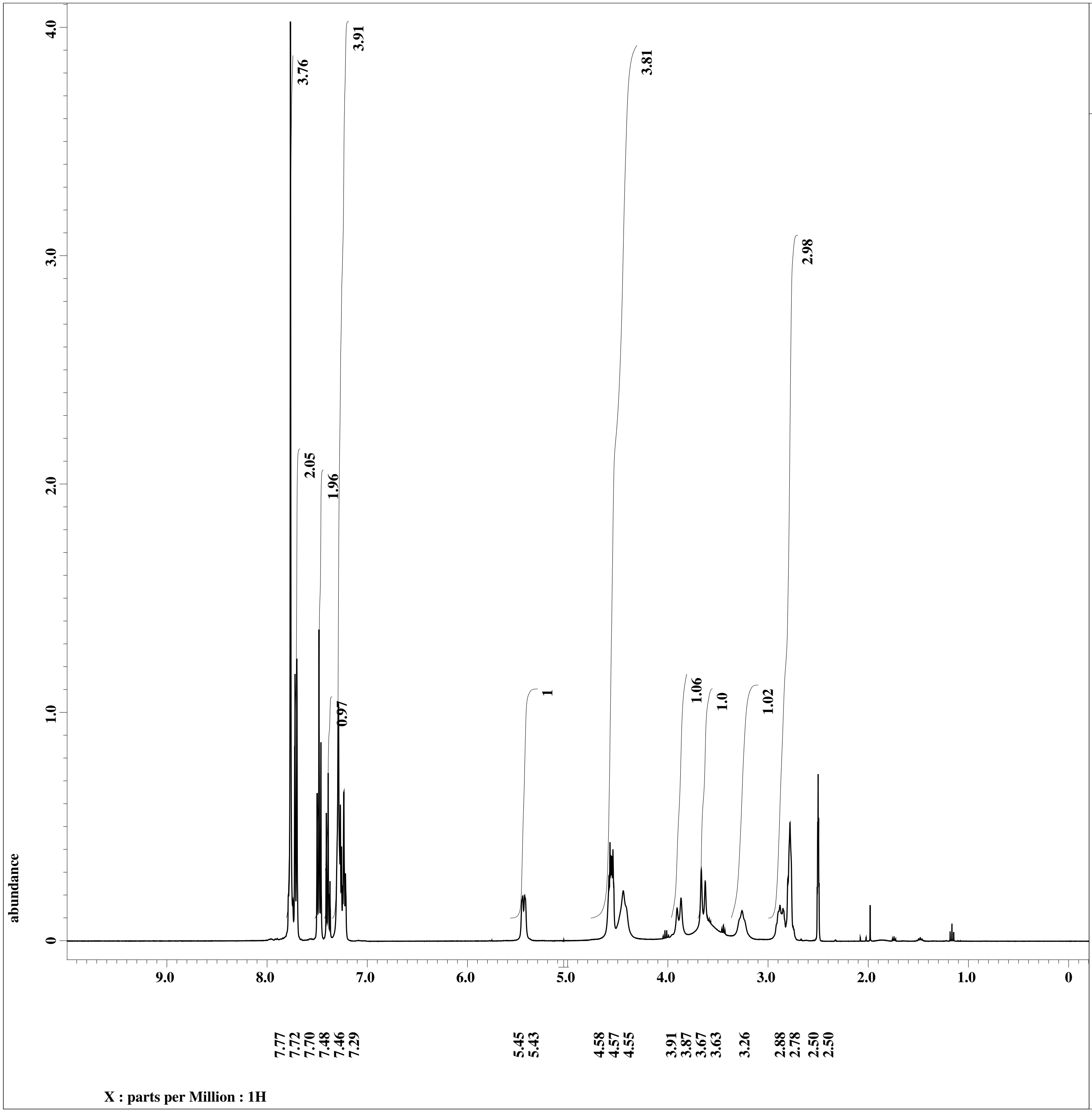


```
Filename          = LSC-1-096 DMSO 1H NMR
Author            = chemlabs
Experiment        = single_pulse.ex2
Sample_id         = 001
Solvent           = DMSO-D6
Creation_time     = 24-JUL-2017 10:01:20
Revision_time     = 22-JUL-2021 09:24:32
Current_time      = 22-JUL-2021 09:28:12

Comment           = single_pulse
Data_format       = 1D COMPLEX
Dim_size          = 13107
Dim_title         = 1H
Dim_units         = [ppm]
Dimensions        = X
Site              = ECS 400
Spectrometer      = JNM-ECS400

Field_strength    = 9.389766[T] (400[MHz]
X_acq_duration    = 2.18365952[s]
X_domain          = 1H
X_freq            = 399.78219838[MHz]
X_offset          = 5[ppm]
X_points          = 16384
X_prescans        = 1
X_resolution      = 0.45794685[Hz]
X_sweep           = 7.5030012[kHz]
Irr_domain        = 1H
Irr_freq          = 399.78219838[MHz]
Irr_offset        = 5[ppm]
Tri_domain        = 1H
Tri_freq          = 399.78219838[MHz]
Tri_offset        = 5[ppm]
Clipped           = FALSE
Mod_return        = 1
Scans             = 8
Total_scans       = 8

X_90_width        = 14.3[us]
X_acq_time        = 2.18365952[s]
X_angle           = 45[deg]
X_atn             = 3[dB]
X_pulse           = 7.15[us]
Irr_mode          = Off
Tri_mode          = Off
Dante_presat      = FALSE
Initial_wait      = 1[s]
Recvr_gain        = 30
Relaxation_delay  = 5[s]
Repetition_time   = 7.18365952[s]
Temp_get          = 23.9[dC]
```

Figure S77: 13C NMR Spectrum of Compound 40.

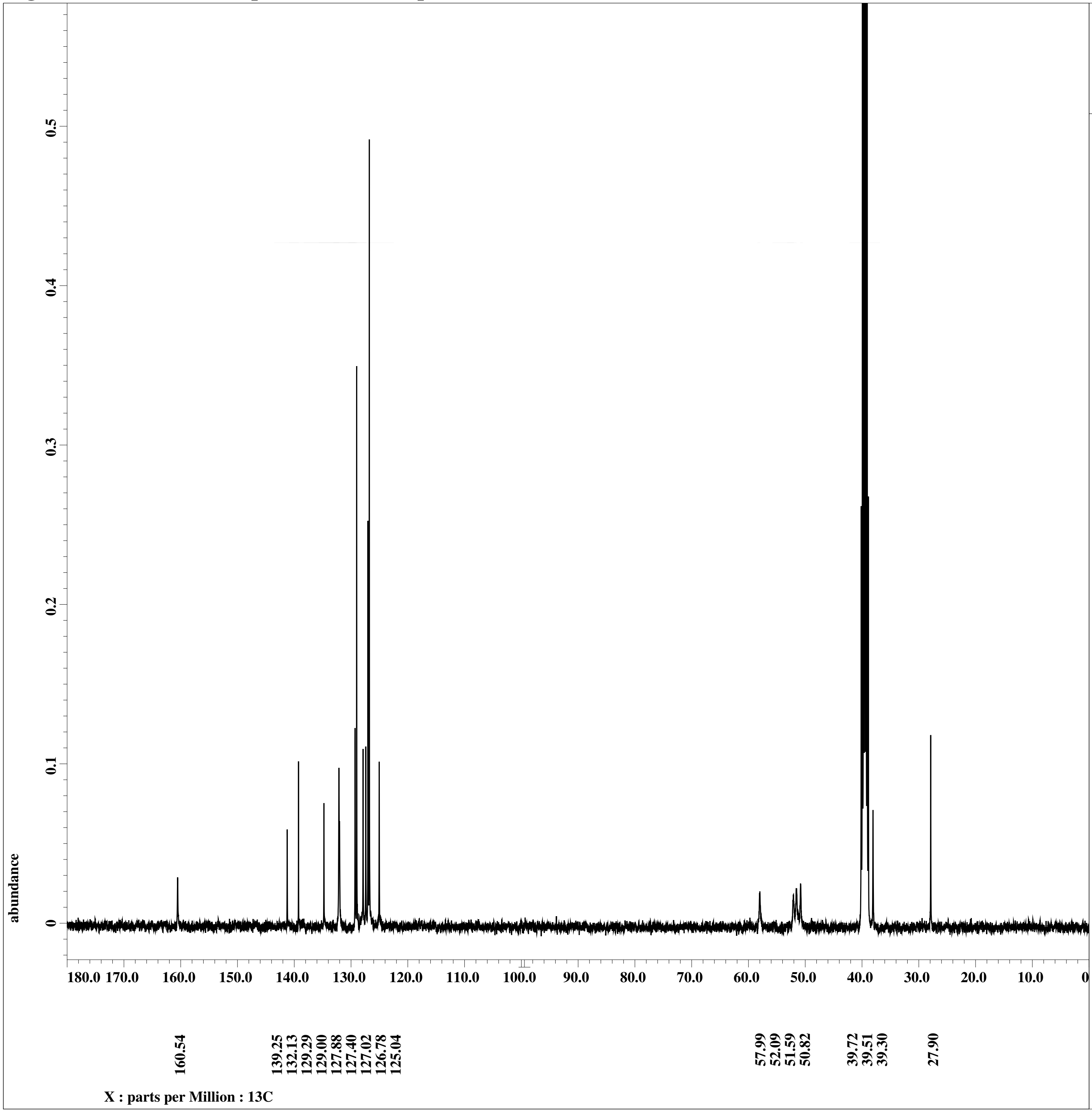

Filename	= LSC-1-096 DMSO 13C NM
Author	= chemlabs
Experiment	= single_pulse_dec
Sample_id	= 001
Solvent	= DMSO-D6
Creation_time	= 24-JUL-2017 11:19:15
Revision_time	= 22-JUL-2021 09:29:23
Current_time	= 22-JUL-2021 09:34:46
Comment	= single pulse decouple
Data_format	= 1D COMPLEX
Dim_size	= 26214
Dim_title	= 13C
Dim_units	= [ppm]
Dimensions	= X
Site	= ECS 400
Spectrometer	= JNM-ECS400
Field_strength	= 9.389766[T] (400[MHz]
X_acq_duration	= 1.04333312[s]
X_domain	= 13C
X_freq	= 100.52530333[MHz]
X_offset	= 100[ppm]
X_points	= 32768
X_prescans	= 4
X_resolution	= 0.95846665[Hz]
X_sweep	= 31.40703518[kHz]
Irr_domain	= 1H
Irr_freq	= 399.78219838[MHz]
Irr_offset	= 5[ppm]
Clipped	= FALSE
Mod_return	= 1
Scans	= 1519
Total_scans	= 1519
X_90_width	= 9.82623[us]
X_acq_time	= 1.04333312[s]
X_angle	= 30[deg]
X_atn	= 5.8[dB]
X_pulse	= 3.27541[us]
Irr_atn_dec	= 21[dB]
Irr_atn_noe	= 21[dB]
Irr_noise	= WALTZ
Decoupling	= TRUE
Initial_wait	= 1[s]
Noe	= TRUE
Noe_time	= 2[s]
Recvr_gain	= 60
Relaxation_delay	= 2[s]
Repetition_time	= 3.04333312[s]
Temp_get	= 24.3[dC]

Figure S78: 1H NMR Spectrum of Compound 41.

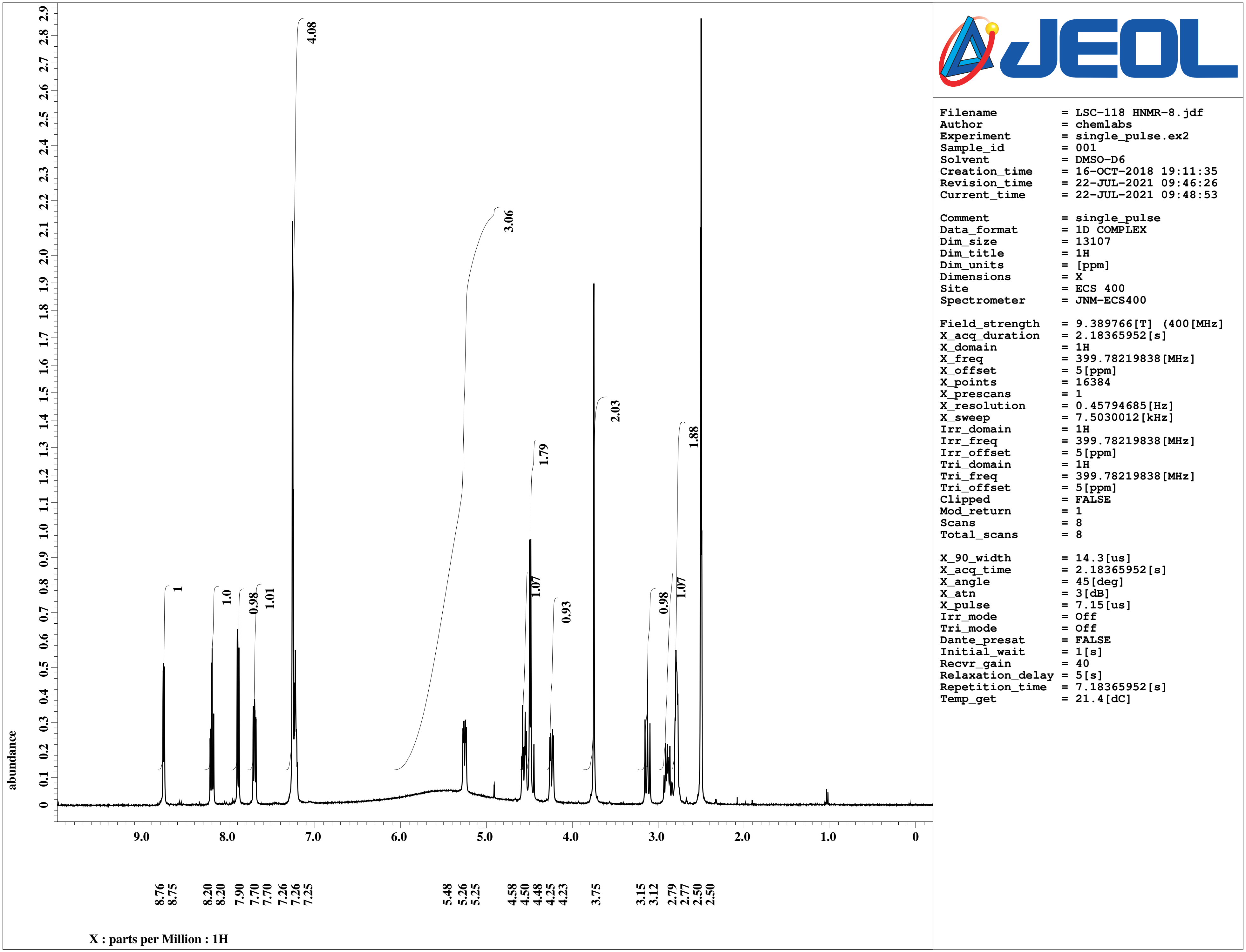

Figure S79: 13C NMR Spectrum of Compound 41.

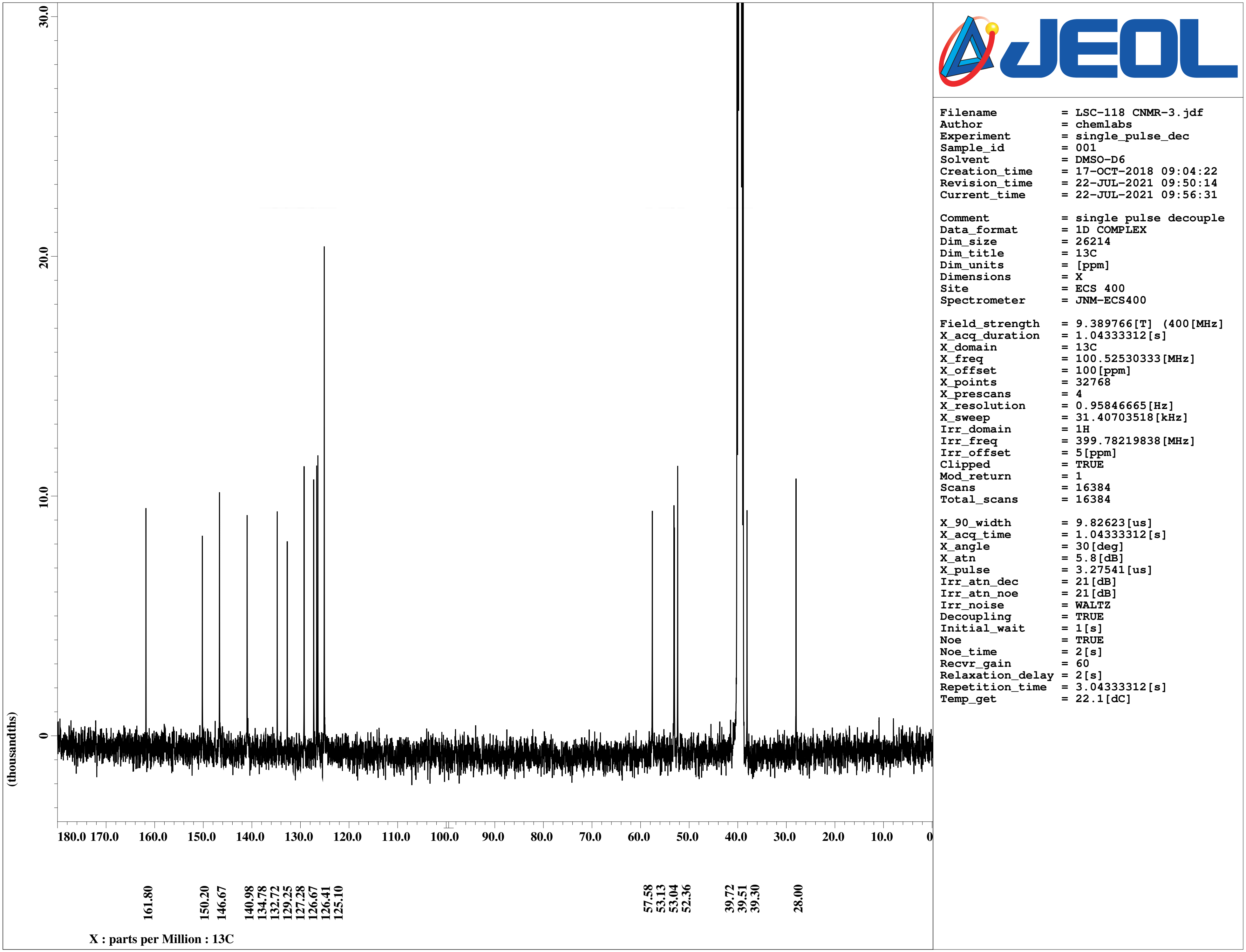

Figure S80: 1H NMR Spectrum of Compound 42.

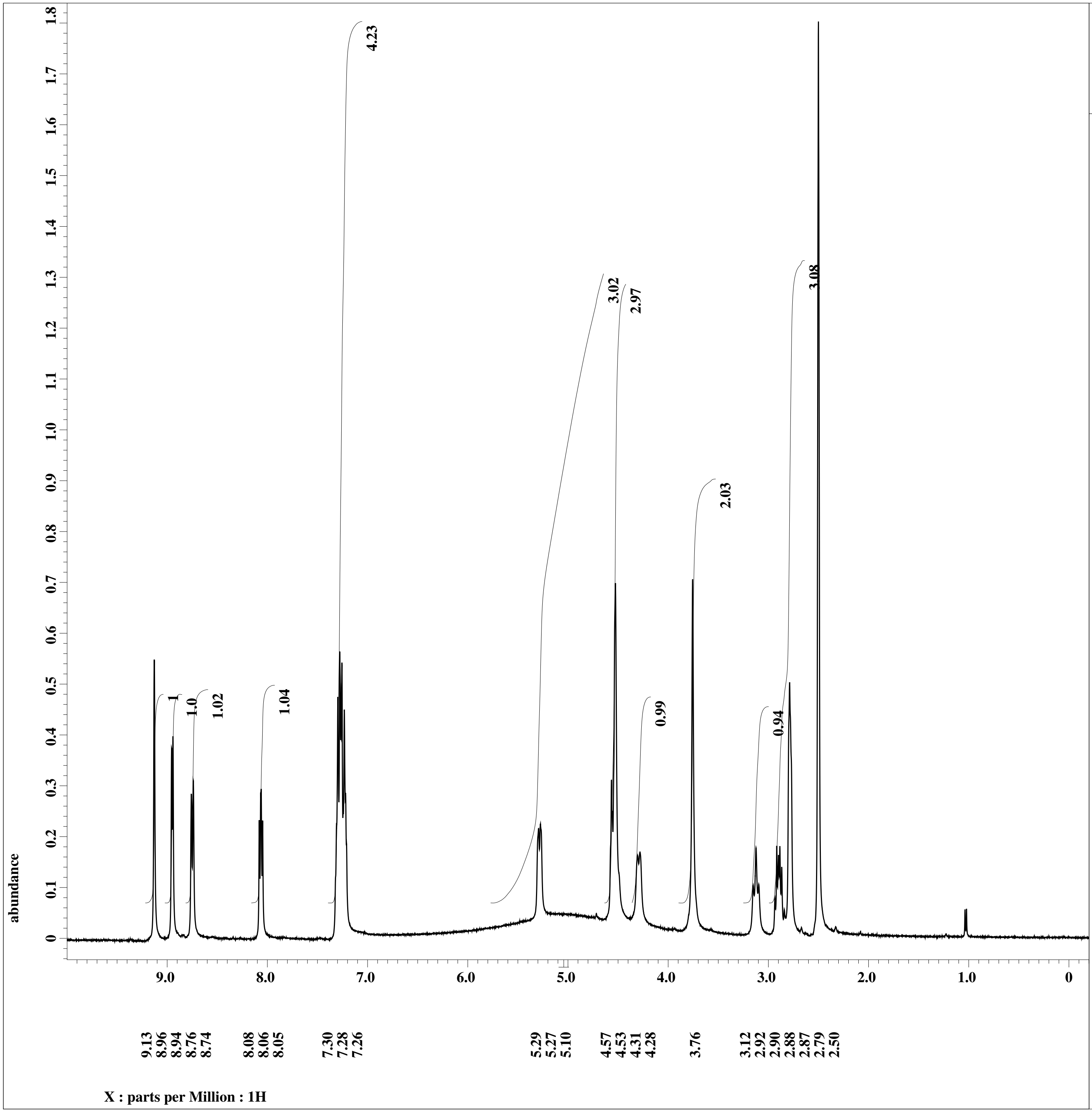


```
Filename          = LSC-120 HNMR-5.jdf
Author            = chemlabs
Experiment        = single_pulse.ex2
Sample_id         = 001
Solvent           = DMSO-D6
Creation_time     = 16-OCT-2018 08:27:28
Revision_time     = 22-JUL-2021 10:02:43
Current_time      = 22-JUL-2021 10:04:22

Comment           = single_pulse
Data_format       = 1D COMPLEX
Dim_size          = 13107
Dim_title         = 1H
Dim_units         = [ppm]
Dimensions        = X
Site              = ECS 400
Spectrometer      = JNM-ECS400

Field_strength    = 9.389766[T] (400[MHz]
X_acq_duration    = 2.18365952[s]
X_domain          = 1H
X_freq            = 399.78219838[MHz]
X_offset          = 5[ppm]
X_points          = 16384
X_prescans        = 1
X_resolution      = 0.45794685[Hz]
X_sweep           = 7.5030012[kHz]
Irr_domain        = 1H
Irr_freq          = 399.78219838[MHz]
Irr_offset        = 5[ppm]
Tri_domain        = 1H
Tri_freq          = 399.78219838[MHz]
Tri_offset        = 5[ppm]
Clipped           = FALSE
Mod_return        = 1
Scans             = 8
Total_scans       = 8

X_90_width        = 14.3[us]
X_acq_time        = 2.18365952[s]
X_angle           = 45[deg]
X_atn             = 3[dB]
X_pulse           = 7.15[us]
Irr_mode          = Off
Tri_mode          = Off
Dante_presat      = FALSE
Initial_wait      = 1[s]
Recvr_gain        = 38
Relaxation_delay  = 5[s]
Repetition_time   = 7.18365952[s]
Temp_get          = 21.7[dC]
```

Figure S81: 13C NMR Spectrum of Compound 42.

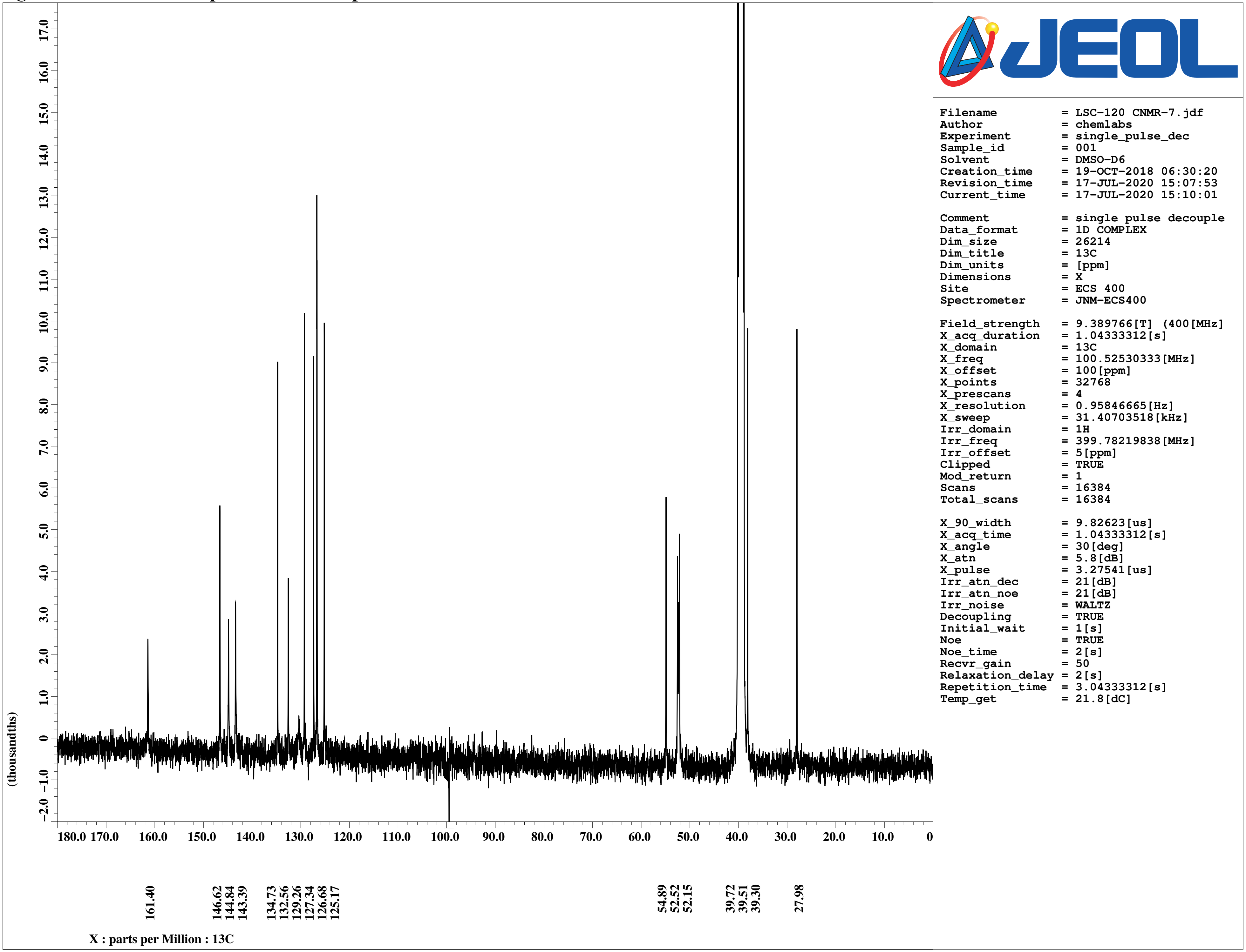

Figure S82: 1H NMR Spectrum of Compound 43.

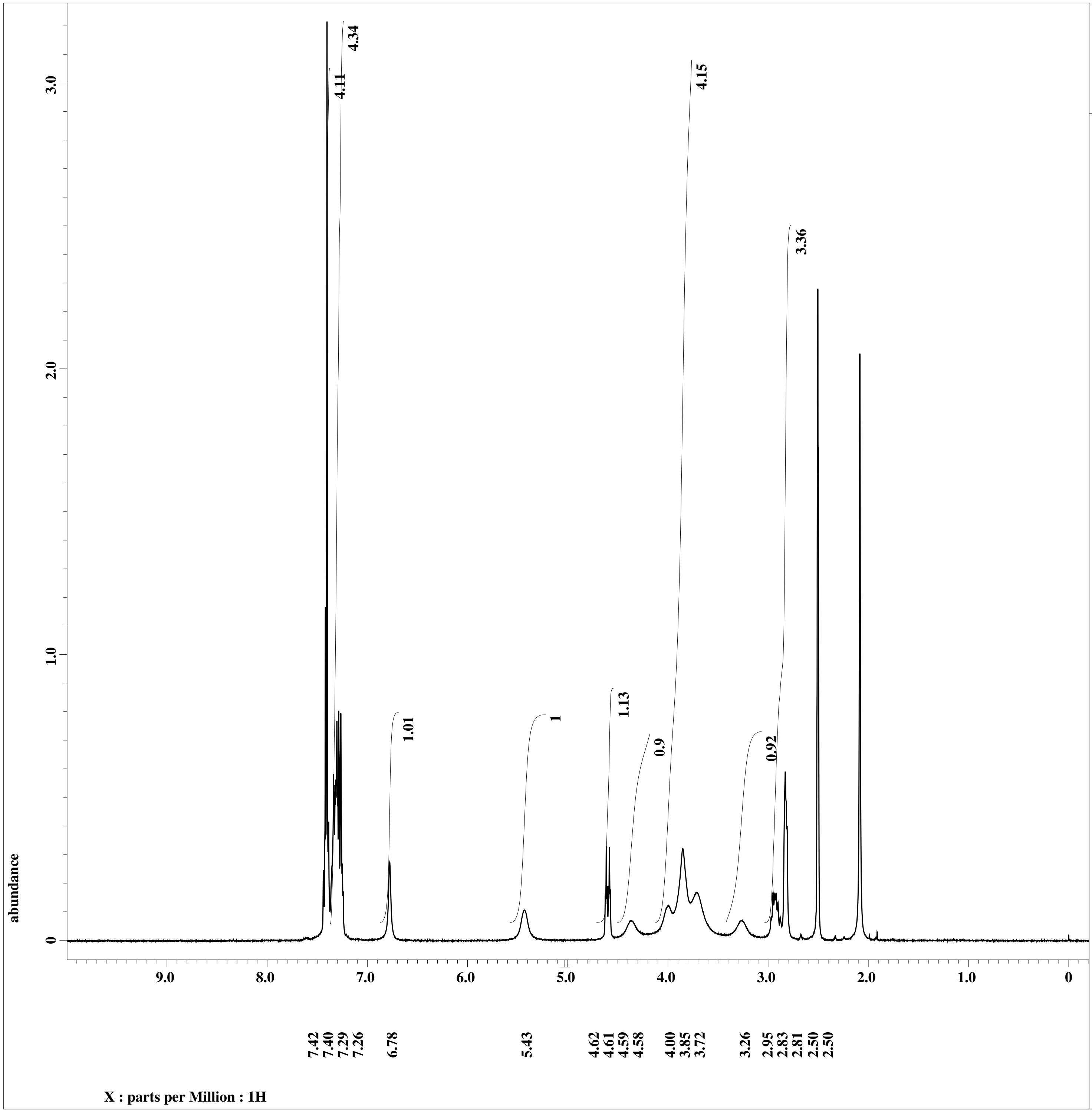


```
Filename           = TAR-II-004 1H NMR Wor
Author             = russell research stud
Experiment         = single_pulse.ex2
Sample_id          = S#734031
Solvent            = DMSO-D6
Creation_time      = 12-OCT-2019 17:58:14
Revision_time      = 22-JUL-2021 10:27:50
Current_time       = 22-JUL-2021 10:30:15

Data_format        = 1D COMPLEX
Dim_size           = 13107
Dim_title          = 1H
Dim_units          = [ppm]
Dimensions         = X
Site               = ECS 400
Spectrometer       = JNM-ECS400

Field_strength     = 9.389766[T] (400[MHz]
X_acq_duration     = 2.18365952[s]
X_domain           = 1H
X_freq             = 399.78219838[MHz]
X_offset           = 5[ppm]
X_points           = 16384
X_prescans         = 1
X_resolution       = 0.45794685[Hz]
X_sweep            = 7.5030012[kHz]
Irr_domain         = 1H
Irr_freq           = 399.78219838[MHz]
Irr_offset         = 5[ppm]
Tri_domain         = 1H
Tri_freq           = 399.78219838[MHz]
Tri_offset         = 5[ppm]
Clipped            = FALSE
Mod_return         = 1
Scans              = 8
Total_scans        = 8

X_90_width         = 14.3[us]
X_acq_time         = 2.18365952[s]
X_angle            = 45[deg]
X_atn              = 3[dB]
X_pulse            = 7.15[us]
Irr_mode           = Off
Tri_mode           = Off
Dante_presat       = FALSE
Initial_wait       = 1[s]
Recvr_gain         = 40
Relaxation_delay   = 5[s]
Repetition_time    = 7.18365952[s]
Temp_get           = 22.7[dC]
```

Figure S83: 13C NMR Spectrum of Compound 43.

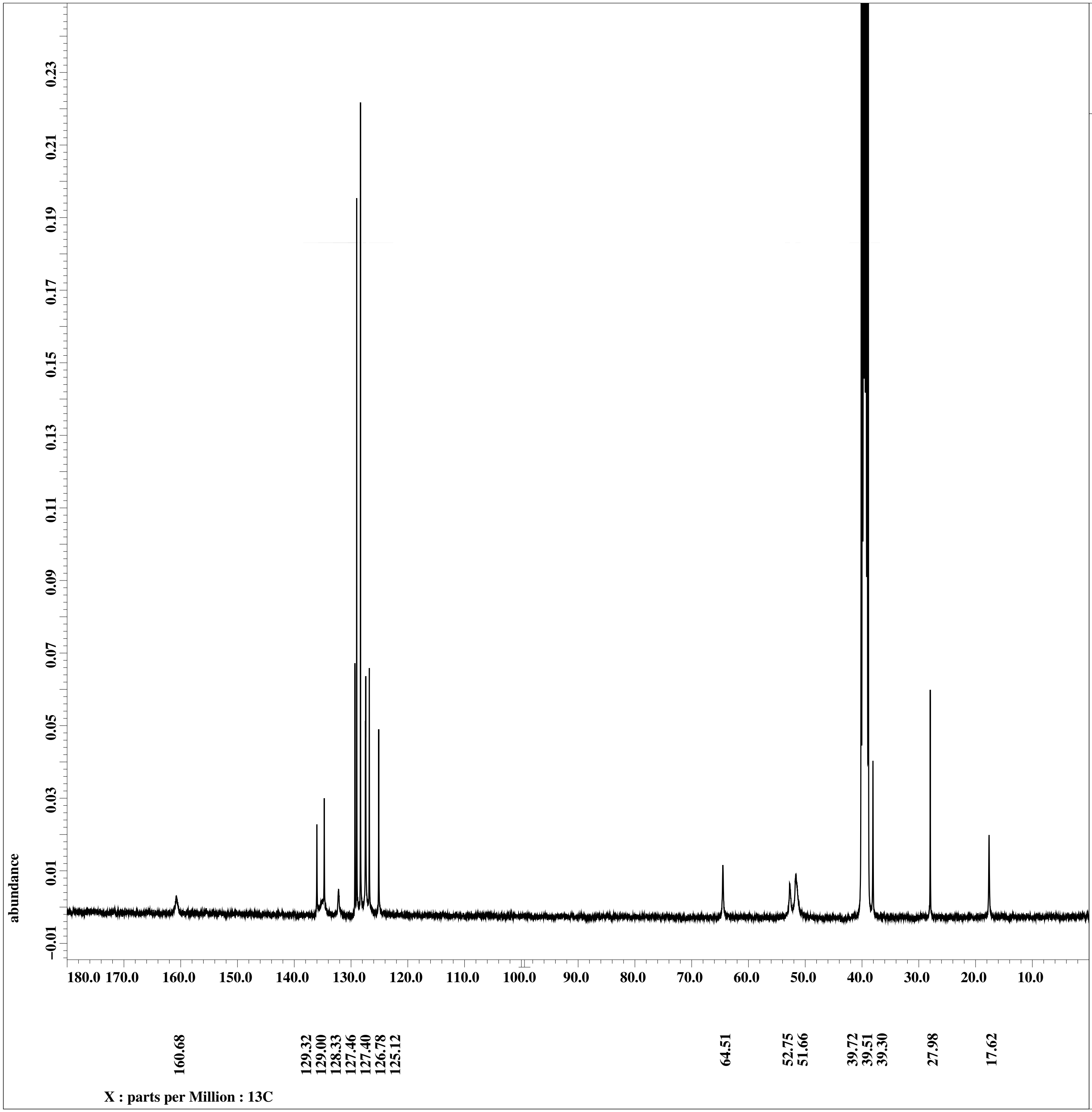


```
Filename            = TAR-II-004 13C NMR Wo
Author              = russell research stud
Experiment          = single_pulse_dec
Sample_id           = S#736763
Solvent             = DMSO-D6
Creation_time       = 13-OCT-2019 21:43:35
Revision_time       = 22-JUL-2021 10:46:15
Current_time        = 22-JUL-2021 10:52:46

Data_format         = 1D COMPLEX
Dim_size            = 26214
Dim_title           = 13C
Dim_units           = [ppm]
Dimensions          = X
Site                = ECS 400
Spectrometer        = JNM-ECS400

Field_strength      = 9.389766[T] (400[MHz]
X_acq_duration      = 1.04333312[s]
X_domain            = 13C
X_freq              = 100.52530333[MHz]
X_offset            = 100[ppm]
X_points            = 32768
X_prescans          = 4
X_resolution        = 0.95846665[Hz]
X_sweep             = 31.40703518[kHz]
Irr_domain          = 1H
Irr_freq            = 399.78219838[MHz]
Irr_offset          = 5[ppm]
Clipped             = FALSE
Mod_return          = 1
Scans               = 32768
Total_scans         = 32768

X_90_width          = 9.82623[us]
X_acq_time          = 1.04333312[s]
X_angle             = 30[deg]
X_atn               = 5.8[dB]
X_pulse             = 3.27541[us]
Irr_atn_dec         = 21[dB]
Irr_atn_noe         = 21[dB]
Irr_noise           = WALTZ
Decoupling          = TRUE
Initial_wait        = 1[s]
Noe                 = TRUE
Noe_time            = 2[s]
Recvr_gain          = 60
Relaxation_delay    = 2[s]
Repetition_time     = 3.04333312[s]
Temp_get            = 23.1[dC]
```

Figure S84: 1H NMR Spectrum of Compound 44.

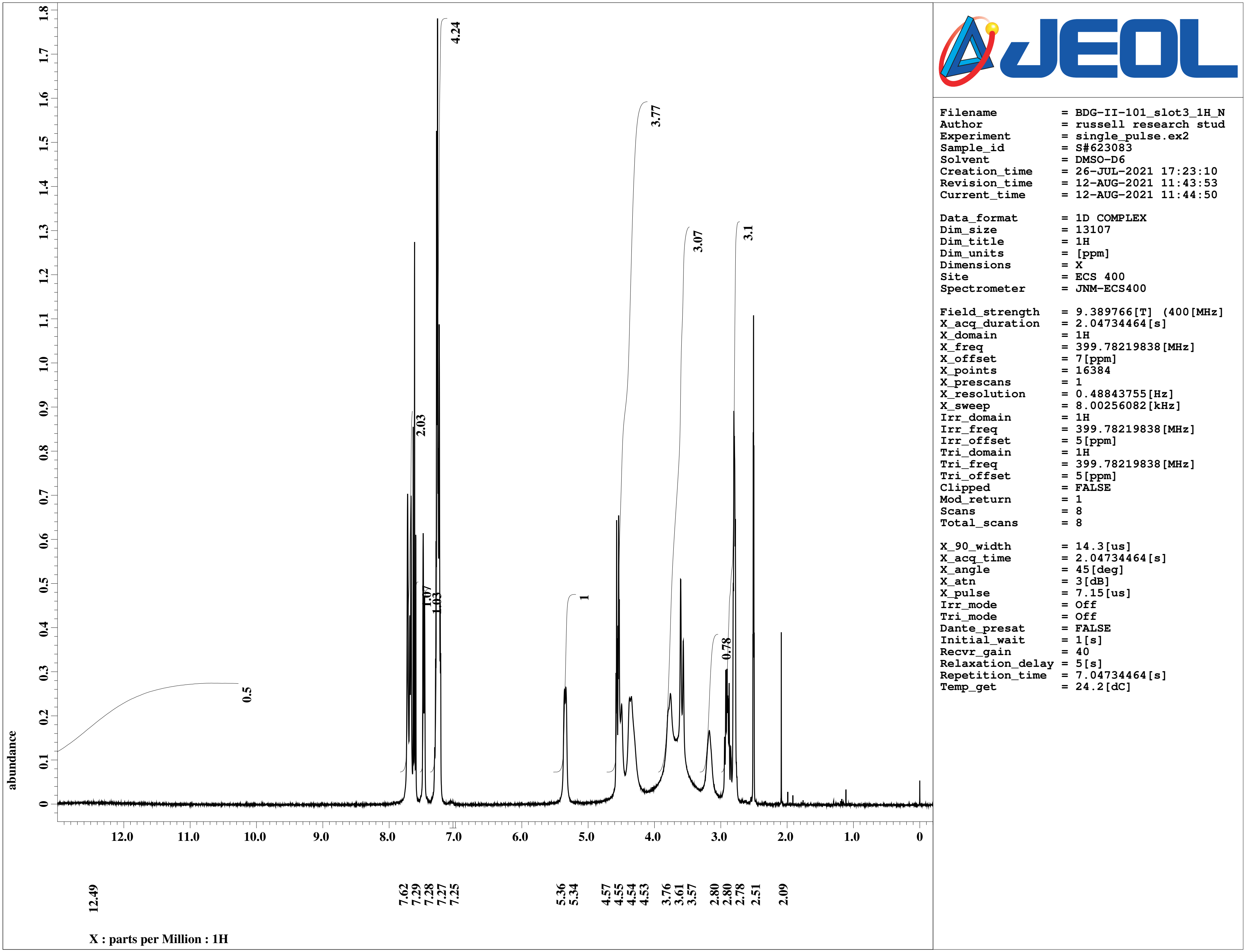

Figure S85: 13C NMR Spectrum of Compound 44.

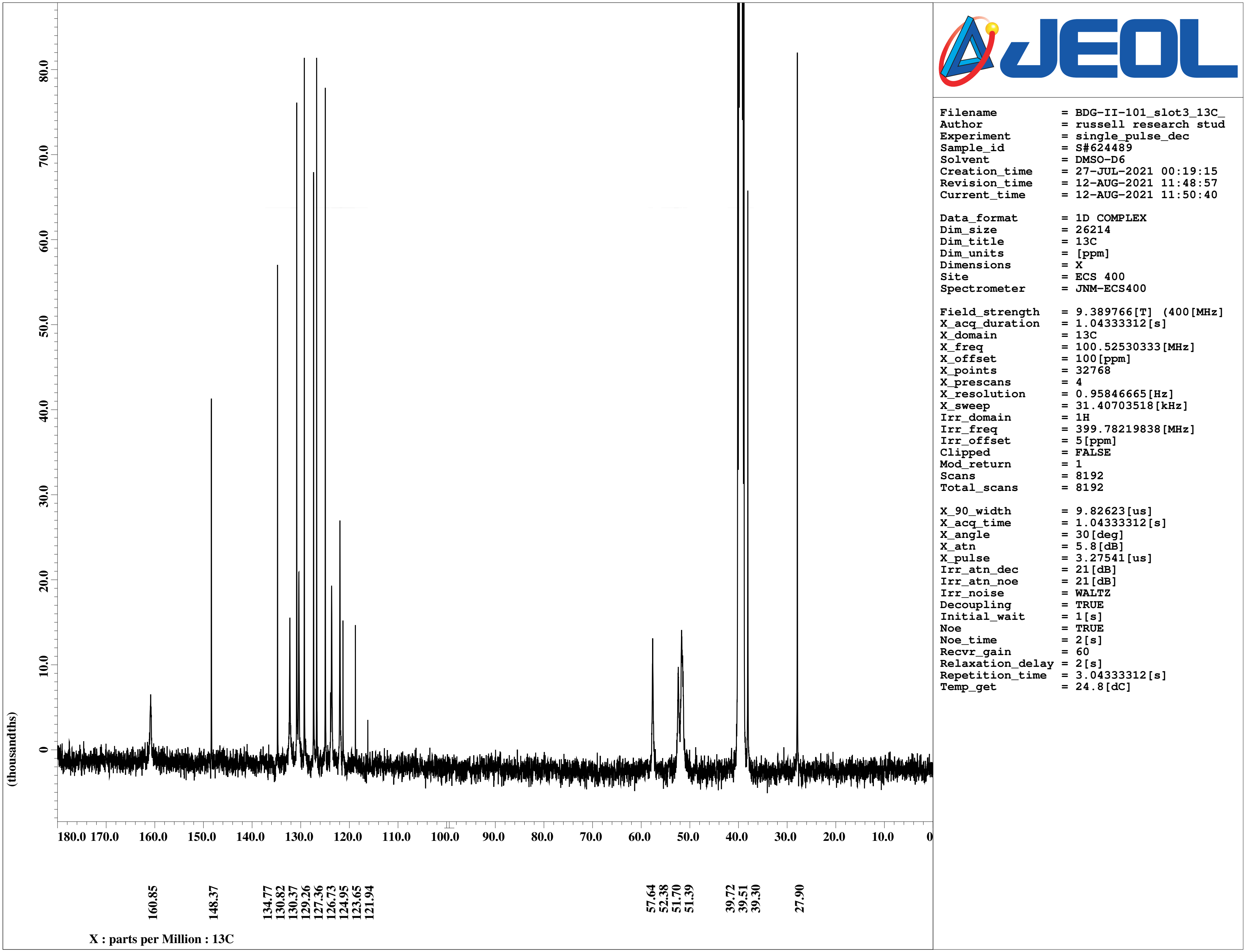

Figure S86: 1H NMR Spectrum of Compound 45.

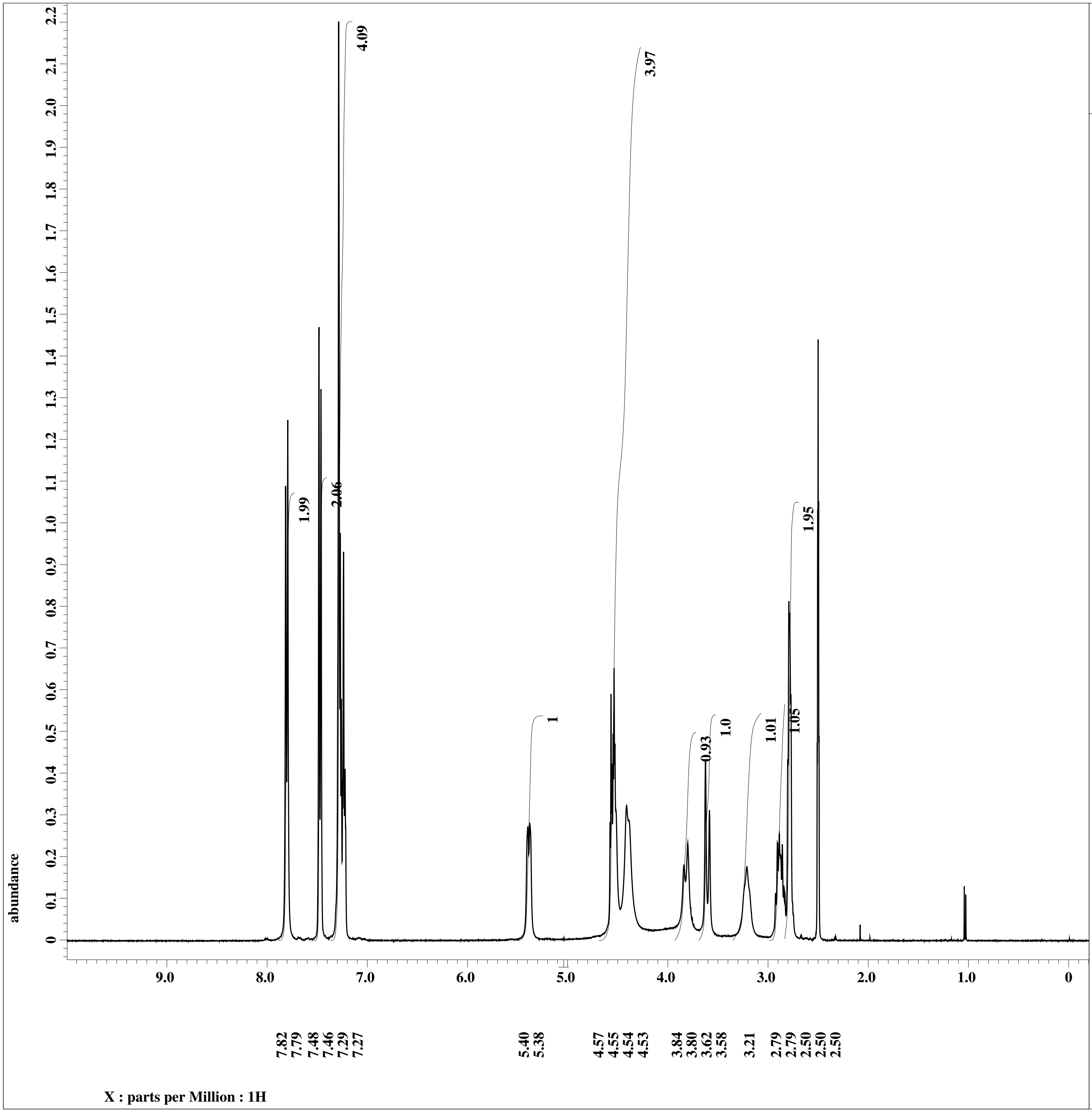


```
Filename           = SMW-1-002 crystals co
Author             = chemlabs
Experiment         = single_pulse.ex2
Sample_id          = 001
Solvent            = DMSO-D6
Creation_time      = 20-JUL-2017 08:30:58
Revision_time      = 22-JUL-2021 11:13:03
Current_time       = 22-JUL-2021 11:21:35

Comment            = single_pulse
Data_format        = 1D COMPLEX
Dim_size           = 13107
Dim_title          = 1H
Dim_units          = [ppm]
Dimensions         = X
Site               = ECS 400
Spectrometer       = JNM-ECS400

Field_strength     = 9.389766[T] (400[MHz]
X_acq_duration     = 2.18365952[s]
X_domain           = 1H
X_freq             = 399.78219838[MHz]
X_offset           = 5[ppm]
X_points           = 16384
X_prescans         = 1
X_resolution       = 0.45794685[Hz]
X_sweep            = 7.5030012[kHz]
Irr_domain         = 1H
Irr_freq           = 399.78219838[MHz]
Irr_offset         = 5[ppm]
Tri_domain         = 1H
Tri_freq           = 399.78219838[MHz]
Tri_offset         = 5[ppm]
Clipped            = FALSE
Mod_return         = 1
Scans              = 8
Total_scans        = 8

X_90_width         = 14.3[us]
X_acq_time         = 2.18365952[s]
X_angle            = 45[deg]
X_atn              = 3[dB]
X_pulse            = 7.15[us]
Irr_mode           = Off
Tri_mode           = Off
Dante_presat       = FALSE
Initial_wait       = 1[s]
Recvr_gain         = 36
Relaxation_delay   = 5[s]
Repetition_time    = 7.18365952[s]
Temp_get           = 25.1[dC]
```

Figure S87: 13C NMR Spectrum of Compound 45.

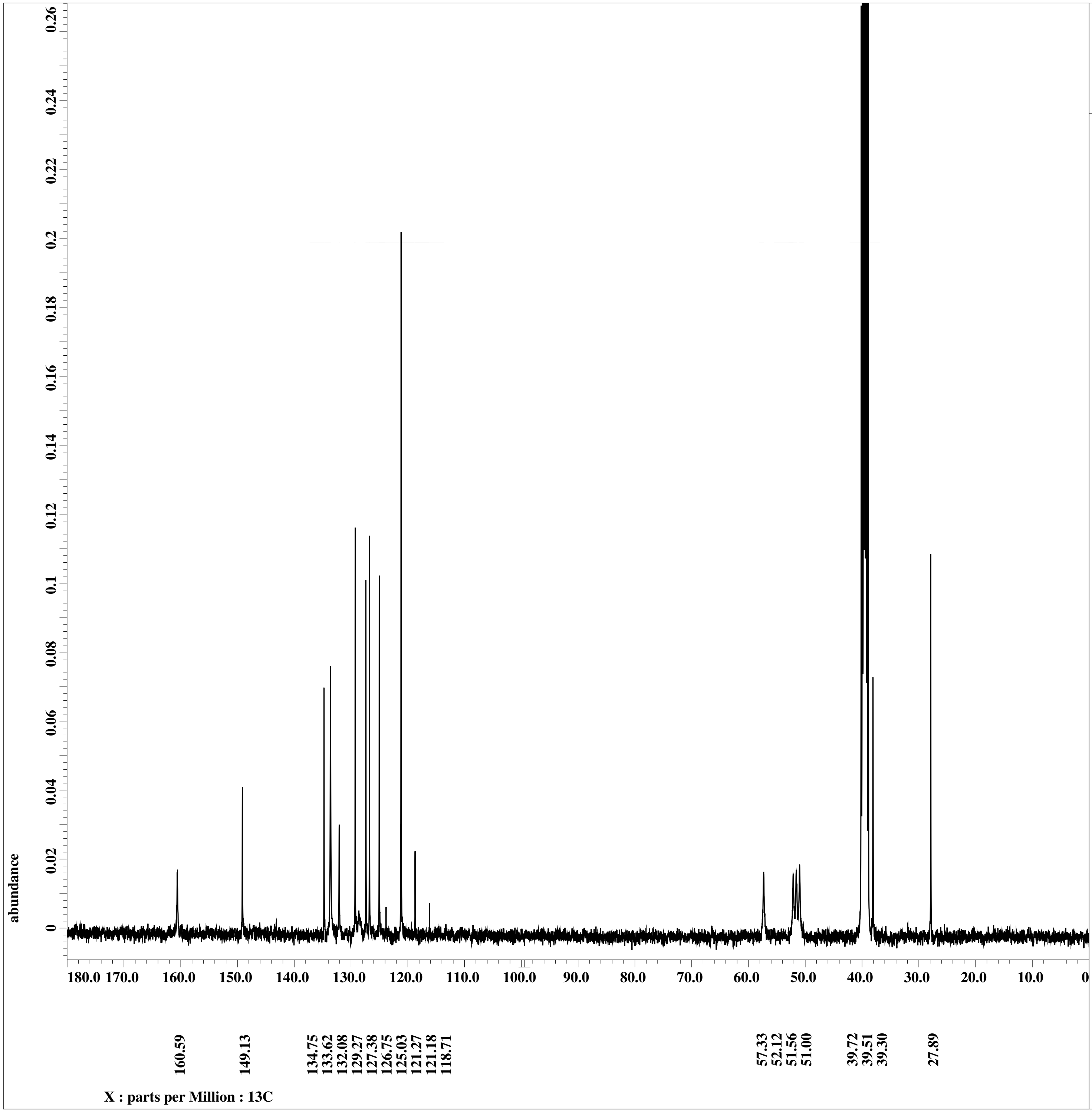

JEOL

```
Filename          = SMW-1-002 crystals co
Author            = chemlabs
Experiment        = single_pulse_dec
Sample_id         = 001
Solvent           = DMSO-D6
Creation_time     = 20-JUL-2017 12:01:14
Revision_time     = 22-JUL-2021 11:25:53
Current_time      = 22-JUL-2021 11:30:38

Comment           = single pulse decouple
Data_format       = 1D COMPLEX
Dim_size          = 26214
Dim_title         = 13C
Dim_units         = [ppm]
Dimensions        = X
Site              = ECS 400
Spectrometer      = JNM-ECS400

Field_strength    = 9.389766[T] (400[MHz]
X_acq_duration    = 1.04333312[s]
X_domain          = 13C
X_freq            = 100.52530333[MHz]
X_offset          = 100[ppm]
X_points          = 32768
X_prescans        = 4
X_resolution      = 0.95846665[Hz]
X_sweep           = 31.40703518[kHz]
Irr_domain        = 1H
Irr_freq          = 399.78219838[MHz]
Irr_offset        = 5[ppm]
Clipped           = FALSE
Mod_return        = 1
Scans             = 4098
Total_scans       = 4098

X_90_width        = 9.82623[us]
X_acq_time        = 1.04333312[s]
X_angle           = 30[deg]
X_atn             = 5.8[dB]
X_pulse           = 3.27541[us]
Irr_atn_dec       = 21[dB]
Irr_atn_noe       = 21[dB]
Irr_noise         = WALTZ
Decoupling        = TRUE
Initial_wait      = 1[s]
Noe               = TRUE
Noe_time          = 2[s]
Recvr_gain        = 60
Relaxation_delay  = 2[s]
Repetition_time   = 3.04333312[s]
Temp_get          = 25.2[dC]
```

Figure S88: 1H NMR Spectrum of Compound 46.

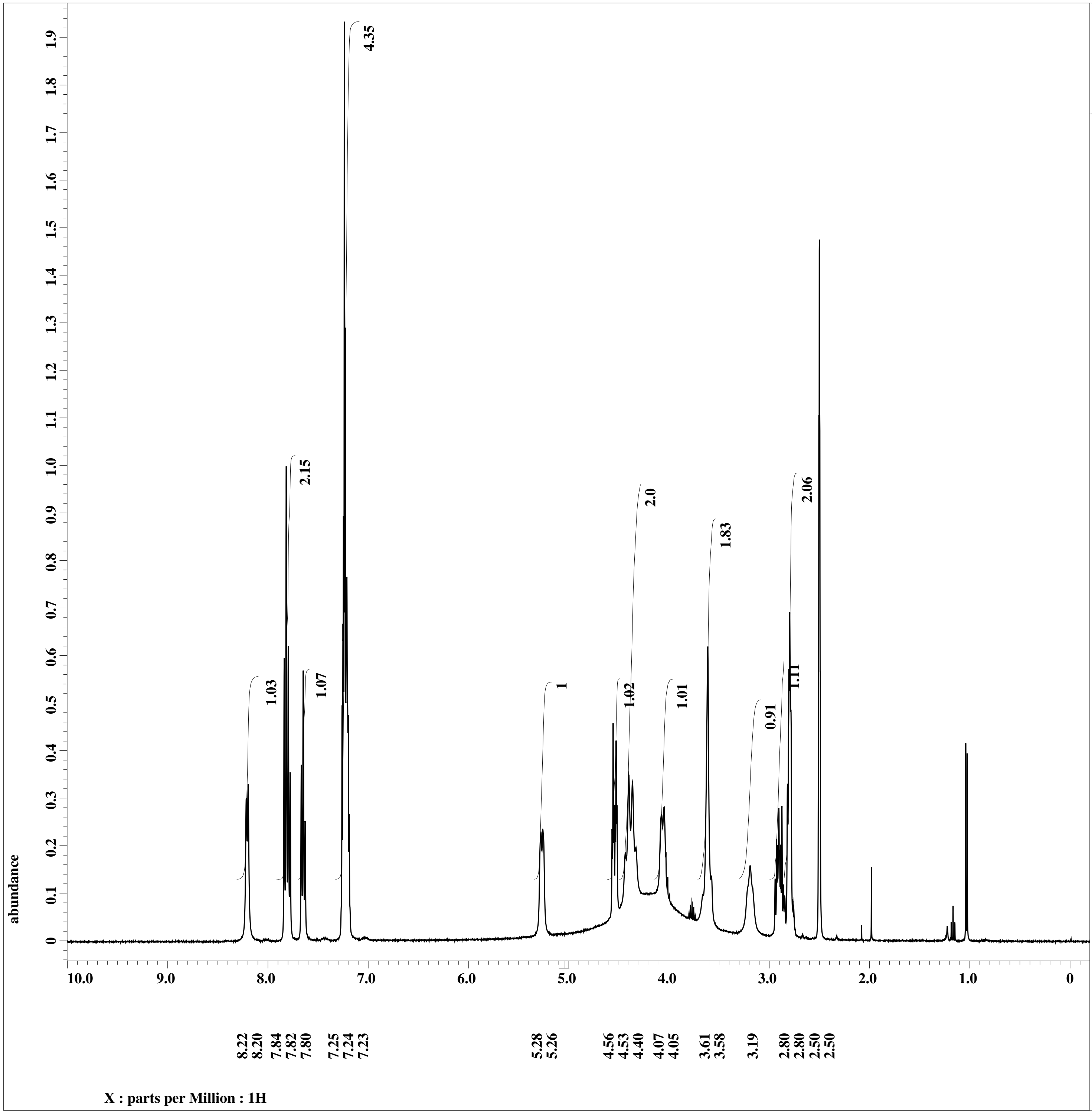

Filename	= RDK-I-012 crystalsmor
Author	= chemlabs
Experiment	= single_pulse.ex2
Sample_id	= 001
Solvent	= DMSO-D6
Creation_time	= 14-JUL-2017 16:01:12
Revision_time	= 22-JUL-2021 11:36:37
Current_time	= 22-JUL-2021 11:42:13
Comment	= single_pulse
Data_format	= 1D COMPLEX
Dim_size	= 13107
Dim_title	= 1H
Dim_units	= [ppm]
Dimensions	= X
Site	= ECS 400
Spectrometer	= JNM-ECS400
Field_strength	= 9.389766[T] (400[MHz])
X_acq_duration	= 2.18365952[s]
X_domain	= 1H
X_freq	= 399.78219838[MHz]
X_offset	= 5[ppm]
X_points	= 16384
X_prescans	= 1
X_resolution	= 0.45794685[Hz]
X_sweep	= 7.5030012[kHz]
Irr_domain	= 1H
Irr_freq	= 399.78219838[MHz]
Irr_offset	= 5[ppm]
Tri_domain	= 1H
Tri_freq	= 399.78219838[MHz]
Tri_offset	= 5[ppm]
Clipped	= FALSE
Mod_return	= 1
Scans	= 8
Total_scans	= 8
X_90_width	= 12.95[us]
X_acq_time	= 2.18365952[s]
X_angle	= 45[deg]
X_atn	= 3[dB]
X_pulse	= 6.475[us]
Irr_mode	= Off
Tri_mode	= Off
Dante_presat	= FALSE
Initial_wait	= 1[s]
Recvr_gain	= 38
Relaxation_delay	= 5[s]
Repetition_time	= 7.18365952[s]
Temp_get	= 25.6[dC]

Figure S89: 13C NMR Spectrum of Compound 46.

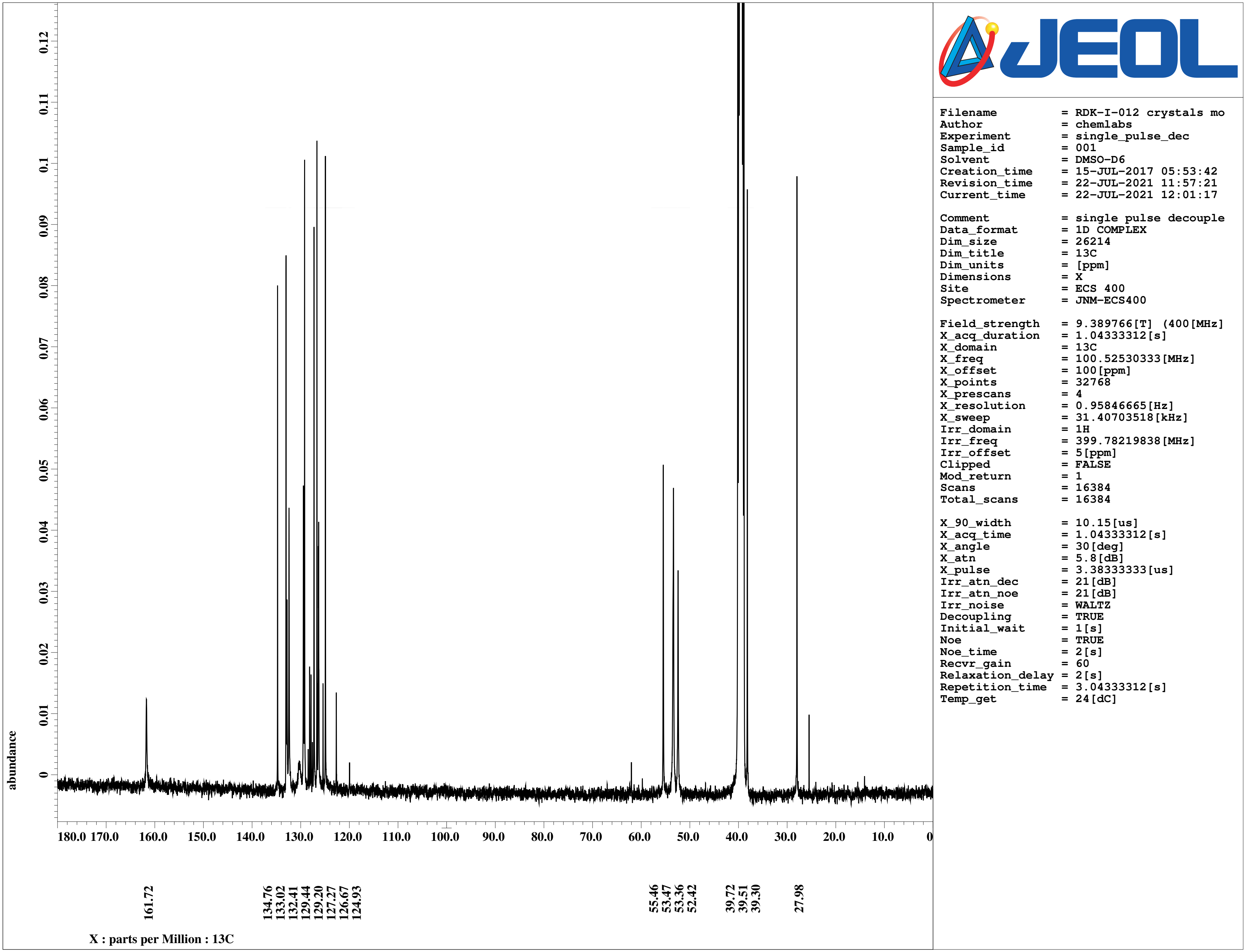

Figure S90: 1H NMR Spectrum of Compound 47.

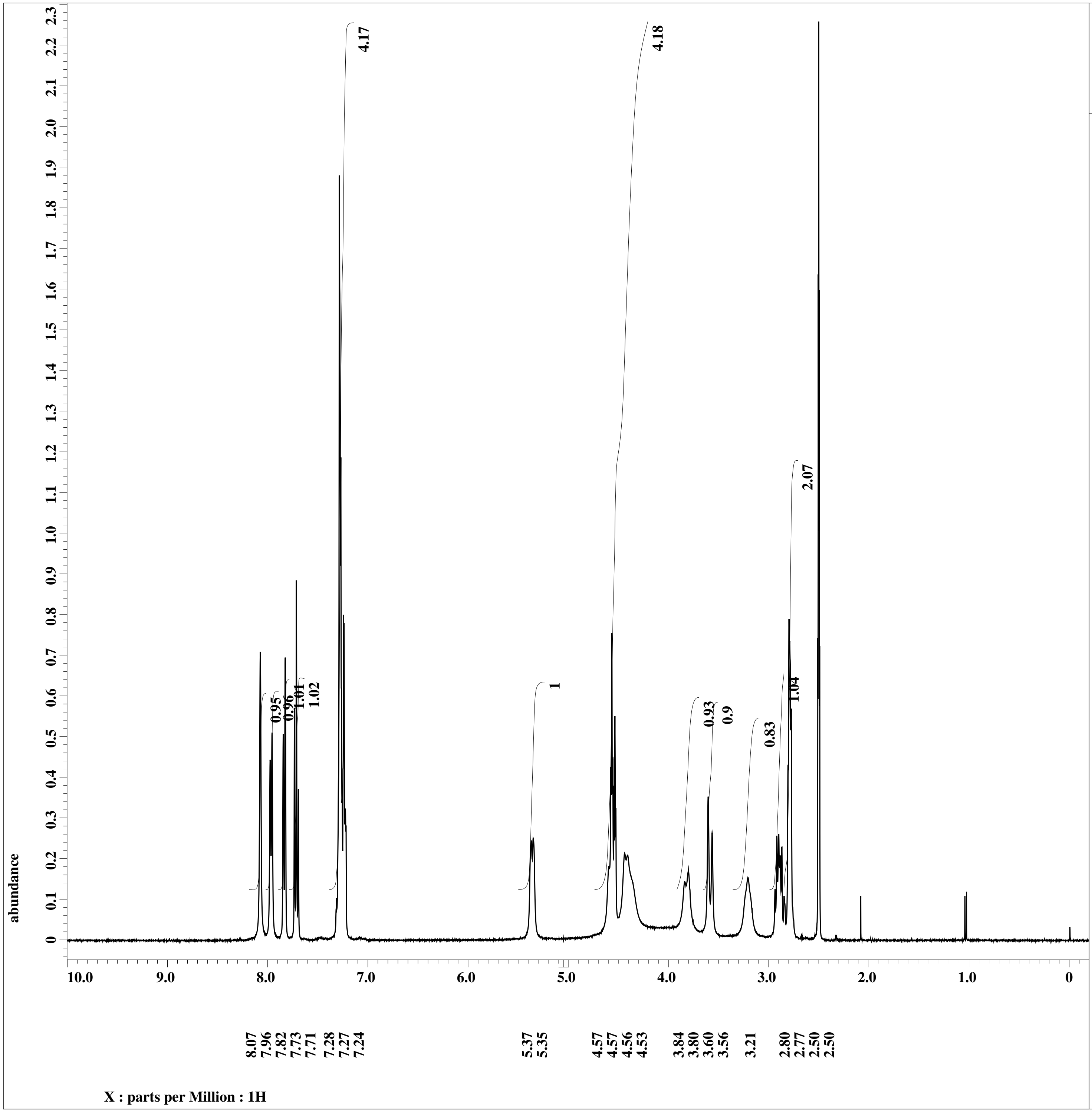


```
Filename           = RDK-I-011 concentrate
Author             = chemlabs
Experiment         = single_pulse.ex2
Sample_id          = 001
Solvent            = DMSO-D6
Creation_time      = 13-JUL-2017 15:22:37
Revision_time      = 22-JUL-2021 12:37:26
Current_time       = 22-JUL-2021 12:40:55

Comment            = single_pulse
Data_format        = 1D COMPLEX
Dim_size           = 13107
Dim_title          = 1H
Dim_units          = [ppm]
Dimensions         = X
Site               = ECS 400
Spectrometer       = JNM-ECS400

Field_strength     = 9.389766[T] (400[MHz]
X_acq_duration     = 2.18365952[s]
X_domain           = 1H
X_freq             = 399.78219838[MHz]
X_offset           = 5[ppm]
X_points           = 16384
X_prescans         = 1
X_resolution       = 0.45794685[Hz]
X_sweep            = 7.5030012[kHz]
Irr_domain         = 1H
Irr_freq           = 399.78219838[MHz]
Irr_offset         = 5[ppm]
Tri_domain         = 1H
Tri_freq           = 399.78219838[MHz]
Tri_offset         = 5[ppm]
Clipped            = FALSE
Mod_return         = 1
Scans              = 8
Total_scans        = 8

X_90_width         = 12.95[us]
X_acq_time         = 2.18365952[s]
X_angle            = 45[deg]
X_atn              = 3[dB]
X_pulse            = 6.475[us]
Irr_mode           = Off
Tri_mode           = Off
Dante_presat       = FALSE
Initial_wait       = 1[s]
Recvr_gain         = 40
Relaxation_delay   = 5[s]
Repetition_time    = 7.18365952[s]
Temp_get           = 24.3[dC]
```

Figure S91: 13C NMR Spectrum of Compound 47.

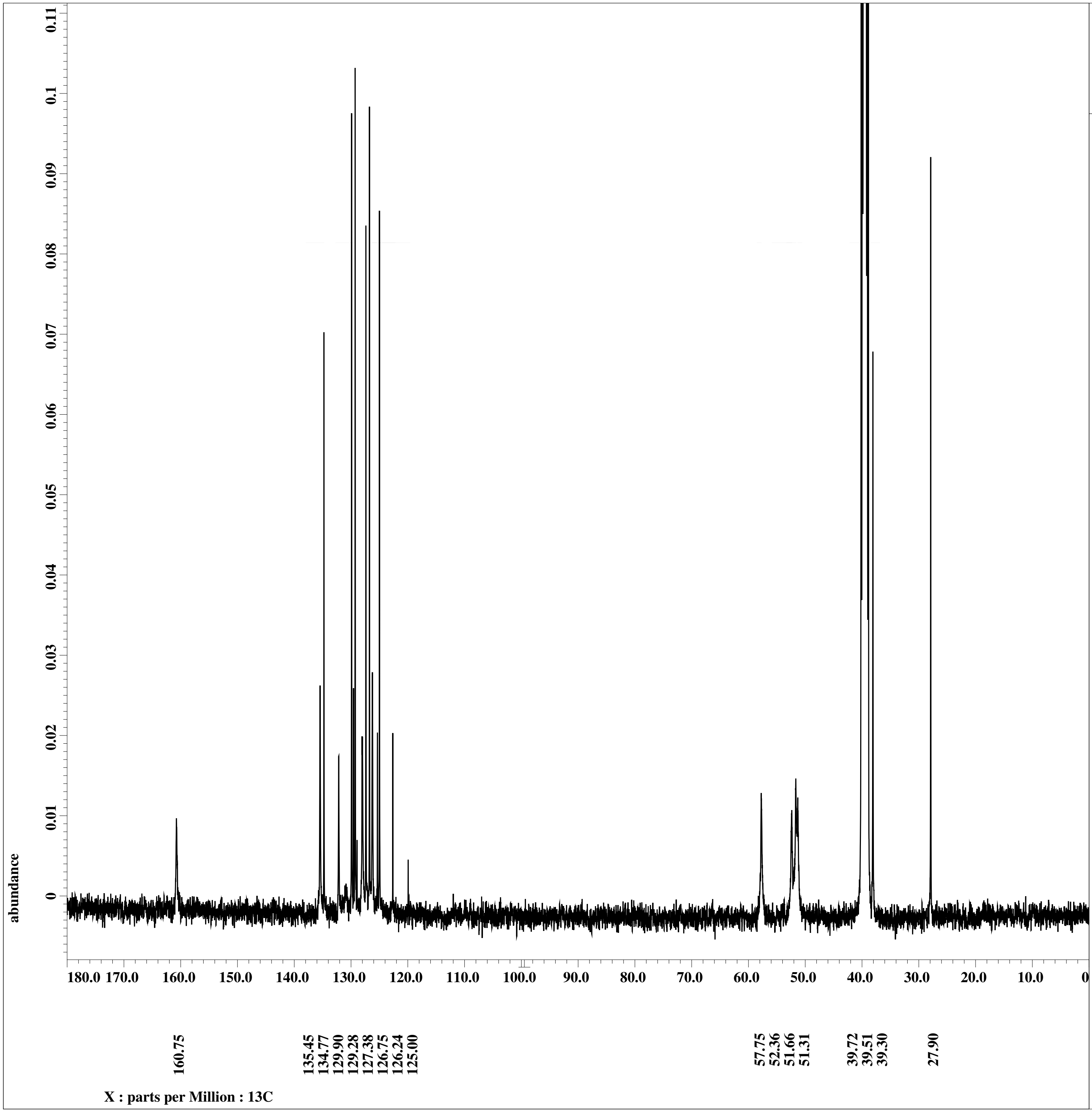

JEOL

Filename	= RDK-I-011 concentrate
Author	= chemlabs
Experiment	= single_pulse_dec
Sample_id	= 001
Solvent	= DMSO-D6
Creation_time	= 13-JUL-2017 22:20:36
Revision_time	= 22-JUL-2021 12:51:38
Current_time	= 22-JUL-2021 12:52:53
Comment	= single pulse decouple
Data_format	= 1D COMPLEX
Dim_size	= 26214
Dim_title	= 13C
Dim_units	= [ppm]
Dimensions	= X
Site	= ECS 400
Spectrometer	= JNM-ECS400
Field_strength	= 9.389766[T] (400[MHz]
X_acq_duration	= 1.04333312[s]
X_domain	= 13C
X_freq	= 100.52530333[MHz]
X_offset	= 100[ppm]
X_points	= 32768
X_prescans	= 4
X_resolution	= 0.95846665[Hz]
X_sweep	= 31.40703518[kHz]
Irr_domain	= 1H
Irr_freq	= 399.78219838[MHz]
Irr_offset	= 5[ppm]
Clipped	= TRUE
Mod_return	= 1
Scans	= 8192
Total_scans	= 8192
X_90_width	= 10.15[us]
X_acq_time	= 1.04333312[s]
X_angle	= 30[deg]
X_atn	= 5.8[dB]
X_pulse	= 3.38333333[us]
Irr_atn_dec	= 21[dB]
Irr_atn_noe	= 21[dB]
Irr_noise	= WALTZ
Decoupling	= TRUE
Initial_wait	= 1[s]
Noe	= TRUE
Noe_time	= 2[s]
Recvr_gain	= 60
Relaxation_delay	= 2[s]
Repetition_time	= 3.04333312[s]
Temp_get	= 23.9[dC]

Figure S92: 1H NMR Spectrum of Compound 48.

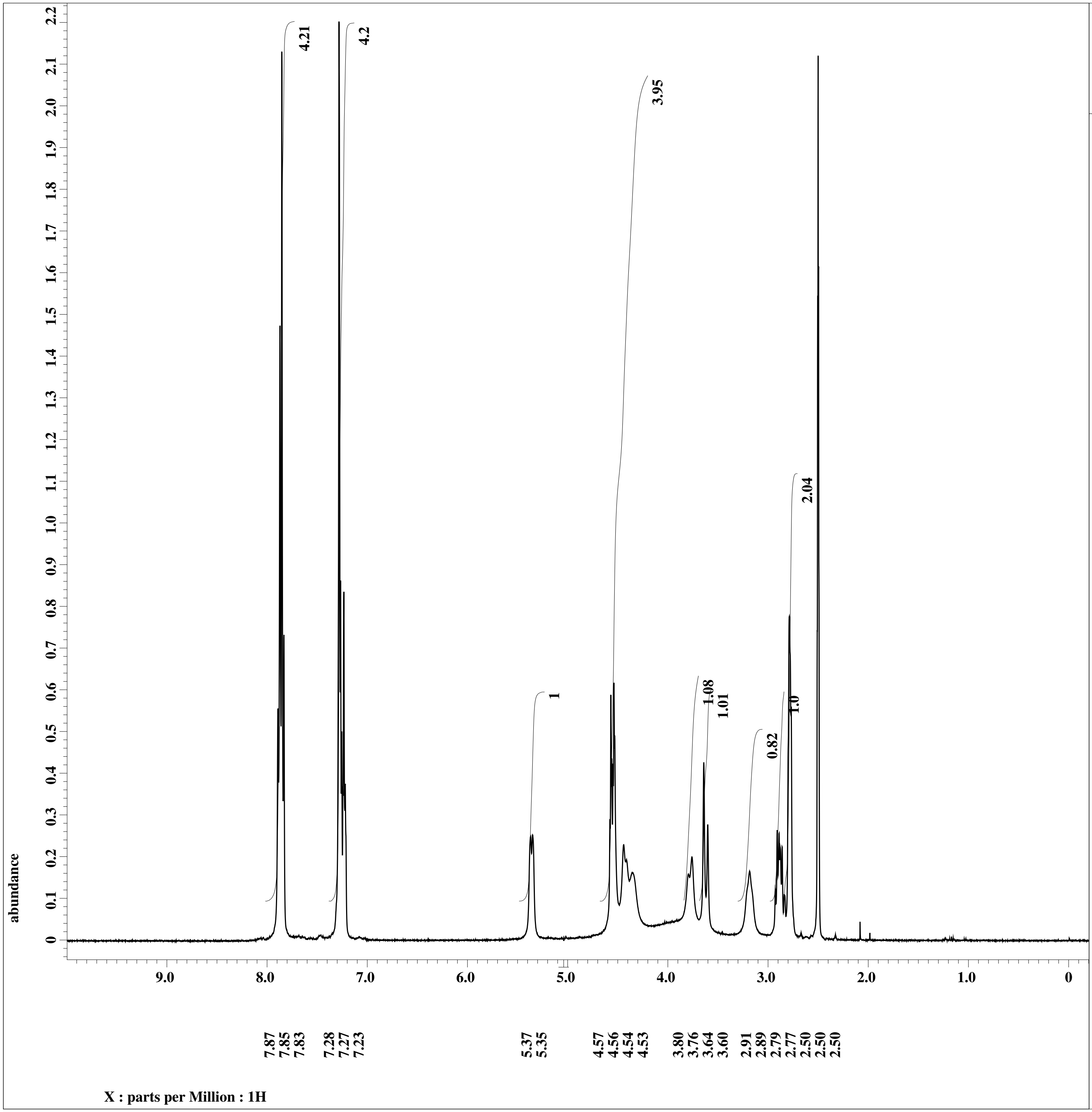

Filename	= SMW-1-012 crystals co
Author	= chemlabs
Experiment	= single_pulse.ex2
Sample_id	= 001
Solvent	= DMSO-D6
Creation_time	= 19-JUL-2017 14:28:43
Revision_time	= 22-JUL-2021 12:55:43
Current_time	= 22-JUL-2021 13:00:53
Comment	= single_pulse
Data_format	= 1D COMPLEX
Dim_size	= 13107
Dim_title	= 1H
Dim_units	= [ppm]
Dimensions	= X
Site	= ECS 400
Spectrometer	= JNM-ECS400
Field_strength	= 9.389766[T] (400[MHz]
X_acq_duration	= 2.18365952[s]
X_domain	= 1H
X_freq	= 399.78219838[MHz]
X_offset	= 5[ppm]
X_points	= 16384
X_prescans	= 1
X_resolution	= 0.45794685[Hz]
X_sweep	= 7.5030012[kHz]
Irr_domain	= 1H
Irr_freq	= 399.78219838[MHz]
Irr_offset	= 5[ppm]
Tri_domain	= 1H
Tri_freq	= 399.78219838[MHz]
Tri_offset	= 5[ppm]
Clipped	= FALSE
Mod_return	= 1
Scans	= 8
Total_scans	= 8
X_90_width	= 14.3[us]
X_acq_time	= 2.18365952[s]
X_angle	= 45[deg]
X_atn	= 3[dB]
X_pulse	= 7.15[us]
Irr_mode	= Off
Tri_mode	= Off
Dante_presat	= FALSE
Initial_wait	= 1[s]
Recvr_gain	= 38
Relaxation_delay	= 5[s]
Repetition_time	= 7.18365952[s]
Temp_get	= 24.7[dC]

Figure S93: 13C NMR Spectrum of Compound 48.

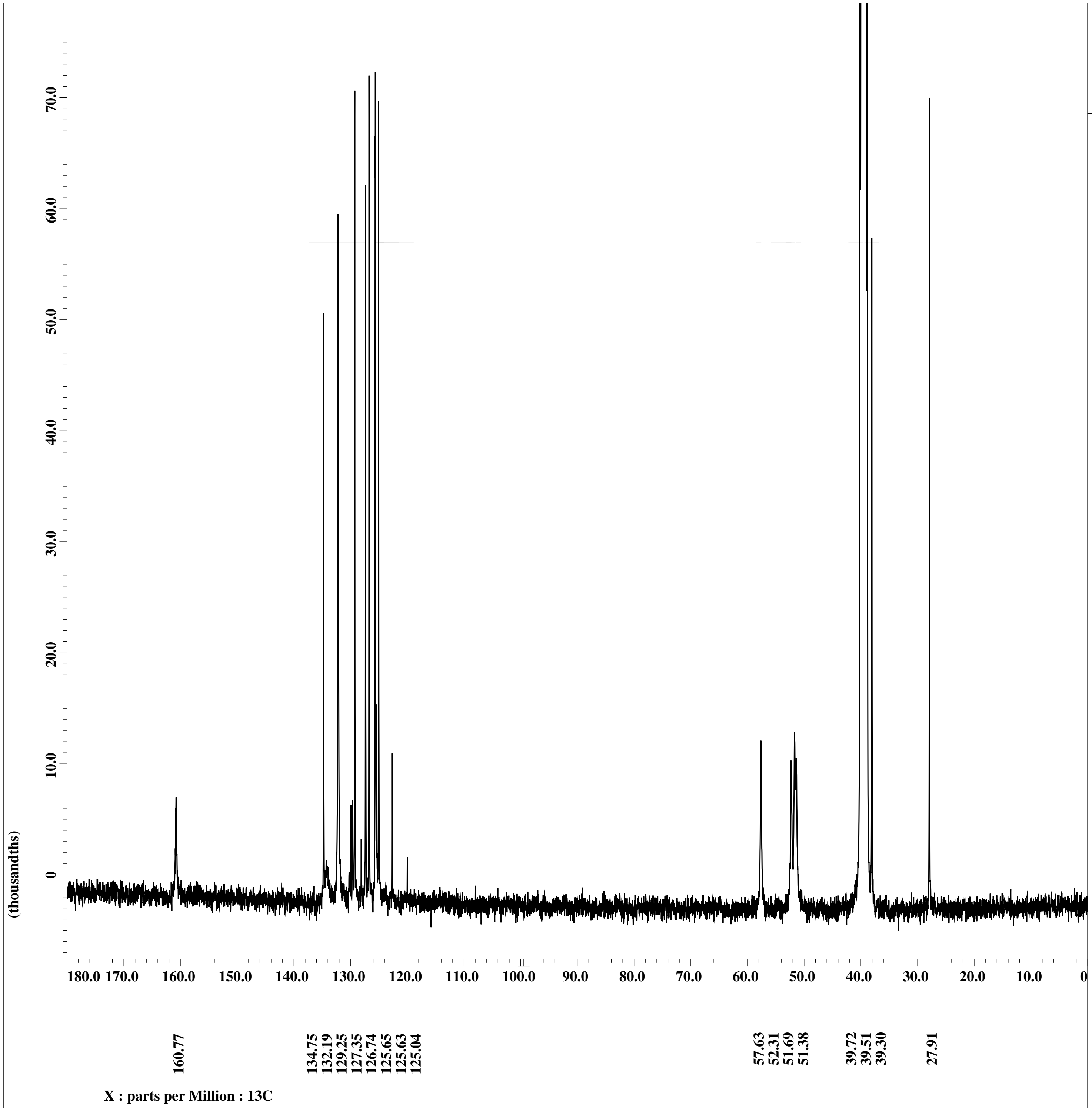

Filename	= SMW-1-012 crystals co
Author	= chemlabs
Experiment	= single_pulse_dec
Sample_id	= 001
Solvent	= DMSO-D6
Creation_time	= 20-JUL-2017 04:23:26
Revision_time	= 22-JUL-2021 13:03:41
Current_time	= 22-JUL-2021 13:05:18
Comment	= single pulse decouple
Data_format	= 1D COMPLEX
Dim_size	= 26214
Dim_title	= 13C
Dim_units	= [ppm]
Dimensions	= X
Site	= ECS 400
Spectrometer	= JNM-ECS400
Field_strength	= 9.389766[T] (400[MHz]
X_acq_duration	= 1.04333312[s]
X_domain	= 13C
X_freq	= 100.52530333[MHz]
X_offset	= 100[ppm]
X_points	= 32768
X_prescans	= 4
X_resolution	= 0.95846665[Hz]
X_sweep	= 31.40703518[kHz]
Irr_domain	= 1H
Irr_freq	= 399.78219838[MHz]
Irr_offset	= 5[ppm]
Clipped	= FALSE
Mod_return	= 1
Scans	= 16384
Total_scans	= 16384
X_90_width	= 9.82623[us]
X_acq_time	= 1.04333312[s]
X_angle	= 30[deg]
X_atn	= 5.8[dB]
X_pulse	= 3.27541[us]
Irr_atn_dec	= 21[dB]
Irr_atn_noe	= 21[dB]
Irr_noise	= WALTZ
Decoupling	= TRUE
Initial_wait	= 1[s]
Noe	= TRUE
Noe_time	= 2[s]
Recvr_gain	= 60
Relaxation_delay	= 2[s]
Repetition_time	= 3.04333312[s]
Temp_get	= 24.2[dC]

Figure S94: 1H NMR Spectrum of Compound 49.

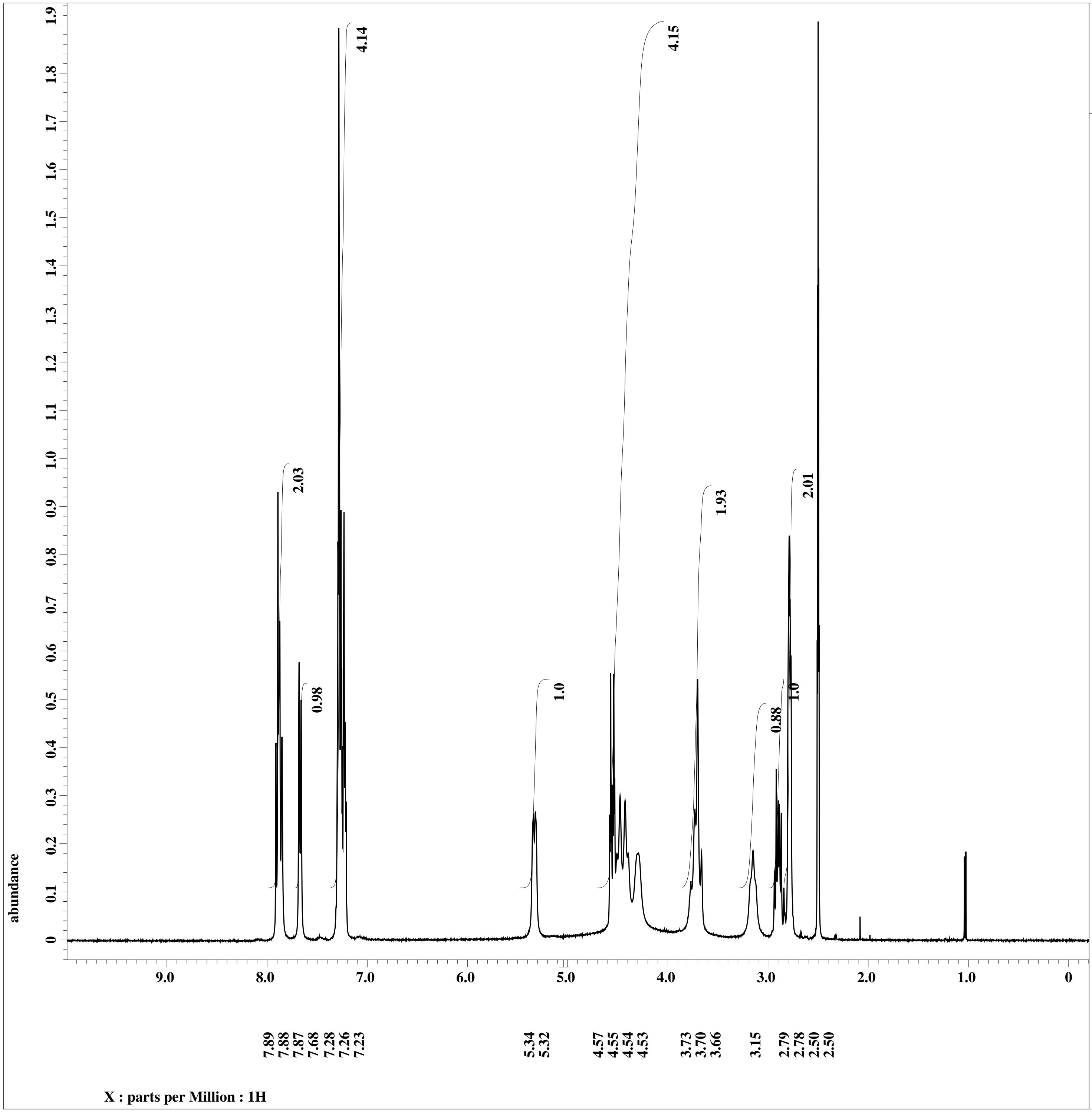

Filename	=	RDK-I-014 1H NMR DMSO
Author	=	chemlabs
Experiment	=	single_pulse.ex2
Sample_id	=	001
Solvent	=	DMSO-D6
Creation_time	=	20-JUL-2017 13:52:04
Revision_time	=	22-JUL-2021 13:09:31
Current_time	=	22-JUL-2021 13:14:17
Comment	=	single_pulse
Data_format	=	1D COMPLEX
Dim_size	=	13107
Dim_title	=	1H
Dim_units	=	[ppm]
Dimensions	=	X
Site	=	ECS 400
Spectrometer	=	JNM-ECS400
Field_strength	=	9.389766[T] (400[MHz]
X_acq_duration	=	2.18365952[s]
X_domain	=	1H
X_freq	=	399.78219838[MHz]
X_offset	=	5[ppm]
X_points	=	16384
X_prescans	=	1
X_resolution	=	0.45794685[Hz]
X_sweep	=	7.5030012[kHz]
Irr_domain	=	1H
Irr_freq	=	399.78219838[MHz]
Irr_offset	=	5[ppm]
Tri_domain	=	1H
Tri_freq	=	399.78219838[MHz]
Tri_offset	=	5[ppm]
Clipped	=	FALSE
Mod_return	=	1
Scans	=	8
Total_scans	=	8
X_90_width	=	14.3[us]
X_acq_time	=	2.18365952[s]
X_angle	=	45[deg]
X_atn	=	3[dB]
X_pulse	=	7.15[us]
Irr_mode	=	Off
Tri_mode	=	Off
Dante_presat	=	FALSE
Initial_wait	=	1[s]
Recvr_gain	=	38
Relaxation_delay	=	5[s]
Repetition_time	=	7.18365952[s]
Temp_get	=	24.6[dC]

Figure S95: 13C NMR Spectrum of Compound 49.

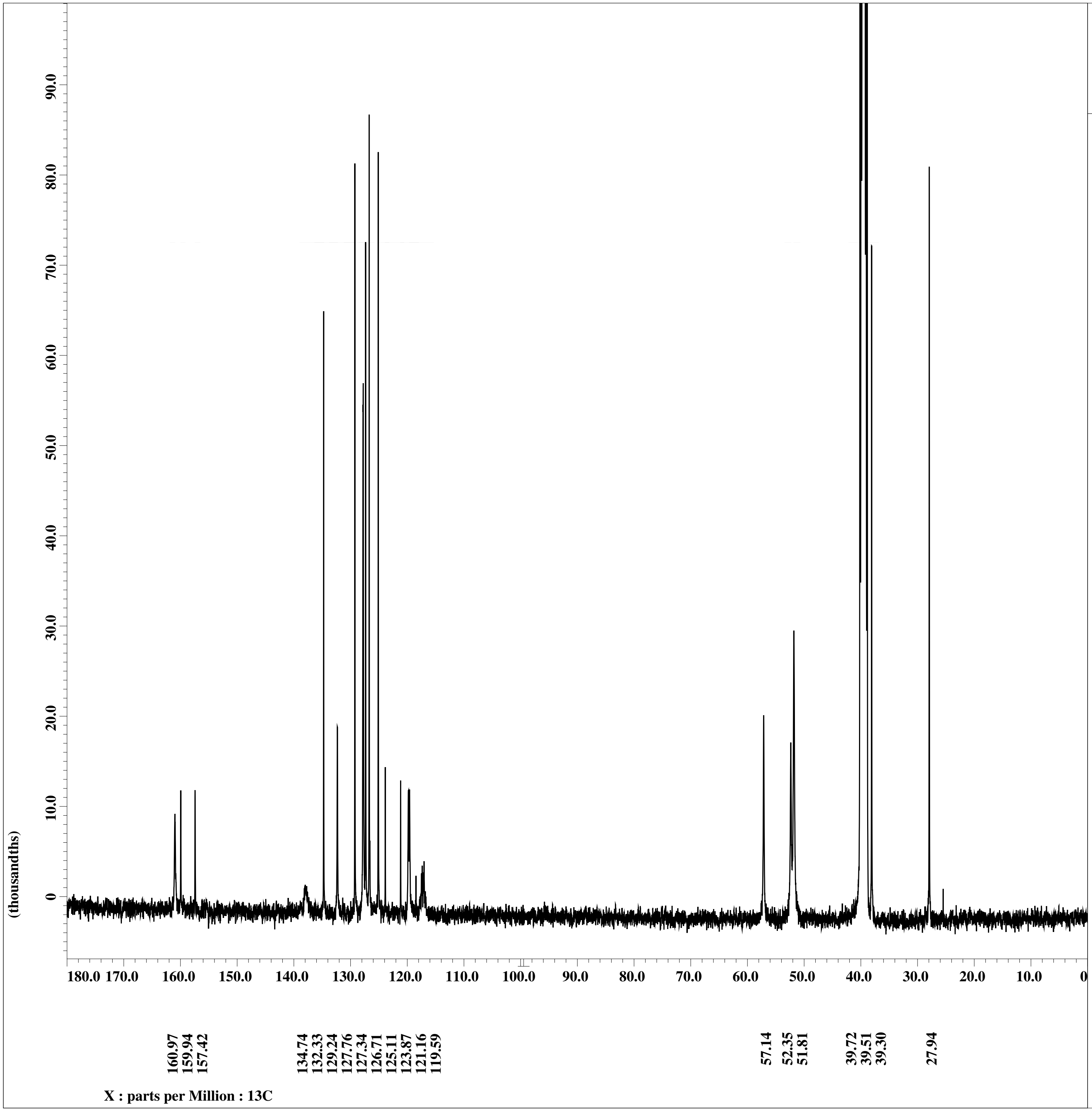


```
Filename          = RDK-I-014 concentrate
Author            = chemlabs
Experiment        = single_pulse_dec
Sample_id         = 001
Solvent           = DMSO-D6
Creation_time     = 21-JUL-2017 03:45:33
Revision_time     = 22-JUL-2021 13:19:37
Current_time      = 22-JUL-2021 13:21:05

Comment           = single pulse decouple
Data_format       = 1D COMPLEX
Dim_size          = 26214
Dim_title         = 13C
Dim_units         = [ppm]
Dimensions        = X
Site              = ECS 400
Spectrometer      = JNM-ECS400

Field_strength    = 9.389766[T] (400[MHz]
X_acq_duration    = 1.04333312[s]
X_domain          = 13C
X_freq            = 100.52530333[MHz]
X_offset          = 100[ppm]
X_points          = 32768
X_prescans        = 4
X_resolution      = 0.95846665[Hz]
X_sweep           = 31.40703518[kHz]
Irr_domain        = 1H
Irr_freq          = 399.78219838[MHz]
Irr_offset        = 5[ppm]
Clipped           = FALSE
Mod_return        = 1
Scans             = 16384
Total_scans       = 16384

X_90_width        = 9.82623[us]
X_acq_time        = 1.04333312[s]
X_angle           = 30[deg]
X_atn             = 5.8[dB]
X_pulse           = 3.27541[us]
Irr_atn_dec       = 21[dB]
Irr_atn_noe       = 21[dB]
Irr_noise         = WALTZ
Decoupling        = TRUE
Initial_wait      = 1[s]
Noe               = TRUE
Noe_time          = 2[s]
Recvr_gain        = 60
Relaxation_delay  = 2[s]
Repetition_time   = 3.04333312[s]
Temp_get          = 24.8[dC]
```

Figure S96: 1H NMR Spectrum of Compound 50.

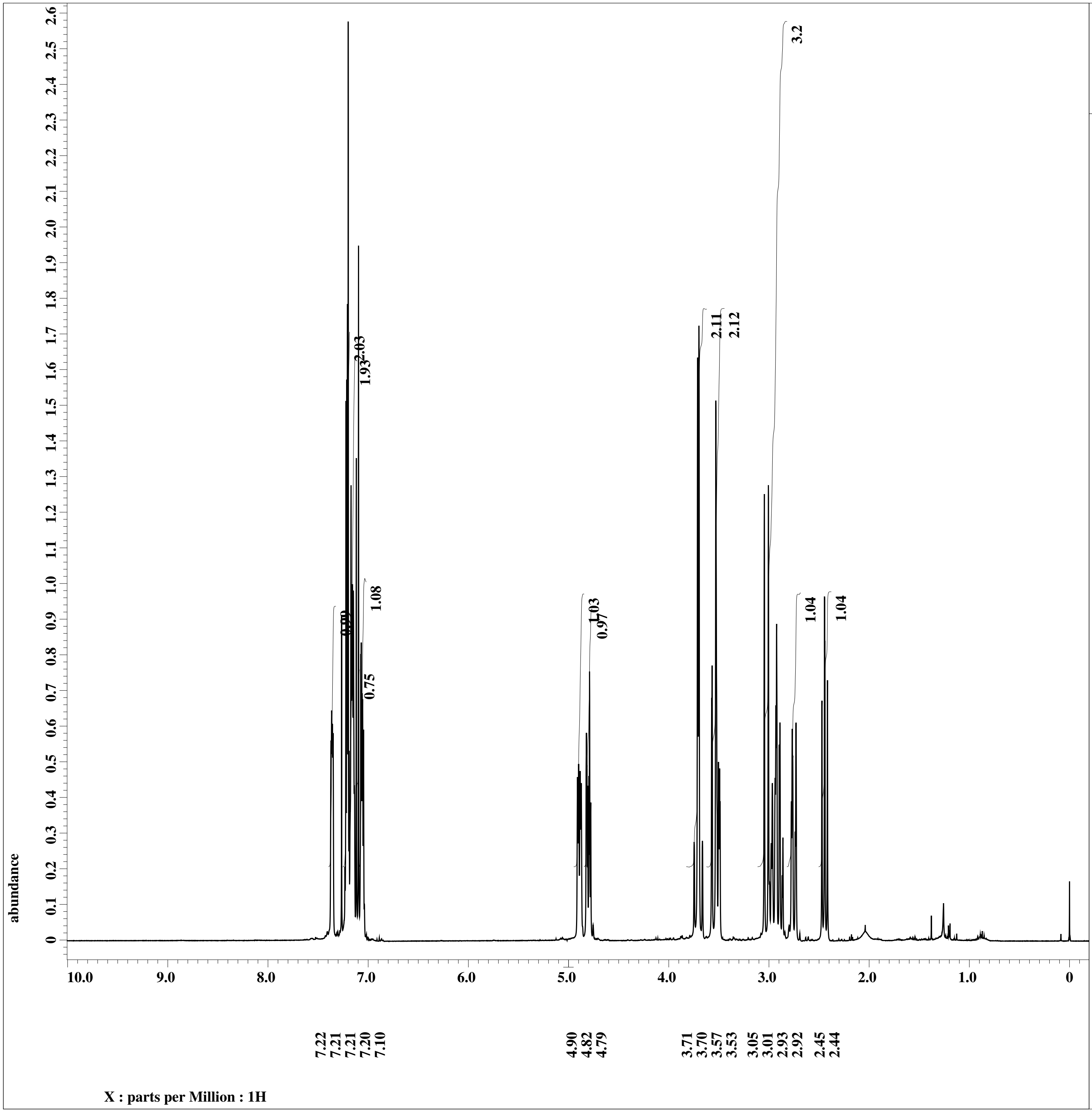


```
Filename          = RDK-I-013 redo column
Author            = chemlabs
Experiment        = single_pulse.ex2
Sample_id         = 001
Solvent           = CHLOROFORM-D
Creation_time     = 21-JUL-2017 11:06:03
Revision_time     = 22-JUL-2021 13:31:54
Current_time      = 22-JUL-2021 13:38:40

Comment           = single_pulse
Data_format       = 1D COMPLEX
Dim_size          = 13107
Dim_title         = 1H
Dim_units         = [ppm]
Dimensions        = X
Site              = ECS 400
Spectrometer      = JNM-ECS400

Field_strength    = 9.389766[T] (400[MHz]
X_acq_duration    = 2.18365952[s]
X_domain          = 1H
X_freq            = 399.78219838[MHz]
X_offset          = 5[ppm]
X_points          = 16384
X_prescans        = 1
X_resolution      = 0.45794685[Hz]
X_sweep           = 7.5030012[kHz]
Irr_domain        = 1H
Irr_freq          = 399.78219838[MHz]
Irr_offset        = 5[ppm]
Tri_domain        = 1H
Tri_freq          = 399.78219838[MHz]
Tri_offset        = 5[ppm]
Clipped           = FALSE
Mod_return        = 1
Scans             = 8
Total_scans       = 8

X_90_width        = 14.3[us]
X_acq_time        = 2.18365952[s]
X_angle           = 45[deg]
X_atn             = 3[dB]
X_pulse           = 7.15[us]
Irr_mode          = Off
Tri_mode          = Off
Dante_presat      = FALSE
Initial_wait      = 1[s]
Recvr_gain        = 28
Relaxation_delay  = 5[s]
Repetition_time   = 7.18365952[s]
Temp_get          = 23[dC]
```

Figure S97: 13C NMR Spectrum of Compound 50.

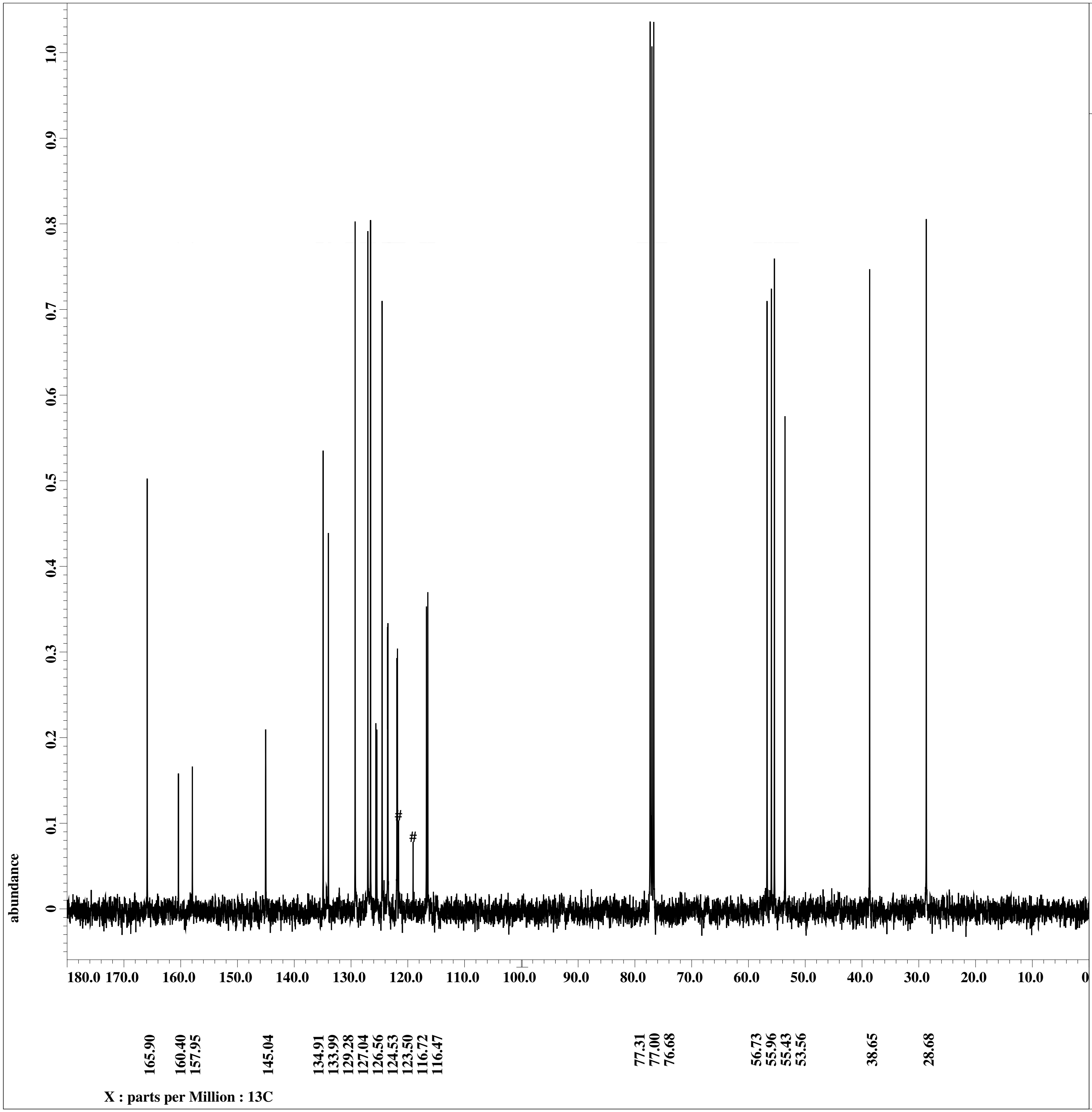

Filename	= RDK-I-013 redo column
Author	= chemlabs
Experiment	= single_pulse_dec
Sample_id	= 001
Solvent	= CHLOROFORM-D
Creation_time	= 21-JUL-2017 11:10:41
Revision_time	= 22-JUL-2021 13:43:24
Current_time	= 22-JUL-2021 13:44:36
Comment	= single pulse decouple
Data_format	= 1D COMPLEX
Dim_size	= 26214
Dim_title	= 13C
Dim_units	= [ppm]
Dimensions	= X
Site	= ECS 400
Spectrometer	= JNM-ECS400
Field_strength	= 9.389766[T] (400[MHz]
X_acq_duration	= 1.04333312[s]
X_domain	= 13C
X_freq	= 100.52530333[MHz]
X_offset	= 100[ppm]
X_points	= 32768
X_prescans	= 4
X_resolution	= 0.95846665[Hz]
X_sweep	= 31.40703518[kHz]
Irr_domain	= 1H
Irr_freq	= 399.78219838[MHz]
Irr_offset	= 5[ppm]
Clipped	= FALSE
Mod_return	= 1
Scans	= 68
Total_scans	= 68
X_90_width	= 9.82623[us]
X_acq_time	= 1.04333312[s]
X_angle	= 30[deg]
X_atn	= 5.8[dB]
X_pulse	= 3.27541[us]
Irr_atn_dec	= 21[dB]
Irr_atn_noe	= 21[dB]
Irr_noise	= WALTZ
Decoupling	= TRUE
Initial_wait	= 1[s]
Noe	= TRUE
Noe_time	= 2[s]
Recvr_gain	= 60
Relaxation_delay	= 2[s]
Repetition_time	= 3.04333312[s]
Temp_get	= 23.3[dC]

Figure S98: 1H NMR Spectrum of Compound 51.

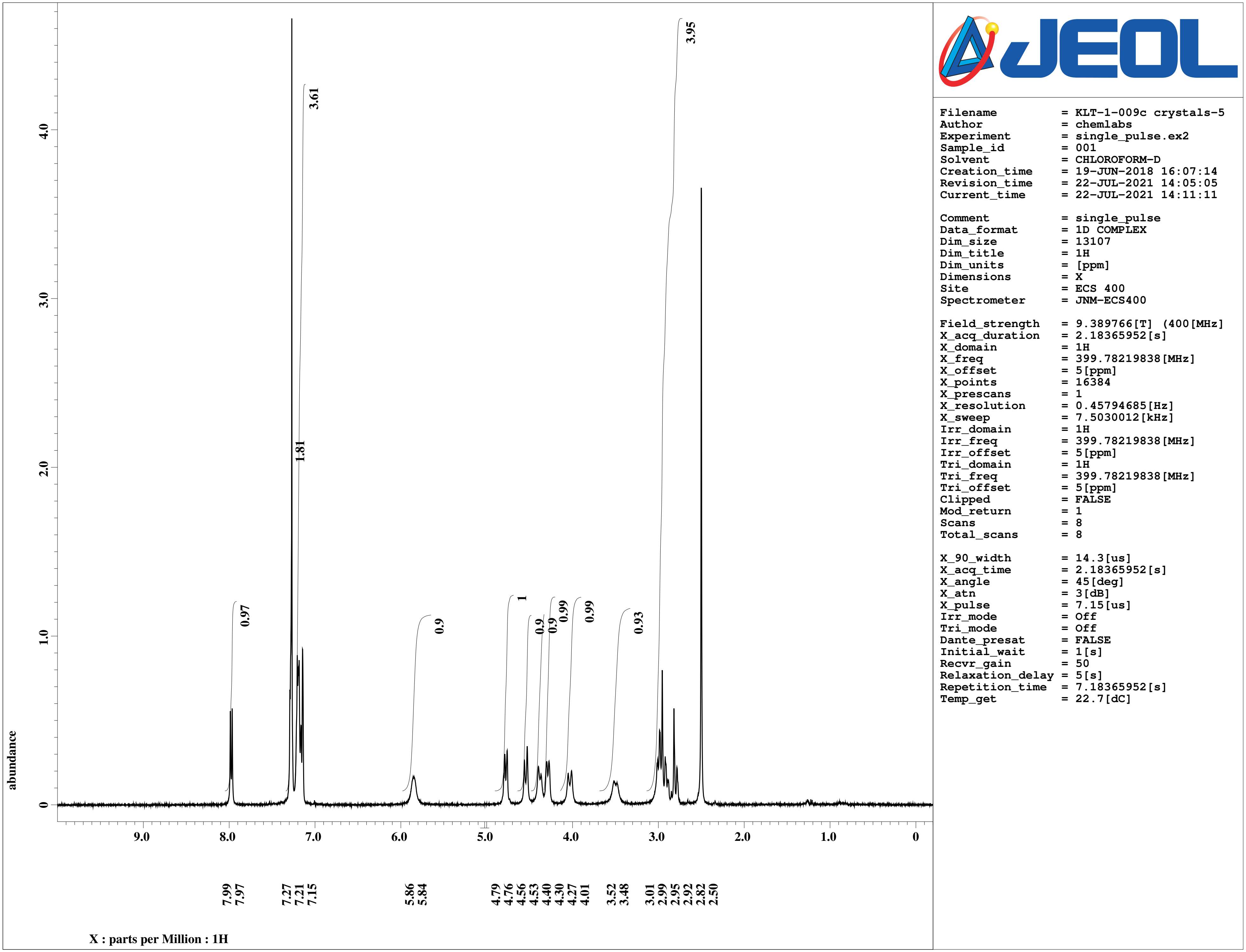

Figure S99: 13C NMR Spectrum of Compound 51.

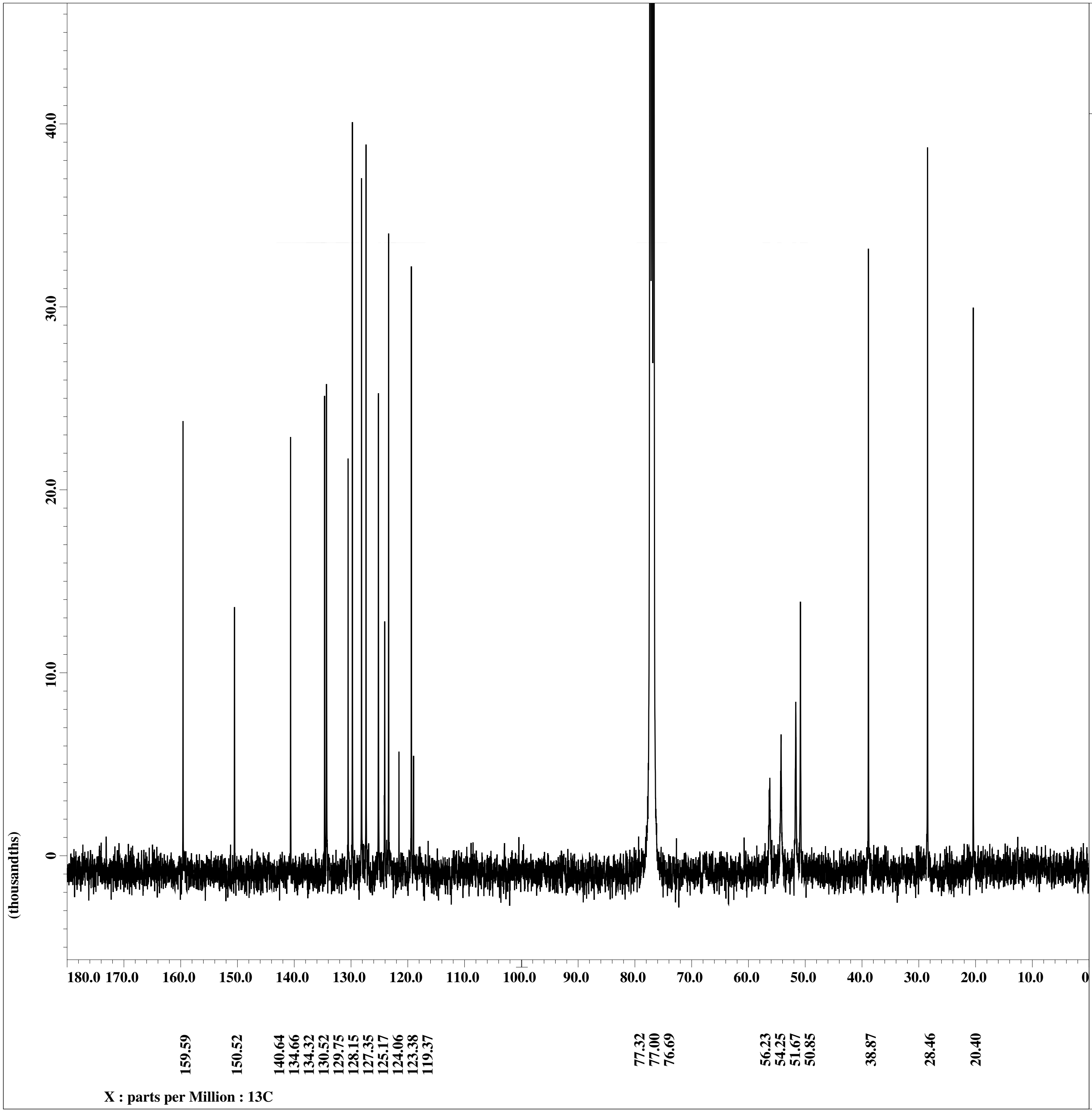

JEOL

Filename	= KLT-1-009c crystals 1
Author	= chemlabs
Experiment	= single_pulse_dec
Sample_id	= 001
Solvent	= CHLOROFORM-D
Creation_time	= 20-JUN-2018 05:59:26
Revision_time	= 22-JUL-2021 14:13:44
Current_time	= 22-JUL-2021 14:14:49
Comment	= single pulse decouple
Data_format	= 1D COMPLEX
Dim_size	= 26214
Dim_title	= 13C
Dim_units	= [ppm]
Dimensions	= X
Site	= ECS 400
Spectrometer	= JNM-ECS400
Field_strength	= 9.389766[T] (400[MHz]
X_acq_duration	= 1.04333312[s]
X_domain	= 13C
X_freq	= 100.52530333[MHz]
X_offset	= 100[ppm]
X_points	= 32768
X_prescans	= 4
X_resolution	= 0.95846665[Hz]
X_sweep	= 31.40703518[kHz]
Irr_domain	= 1H
Irr_freq	= 399.78219838[MHz]
Irr_offset	= 5[ppm]
Clipped	= FALSE
Mod_return	= 1
Scans	= 16384
Total_scans	= 16384
X_90_width	= 9.82623[us]
X_acq_time	= 1.04333312[s]
X_angle	= 30[deg]
X_atn	= 5.8[dB]
X_pulse	= 3.27541[us]
Irr_atn_dec	= 21[dB]
Irr_atn_noe	= 21[dB]
Irr_noise	= WALTZ
Decoupling	= TRUE
Initial_wait	= 1[s]
Noe	= TRUE
Noe_time	= 2[s]
Recvr_gain	= 60
Relaxation_delay	= 2[s]
Repetition_time	= 3.04333312[s]
Temp_get	= 22.8[dC]

Figure S100: 1H NMR Spectrum of Compound 52.

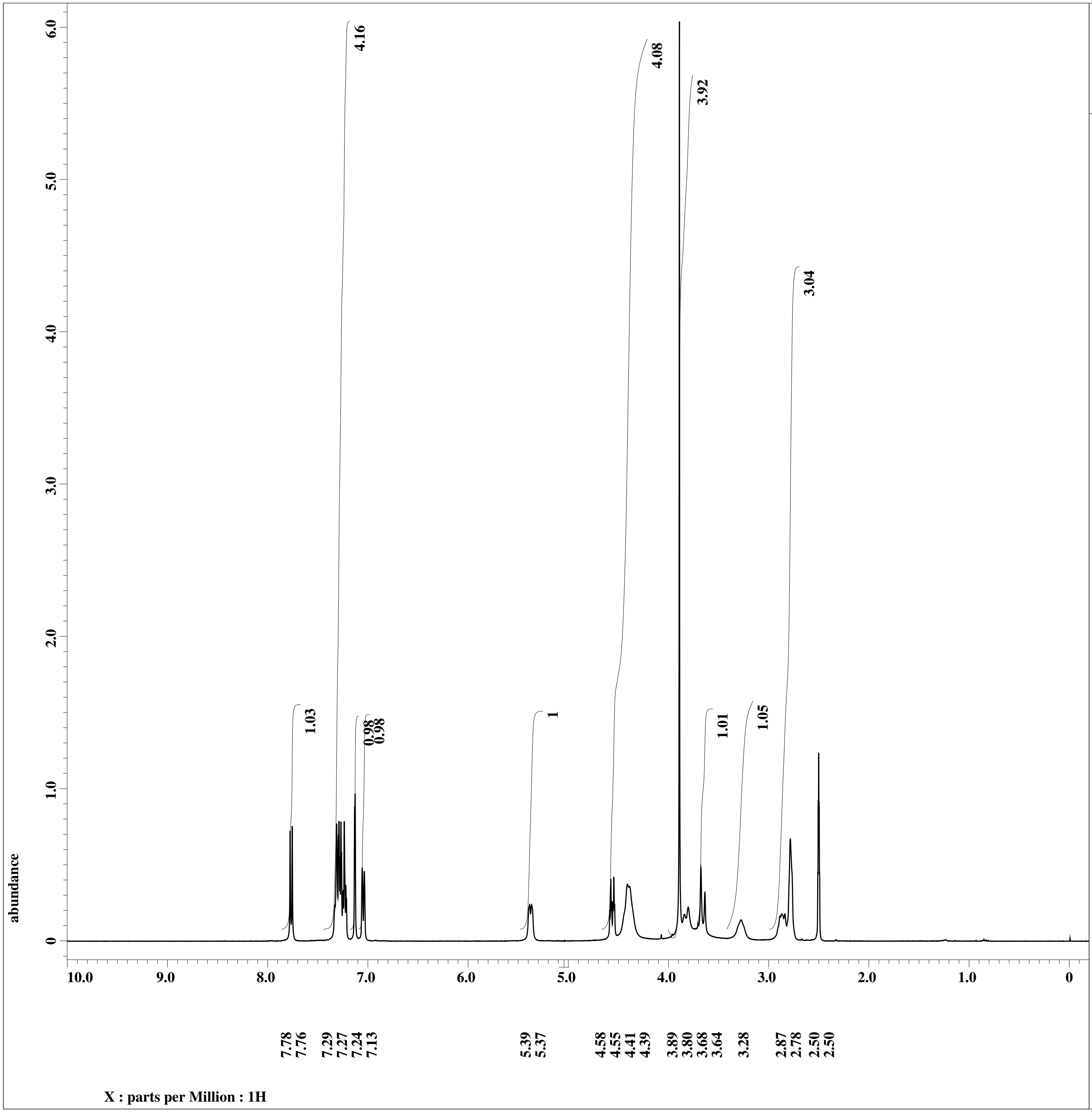


```
Filename           = JWH_I-023 1H NMR Work
Author             = russell research stud
Experiment         = single_pulse.ex2
Sample_id          = S#722291
Solvent            = DMSO-D6
Creation_time      =  5-OCT-2019 17:38:16
Revision_time      = 22-JUL-2021 14:20:11
Current_time       = 22-JUL-2021 14:22:22

Data_format        = 1D COMPLEX
Dim_size           = 13107
Dim_title          = 1H
Dim_units          = [ppm]
Dimensions         = X
Site               = ECS 400
Spectrometer       = JNM-ECS400

Field_strength     = 9.389766[T] (400[MHz]
X_acq_duration     = 2.18365952[s]
X_domain           = 1H
X_freq             = 399.78219838[MHz]
X_offset           = 5[ppm]
X_points           = 16384
X_prescans         = 1
X_resolution       = 0.45794685[Hz]
X_sweep            = 7.5030012[kHz]
Irr_domain         = 1H
Irr_freq           = 399.78219838[MHz]
Irr_offset         = 5[ppm]
Tri_domain         = 1H
Tri_freq           = 399.78219838[MHz]
Tri_offset         = 5[ppm]
Clipped            = FALSE
Mod_return         = 1
Scans              = 8
Total_scans        = 8

X_90_width         = 14.3[us]
X_acq_time         = 2.18365952[s]
X_angle            = 45[deg]
X_atn              = 3[dB]
X_pulse            = 7.15[us]
Irr_mode           = Off
Tri_mode           = Off
Dante_presat       = FALSE
Initial_wait       = 1[s]
Recvr_gain         = 34
Relaxation_delay   = 5[s]
Repetition_time    = 7.18365952[s]
Temp_get           = 22.4[dC]
```

Figure S101: 13C NMR Spectrum of Compound 52.

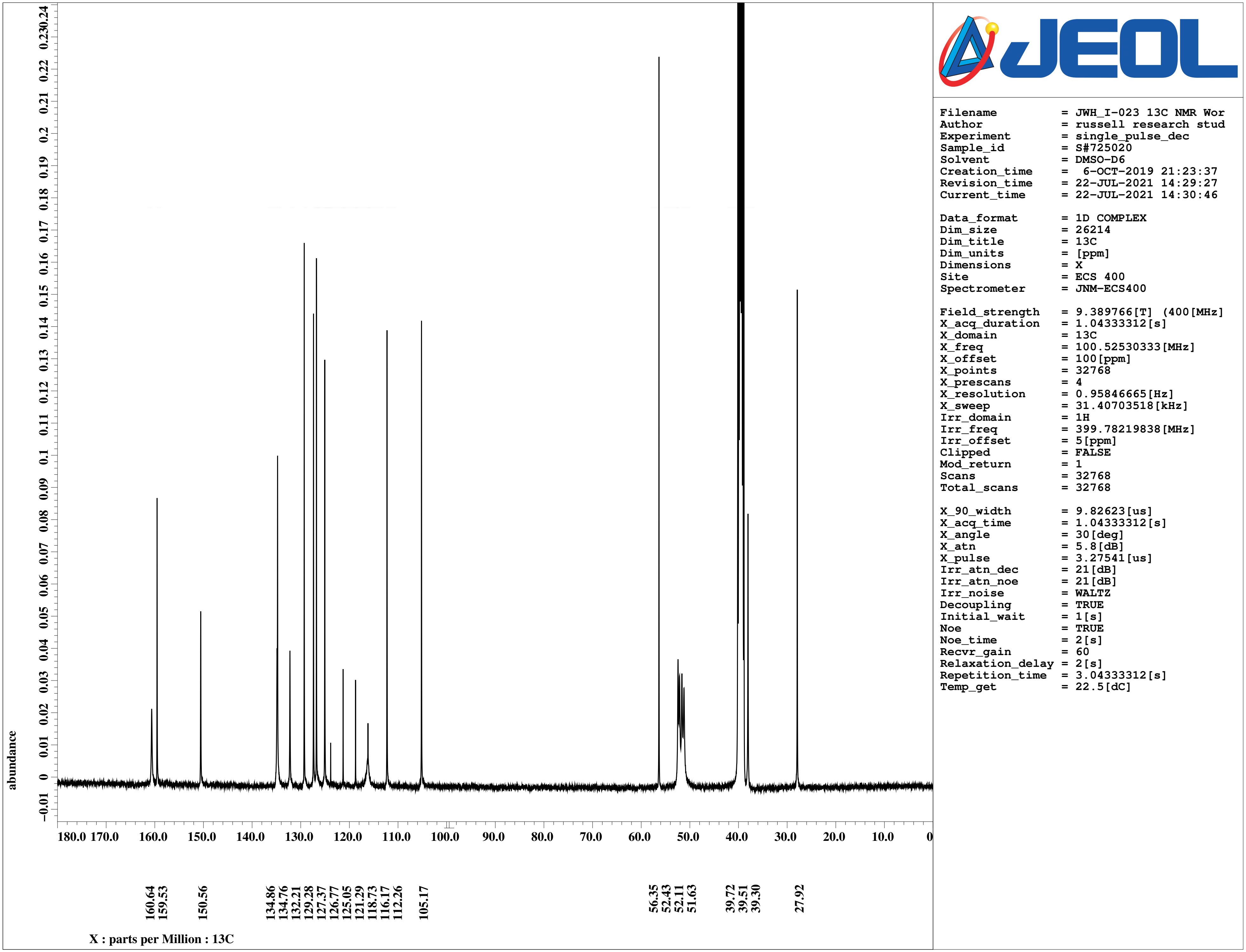

Figure S102: 1H NMR Spectrum of Compound 53.

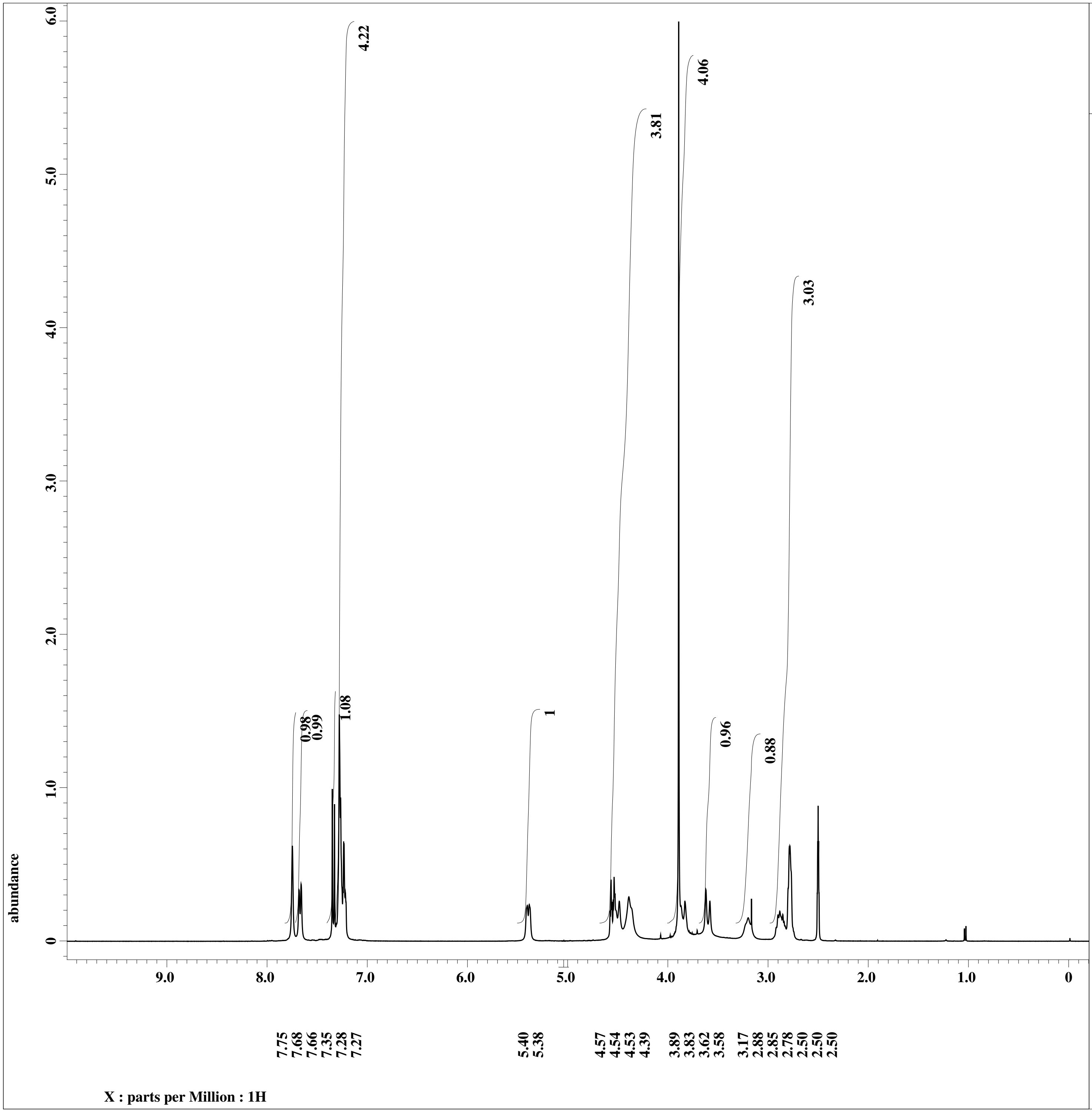


```
Filename          = RDK-I-017 DMSO 1H-con
Author            = chemlabs
Experiment        = single_pulse.ex2
Sample_id         = 001
Solvent           = DMSO-D6
Creation_time     = 28-JUL-2017 14:19:20
Revision_time     = 22-JUL-2021 14:34:00
Current_time      = 22-JUL-2021 14:36:54

Comment           = single_pulse
Data_format       = 1D COMPLEX
Dim_size          = 13107
Dim_title         = 1H
Dim_units         = [ppm]
Dimensions        = X
Site              = ECS 400
Spectrometer      = JNM-ECS400

Field_strength    = 9.389766[T] (400[MHz]
X_acq_duration    = 2.18365952[s]
X_domain          = 1H
X_freq            = 399.78219838[MHz]
X_offset          = 5[ppm]
X_points          = 16384
X_prescans        = 1
X_resolution      = 0.45794685[Hz]
X_sweep           = 7.5030012[kHz]
Irr_domain        = 1H
Irr_freq          = 399.78219838[MHz]
Irr_offset        = 5[ppm]
Tri_domain        = 1H
Tri_freq          = 399.78219838[MHz]
Tri_offset        = 5[ppm]
Clipped           = FALSE
Mod_return        = 1
Scans             = 8
Total_scans       = 8

X_90_width        = 14.3[us]
X_acq_time        = 2.18365952[s]
X_angle           = 45[deg]
X_atn             = 3[dB]
X_pulse           = 7.15[us]
Irr_mode          = Off
Tri_mode          = Off
Dante_presat      = FALSE
Initial_wait      = 1[s]
Recvr_gain        = 30
Relaxation_delay  = 5[s]
Repetition_time   = 7.18365952[s]
Temp_get          = 25[dC]
```

Figure S103: 13C NMR Spectrum of Compound 53.

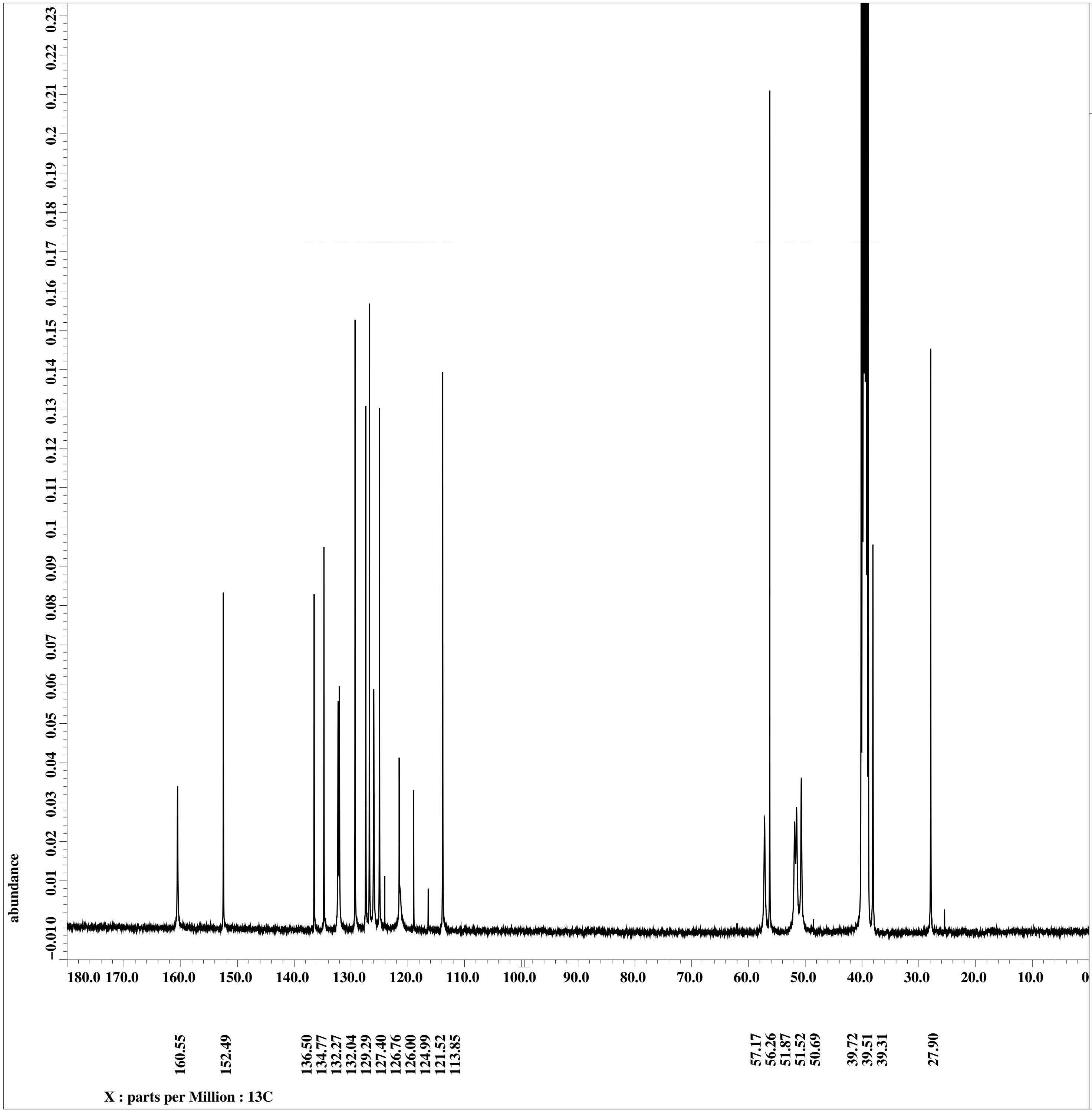


```
Filename          = RDK-I-017 DMSO 13C-co
Author            = chemlabs
Experiment        = single_pulse_dec
Sample_id         = 001
Solvent           = DMSO-D6
Creation_time     = 29-JUL-2017 18:02:01
Revision_time     = 22-JUL-2021 14:41:04
Current_time      = 22-JUL-2021 14:43:21

Comment           = single pulse decouple
Data_format       = 1D COMPLEX
Dim_size          = 26214
Dim_title         = 13C
Dim_units         = [ppm]
Dimensions        = X
Site              = ECS 400
Spectrometer      = JNM-ECS400

Field_strength    = 9.389766[T] (400[MHz]
X_acq_duration    = 1.04333312[s]
X_domain          = 13C
X_freq            = 100.52530333[MHz]
X_offset          = 100[ppm]
X_points          = 32768
X_prescans        = 4
X_resolution      = 0.95846665[Hz]
X_sweep           = 31.40703518[kHz]
Irr_domain        = 1H
Irr_freq          = 399.78219838[MHz]
Irr_offset        = 5[ppm]
Clipped           = FALSE
Mod_return        = 1
Scans             = 32768
Total_scans       = 32768

X_90_width        = 9.82623[us]
X_acq_time        = 1.04333312[s]
X_angle           = 30[deg]
X_atn             = 5.8[dB]
X_pulse           = 3.27541[us]
Irr_atn_dec       = 21[dB]
Irr_atn_noe       = 21[dB]
Irr_noise         = WALTZ
Decoupling        = TRUE
Initial_wait      = 1[s]
Noe               = TRUE
Noe_time          = 2[s]
Recvr_gain        = 60
Relaxation_delay  = 2[s]
Repetition_time   = 3.04333312[s]
Temp_get          = 25[dC]
```
